# Associations between breast cancer survivorship and adverse mental health outcomes: A matched population-based cohort study in the United Kingdom

**DOI:** 10.1371/journal.pmed.1003504

**Published:** 2021-01-07

**Authors:** Helena Carreira, Rachael Williams, Garth Funston, Susannah Stanway, Krishnan Bhaskaran

**Affiliations:** 1 Faculty of Epidemiology and Population Health, London School of Hygiene & Tropical Medicine, London, United Kingdom; 2 Clinical Practice Research Datalink (CPRD), Medicines and Healthcare products Regulatory Agency, London, United Kingdom; 3 Department of Public Health and Primary Care, University of Cambridge, Cambridge, United Kingdom; 4 Department of Medicine, The Royal Marsden NHS Foundation Trust, London and Surrey, United Kingdom; Harvard Medical School, UNITED STATES

## Abstract

**Background:**

Breast cancer is the most common cancer diagnosed in women globally, and 5-year net survival probabilities in high-income countries are generally >80%. A cancer diagnosis and treatment are often traumatic events, and many women struggle to cope during this period. Less is known, however, about the long-term mental health impact of the disease, despite many women living several years beyond their breast cancer and mental health being a major source of disability in modern societies. The objective of this study was to quantify the risk of several adverse mental health–related outcomes in women with a history of breast cancer followed in primary care in the United Kingdom National Health Service, compared to similar women who never had cancer.

**Methods and findings:**

We conducted a matched cohort study using data routinely collected in primary care across the UK to quantify associations between breast cancer history and depression, anxiety, and other mental health–related outcomes. All women with incident breast cancer in the Clinical Practice Research Datalink (CPRD) GOLD primary care database between 1988 and 2018 (*N* = 57,571, mean = 62 ± 14 years) were matched 1:4 to women with no prior cancer (*N* = 230,067) based on age, primary care practice, and eligibility of the data for linkage to hospital data sources. Cox models were used to estimate associations between breast cancer survivorship and each mental health–related outcome, further adjusting for diabetes, body mass index (BMI), and smoking and drinking status at baseline. Breast cancer survivorship was positively associated with anxiety (adjusted hazard ratio (HR) = 1.33; 95% confidence interval (CI): 1.29–1.36; *p* < 0.001), depression (1.35; 1.32–1.38; *p* < 0.001), sexual dysfunction (1.27; 1.17–1.38; *p* < 0.001), and sleep disorder (1.68; 1.63–1.73; *p* < 0.001), but not with cognitive dysfunction (1.00; 0.97–1.04; *p* = 0.88). Positive associations were also found for fatigue (HR = 1.28; 1.25–1.31; *p* < 0.001), pain (1.22; 1.20–1.24; *p* < 0.001), receipt of opioid analgesics (1.86; 1.83–1.90; *p* < 0.001), and fatal and nonfatal self-harm (1.15; 0.97–1.36; *p* = 0.11), but CI was wide, and the relationship was not statistically significant for the latter. HRs for anxiety and depression decreased over time (*p*-interaction <0.001), but increased risks persisted for 2 and 4 years, respectively, after cancer diagnosis. Increased levels of pain and sleep disorder persisted for 10 years. Younger age was associated with larger HRs for depression, cognitive dysfunction, pain, opioid analgesics use, and sleep disorders (*p*-interaction <0.001 in each case). Limitations of the study include the potential for residual confounding by lifestyle factors and detection bias due to cancer survivors having greater healthcare contact.

**Conclusions:**

In this study, we observed that compared to women with no prior cancer, breast cancer survivors had higher risk of anxiety, depression, sleep problems, sexual dysfunction, fatigue, receipt of opioid analgesics, and pain. Relative risks estimates tended to decrease over time, but anxiety and depression were significantly increased for 2 and 4 years after breast cancer diagnosis, respectively, while associations for fatigue, pain, and sleep disorders were elevated for at least 5–10 years after diagnosis. Early diagnosis and increased awareness among patients, healthcare professionals, and policy makers are likely to be important to mitigate the impacts of these raised risks.

## Abstract 2

In a matched cohort study, Helena Carreira and colleagues investigate risks of pain, fatigue, and mental health-related outcomes among women with a history of breast cancer.

## Author summary

**Why was this study done?:**

- More than 570,000 women are carrying on with their lives after having been diagnosed and treated for breast cancer in the UK. This number is expected to increase.
- Many women feel anxious and/or depressed while undergoing the cancer treatments, but it is less clear how their mental health fares in the longer term. The evaluation of the long-term mental health of these women is important to understand what their needs are and plan health services that are able to mitigate the long-term consequences of cancer.
- We aimed to estimate the risk of a range of mental health–related outcomes in a population-based sample of breast cancer survivors in the UK, compared to women with no prior cancer.

**What did the researchers do and find?:**

- This study compared the risk of several adverse mental health–related outcomes in 57,571 women with history of breast cancer and 230,067 women with no history of cancer followed in primary care in the UK.
- The results suggest that breast cancer survivorship is associated with raised risks of anxiety, depression, fatigue, sleep disorders, pain, and sexual dysfunction, persisting well into the survivorship period.

**What do these findings mean?:**

- Breast cancer survivorship is associated with raised risk of several adverse mental health–related outcomes. For all outcomes, the risk was particularly increased around diagnosis and during the period of the main treatments for breast cancer.
- Early diagnosis and management of these conditions is likely to be key to mitigate the impact on the long-term health of cancer survivors.
- Future studies might investigate whether screening breast cancer patients for mental disorders during this period could help identify patients struggling to cope and enable interventions to help reduce the long-term burden of the disease.

## Introduction

Large numbers of women around the world are currently living beyond a breast cancer diagnosis, including over 2.9 million in the United States and 570,000 in the UK, with numbers projected to rise further [1,2]. Women with a history of breast cancer may experience long-term physical consequences of treatment, worries about cancer recurrence, employment or financial difficulties, and other challenges, with potential negative consequences for broader mental health [3,4]. However, little evidence is available from large-scale population-based studies to quantify the impact of a history of cancer on long-term mental health.

In a recent systematic review [5], anxiety and depression were found to be more common in breast cancer survivors than in women with no cancer history, but, evidence was often drawn from studies at high risk of selection and information bias, likely to be confounded by age and socioeconomic status, and lacking generalisability. Only a few population-based studies have been done, finding conflicting effect sizes, with relative risks of clinically diagnosed depression or anxiety ranging from no association to a doubling of risk in cancer survivors [5–7]. Increased risks of other mental health outcomes including suicide and neurocognitive and sexual dysfunctions have also been observed; existing evidence on sleep disturbance is insufficient to draw conclusions [5]. The overall burden of mental health disorders is remarkably high, particularly in high-income settings [8], and the efficient planning and delivery of cancer support and mental health services that suit the needs of the largest group of cancer survivors in the population requires timely and robust estimates on the risk of clinically assessed outcomes at population level.

We therefore aimed to quantify the associations between breast cancer history and the primary outcomes of anxiety and depression and the secondary outcomes of cognitive dysfunction, fatigue, sleep disorder, sexual dysfunction, fatal and nonfatal self-harm, pain, and opioid analgesics prescription using population-based electronic health records data from UK primary care. Pain and fatigue were included due to their likely links with both cancer history and mental health.

## Methods

### Ethics statement

This study was conducted following a priori defined methods and is reported as per the Strengthening the Reporting of Observational Studies in Epidemiology (STROBE) guideline (S1 Checklist).

The study protocol was approved by the Independent Scientific Advisory Committee (ISAC) at the Medicines and Healthcare products Regulatory Agency (S1 Protocol, ref 18_253) and received favourable ethical opinion from the Research Ethics Committee at the London School of Hygiene and Tropical Medicine (ref 16225). Individual participant consent is not required.

### Study design and data sources

We conducted a matched cohort study using data from the UK Clinical Practice Research Datalink (CPRD) GOLD primary care database (July 2018 version), which contains anonymised electronic health records data from 761 general practitioners’ (GPs) practices in England, Wales, Northern Ireland, and Scotland [9] and is broadly representative of the UK population in terms of age, sex, and ethnicity [10]. All patients were assigned a measure of relative deprivation (proxy for socioeconomic status) based on their GP practice postcode Index of Multiple Deprivation (IMD) [11–14].

Approximately 75% of the GP practices in England consent to have their data linked to other sources of data (linkage is not allowed for other UK jurisdictions). For eligible patients, the patient-level primary care data were linked, using deterministic methods [15], to the Office for National Statistics (ONS) mortality data [16], the Hospital Episodes Statistics–Admitted Patient Care (HES-APC) database [17], and the patient postcode level of IMD [11]. Linked ONS mortality data were used to identify cases of suicide; HES-APC data were used to define self-harm and improve outcome ascertainment in sensitivity analyses.

### Study populations

The exposed cohort included all women (≥18 years) with an incident diagnosis of breast cancer (not including in situ tumours; code list available in Table A in S1 Codelists) recorded in their primary care record between database inception (1987) and July 2018. To ensure breast cancer diagnoses were incident, we required 12 months of follow-up in CPRD prior to the diagnosis. We excluded women with severe mental or neurological disorders (i.e., schizophrenia and other psychotic disorders, bipolar disorders, neurocognitive disorders, and substance-related disorders) or another cancer diagnosis (except nonmelanoma skin cancer) prior to breast cancer. To ensure that all mental health–related outcome events were incident, we excluded from each outcome-specific analysis patients who had evidence of that outcome in the year before the breast cancer diagnosis (index date). Patients with an outcome last recorded >1 year before the breast cancer diagnosis were not excluded, and the condition was assumed to be in remission.

For each breast cancer survivor, we randomly selected 4 control women with no history of cancer (except nonmelanoma skin cancer) at the index date (date of diagnosis), matched to the cancer survivor on age (within a 3-year range), primary care practice, and eligibility of the data for hospital data linkage (to enable a sensitivity analyses among patients with linked data). Ethnicity was missing for approximately 60% of women and not included in the matching process. Women in the exposed cohort were eligible for selection as controls up to the date of breast cancer diagnosis. Similar to the exposed group, women in the unexposed cohort had ≥12 months of uninterrupted prior registration before the index date (date of breast cancer diagnosis of the matched case). Exclusion criteria were as for the exposed.

### Outcomes

The primary outcomes were anxiety and depression. Anxiety included generalised anxiety disorder, panic disorder, social anxiety, agoraphobia, obsessive–compulsive disorders, and stress-related disorders with anxiety (see S1 Methods for more details). Depression included major depressive disorder, dysthymia, and stress-related disorders with depressed mood. Mixed anxiety and depression was included in both definitions. Outcomes were identified by either a diagnostic Read code for the conditions named above, or a Read code for a symptom (e.g., low mood) accompanied by a prescription within 90 days for an antidepressant, for depression, or an anxiolytic or relevant antidepressant, for anxiety (S1 Methods). The Read code lists (Tables B–P in S1 Codelists) were informed by a systematic review on the topic [15] and revised by a practicing GP (author GF).

Secondary outcomes were cognitive dysfunction, sleep disorder, sexual dysfunction, fatal and nonfatal self-harm, fatigue, pain, and opioid analgesics prescription. Detailed definitions are provided in S1 Methods. Briefly, cognitive dysfunction was identified as a Read code for cognitive impairment, including dementia, or a dementia-specific drug prescription. Sleep disorder was defined as a Read code for diagnosis, or a combination of symptoms codes and prescriptions of anxiolytics/hypnotics within 90 days. Fatigue, sexual dysfunction, and pain were defined using Read codes alone. Prescriptions of opioid analgesics were identified from primary care prescription data. Fatal and nonfatal self-harm was ascertained using a validated list of Read codes [18], updated for this study. Suicide was ascertained from linked official death registration data and was defined by the International Statistical Classification of Diseases and Related Health Problems 10th Revision (ICD-10) codes X60-X84 and Y10-34, excluding Y33.9 (verdict pending). All Read codelists are provided in Tables B–P in S1 Codelists.

### Covariates

Diabetes mellitus, body mass index (BMI), and alcohol and smoking status, all measured at (or near) the index date, were considered as potential confounders (definitions in S1 Methods). Deprivation and cardiovascular morbidity were studied as potential effect modifiers.

### Statistical analysis

The analysis plan was defined prior to the start of the study and is available in S1 Protocol. Incidence rates were calculated separately for each outcome in each cohort. Follow-up started at the index date (date of cancer diagnosis in the exposed cohort; unexposed patient took the same index date as their matched case) and terminated at the earliest of mental health–related outcome observed, cancer diagnosis other than breast in the exposed cohort, any cancer diagnosis in the comparison cohort, death, transference out of the practice, and last data collection for the practice.

The association between breast cancer survivorship and each mental health–related outcome was quantified using Cox regression models (per outcome), with time since index as the underlying timescale, and stratified on matched set to account for matching. Stratification of the baseline hazard is a valid way of handling multicentre and matched data if the clustering is of no intrinsic interest [19,20]. Hospitalisations data are considered the gold standard for capturing self-harm [21], and thus all analyses of fatal and nonfatal self-harm were restricted to individuals with linked HES data. Hazard ratios (HR) adjusted for diabetes mellitus at baseline (yes/no), BMI (restricted cubic spline), smoking status (nonsmoker, current smoker, and former smoker) and drinking status (never drinker, current drinker, and former drinker), were estimated for all outcomes in complete cases analyses; multiple imputation was not used, as the missingness was considered likely to be missing not at random in a primary care setting [22,23], and complete case analysis minimises bias in this situation, providing missingness is conditionally independent of the outcome [24]. In the protocol, we planned to include BMI as categorical covariate; following peer-review comments, the final models included BMI as a continuous variable modelled using restricted cubic splines. The probability of type I error was set to 5%; all tests were 2-sided. Robust standard errors were used to calculate 95% confidence intervals (CIs).

We assessed the potential for effect modification by age group at index date (18 to 34; 35 to 44; 45 to 54; 55 to 64; 65 to 74; 75 to 84; ≥85 years), practice postcode-linked quintile of IMD, calendar period of index date (1988 to 1994; 1995 to 1999; 2000 to 2004; 2005 to 2009; 2010 to 2014; 2015 to 2018), follow-up time (1-year interval up to 5 years, 5 to 10 years, and >10 years, an implicit test of proportional hazards), cardiovascular comorbidity (yes/no), and history of the mental health–related outcome <1 year before index date (yes/no), by fitting interaction terms between the exposure and these variables.

### Sensitivity analysis

We carried out several sensitivity analyses. For each mental health–related outcome, we repeated the analysis excluding patients with lifetime history of the outcome before the index date. To account for the potential that breast cancer survivors may have more contact with health services, we ran a sensitivity analysis including only patients that had a consultation with their GP in the year before the index date. For the subset of patients for whom linked data were available (i.e., approximately 50% of the patients in England), we ran analyses using linked HES-APC data to improve outcome ascertainment and with adjustment for patient- (rather than practice-) level IMD. For anxiety, depression, and sleep disorders, we ran analyses with definitions that are expected to have high specificity, to assess the impact of the code list in our results. For opioid analgesics, we reran the analysis excluding codeine, which can be prescribed for its antitussive or antidiarrheal properties. For fatal and nonfatal self-harm, we ran analyses separately for self-harm and completed suicide.

## Results

**Fig 1:**
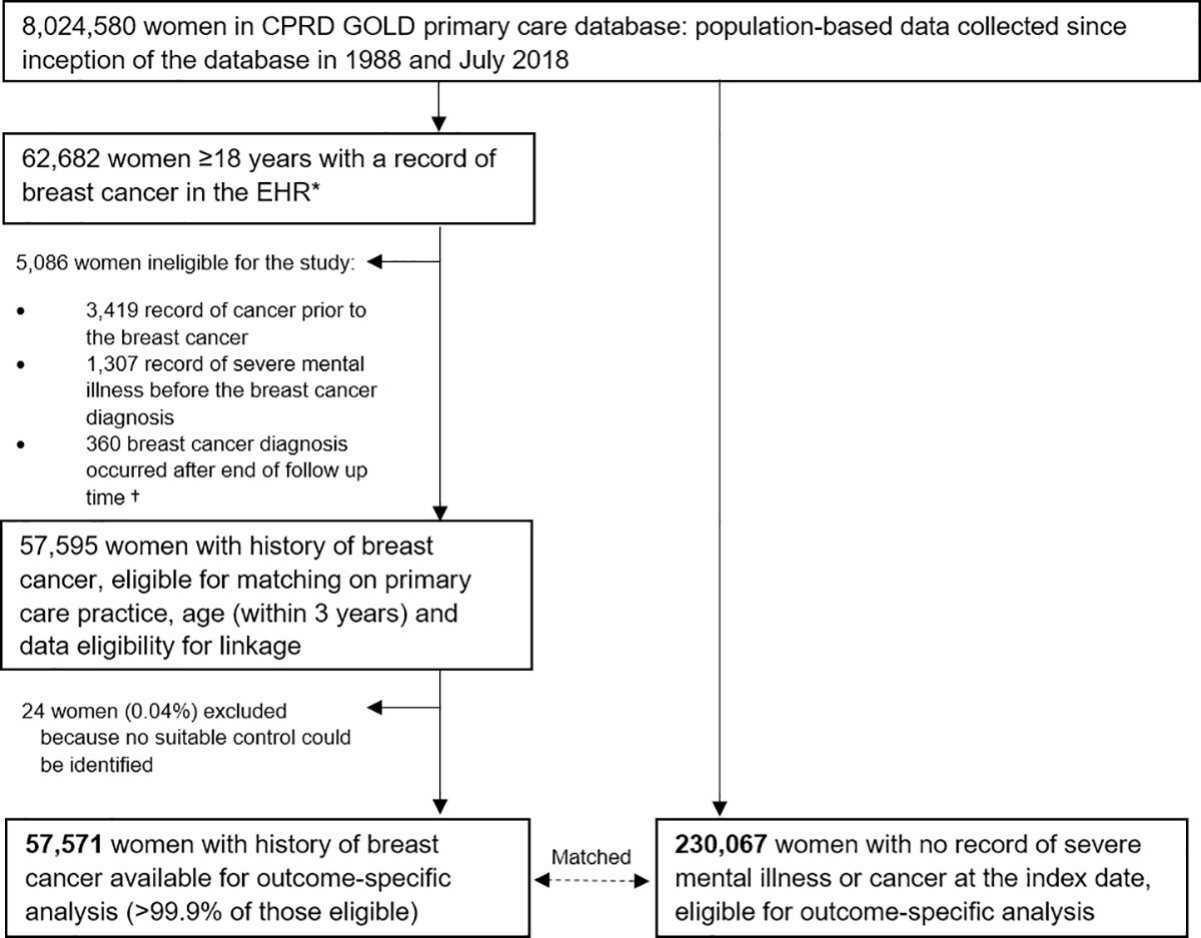
Flowchart of the selection of the cohorts used in analyses. CPRD, Clinical Practice Research Datalink; EHR, electronic health record. *Women with research quality follow-up, as defined by CPRD based on systematic checks for data quality at both patient and practice level. ^†^Almost all had a first record of breast cancer a few days after the recorded date of death.

A total of 57,571 women with history of breast cancer and 230,067 women with no history of cancer were included in the study (Fig 1). Approximately 20% of all participants had anxiety recorded >1 year before the index date (date of cancer diagnosis in the exposed group; controls took the same date as matched case), and 29% had history of depression (Table 1). Overall median follow-up time was 4.5 years in the exposed group (interquartile range (IQR): 1.9 to 8.5 years) and 5.2 years in the comparison group (IQR: 2.2 to 9.3 years). Moreover, 11,790 breast cancer survivors (24%) and 55,609 women in the comparison group (20%) had ≥10 years of follow-up. Mental health–related outcome-specific follow-up time and person-time at risk are included in S1A–S1C Table.

**Table 1:** Characteristics of the study participants (1988–2018)*.

	Women with history of breast cancer		Women with no history of cancer	
	No.	%	No.	%
**All participants**	**57,571**	100.00	**230,067**	100.00
**Sociodemographic**				
** Age group at index date (years)**^**†**^				
** **18–34	781	1.36	3,125	1.36
** **35–44	4,768	8.28	19,059	8.28
** **45–54	13,039	22.5	52,114	22.65
** **55–64	14,436	25.08	57,707	25.08
** **65–74	12,361	21.47	49,395	21.47
** **75–84	8,386	14.57	33,524	14.57
** **85+	3,800	6.60	15,143	6.58
** Calendar period of index date**^**‡**^				
** **1988–1994	2,656	4.61	10,619	4.62
** **1995–1999	4,796	8.33	19,149	8.32
** **2000–2004	11,590	20.13	46,302	20.13
** **2005–2009	16,381	28.45	65,480	28.46
** **2010–2014	15,733	27.33	62,862	27.32
** **2015–2018	6,415	11.14	25,655	11.15
** Ethnicity**				
** **White	21,187	36.80	86,187	37.46
** **South Asian	411	0.71	2,247	0.98
** **Black	271	0.47	1,384	0.60
** **Other and mixed	221	0.38	1,228	0.53
** **Unknown	35,481	61.63	139,021	60.43
** Practice postcode quintile of IMD**				
** **1 (least deprived)	11,381	19.78	45,502	19.78
** **2	9,913	17.22	39,618	17.22
** **3	11,820	20.53	47,239	20.53
** **4	11,736	20.38	46,899	20.39
** **5 (most deprived)	12,721	22.08	50,809	22.10
**Lifestyle**				
** BMI (kg/m**^**2**^**) at index date**				
** **<18.50	908	1.58	4,561	1.98
** **18.50–24.99	20,958	36.40	85,030	36.96
** **25.00–29.99	17,565	30.51	69,052	30.01
** **30.00–34.99	8,666	15.05	32,401	14.08
** **35.00–39.99	3,302	5.74	12,549	5.45
** **≥40.00	1,490	2.59	6,350	2.76
** **Unknown	4,682	8.13	20,124	8.75
** Alcohol intake at index date**				
** **Never drinker	7,780	13.51	35,065	15.24
** **Current drinker	40,438	70.24	156,604	68.07
** **Former drinker	4,436	7.71	17,181	7.47
** **Unknown	4,917	8.54	21,217	9.22
** Smoking status at index date**				
** **Nonsmoker	30,452	52.89	122,985	53.46
** **Current smoker	9,565	16.61	39,986	17.38
** **Former smoker	16,343	28.39	60,604	26.34
** **Unknown	1,211	2.10	6,492	2.82
**Comorbidities at index date**				
** **Diabetes	3,844	6.68	14,030	6.10
** **History of coronary heart disease or stroke	4,648	8.07	18,523	8.05
**Prior history of (>1 year before index date)**^**§**^				
** **Anxiety	11,986	20.82	45,482	19.77
** **Depression	16,771	29.13	65,628	28.53
** **Cognitive dysfunction^¥^	-	-	-	-
** **Fatigue	11,200	19.45	42,578	18.51
** **Sleep disorder	7,221	12.55	27,528	11.97
** **Opioid prescribing	12,761	22.17	47,765	20.76
** **Pain	44,752	77.73	173,112	75.24
** **Sexual dysfunction	1,670	2.90	6,479	2.82
** **Self-harm	1,652	2.87	6,847	2.98

BMI, body mass index; IMD, Index of Multiple Deprivation.

* Refers to all patients potentially eligible for analyses. The number of patients included in analyses varied by outcome because we excluded women who had that particular outcome in the year before the index date, as we assumed that these were likely to still be under treatment at the index date.

† Women with no history of cancer were individually matched by age (within a 3-year age range) to women in the exposed group.

‡ For women in the exposed cohort, the index date was the date of their cancer diagnosis. Women in the comparison group were matched to exposed women on age and primary care practice and took the same index date as their matched case.

§ Refers to women who had the outcome recorded at >1 year before the index date. Women who had the outcome in the year before the index date were excluded from the cohort.

¥ Patients with a record of cognitive dysfunction at any point prior to the index date were excluded, as changes in cognitive function are often nonreversible.

**Fig 2:**
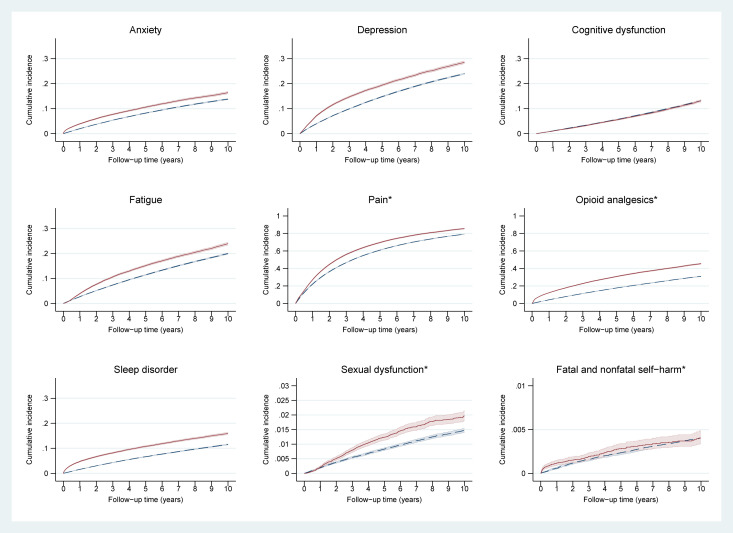
Incidence of adverse mental health outcomes, fatigue, and pain in breast cancer survivors and in women who did not have cancer. Full red line: breast cancer survivors; dashed blue line: women who never had cancer. *Please note the different scale used in this graph.

Table 2 shows the minimally adjusted (matching factors) and fully adjusted (for diabetes, smoking, drinking, and BMI) associations between breast cancer survivorship and each adverse mental health outcome. Minimally adjusted and fully adjusted associations were very similar. Compared with cancer-free controls, breast cancer survivorship was positively associated with the primary outcomes of anxiety (fully adjusted HR = 1.33, 95% CI: 1.29 to 1.36, *p* < 0.001) and depression (1.35, 1.32 to 1.38, *p* < 0.001), as well as the secondary outcomes of sexual dysfunction (1.27; 1.17 to 1.38; *p* < 0.001), sleep disorder (1.68; 1.63 to 1.73; *p* < 0.001), fatigue (1.28; 1.25 to 1.31; *p* < 0.001), pain (1.22; 1.20 to 1.24; *p* < 0.001), receipt of opioid analgesics(1.86; 1.83 to 1.90; *p* < 0.001), and fatal and nonfatal self-harm (1.15; 0.97 to 1.36, *p* = 0.11), although the CI was wide for the latter. There was no statistical evidence of an association for cognitive dysfunction (1.00; 0.97 to 1.04, *p* = 0.88). The full models are presented in Tables A and B in S2 Table. At 10 years of follow-up, the outcomes with highest cumulative incidence were pain (breast cancer survivors 85.6%; controls 79.1%), opioid analgesics (breast cancer survivors 45.5%; controls: 31.1%), depression (breast cancer survivors 28.5%; controls 24.0%), and fatigue (breast cancer survivors 23.9%; controls 19.9%) (Fig 2, S3 Table).

**Table 2:** Associations between breast cancer survivorship and adverse mental health outcomes, fatigue, and pain; women diagnosed with breast cancer in the UK between 1988 and 2018, compared to women who never had cancer.

	Breast cancer survivors			Women without cancer			Minimally adjusted associations*			Fully adjusted associations^†^		
Outcome	No. in analysis	No. of events	PY at risk	No. in analysis	No. of events	PY at risk	HR	95% CI	*p*-value	HR	95% CI	*p*-value
Anxiety	55,616	5,888	288,115	224,138	20,224	1,306,784	1.35	1.31–1.38	*p* < 0.001	1.33	1.29–1.36	*p* < 0.001
Depression	54,073	10,175	261,081	216,355	34,558	1,202,647	1.37	1.35–1.40	*p* < 0.001	1.35	1.32–1.38	*p* < 0.001
Cognitive dysfunction	56,052	4,368	315,453	224,444	19,845	1,385,179	1.03	1.00–1.06	*p* = 0.08	1.00	0.97–1.04	*p* = 0.88
Fatigue	55,911	8,359	280,982	223,506	28,886	1,266,975	1.31	1.28–1.34	*p* < 0.001	1.28	1.25–1.31	*p* < 0.001
Pain	38,771	24,522	100,312	162,037	94,171	505,451	1.28	1.26–1.30	*p* < 0.001	1.22	1.20–1.24	*p* < 0.001
Sexual dysfunction	57,444	683	325,393	229,577	2,153	1,435,837	1.34	1.24–1.44	*p* < 0.001	1.27	1.17–1.38	*p* < 0.001
Sleep disorder	56,210	6,002	290,786	225,583	16,798	1,338,065	1.71	1.66–1.76	*p* < 0.001	1.68	1.63–1.73	*p* < 0.001
Opioid analgesics	52,672	17,315	248,654	213,190	44,850	1,181,155	1.94	1.92–1.98	*p* < 0.001	1.86	1.83–1.90	*p* < 0.001
Fatal and nonfatal self-harm^§^	32,381	157	188,102	130,590	595	827,007	1.16	0.99–1.36	*p* = 0.07	1.15	0.97–1.36	*p* = 0.11

95% CI, 95% confidence interval; BMI, body mass index; CPRD, Clinical Practice Research Datalink; HR, hazard ratio; ONS, Office for National Statistics; PY, person-years.

* Women with a breast cancer diagnosis were matched with women who never had cancer on age (within a 3-year range), primary care practice (proxy of socioeconomic status), and eligibility for data linkage (to avoid loss of precision in subset analyses). No other variables were adjusted for in these analyses.

† Further adjusted for diabetes, BMI (restricted cubic spline), and smoking and drinking status.

§ Patients in England only, as the analysis was restricted to CPRD GOLD primary care data linked to the Hospital and Episode Statistics and ONS mortality data.

**Fig 3:**
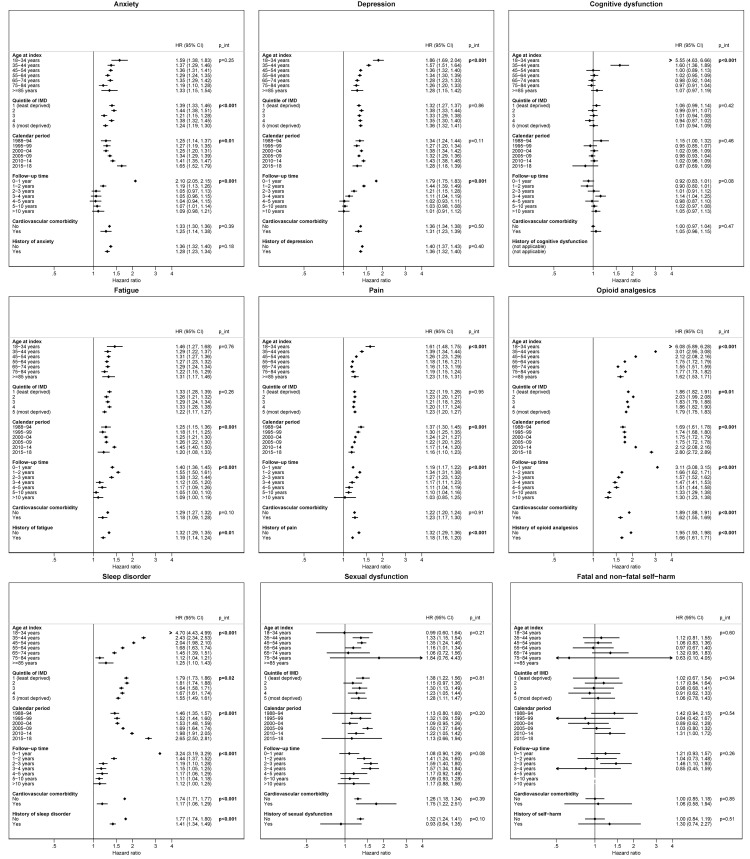
Forest plots of the association between breast cancer survivorship and adverse mental health outcomes, fatigue, and pain, stratified by potential effect modifiers. 95% CI, 95% confidence interval; HR, hazard ratio; IMD, Index of Multiple Deprivation.

Fig 3 shows results stratified by potential effect modifiers. We found strong statistical evidence supporting interactions between age and the relative risk of depression, cognitive dysfunction, pain, opioid analgesics prescribing, and sleep disorder, with HRs larger for younger women (for depression: HR = 1.86 [1.69 to 2.04] in women age 18 to 34, falling to 1.28 [1.15 to 1.42] in those 75 to 84, *p*-interaction <0.001). Women with breast cancer living in the less deprived areas had higher risks for anxiety, sleep disorders, and opioid analgesic use (for anxiety: HR = 1.39 [1.33 to 1.46] in the least deprived areas, falling to 1.24 [1.19 to 1.30] in the most deprived areas, *p*-interaction <0.001). HRs also varied by survivorship time, with the largest risks around diagnosis that tended to decline over time: In the first year of diagnosis, HRs for anxiety and depression were 2.10 (2.05 to 2.15) and 1.79 (1.75 to 1.83), and these outcomes reached similar levels to those of women with no cancer history by the third and fifth year after diagnosis respectively (*p*-interaction <0.001 for both outcomes). Finally, there was some variation by calendar period, with HRs for anxiety, fatigue, sleep disorders, and opioid prescription largest in the latest period, while the HR for pain was smaller later in calendar time.

### Sensitivity analyses

**Fig 4:**
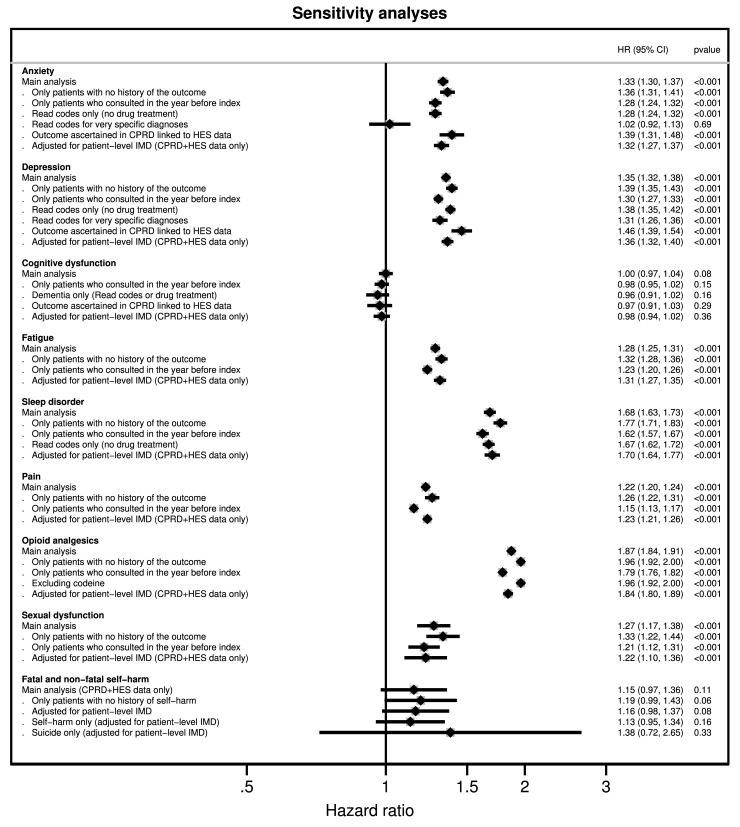
Results of sensitivity analyses. Note: Read codes for very specific diagnoses are provided in S1 Codelists. For anxiety, these included, for example, generalised anxiety disorder and panic, and for depression, major depressive disorder. 95% CI, 95% confidence interval; CPRD, Clinical Practice Research Datalink; HES, Hospital Episodes Statistics; HR, hazard ratio; IMD, Index of Multiple Deprivation.

Results of sensitivity analyses are shown in Fig 4. Restricting the analysis to patients with no prior history of the outcome, or to patients who had been in contact with the GP practice in the year prior the index date, yielded similar results to those of the main analysis. Sensitivity analyses adjusting for patient-level IMD did not change the results meaningfully. Analyses where hospital data were also used to identify outcomes resulted in similar risk estimates, as did the analyses excluding drugs from the definition of outcomes. A sensitivity analysis using only very specific codes for diagnoses of depressive disorders (e.g., major depressive disorder) yielded similar results; however, no association was observed when specific codes were used for anxiety disorders (e.g., generalised anxiety disorder) (HR = 1.02, 95% CI: 0.92 to 1.13, *p* = 0.69).

## Discussion

We found that breast cancer survivorship was associated with our primary outcomes of anxiety and depression in breast cancer survivors in the UK, compared to women with no prior cancer, as well as increased frequency of sleep disorder, sexual dysfunction, fatigue, receipt of opioid analgesics, and pain. No statistically significant differences between breast cancer survivors and controls were found for cognitive dysfunction and fatal and nonfatal self-harm, although there was a suggestion of a positive association for the latter. Younger age and less time elapsed since diagnosis were strongly associated with larger HRs for most outcomes. The relative risks estimates tended to diminish over time, but anxiety and depression were significantly increased for 2 and 4 years after breast cancer diagnosis, respectively, while associations for fatigue, pain, and sleep disorders were elevated for at least 5 to 10 years after diagnosis.

The association between breast cancer survivorship and anxiety and depression in the UK is consistent with previous studies, although incidences and effect sizes have varied between studies [5,7,25–28]. Of 60 studies investigating mental health outcomes in female breast cancer survivors compared to those without cancer included in a recent systematic review [5], only 1 was conducted in the UK [6]. Khan and colleagues [6] reported similar odds of consulting for depression and anxiety of breast cancer survivors 5 or more years post diagnosis, compared to the general population. This study builds on this work, analysing data from 1988 to 2018, to evaluate the risk of not only anxiety and depression, but 7 other mental health–related outcomes, and investigated associations from as early as the first year after diagnosis. This generated evidence for outcomes where none existed in the UK (cognitive dysfunction, fatigue, sexual dysfunction, pain, opioid prescriptions, sleep disorders, and fatal and nonfatal self-harm) and provided estimates of the risk of anxiety and depression in the early period of cancer survivorship.

The higher incidence of anxiety and depression in this study, compared to studies based on psychiatric assessment [7,28–30], is likely to be explained by less stringent diagnostic criteria in primary care. HR estimates for anxiety increased over calendar time; the drivers of this increase are unclear but might include more recording of symptoms in later years [31]. We did not observe increased associations of anxiety or depression 5 years after breast cancer diagnosis, although we cannot rule out that longer-lasting increased risks may apply for some subgroups of survivors. Our results for the secondary outcomes were consistent with the limited previous research available [5], except that we did not find significantly raised associations for suicide or cognitive dysfunction. The former is likely a power issue: An increased risk of suicide has been demonstrated in a large international study [32], and our point estimates were in the same direction, but CIs were very wide due to small numbers of events. For cognitive dysfunction, we found no suggestion of an association except in the youngest patients (18 to 34 years), in contrast with other studies that have estimated 13% to 70% of breast cancer patients to be affected by this outcome within 2 years of treatment [33]. However, this often involved mild memory impairments, which may cause distress but usually do not completely impede most daily activities [34], and it is possible that patients do not report this to their GPs, explaining the lack of effect in our data. In addition, memory problems are not uncommon later in life, and GPs may not record these concerns as much as for younger women.

A major strength of our study is its population-based nature; we used primary care data and registration with a primary care practice is nearly universal in the UK [35], so our findings are likely to be generalisable to the broad population of breast cancer survivors in the UK and similar settings. CPRD GOLD primary care data have been shown to have good validity for both cancer and mental health outcomes [36,37]. Confounding by age and socioeconomic status was controlled for through matching for narrow age groups and patients’ GP practice; the latter also had the advantage of accounting for practice-level characteristics that are difficult to measure (e.g., shared environment). The large study size and the breadth of data available in the CPRD GOLD primary care database permitted the study of several outcomes over 31 years with sufficient power to detect small effects and to generate evidence for little studied outcomes such as sleep disorders. We carried out multiple sensitivity analyses to check the robustness of our results. However, our study also has some limitations. There may have been detection bias if breast cancer survivors had more regular contact with health services, even if we expected this to have little impact as the sensitivity analysis including only patients who consulted in the year before index showed similar results. There is a potential for misclassification of outcomes due to incomplete information registered in the primary care record, for example, due to secondary care diagnoses not being fed back to the GP. There may have been residual confounding: Area-based deprivation was used as a proxy for socioeconomic status, but this may not accurately represent the individual socioeconomic situation; smoking and alcohol data relied on patients self-reporting accurately to their GP, and no detail on quantity of smoking/drinking was available; and we had no data on menopausal status, physical activity, or mammography screening, although age matching should to some extent have taken account of menopausal status. We did not have data on cancer stage and treatment to allow stratification by these factors.

The results of this study can be used to inform clinical practice. Patient prehabilitation and rehabilitation strategies often focus on the physical consequences of the disease and treatments [38,39]. In 2018, a survey of 2,862 women with breast cancer in England revealed that 84% were not provided with information about the potential negative effect of the cancer on their long-term mental health, and 75% felt more socially isolated at the end of treatment than at diagnosis [40]. Talking about common emotional challenges experienced by other patients may help women to understand better their own emotional journey, reduce stigma, and encourage patients to raise concerns about their mental health should they need. Increased awareness is also needed among healthcare professionals to improve detection of the mental health conditions and may help communication between patients and clinicians, particularly about sex-related issues, which appears to be poor [41].

Further research is needed to investigate the precise drivers of the associations we have observed between breast cancer survivorship and adverse mental health outcomes, which may include type of surgery, receipt and type of systemic treatment, tumour characteristics, presence of lymphedema, and other physical sequelae, among others. The effect of age on the likelihood of having cognitive dysfunction recorded in the clinical record also deserves further attention to ascertain whether this reflects differential recording by GPs. Interventions to identify, prevent, and treat mental health conditions in breast cancer survivors need to be designed and tested in robust trials. There is an important gap in evidence regarding effective treatment strategies for fatigue, sexual dysfunction, and cognitive dysfunction, all of which may negatively affect quality of life [42].

In conclusion, in this population-based study, we found that breast cancer survivorship was associated with increased anxiety, depression, sexual dysfunction, and sleep disorder, as well as fatigue, pain, and being prescribed opioid analgesics, compared to women with no history of cancer. Incidence of these disorders was particularly elevated in women within the first few years of breast cancer survivorship and more pronounced in younger women. Increased fatigue and pain were found for at least 5 to 10 years post-diagnosis. It is imperative to raise awareness among patients, healthcare professionals, and policy makers about the mental health needs of the growing population of breast cancer survivors.

## Supporting information

S1 Checklist STROBE Statement—checklist of items that should be included in reports of cohort studies. (DOCX)

S1 Methods Definition of outcomes and covariates. (DOCX)

S1 Codelists Codelist used to define outcomes. (DOCX)

S1 Protocol Study protocol. (DOCX)

S1 Table Table A: characteristics of the patients excluded and number and mean follow-up time of patients available for analyses: anxiety, depression, and cognitive dysfunction. Table B: characteristics of the patients excluded and number and mean follow-up time of patients available for analyses: fatigue, sexual dysfunction, and sleep disorder. Table C: characteristics of the patients excluded and number and mean follow-up time of patients available for analyses: pain, opioids analgesics, and fatal and nonfatal self-harm.
(DOCX)

S2 Table Cumulative incidence of adverse mental health outcomes, fatigue, and pain in breast cancer survivors and in women who did not have cancer. (DOCX)

S3 Table Table A: adjusted associations between breast cancer survivors and anxiety, depression, cognitive dysfunction, and fatigue. Table B: adjusted associations between breast cancer survivors and sexual dysfunction, sleep disorders, pain, opioid prescription, and fatal and nonfatal self-harm.
(DOCX)

## Abbreviations

BMI: body mass index
CI: confidence interval
CPRD: Clinical Practice Research Datalink
GP: general practitioner
HES-APC: Hospital Episodes Statistics—Admitted Patient Care
HR: hazard ratio
ICD-10: International Statistical Classification of Diseases and Related Health Problems 10th Revision
IMD: Index of Multiple Deprivation
IQR: interquartile range
ISAC: Independent Scientific Advisory Committee
ONS: Office for National Statistics
STROBE: Strengthening the Reporting of Observational Studies in Epidemiology
