# Supplementary material for: Associations between breast cancer survivorship and adverse mental health outcomes: A matched population-based cohort study in the United Kingdom
Source: PLoS Med. 2021 Jan 7;18(1):e1003504. doi: 10.1371/journal.pmed.1003504 (PMC7822529; doi:10.1371/journal.pmed.1003504)
Supplement: S1 Methods — (DOCX) [file pmed.1003504.s002.docx]

# **S1 Methods Definition of outcomes and covariates**

## Search and selection of Read codes and pharmacological drugs

We searched the dictionary of codes using keywords defined by a general practitioner experienced in using the codes in clinical practice (GF). We then identified the relevant parent code for the outcome, and included all Read codes within that group. Finally, we added all codes identified in a comprehensive systematic review of the lists of Read codes used to identify mental health and quality of life outcomes in primary care databases of electronic health records in the UK (Carreira et al, BMJ Open, 2019). Two researchers (HC and GF) independently assigned each Read code to a certainty group, compared and agreed the final list of codes (available online).

Drugs for anxiety, depression, sleep disorders and pain were identified in product dictionary by searching formulations listed in the British National Formulary (BNF) as indicated to treat these conditions. The final list of products was checked for suitability by a GP (GF) and irrelevant products were excluded (e.g. doxepin topical).

## Primary outcomes

### Anxiety

Anxiety was defined with Read codes, if the Read code was considered sufficiently specific. When the Read code referred to typical symptoms of anxiety, which are not necessarily pathological, we considered the patient to have anxiety only if they had been prescribed with a drug with anxiety within 90 days of the Read code.

Read codes for the following conditions were included/excluded from our definition:

| **Included** | **Excluded** |
| --- | --- |
| Generalised anxiety disorder | Specific phobias (e.g. heights) |
| Panic disorder | Somatic symptoms disorder |
| Agoraphobia |  |
| Social anxiety disorder |  |
| Mixed anxiety and depression |  |
| Obsessive compulsive disorders * |  |
| Trauma- and stress-related disorders* with anxiety, including PTSD, acute stress disorder, and adjustment disorder with anxiety |  |

N.B. ‘Included’ and ‘Excluded’ refer to *symptoms* and *diagnoses* of the conditions listed.

* In DSM-5, published in 2013, obsessive-compulsive and stress-related disorders are classified separately from anxiety disorders. This was a major change from previous editions of the DSM, in which these two categories were considered as anxiety disorders. The data for this study refer to patients under observation during 1988 and 2018 (or part of this period); it is unclear how, or if, the changes in nosology affected the use of Read codes by GPs at the point of patient care. In addition, the accuracy of the Read codes to identify each of the sub conditions is likely to be sub-optimal at any given point in time. For these reasons, we decided to include OCDs and stress-related disorders in our definition of anxiety. See below, in the depression section, a note about adjustment disorders with anxiety.

Drugs indicated to treat anxiety according to the British National Formulary:

| **Substance name** |
| --- |
| Alprazolam |
| Amitriptyline hydrochloride/ Chlordiazepoxide |
| Buspirone hydrochloride |
| Chlordiazepoxide hydrochloride |
| Diazepam |
| Duloxetine hydrochloride |
| Escitalopram oxalate |
| Lorazepam |
| Meprobamate |
| Moclobemide |
| Oxazepam |
| Oxprenolol hydrochloride |
| Paroxetine hydrochloride |
| Pericyazine |
| Perphenazine |
| Pregabalin |
| Trazodone hydrochloride |
| Venlafaxine hydrochloride |

International Classification of Diseases, 10^th^ revision, codes for anxiety:

| **ICD-10 codes** | **Description** |
| --- | --- |
| F40 | Phobic anxiety disorders |
| F41 | Other anxiety disorders |
| F42 | Obsessive-compulsive disorder |
| F43 | Reaction to severe stress, and adjustment disorders |
| F44 | Dissociative [conversion] disorders |
| F48 | Other neurotic disorders |

Definition used in sensitivity analysis including specific diagnoses:

| **Included (Read codes for diagnoses only)** | **Excluded** |
| --- | --- |
| Panic disorder | All symptom codes (e.g. ‘anxious’) |
| Generalized anxiety disorder |  |
| Mixed anxiety and depression |  |
| Obsessive-compulsive disorder |  |
| Acute stress disorder |  |
| Post-traumatic stress disorder |  |
| Anxiety disorder, NOS |  |

### Depression

We used Read codes alone to classify patients with depression, if the Read code was considered sufficiently specific. When the Read code referred to typical symptoms of depression that could not be sufficient to classify as a depressive episode, we checked whether there as a prescription of a drug commonly used to depression within 90 days, and considered patients to be depressed if yes.

Read codes for the following conditions were included/excluded from our definition:

| **Included** | **Excluded** |
| --- | --- |
| Major depressive disorder | Bipolar and related disorders (incl. bipolar I, II and cyclothymic disorder) |
| Dysthymia | Premenstrual dysphoric disorder |
| Recurrent depressive disorder | Suicide ^†^ |
| Mixed anxiety and depression | Self-harm ^†^ |
| Disruptive mood dysregulation disorder | Maternal depression |
| Depression in dementia (or other condition) |  |
| Trauma- and stress-related disorders* with depressed mood, including adjustment disorders with depressed mood* |  |

N.B. ‘Included’ and ‘Excluded’ refer to symptoms and diagnoses of the conditions listed.

* See note on anxiety table. Adjustment disorders are considered to be a short-term reaction to a stressor (i.e. diagnosed usually within 3 months of the onset of the stressor). The core symptoms of adjustment disorders overlap with those of the anxiety and depressive disorders, which would be diagnosed if the symptoms persist for longer than a 6-month period after the terminus of the stressor. The potential for misclassification between adjustment and depressive disorders is high, as they share the same symptomatology and treatment. The data for this study will include patients recently diagnosed with breast cancer (included in the cohort in the day of the cancer recording in the CPRD GOLD primary care database). To avoid misclassification of the outcome, we included adjustment disorders in our definitions of anxiety and depression.

† Self-harm and suicide most often occur in patients with a depressive disorder. We will examine these two outcomes separately.

Drugs indicated to treat depression according to the British National Formulary:

| **Substance name** |
| --- |
| Agomelatine |
| Amitriptyline Hydrochloride |
| Amitriptyline Hydrochloride/ Perphenazine |
| Citalopram hydrobromide |
| Citalopram hydrochloride |
| Clomipramine hydrochloride |
| Dosulepin hydrochloride |
| Dosulepin Hydrochloride |
| Duloxetine hydrochloride |
| Escitalopram oxalate |
| Fluoxetine hydrochloride |
| Fluvoxamine maleate |
| Imipramine hydrochloride |
| Isocarboxazid |
| Lofepramine hydrochloride |
| Mianserin hydrochloride |
| Mirtazapine |
| Moclobemide |
| Nortriptyline hydrochloride |
| Nortriptyline Hydrochloride |
| Paroxetine hydrochloride |
| Phenelzine sulfate |
| Reboxetine mesilate |
| Sertraline |
| Sertraline hydrochloride |
| Tranylcypromine sulfate |
| Trazodone Hydrochloride |
| Trimipramine maleate |
| Venlafaxine hydrochloride |
| Venlafaxine Hydrochloride |
| Vortioxetine hydrobromide |

International Classification of Diseases, 10^th^ revision codes for depression:

| **ICD-10 codes** | **Description** |
| --- | --- |
| F32 | Depressive episode |
| F33 | Recurrent depressive disorder |
| F34 | Persistent mood [affective] disorders |
| F41.2 | Mixed anxiety and depressive disorder |
| F92.0 | Depressive conduct disorder |

Definition used in sensitivity analysis including specific diagnoses:

| **Included (Read codes for diagnoses only)** | **Excluded** |
| --- | --- |
| Depressive episode | All symptom codes (e.g. ‘depressed’) |
| Major depression |  |
| Seasonal affective disorder |  |
| Dysthymia |  |
| Mixed anxiety and depression |  |

## Secondary outcomes

### Cognitive dysfunction

Cognitive dysfunction was defined by Read codes for impairments in domain of cognitive function (e.g. ‘amnesia symptom’, ‘orientation confused’), or Read codes related to cognitive assessments (e.g. ‘mini-mental state examination’, ‘unable to remember own date of birth’), Read codes for dementia and drugs commonly used to treat dementia.

We used a broad definition of cognitive dysfunction because we were interested in mild cognitive dysfunction, which is often reported by women with history of breast cancer after diagnosis and treatment. However, changes to cognitive dysfunction, especially those in older adults, may not lead to primary care until it becomes troublesome for the patient or their family. At this point, the patient may be diagnosed with more severe levels of cognitive dysfunction, and we would not be able to identify an outcome of ‘mild cognitive dysfunction’ in the CPRD primary care database. As loss of cognitive function is a gradual process, we defined cognitive dysfunction using codes that ranged from mild cognitive dysfunction to dementia. We also included codes for scales/tests because we assumed that patients who have had a cognitive assessment registered by their GP might have relevant cognitive complains. Drugs were considered sufficient to ascertain the outcome because these are very specific to dementia.

Read codes for the following conditions were included/excluded from our definition:

| **Included** | **Excluded** |
| --- | --- |
| Mild cognitive impairment | Delirium |
| Alzheimer’s disease | Dementia in Creutzfeldt-Jakob disease † |
| Vascular dementia | Dementia in Huntington's disease † |
| Frontotemporal dementia | Dementia in Parkinson's disease † |
| Dementia in Pick's disease | Dementia in human immunodeficiency virus [HIV] disease † |
| Unspecified dementia | Normal pressure hydrocephalus † |

† We excluded dementia with well-described cause, which is unlikely to be associated with a cancer history.

Drugs indicated to treat dementia according to the British National Formulary:

| **Substance name** |
| --- |
| Donepezil hydrochloride |
| Galantamine hydrobromide |
| Memantine hydrochloride |

International Classification of Diseases, 10^th^ revision codes for dementia:

| **ICD-10 codes** | **Description** |
| --- | --- |
| F00 | Dementia in Alzheimer disease |
| F01 | Vascular dementia |
| F02.0 | Dementia in Pick disease |
| F03 | Unspecified dementia |
| F06.7 | Mild cognitive disorder |

### Fatigue

Patients were classified as having had fatigue using Read codes (list of Read codes available online). We included/excluded the following conditions in our definition of fatigue:

| **Included** | **Excluded** |
| --- | --- |
| Chronic fatigue syndrome/ myalgic encephalitis | Combat fatigue |
| Neurasthenia | Fatigue in pregnancy |
| Post viral fatigue syndrome * | Fibromyalgia † |

N.B. ‘Included’ and ‘Excluded’ refer to *symptoms* and diagnoses of the conditions listed. We included symptoms such as ‘tired all the time’ because GPs may be less likely to diagnose chronic fatigue syndrome if symptoms can be attributed to the breast cancer treatments.

* Post-viral fatigue syndrome our definition of fatigue because there is a high potential for misclassification of these outcomes at primary care level, as viral infections are common.

† Studies have shown a considerable overlap between fatigue and fibromyalgia, with at least 75% of the patients diagnosed with fibromyalgia report fatigue (Clin Rev Allergy Immunol. 2015 Oct;49(2):100-51). We expect these patients to be captured by the terms for fatigue defined in the conditions of interest for this study.

### Pain

Pain was defined using Read codes for pain of specific regions of the body (e.g. chest pain), and of known conditions that may be caused by treatments (e.g. arthralgia in patients who are treated with hormone therapy, or post-surgical pain), unspecified pain (e.g. pain symptom). Pain syndromes (e.g. fibromyalgia) were included. Read codes for pain scales were also considered as evidence of pain, as we assumed that this would not be offered to the patient if s/he did not complain of pain. Codes for rheumatoid arthritis and arthroses were excluded, as well as codes for fractures. We also excluded codes for pain of known aetiology that is unlikely to be related to breast cancer treatments (e.g. post-herpetic pain, diabetic neuropathic pain, menstrual pain, fractures, accidents, etc.).

### Opioid analgesics

All opioid analgesics listed in the British National Formulary were considered eligible. We did not include codes for treatment of opioid dependency.

### Sleep disorder

Sleep disorder was defined with Read codes, if the Read code was considered sufficiently specific. When the Read code referred to symptoms or possible treatment of sleep disorder (e.g. ‘poor sleep pattern’, ‘sleep hygiene behaviour education’), we considered the patient to have a sleep disorder only if they had been prescribed with an anxiolytic/hypnotic (table below) within 90 days of the code.

Read codes for the following conditions were included/excluded from our definition:

| **Included** | **Excluded** |
| --- | --- |
| Insomnia | Narcolepsy |
| Hypersomnia | Breathing-related disorders (including sleep apnoea) |
| Circadian rhythm sleep-wake disorders | Cataplexy |
| Parasomnias |  |

N.B. ‘Included’ and ‘Excluded’ refer to *symptoms* and diagnoses of the conditions listed.

Drugs indicated to treat sleep disorders according to the British National Formulary:

| **Substance name** |
| --- |
| Temazepam |
| Nitrazepam |
| Diazepam |
| Zopiclone |
| Clomethiazole |
| Promethazine hydrochloride |
| Promethazine teoclate |
| Zolpidem tartrate |
| Codeine phosphate/promethazine hydrochloride |
| Clomethiazole edisilate |
| Flunitrazepam |
| Flurazepam hydrochloride |
| Lormetazepam |
| Oxazepam |
| Promethazine hydrochloride/paracetamol |
| Loprazolam mesilate |
| Melatonin |
| Promethazine hydrochloride/pholcodine |
| Pethidine hydrochloride/promethazine hydrochloride |
| Paracetamol/promethazine hydrochloride/ dextromethorphan hydrobromide |
| Dextromethorphan hydrobromide/promethazine hydrochloride/ paracetamol |

### Female sexual dysfunction

We included only Read codes in our definition of sexual dysfunction. We included Read codes for scales of sexual function (e.g. Derogatis Sexual Dysfunction Inventory) because the patient is likely to have had subjective complains of sexual function in order for the GP to apply the test.

We considered the follow clinical disorders as relevant outcomes:

| **Included** | **Excluded** |
| --- | --- |
| Female orgasmic disorder | Paraphilia |
| Female arousal disorder | Excess sex drive |
| Dyspareunia | Sexual orientation related codes |

We acknowledge that codes for improved sexual function are likely to indicate that a disorder has been present; these were nevertheless excluded as the date of the disorder cannot be ascertained.

In addition, prescriptions for sexual dysfunction are not likely to capture accurately the disorder, as it may include lubricants that are often sold over the counter, or creams containing oestrogen that may be under prescribed to breast cancer survivors due to concerns related with oestrogen positive receptor tumours.

### Fatal and non-fatal self-harm

Fatal and non-fatal self-harm included codes for intentional self-harm and suicidal ideation, using an updated version of a previously validated list of Read codes (Br J Clin Pharmacol 2013; 76(1): 145-57), and International Classification of Diseases, tenth revision, (ICD-10) codes for completed suicide. Completed suicide was defined using the ICD-10 codes X60-X84 and Y10-34, excluding Y33.9 where the verdict is pending.

International Classification of Diseases, 10^th^ revision codes for self-harm:

| **ICD-10 codes** | **Description** |
| --- | --- |
| X60-X84 | Intentional self-harm |
| Y10-Y34 | Event of undetermined intent |
| Y87.0 | **Sequelae of intentional self-harm** |
| Y87.2 | **Sequelae of events of undetermined intent** |

## Definition of covariates

### Alcohol and smoking status

Information on alcohol drinking and smoking status was obtained from primary care records, and patients were assigned into the following categories for each variable: non-users, current users, and former users. For these two variables, we prioritised information registered in the year prior to the index date, or up to 30 days after the index date; where this was unavailable we used information recorded at any point prior to the index date, and if still missing, we used information recorded at any point in the clinical record.

### Body mass index

Body mass index (BMI) (kg/m^2^) was computed as weight divided by the square of height, using a previously defined algorithm to ascertain this information from the clinical records (BMJ Open 2013; 3(9): e003389).

### Diabetes mellitus

Diabetes mellitus was defined as having had a Read code for type I or type II diabetes mellitus recorded up to 30 days after the index date (assuming that any event recorded within the first month would be prevalent).

### Cardiovascular comorbidity

Cardiovascular comorbidity was defined as having a record of stroke or ischaemic heart disease (angina and coronary heart disease) before the study index date, or up to 30 days after the index date.

### Deprivation

Deprivation was defined by quintiles of the practice-postcode linked Index of Multiple Deprivation (IMD), which is an ecological measure of deprivation for small areas in England (Lower Super Output Areas). For a subset of patients, patient-postcode linked quintile of IMD was available.
