# Supplementary material for: Associations between breast cancer survivorship and adverse mental health outcomes: A matched population-based cohort study in the United Kingdom
Source: PLoS Med. 2021 Jan 7;18(1):e1003504. doi: 10.1371/journal.pmed.1003504 (PMC7822529; doi:10.1371/journal.pmed.1003504)
Supplement: S1 Codelists — (DOCX) [file pmed.1003504.s003.docx]

# S1 Table A Codelist for breast cancer – study population.

| medcode | Read code | Description |
| --- | --- | --- |
| 348 | B34..11 | CA FEMALE BREAST |
| 64686 | B340100 | MALIGNANT NEOPLASM OF AREOLA OF FEMALE BREAST |
| 67884 | B350100 | MALIGNANT NEOPLASM OF AREOLA OF MALE BREAST |
| 20685 | B346.00 | MALIGNANT NEOPLASM OF AXILLARY TAIL OF FEMALE BREAST |
| 31546 | B341.00 | MALIGNANT NEOPLASM OF CENTRAL PART OF FEMALE BREAST |
| 95057 | B34y000 | MALIGNANT NEOPLASM OF ECTOPIC SITE OF FEMALE BREAST |
| 3968 | B34..00 | MALIGNANT NEOPLASM OF FEMALE BREAST |
| 9470 | B34z.00 | MALIGNANT NEOPLASM OF FEMALE BREAST NOS |
| 45222 | B343.00 | MALIGNANT NEOPLASM OF LOWER-INNER QUADRANT OF FEMALE BREAST |
| 42070 | B345.00 | MALIGNANT NEOPLASM OF LOWER-OUTER QUADRANT OF FEMALE BREAST |
| 26853 | B340.00 | MALIGNANT NEOPLASM OF NIPPLE AND AREOLA OF FEMALE BREAST |
| 23380 | B340000 | MALIGNANT NEOPLASM OF NIPPLE OF FEMALE BREAST |
| 59831 | B340z00 | MALIGNANT NEOPLASM OF NIPPLE OR AREOLA OF FEMALE BREAST NOS |
| 56715 | B34y.00 | MALIGNANT NEOPLASM OF OTHER SITE OF FEMALE BREAST |
| 38475 | B34yz00 | MALIGNANT NEOPLASM OF OTHER SITE OF FEMALE BREAST NOS |
| 29826 | B342.00 | MALIGNANT NEOPLASM OF UPPER-INNER QUADRANT OF FEMALE BREAST |
| 23399 | B344.00 | MALIGNANT NEOPLASM OF UPPER-OUTER QUADRANT OF FEMALE BREAST |
| 49148 | B347.00 | MALIGNANT NEOPLASM, OVERLAPPING LESION OF BREAST |
| 58131 | BB93.00 | [M]COMEDOCARCINOMA NOS |
| 59251 | BBM9.00 | [M]CYSTOSARCOMA PHYLLODES, MALIGNANT |
| 39760 | BB91100 | [M]INFILTRATING DUCT AND LOBULAR CARCINOMA |
| 8351 | BB91.00 | [M]INFILTRATING DUCT CARCINOMA |
| 7319 | BB9G.00 | [M]INFILTRATING DUCTULAR CARCINOMA |
| 32472 | BB9H.00 | [M]INFLAMMATORY CARCINOMA |
| 30189 | BB91000 | [M]INTRADUCTAL PAPILLARY ADENOCARCINOMA WITH INVASION |
| 40359 | BB94.00 | [M]JUVENILE BREAST CARCINOMA |
| 12427 | BB9F.00 | [M]LOBULAR CARCINOMA NOS |
| 98883 | BB9D.00 | [M]MEDULLARY CARCINOMA WITH LYMPHOID STROMA |
| 42542 | BB9K.00 | [M]PAGET'S DISEASE AND INFILTRATING BREAST DUCT CARCINOMA |
| 12480 | BB9K000 | [M]PAGET'S DISEASE AND INTRADUCTAL CARCINOMA OF BREAST |
| 60803 | BB9J.11 | [M]PAGET'S DISEASE, BREAST |
| 12300 | BB9J.00 | [M]PAGET'S DISEASE, MAMMARY |
| 67701 | BB94.11 | [M]SECRETORY BREAST CARCINOMA |
| 12499 | Byu6.00 | [X]MALIGNANT NEOPLASM OF BREAST |

# S1 Table B Codelist for anxiety – main analysis.

| medcode | Read code | Read term | Anxiety |
| --- | --- | --- | --- |
| 131 | 1B13.00 | Anxiousness | Probable |
| 276 | E28..00 | Acute reaction to stress | Probable |
| 462 | E200111 | Panic attack | Probable |
| 636 | E200.00 | Anxiety states | Probable |
| 655 | E200300 | Anxiety with depression | Probable |
| 962 | Eu41111 | [X]Anxiety neurosis | Probable |
| 1758 | E200400 | Chronic anxiety | Probable |
| 2030 | E203100 | Obsessional neurosis | Probable |
| 2571 | Eu40000 | [X]Agoraphobia | Probable |
| 3076 | E202100 | Agoraphobia with panic attacks | Probable |
| 3208 | E203.00 | Obsessive-compulsive disorders | Probable |
| 4069 | E200100 | Panic disorder | Probable |
| 4081 | Eu41012 | [X]Panic state | Probable |
| 4171 | Eu43100 | [X]Post - traumatic stress disorder | Probable |
| 4534 | E200z00 | Anxiety state NOS | Probable |
| 4634 | E200500 | Recurrent anxiety | Probable |
| 4659 | E200200 | Generalised anxiety disorder | Probable |
| 5304 | Eu42.00 | [X]Obsessive - compulsive disorder | Probable |
| 5385 | Eu41.00 | [X]Other anxiety disorders | Probable |
| 5678 | E203000 | Compulsive neurosis | Probable |
| 6221 | E292000 | Separation anxiety disorder | Probable |
| 6408 | Eu41011 | [X]Panic attack | Probable |
| 6939 | E200000 | Anxiety state unspecified | Probable |
| 7749 | Eu41211 | [X]Mild anxiety depression | Probable |
| 7999 | Z4L1.00 | Anxiety counselling | Probable |
| 8205 | Eu41000 | [X]Panic disorder [episodic paroxysmal anxiety] | Probable |
| 9125 | 8G94.00 | Anxiety management training | Probable |
| 9386 | Eu40.00 | [X]Phobic anxiety disorders | Probable |
| 9944 | E202.12 | Phobic anxiety | Probable |
| 10344 | Eu41100 | [X]Generalized anxiety disorder | Probable |
| 10535 | Eu43012 | [X]Acute reaction to stress | Probable |
| 11098 | Eu43.00 | [X]Reaction to severe stress, and adjustment disorders | Probable |
| 11607 | Eu43000 | [X]Acute stress reaction | Probable |
| 11890 | 1B1V.00 | C/O - panic attack | Probable |
| 11913 | Eu41200 | [X]Mixed anxiety and depressive disorder | Probable |
| 11940 | E280.00 | Acute panic state due to acute stress reaction | Probable |
| 12838 | E202200 | Agoraphobia without mention of panic attacks | Probable |
| 13124 | 2258 | O/E - anxious | Probable |
| 14890 | Eu40012 | [X]Panic disorder with agoraphobia | Probable |
| 15220 | Eu34114 | [X]Persistant anxiety depression | Probable |
| 15566 | E203z00 | Obsessive-compulsive disorder NOS | Probable |
| 16729 | Eu40011 | [X]Agoraphobia without history of panic disorder | Probable |
| 18399 | Eu42200 | [X]Mixed obsessional thoughts and acts | Probable |
| 19000 | 225J.00 | O/E - panic attack | Probable |
| 20773 | Eu05400 | [X]Organic anxiety disorder | Probable |
| 21753 | Eu43y00 | [X]Other reactions to severe stress | Probable |
| 21836 | Eu42.12 | [X]Obsessive-compulsive neurosis | Probable |
| 22019 | Eu42100 | [X]Predominantly compulsive acts [obsessional rituals] | Probable |
| 22159 | Z4I7.00 | Acknowledging anxiety | Probable |
| 22721 | Eu42z00 | [X]Obsessive-compulsive disorder, unspecified | Probable |
| 23838 | Eu41z00 | [X]Anxiety disorder, unspecified | Probable |
| 23869 | E284.00 | Stress reaction causing mixed disturbance of emotion/conduct | Probable |
| 24066 | Eu41y00 | [X]Other specified anxiety disorders | Probable |
| 25638 | Eu41z11 | [X]Anxiety NOS | Probable |
| 26138 | E28z.00 | Acute stress reaction NOS | Probable |
| 26295 | Z4I7211 | Reducing anxiety | Probable |
| 27685 | Eu40y00 | [X]Other phobic anxiety disorders | Probable |
| 28167 | Eu41y11 | [X]Anxiety hysteria | Probable |
| 28381 | Z4I7200 | Alleviating anxiety | Probable |
| 28925 | 8HHp.00 | Referral for guided self-help for anxiety | Probable |
| 29707 | E283z00 | Other acute stress reaction NOS | Probable |
| 31515 | Eu43z00 | [X]Reaction to severe stress, unspecified | Probable |
| 32387 | E29y100 | Other post-traumatic stress disorder | Probable |
| 34064 | Eu40z00 | [X]Phobic anxiety disorder, unspecified | Probable |
| 35825 | Eu41112 | [X]Anxiety reaction | Probable |
| 38640 | E283.00 | Other acute stress reactions | Probable |
| 38809 | Eu42y00 | [X]Other obsessive-compulsive disorders | Probable |
| 42737 | E281.00 | Acute fugue state due to acute stress reaction | Probable |
| 44321 | Eu41300 | [X]Other mixed anxiety disorders | Probable |
| 50191 | Eu41113 | [X]Anxiety state | Probable |
| 56924 | E292400 | Adjustment reaction with anxious mood | Probable |
| 62935 | Z4I7100 | Recognising anxiety | Probable |
| 93401 | 1B13.12 | Anxious | Probable |
| 107134 | Z522600 | Flooding - obsessional compulsive disorder | Probable |
| 108107 | 8CAZ000 | Patient given advice about management of anxiety | Probable |
| 111028 | U607y00 | [X]Oth sedat/hypnot/antianx drug caus advers eff therap use | Probable |
| 514 | 1B12.12 | Tension - nervous | Possible |
| 791 | E20z.11 | Nervous breakdown | Possible |
| 1582 | E205.11 | Nervous exhaustion | Possible |
| 2291 | 13JK.14 | Work worries | Possible |
| 2509 | R2y2.00 | [D]Nervousness | Possible |
| 2524 | 1BK..00 | Worried | Possible |
| 2585 | 1B14.11 | Tenseness - symptom | Possible |
| 3328 | 1B1..00 | General nervous symptoms | Possible |
| 3586 | 1B12.11 | 'Nerves' | Possible |
| 4127 | 13JK.00 | Business worries | Possible |
| 5347 | 1B1H.11 | Fear | Possible |
| 5811 | 1B16.11 | Agitated - symptom | Possible |
| 5902 | 1B13.11 | Anxiousness - symptom | Possible |
| 6142 | 1B15.11 | Irritable - symptom | Possible |
| 7383 | R00zD00 | [D]Restlessness and agitation | Possible |
| 8250 | R00zW00 | [D]State of emotional shock and stress, unspecified | Possible |
| 8725 | 2259 | O/E - nervous | Possible |
| 9970 | 388Z.00 | Depression anxiety stress scales depression score | Possible |
| 10723 | R2y2.12 | [D]Nervous tension | Possible |
| 17853 | R2y2.11 | [D]Nerves | Possible |
| 18494 | 1Ba0.00 | Obsessional thoughts | Possible |
| 19163 | 388b.00 | Depression anxiety stress scales anxiety score | Possible |
| 19245 | ZRLr.00 | Hospital anxiety and depression scale | Possible |
| 19630 | 388J.00 | Hospital anxiety and depression scale | Possible |
| 19631 | 388N.00 | HAD scale: anxiety score | Possible |
| 20089 | 1B1Z.00 | General nervous symptom NOS | Possible |
| 20163 | 1B1H.12 | Apprehension | Possible |
| 20375 | ZV65511 | [V]'Worried well' | Possible |
| 20634 | Eu42000 | [X]Predominantly obsessional thoughts or ruminations | Possible |
| 21431 | Eu46z11 | [X]Neurosis NOS | Possible |
| 22654 | 1P3..00 | Compulsive behaviour | Possible |
| 23808 | Eu4..00 | [X]Neurotic, stress - related and somoform disorders | Possible |
| 24251 | Eu42.11 | [X]Anankastic neurosis | Possible |
| 24820 | ZRLr.12 | HADS - Hospital anxiety and depression scale | Possible |
| 25213 | 28Z..00 | Nervous syst/mental state NOS | Possible |
| 25344 | E241.00 | Hypnotic or anxiolytic dependence | Possible |
| 26079 | ZR29.00 | Beck anxiety inventory | Possible |
| 26208 | E241100 | Hypnotic or anxiolytic dependence, continuous | Possible |
| 27665 | Ry15.00 | [D]Undue concern and preoccupation with stressful events | Possible |
| 28408 | 2J4..00 | Worried well | Possible |
| 29569 | 1B14.00 | Tenseness | Possible |
| 29608 | 1B12.00 | 'Nerves' - nervousness | Possible |
| 29797 | E241z00 | Hypnotic or anxiolytic dependence NOS | Possible |
| 35398 | ZRLr.11 | HAD - Hospital anxiety and depression scale | Possible |
| 35733 | E241.11 | Anxiolytic dependence | Possible |
| 40578 | 13JM.14 | Work worries | Possible |
| 40739 | ZR2C.00 | Beck anxiety standardised rating scale | Possible |
| 41066 | 388a.00 | Depression anxiety stress scales stress score | Possible |
| 43296 | E254.12 | Hypnotic or anxiolytic abuse | Possible |
| 47365 | E203.11 | Anancastic neurosis | Possible |
| 50778 | E241200 | Hypnotic or anxiolytic dependence, episodic | Possible |
| 52243 | ZR3U.00 | Clinical anxiety scale | Possible |
| 52655 | ZR7..00 | Depression anxiety scale | Possible |
| 53025 | E241000 | Hypnotic or anxiolytic dependence, unspecified | Possible |
| 56020 | Ryu5800 | [X]State of emotional shock and stress, unspecified | Possible |
| 56034 | ZRVM.00 | Leeds scale for the self-assessment of anxiety & depression | Possible |
| 56445 | ZRre.11 | SASZ - Zung's self-rating anxiety scal | Possible |
| 60180 | E254.00 | Nondependent hypnotic or anxiolytic abuse | Possible |
| 64519 | ZRkb.11 | STAI - Speilberger state-trait anxiety inventory | Possible |
| 65942 | E254200 | Nondependent hypnotic or anxiolytic abuse, episodic | Possible |
| 66243 | E254000 | Nondependent hypnotic or anxiolytic abuse, unspecified | Possible |
| 72649 | ZRre.00 | Zung's self-rating anxiety scale | Possible |
| 73859 | U607.00 | [X]Sedats/hypnots/antianx drug caus advers eff in therap use | Possible |
| 94196 | 388w.00 | Generalised anxiety disorder 7 item score | Possible |
| 94394 | E254z00 | Nondependent hypnotic or anxiolytic abuse NOS | Possible |
| 94671 | 388w.11 | GAD-7 score | Possible |
| 95547 | ZRrd.00 | Zung's anxiety status inventory | Possible |
| 95954 | E254100 | Nondependent hypnotic or anxiolytic abuse, continuous | Possible |
| 101422 | 16ZB100 | Feeling low or worried | Possible |
| 102106 | 38Du100 | IAPT phobia scale - Cert situ fear panic attak distres symp | Possible |
| 105292 | 38GQ.00 | Short health anxiety inventory | Possible |
| 107410 | 173f.00 | Anxiety about breathlessness | Possible |
| 108194 | 6897 | Anxiety screening | Possible |
| 108245 | 6897000 | Anxiety screening using questions | Possible |
| 108416 | 38QN.00 | Generalised anxiety disorder 2 scale | Possible |
| 108928 | 38GJ000 | EuroQol five dimension five level anxiety depression score | Possible |
| 109490 | 8IH3000 | Anxiety screening declined | Possible |
| 109688 | 8IH8.00 | GAD-7 (Generalized Anxiety Disorder 7) scale declined | Possible |
| 110350 | 8IE5100 | Hospital Anxiety and Depression Scale declined | Possible |
| 110355 | ZRkb.00 | Speilberger state-trait anxiety inventory | Possible |
| 3407 | 1466 | H/O: anxiety state | History of |
| 48702 | E241300 | Hypnotic or anxiolytic dependence in remission | History of |
| 68396 | E254300 | Nondependent hypnotic or anxiolytic abuse in remission | History of |

# S1 Table C Codelist for anxiolytics – main analysis.

| prodcode | Product Name |
| --- | --- |
| 46 | Diazepam 2mg tablets |
| 47 | Diazepam 5mg tablets |
| 50 | Paroxetine 20mg tablets |
| 301 | Venlafaxine 37.5mg tablets |
| 470 | Venlafaxine 75mg modified-release capsules |
| 527 | Paroxetine 10mg/5ml oral suspension sugar free |
| 595 | Amitriptyline 25mg / Perphenazine 2mg tablets |
| 603 | Escitalopram 10mg tablets |
| 609 | Perphenazine 2mg tablets |
| 623 | Efexor 37.5mg tablets (Wyeth Pharmaceuticals) |
| 648 | Cipralex 10mg tablets (Lundbeck Ltd) |
| 785 | Cipralex 5mg tablets (Lundbeck Ltd) |
| 790 | Pregabalin 25mg capsules |
| 819 | Pregabalin 75mg capsules |
| 840 | Fentazin 2mg tablets (AMCo) |
| 841 | Seroxat 20mg tablets (GlaxoSmithKline UK Ltd) |
| 1088 | Lorazepam 1mg tablets |
| 1159 | Trifluoperazine 2mg modified-release capsules |
| 1208 | Triptafen tablets (AMCo) |
| 1222 | Venlafaxine 75mg tablets |
| 1245 | Trifluoperazine 5mg tablets |
| 1316 | Stelazine 5mg tablets (Mercury Pharma Group Ltd) |
| 1318 | Stelazine 1mg tablets (Mercury Pharma Group Ltd) |
| 1333 | Oxprenolol 40mg tablets |
| 1334 | Oxprenolol 160mg modified-release tablets |
| 1397 | Paroxetine 30mg tablets |
| 1400 | Diazepam 10mg tablets |
| 1453 | Triptafen m 2mg+10mg Tablet (Goldshield Pharmaceuticals Ltd) |
| 1463 | Chlordiazepoxide 10mg capsules |
| 1474 | Efexor XL 75mg capsules (Pfizer Ltd) |
| 1575 | Seroxat 30mg tablets (GlaxoSmithKline UK Ltd) |
| 1730 | Trazodone 100mg capsules |
| 1735 | Stelazine 10mg Spansules (Mercury Pharma Group Ltd) |
| 1857 | Trifluoperazine 1mg tablets |
| 2078 | Diazepam 5mg RecTubes (Wockhardt UK Ltd) |
| 2083 | Diazepam 10mg RecTubes (Wockhardt UK Ltd) |
| 2091 | Lorazepam 2.5mg tablets |
| 2122 | Chlordiazepoxide 5mg capsules |
| 2157 | Perphenazine 4mg tablets |
| 2352 | Diazepam 2mg/5ml oral solution |
| 2361 | Trasicor 80mg Tablet (Novartis Pharmaceuticals UK Ltd) |
| 2394 | Buspar 5mg tablets (IXL Pharma Ltd) |
| 2401 | Valium 2mg Tablet (Roche Products Ltd) |
| 2617 | Venlafaxine 50mg tablets |
| 2654 | Venlafaxine 150mg modified-release capsules |
| 2713 | Stelazine 2mg Spansules (Mercury Pharma Group Ltd) |
| 2714 | Trifluoperazine 10mg modified-release capsules |
| 2780 | Oxprenolol 80mg tablets |
| 2828 | Meprobamate 400mg tablets |
| 2883 | Moclobemide 150mg tablets |
| 3205 | Diazepam 5mg |
| 3355 | Trazodone 50mg capsules |
| 3490 | Amitriptyline 10mg / Perphenazine 2mg tablets |
| 3516 | Oxprenolol 20mg tablets |
| 3574 | Buspirone 5mg tablets |
| 3601 | Seroxat 20mg/10ml liquid (GlaxoSmithKline UK Ltd) |
| 3639 | Meprobamate 200mg Tablet |
| 3870 | Diazepam 2mg capsules |
| 3937 | Trifluoperazine 15mg modified-release capsules |
| 3973 | Tensium 10mg tablets (DDSA Pharmaceuticals Ltd) |
| 4003 | Molipaxin 150mg tablets (Zentiva) |
| 4020 | Trazodone 150mg tablets |
| 4025 | Slow-Trasicor 160mg tablets (AMCo) |
| 4140 | Oxazepam 30mg Capsule |
| 4141 | Oxazepam 15mg tablets |
| 4176 | Diazepam 10mg/2.5ml rectal solution tube |
| 4194 | Molipaxin 100mg capsules (Zentiva) |
| 4338 | Valium 5mg Tablet (Roche Products Ltd) |
| 4395 | Stesolid 5mg rectal tube (Actavis UK Ltd) |
| 4566 | Oxazepam 10mg tablets |
| 4874 | Molipaxin 50mg capsules (Zentiva) |
| 5187 | Moclobemide 300mg tablets |
| 5294 | Chlordiazepoxide 10mg tablets |
| 5385 | Buspirone 10mg tablets |
| 5710 | Efexor XL 150mg capsules (Pfizer Ltd) |
| 5793 | Stesolid 10mg Rectal tubes (Dumex Ltd) |
| 5832 | Manerix 300mg tablets (Meda Pharmaceuticals Ltd) |
| 5842 | Diazepam 2.5mg/1.25ml rectal solution tube |
| 6025 | Chlordiazepoxide 5mg tablets |
| 6218 | Escitalopram 20mg tablets |
| 6274 | Efexor 50mg tablets (Wyeth Pharmaceuticals) |
| 6360 | Cipralex 20mg tablets (Lundbeck Ltd) |
| 6405 | Escitalopram 5mg tablets |
| 6442 | Trazodone 50mg/5ml oral solution sugar free |
| 6584 | Lyrica 75mg capsules (Pfizer Ltd) |
| 6631 | Pregabalin 150mg capsules |
| 6747 | Diazepam 5mg/2.5ml rectal solution tube |
| 6895 | Duloxetine 60mg gastro-resistant capsules |
| 6936 | Pregabalin 50mg capsules |
| 6949 | Pregabalin 200mg capsules |
| 6999 | Pregabalin 100mg capsules |
| 7005 | Pregabalin 300mg capsules |
| 7122 | Duloxetine 30mg gastro-resistant capsules |
| 7147 | Duloxetine 40mg gastro-resistant capsules |
| 7153 | Duloxetine 20mg gastro-resistant capsules |
| 7208 | Lyrica 100mg capsules (Pfizer Ltd) |
| 7209 | Lyrica 50mg capsules (Pfizer Ltd) |
| 7394 | Lyrica 200mg capsules (Pfizer Ltd) |
| 7474 | Trasicor 20mg Tablet (Novartis Pharmaceuticals UK Ltd) |
| 7833 | Neulactil 2.5mg Tablet (JHC Healthcare Ltd) |
| 7834 | Pericyazine 2.5mg tablets |
| 7919 | Fentazin 4mg tablets (AMCo) |
| 8031 | Neulactil 10mg Tablet (JHC Healthcare Ltd) |
| 8032 | Pericyazine 10mg tablets |
| 8042 | Stelazine 15mg Spansules (Mercury Pharma Group Ltd) |
| 8174 | Molipaxin 50mg/5ml oral liquid (Sanofi) |
| 8290 | Trasicor 40mg Tablet (Novartis Pharmaceuticals UK Ltd) |
| 8334 | Diazepam 10mg suppositories |
| 8344 | Diazepam 5mg suppository |
| 8345 | Stesolid 10mg rectal tube (Accord Healthcare Ltd) |
| 8721 | Oxazepam 30mg Tablet |
| 8913 | Librium 10mg Capsule (ICN Pharmaceuticals France S.A.) |
| 8985 | Stelazine 1mg/5ml syrup (Mercury Pharma Group Ltd) |
| 9008 | Buspar 10mg tablets (IXL Pharma Ltd) |
| 9045 | Diazepam 1mg/5ml suspension |
| 9048 | Librium 5mg Tablet (ICN Pharmaceuticals France S.A.) |
| 9065 | Diazepam 5mg/5ml oral solution |
| 9111 | Diazepam 10mg capsules |
| 9182 | Efexor 75mg tablets (Wyeth Pharmaceuticals) |
| 9206 | Manerix 150mg tablets (Meda Pharmaceuticals Ltd) |
| 9430 | Diazepam 2.5mg/5ml oral suspension |
| 9696 | Alprazolam 250microgram tablets |
| 10189 | Lyrica 300mg capsules (Pfizer Ltd) |
| 10274 | Diazepam 2mg/5ml oral solution sugar free |
| 10402 | Valium 10mg Tablet (Roche Products Ltd) |
| 10802 | Xanax 250microgram tablets (Pfizer Ltd) |
| 10954 | Ativan 1mg Tablet (Wyeth Pharmaceuticals) |
| 11486 | Alprazolam 500microgram tablets |
| 11531 | Trifluoperazine 5mg/5ml oral solution sugar free |
| 11963 | Limbitrol 10 Capsule (Roche Products Ltd) |
| 12195 | Pericyazine 10mg/5ml oral solution |
| 12237 | Diazepam 10mg/2ml emulsion for injection ampoules |
| 12477 | Librium 5mg Capsule (ICN Pharmaceuticals France S.A.) |
| 12484 | Equanil 200mg Tablet (Wyeth Pharmaceuticals) |
| 12512 | Equanil 400mg Tablet (Wyeth Pharmaceuticals) |
| 12598 | Xanax 500microgram tablets (Pfizer Ltd) |
| 12710 | Trazodone 150mg modified-release tablets |
| 12849 | Diazepam 10mg/5ml oral suspension |
| 13145 | Trifluoperazine 1mg/5ml oral solution sugar free |
| 13151 | Cymbalta 30mg gastro-resistant capsules (Eli Lilly and Company Ltd) |
| 13237 | Venlafaxine 37.5mg/5ml oral suspension |
| 13279 | Lorazepam 500micrograms/5ml oral suspension |
| 13621 | Molipaxin CR 150mg tablets (Aventis Pharma) |
| 13756 | Diazemuls 10mg/2ml emulsion for injection ampoules (Accord Healthcare Ltd) |
| 13902 | Neulactil Forte syrup (Sanofi) |
| 14534 | Limbitrol 5 Capsule (Roche Products Ltd) |
| 14803 | Yentreve 40mg gastro-resistant capsules (Eli Lilly and Company Ltd) |
| 14849 | Cymbalta 60mg gastro-resistant capsules (Eli Lilly and Company Ltd) |
| 14987 | Perphenazine 2mg/5ml oral solution sugar free |
| 15472 | Pericyazine 25mg tablet |
| 16509 | Lyrica 150mg capsules (Pfizer Ltd) |
| 16542 | Lyrica 25mg capsules (Pfizer Ltd) |
| 16734 | Diazepam rectubes 20mg Rectal tubes (C P Pharmaceuticals Ltd) |
| 16969 | Yentreve 20mg gastro-resistant capsules (Eli Lilly and Company Ltd) |
| 17294 | Librium 10mg Tablet (ICN Pharmaceuticals France S.A.) |
| 17830 | Ativan 2.5mg Tablet (Wyeth Pharmaceuticals) |
| 18125 | Librium 5mg capsules (Meda Pharmaceuticals Ltd) |
| 18342 | Amitriptyline 25mg / Chlordiazepoxide 10mg capsules |
| 18488 | Diazepam 2.5mg RecTubes (Wockhardt UK Ltd) |
| 19181 | Trazodone 100mg capsules (Mylan) |
| 19299 | Valium 5mg Capsule (Roche Products Ltd) |
| 20152 | Escitalopram 10mg/ml oral drops sugar free |
| 20164 | Valium 2mg Capsule (Roche Products Ltd) |
| 20514 | Valium 10mg Suppository (Roche Products Ltd) |
| 20968 | Diazepam 2mg/5ml oral solution sugar free (Actavis UK Ltd) |
| 21064 | Neulactil 25mg Tablet (JHC Healthcare Ltd) |
| 21081 | Amitriptyline 12.5mg / Chlordiazepoxide 5mg capsules |
| 23820 | Diazepam 20mg rectal tubes |
| 24094 | Trasicor 40mg tablets (Amdipharm Plc) |
| 24599 | Librium 10mg capsules (Meda Pharmaceuticals Ltd) |
| 24642 | Milonorm 400mg Tablet (Wallace Manufacturing Chemists Ltd) |
| 25273 | Oxanid 10mg Tablet (M A Steinhard Ltd) |
| 25644 | Apsolox 80mg Tablet (Approved Prescription Services Ltd) |
| 25909 | Perphenazine 4mg/5ml Oral solution sugar free |
| 26056 | Cipralex 10mg/ml oral drops (Lundbeck Ltd) |
| 27357 | Oxprenolol 40mg Tablet (Actavis UK Ltd) |
| 27880 | Tropium 5mg capsules (Dr Reddy's Laboratories (UK) Ltd) |
| 28347 | Diazepam 2mg Tablet (Crosspharma Ltd) |
| 28698 | Valium 5mg Suppository (Roche Products Ltd) |
| 28703 | Evacalm 5mg Tablet (Unimed Pharmaceuticals Ltd) |
| 28879 | Chlordiazepoxide 5mg Tablet (DDSA Pharmaceuticals Ltd) |
| 28880 | Buspirone 5mg Tablet (Galen Ltd) |
| 29180 | Trasicor 80mg tablets (Amdipharm Plc) |
| 29230 | Slow-pren 160mg Tablet (IVAX Pharmaceuticals UK Ltd) |
| 29339 | Trazodone 50mg capsules (Mylan) |
| 29857 | Trazodone 150mg tablets (Teva UK Ltd) |
| 29945 | Diazepam 2mg tablets (Ranbaxy (UK) Ltd) |
| 29948 | Stelazine Forte 1mg/ml oral solution (Mercury Pharma Group Ltd) |
| 30273 | Tropium 5mg tablets (Dr Reddy's Laboratories (UK) Ltd) |
| 30321 | Valium 2mg/5ml Oral solution (Roche Products Ltd) |
| 30983 | Trazodone 150mg tablets (Mylan) |
| 31633 | Valclair 10mg suppositories (Durbin Plc) |
| 32296 | Diazepam 5mg tablets (Actavis UK Ltd) |
| 32853 | Diazepam 2mg/5ml oral solution (Sandoz Ltd) |
| 32899 | Paroxetine 20mg tablets (Actavis UK Ltd) |
| 33070 | Solis 5mg Capsule (Galen Ltd) |
| 33086 | Lorazepam 1mg tablets (Teva UK Ltd) |
| 33569 | Oxprenolol sr 160mg Modified-release tablet (Hillcross Pharmaceuticals Ltd) |
| 33672 | Diazepam 2mg tablets (A A H Pharmaceuticals Ltd) |
| 33978 | Paroxetine 20mg tablets (Mylan) |
| 34003 | Trazodone 50mg capsules (A A H Pharmaceuticals Ltd) |
| 34033 | Diazepam 5mg/2.5ml rectal solution tube (Sandoz Ltd) |
| 34045 | Diazepam 5mg/5ml oral solution (Sandoz Ltd) |
| 34293 | Diazepam 10mg tablets (Mylan) |
| 34335 | Diazepam 2mg tablets (Teva UK Ltd) |
| 34338 | Diazepam 2mg tablets (Mylan) |
| 34340 | Diazepam 10mg tablets (Ranbaxy (UK) Ltd) |
| 34351 | Paroxetine 20mg tablets (IVAX Pharmaceuticals UK Ltd) |
| 34419 | Paroxetine 20mg tablets (A A H Pharmaceuticals Ltd) |
| 34421 | Trazodone 50mg capsules (Zentiva) |
| 34470 | Trazodone 150mg tablets (Zentiva) |
| 34482 | Diazepam 5mg tablets (Teva UK Ltd) |
| 34524 | Diazepam 2mg tablets (Actavis UK Ltd) |
| 34561 | Diazepam 2mg Tablet (Regent Laboratories Ltd) |
| 34580 | Trazodone 100mg capsules (A A H Pharmaceuticals Ltd) |
| 34587 | Paroxetine 30mg tablets (A A H Pharmaceuticals Ltd) |
| 34614 | Diazepam 10mg/2.5ml rectal solution tube (Sandoz Ltd) |
| 34615 | Diazepam 5mg tablets (Mylan) |
| 34635 | Diazepam 5mg tablets (A A H Pharmaceuticals Ltd) |
| 34677 | Diazepam 2mg tablets (IVAX Pharmaceuticals UK Ltd) |
| 34681 | Diazepam 5mg Tablet (Crosspharma Ltd) |
| 34807 | Diazepam 10mg tablets (Actavis UK Ltd) |
| 34876 | Diazepam 2mg Tablet (Berk Pharmaceuticals Ltd) |
| 34892 | Diazepam 5mg Tablet (Berk Pharmaceuticals Ltd) |
| 34928 | Chlordiazepoxide 5mg Capsule (DDSA Pharmaceuticals Ltd) |
| 35021 | Paroxetine 10mg tablets |
| 35062 | Trasicor 20mg tablets (Amdipharm Plc) |
| 35112 | Seroxat 10mg tablets (GlaxoSmithKline UK Ltd) |
| 35932 | Lorazepam 2.5mg tablets (Teva UK Ltd) |
| 35936 | Chlordiazepoxide 5mg capsules (A A H Pharmaceuticals Ltd) |
| 36200 | Lorazepam 1mg tablets (Mylan) |
| 36581 | Atensine 10mg Tablet (Rorer Pharmaceuticals Ltd) |
| 36604 | Oxazepam 10mg tablets (A A H Pharmaceuticals Ltd) |
| 37566 | Lorazepam 1mg/5ml oral suspension |
| 37745 | Lorazepam 1mg/5ml oral solution |
| 37801 | Pregabalin 225mg capsules |
| 38293 | Lyrica 225mg capsules (Pfizer Ltd) |
| 38410 | Diazepam 5mg Rectal tubes (Hillcross Pharmaceuticals Ltd) |
| 38827 | Triptafen-M tablets (Mercury Pharma Group Ltd) |
| 39284 | Lorazepam 1mg tablets (Genus Pharmaceuticals Ltd) |
| 39359 | Venlafaxine 75mg modified-release tablets |
| 39360 | Venlafaxine 150mg modified-release tablets |
| 39770 | Tifaxin XL 75mg capsules (Genus Pharmaceuticals Ltd) |
| 39809 | Tifaxin XL 150mg capsules (Genus Pharmaceuticals Ltd) |
| 39830 | Neulactil 2.5mg tablets (Sanofi) |
| 40048 | ViePax XL 75mg tablets (Dexcel-Pharma Ltd) |
| 40049 | ViePax XL 150mg tablets (Dexcel-Pharma Ltd) |
| 40054 | Venlafaxine 225mg modified-release tablets |
| 40059 | Venlalic XL 75mg tablets (Ethypharm UK Ltd) |
| 40062 | Venlalic XL 150mg tablets (Ethypharm UK Ltd) |
| 40092 | Vensir XL 150mg capsules (Morningside Healthcare Ltd) |
| 40153 | Buspirone 10mg Tablet (Galen Ltd) |
| 40162 | Trifluoperazine 1mg tablets (A A H Pharmaceuticals Ltd) |
| 40165 | Paroxetine 30mg tablets (Actavis UK Ltd) |
| 40277 | Vensir XL 75mg capsules (Morningside Healthcare Ltd) |
| 40407 | Venlalic XL 225mg tablets (Ethypharm UK Ltd) |
| 40514 | Venaxx XL 150mg capsules (AMCo) |
| 40515 | Venaxx XL 75mg capsules (AMCo) |
| 40517 | Vexarin XL 150mg capsules (Mylan) |
| 40726 | Escitalopram 20mg/ml oral drops sugar free |
| 40764 | ViePax 37.5mg tablets (Dexcel-Pharma Ltd) |
| 40815 | Tardcaps XL 75mg capsules (IXL Pharma Ltd) |
| 40817 | Tardcaps XL 150mg capsules (IXL Pharma Ltd) |
| 40881 | Neulactil 10mg tablets (Sanofi) |
| 40892 | Paroxetine 20mg tablets (Genus Pharmaceuticals Ltd) |
| 40917 | ViePax 75mg tablets (Dexcel-Pharma Ltd) |
| 41033 | Rodomel XL 75mg capsules (Teva UK Ltd) |
| 41062 | Cipralex 20mg/ml oral drops (Lundbeck Ltd) |
| 41299 | Politid XL 75mg capsules (Actavis UK Ltd) |
| 41314 | Rodomel XL 150mg capsules (Teva UK Ltd) |
| 41391 | Lorazepam 1mg tablets (Arrow Generics Ltd) |
| 41411 | Oxazepam 15mg tablets (A A H Pharmaceuticals Ltd) |
| 41531 | Oxazepam 10mg tablets (Actavis UK Ltd) |
| 41542 | Oxazepam 10mg Tablet (IVAX Pharmaceuticals UK Ltd) |
| 41553 | Oxazepam 10mg tablets (Thornton & Ross Ltd) |
| 41574 | Chlordiazepoxide 10mg Capsule (Approved Prescription Services Ltd) |
| 41581 | Chlordiazepoxide 10mg Capsule (IVAX Pharmaceuticals UK Ltd) |
| 41582 | Chlordiazepoxide 5mg capsules (Actavis UK Ltd) |
| 41583 | Chlordiazepoxide 10mg capsules (Actavis UK Ltd) |
| 41601 | Oxazepam 15mg Tablet (IVAX Pharmaceuticals UK Ltd) |
| 41602 | Oxazepam 15mg tablets (Actavis UK Ltd) |
| 41606 | Chlordiazepoxide 5mg Capsule (Approved Prescription Services Ltd) |
| 41607 | Diazepam 10mg tablets (Teva UK Ltd) |
| 41609 | Trazodone 50mg capsules (Teva UK Ltd) |
| 41629 | Chlordiazepoxide 10mg Capsule (DDSA Pharmaceuticals Ltd) |
| 41632 | Diazepam 10mg tablets (A A H Pharmaceuticals Ltd) |
| 41663 | Trifluoperazine 5mg tablets (A A H Pharmaceuticals Ltd) |
| 41689 | Diazepam 10mg Suppository (Sinclair IS Pharma Plc) |
| 41709 | Trazodone 100mg capsules (Teva UK Ltd) |
| 41710 | Trazodone 100mg capsules (Zentiva) |
| 41747 | Moclobemide 150mg tablets (Teva UK Ltd) |
| 41988 | Chlordiazepoxide 10mg Tablet (DDSA Pharmaceuticals Ltd) |
| 42503 | Diazepam 5mg/5ml oral solution (A A H Pharmaceuticals Ltd) |
| 42600 | Vexarin XL 75mg capsules (Mylan) |
| 42814 | Lorazepam 2.5mg tablets (Genus Pharmaceuticals Ltd) |
| 43203 | Venlafaxine 75mg modified-release capsules (Sandoz Ltd) |
| 43240 | Buspirone 5mg tablets (Mylan) |
| 43334 | Venlafaxine 150mg modified-release capsules (Sandoz Ltd) |
| 43438 | Chlordiazepoxide 5mg tablets (A A H Pharmaceuticals Ltd) |
| 43673 | Politid XL 150mg capsules (Actavis UK Ltd) |
| 43968 | Foraven XL 75mg capsules (Forum Products Ltd) |
| 44302 | Tropium 10mg tablets (Dr Reddy's Laboratories (UK) Ltd) |
| 44936 | Venlaneo XL 150mg capsules (Kent Pharmaceuticals Ltd) |
| 44937 | Venlaneo XL 75mg capsules (Kent Pharmaceuticals Ltd) |
| 45135 | Diazepam 2mg Tablet (M & A Pharmachem Ltd) |
| 45218 | Diazepam 5mg tablets (Ranbaxy (UK) Ltd) |
| 45241 | Chlordiazepoxide 10mg capsules (A A H Pharmaceuticals Ltd) |
| 45244 | Diazepam 5mg tablets (Sandoz Ltd) |
| 45275 | Buspirone 5mg tablets (Actavis UK Ltd) |
| 45313 | Diazepam 2mg tablets (Almus Pharmaceuticals Ltd) |
| 45664 | Depefex XL 150mg capsules (Chiesi Ltd) |
| 45806 | Venlafaxine 37.5mg modified-release tablets |
| 45818 | Venlalic XL 37.5mg tablets (Ethypharm UK Ltd) |
| 45829 | Lorazepam 1mg tablets (Sandoz Ltd) |
| 45959 | Depefex XL 75mg capsules (Chiesi Ltd) |
| 46847 | Buspirone 10mg tablets (Actavis UK Ltd) |
| 46896 | Lorazepam 500micrograms/5ml oral solution |
| 46913 | Diazepam 10mg tablets (IVAX Pharmaceuticals UK Ltd) |
| 46946 | Oxazepam 15mg tablets (Thornton & Ross Ltd) |
| 46966 | Diazepam 5mg tablets (IVAX Pharmaceuticals UK Ltd) |
| 48199 | Ranfaxine XL 75mg capsules (Ranbaxy (UK) Ltd) |
| 48253 | Lyrica 150mg capsules (Lexon (UK) Ltd) |
| 49504 | Buspar 10mg tablets (Lexon (UK) Ltd) |
| 49511 | Venlablue XL 75mg capsules (Creo Pharma Ltd) |
| 50081 | Venlablue XL 150mg capsules (Creo Pharma Ltd) |
| 50934 | Venlafaxine 150mg/5ml oral solution |
| 51227 | Pregabalin 20mg/ml oral solution sugar free |
| 51280 | Efexor XL 150mg capsules (Waymade Healthcare Plc) |
| 51335 | Diazepam 10mg/5ml oral solution |
| 51361 | Venlafaxine 37.5mg tablets (Ranbaxy (UK) Ltd) |
| 51383 | Duloxetine 60mg gastro-resistant capsules (Sigma Pharmaceuticals Plc) |
| 51699 | Venlafaxine 37.5mg/5ml oral solution |
| 51924 | Lyrica 20mg/ml oral solution (Pfizer Ltd) |
| 51985 | Diazepam 2mg/5ml oral suspension |
| 52074 | Alventa XL 75mg capsules (Consilient Health Ltd) |
| 52516 | Alventa XL 150mg capsules (Consilient Health Ltd) |
| 52547 | Lyrica 50mg capsules (Waymade Healthcare Plc) |
| 52716 | Tonpular XL 75mg capsules (Wockhardt UK Ltd) |
| 53326 | Venlafaxine 75mg/5ml oral solution |
| 53461 | Diazepam 2mg/5ml oral solution sugar free (A A H Pharmaceuticals Ltd) |
| 53566 | Diazepam 2.5mg/5ml oral solution |
| 54695 | Diazepam 10mg Tablet (M & A Pharmachem Ltd) |
| 55023 | Paroxetine 20mg tablets (Medreich Plc) |
| 55137 | Trazodone 150mg/5ml oral suspension |
| 55138 | Trazodone 250mg/5ml oral solution |
| 55382 | Trifluoperazine 1mg/5ml oral solution sugar free (AMCo) |
| 55501 | Venlafaxine 150mg Modified-release capsule (Hillcross Pharmaceuticals Ltd) |
| 55537 | Seroxat 30mg tablets (Lexon (UK) Ltd) |
| 55972 | Pregabalin 150mg/5ml oral solution |
| 56236 | Diazepam 2mg tablets (Wockhardt UK Ltd) |
| 56457 | Venlafaxine 75mg tablets (Teva UK Ltd) |
| 56551 | Lorazepam 5mg/5ml oral solution |
| 56662 | Venlafaxine 37.5mg tablets (A A H Pharmaceuticals Ltd) |
| 57226 | Trazodone 25mg/5ml oral suspension |
| 57268 | Lorazepam 2.5mg tablets (Sandoz Ltd) |
| 57605 | Stelazine 1mg tablets (Lexon (UK) Ltd) |
| 57749 | Diazepam 5mg tablets (Waymade Healthcare Plc) |
| 57751 | Tonpular XL 150mg capsules (Wockhardt UK Ltd) |
| 57838 | Diazepam 5mg tablets (Sovereign Medical Ltd) |
| 58681 | Venladex XL 75mg tablets (Dexcel-Pharma Ltd) |
| 58726 | Venladex XL 150mg tablets (Dexcel-Pharma Ltd) |
| 58837 | Venlafaxine 37.5mg modified-release capsules |
| 58959 | Diazepam 10mg/5ml oral solution (AM Distributions (Yorkshire) Ltd) |
| 59035 | Venlablue XL 37.5mg capsules (Creo Pharma Ltd) |
| 59095 | Buspirone 5mg tablets (A A H Pharmaceuticals Ltd) |
| 59122 | Diazepam 5mg/5ml oral solution (AM Distributions (Yorkshire) Ltd) |
| 59288 | Paroxetine 10mg tablets (Actavis UK Ltd) |
| 59407 | Diazepam 5mg tablets (DE Pharmaceuticals) |
| 59563 | Venlafaxine 75mg modified-release capsules (Kent Pharmaceuticals Ltd) |
| 59753 | Sunveniz XL 150mg tablets (Sun Pharmaceuticals UK Ltd) |
| 59923 | Venlafaxine 37.5mg tablets (Bristol Laboratories Ltd) |
| 59931 | Trazodone 50mg/5ml oral solution sugar free (A A H Pharmaceuticals Ltd) |
| 60449 | Venlafaxine 75mg tablets (A A H Pharmaceuticals Ltd) |
| 60543 | Pregabalin 75mg/5ml oral solution |
| 60549 | Venlafaxine 150mg modified-release capsules (Kent Pharmaceuticals Ltd) |
| 60843 | Sunveniz XL 75mg tablets (Sun Pharmaceuticals UK Ltd) |
| 60895 | Venlafaxine 37.5mg tablets (Teva UK Ltd) |
| 60936 | Diazepam 5mg tablets (Arrow Generics Ltd) |
| 61236 | Bonilux XL 150mg capsules (Sandoz Ltd) |
| 61443 | Buspirone 10mg tablets (A A H Pharmaceuticals Ltd) |
| 61450 | Lorazepam 1mg tablets (A A H Pharmaceuticals Ltd) |
| 61657 | Trazodone 75mg/5ml oral solution |
| 61842 | Trazodone 50mg/5ml oral solution |
| 61886 | Lorazepam 5mg/5ml oral suspension |
| 62216 | Diazepam 10mg tablets (Almus Pharmaceuticals Ltd) |
| 62270 | Chlordiazepoxide 10mg tablets (A A H Pharmaceuticals Ltd) |
| 62541 | Diazepam 2mg tablets (Sovereign Medical Ltd) |
| 62688 | Duloxetine 30mg gastro-resistant capsules (Sigma Pharmaceuticals Plc) |
| 62734 | Venlafaxine 150mg/5ml oral suspension |
| 63069 | Rewisca 75mg capsules (Consilient Health Ltd) |
| 63088 | Rewisca 50mg capsules (Consilient Health Ltd) |
| 63089 | Rewisca 300mg capsules (Consilient Health Ltd) |
| 63090 | Rewisca 150mg capsules (Consilient Health Ltd) |
| 63091 | Rewisca 100mg capsules (Consilient Health Ltd) |
| 63174 | Rewisca 200mg capsules (Consilient Health Ltd) |
| 63216 | Cymbalta 60mg gastro-resistant capsules (Mawdsley-Brooks & Company Ltd) |
| 63238 | Diazepam 2mg tablets (Waymade Healthcare Plc) |
| 63268 | Venlafaxine 75mg/5ml oral suspension |
| 63300 | Rewisca 25mg capsules (Consilient Health Ltd) |
| 63317 | Rewisca 225mg capsules (Consilient Health Ltd) |
| 63370 | Duloxetine 30mg gastro-resistant capsules (Mawdsley-Brooks & Company Ltd) |
| 63694 | Diazepam 2mg tablets (Phoenix Healthcare Distribution Ltd) |
| 63763 | Duloxetine 60mg gastro-resistant capsules (A A H Pharmaceuticals Ltd) |
| 63859 | Venlafaxine 75mg tablets (Waymade Healthcare Plc) |
| 63877 | Pregabalin 50mg capsules (A A H Pharmaceuticals Ltd) |
| 63916 | Escitalopram 10mg tablets (Actavis UK Ltd) |
| 63964 | Alzain 25mg capsules (Dr Reddy's Laboratories (UK) Ltd) |
| 63965 | Alzain 100mg capsules (Dr Reddy's Laboratories (UK) Ltd) |
| 64005 | Pregabalin 100mg capsules (A A H Pharmaceuticals Ltd) |
| 64037 | Alzain 300mg capsules (Dr Reddy's Laboratories (UK) Ltd) |
| 64038 | Alzain 225mg capsules (Dr Reddy's Laboratories (UK) Ltd) |
| 64039 | Alzain 150mg capsules (Dr Reddy's Laboratories (UK) Ltd) |
| 64040 | Alzain 75mg capsules (Dr Reddy's Laboratories (UK) Ltd) |
| 64041 | Alzain 50mg capsules (Dr Reddy's Laboratories (UK) Ltd) |
| 64042 | Alzain 200mg capsules (Dr Reddy's Laboratories (UK) Ltd) |
| 64200 | Diazepam 2mg tablets (Alliance Healthcare (Distribution) Ltd) |
| 64216 | Stelazine 5mg tablets (Imported (South Africa)) |
| 64285 | Pregabalin 150mg/5ml oral suspension |
| 64442 | Duloxetine 60mg gastro-resistant capsules (Teva UK Ltd) |
| 64470 | Chlordiazepoxide 10mg tablets (Phoenix Healthcare Distribution Ltd) |
| 64497 | Lyrica 75mg capsules (Waymade Healthcare Plc) |
| 64568 | Lyrica 150mg capsules (Sigma Pharmaceuticals Plc) |
| 64729 | Lorazepam 1mg tablets (Genesis Pharmaceuticals Ltd) |
| 64785 | Paroxetine 30mg tablets (Alliance Healthcare (Distribution) Ltd) |
| 64876 | Lorazepam 2mg/5ml oral suspension |
| 65069 | Pregabalin 75mg capsules (Teva UK Ltd) |
| 65073 | Pregabalin 25mg capsules (Teva UK Ltd) |
| 65152 | Trazodone 100mg/5ml oral solution |
| 65165 | Duloxetine 20mg gastro-resistant capsules (DE Pharmaceuticals) |
| 65218 | Lecaent 50mg capsules (Actavis UK Ltd) |
| 65606 | Pregabalin 50mg capsules (Accord Healthcare Ltd) |
| 65618 | Duloxetine 30mg gastro-resistant capsules (A A H Pharmaceuticals Ltd) |
| 65666 | Venlafaxine 225mg modified-release capsules |
| 65738 | Efexor 37.5mg tablets (Sigma Pharmaceuticals Plc) |
| 65787 | Pregabalin 50mg capsules (Sandoz Ltd) |
| 65809 | Duloxetine 30mg gastro-resistant capsules (Actavis UK Ltd) |
| 65863 | Lyrica 75mg capsules (Stephar (U.K.) Ltd) |
| 65888 | Duloxetine 60mg gastro-resistant capsules (Actavis UK Ltd) |
| 65892 | Duloxetine 60mg gastro-resistant capsules (Mawdsley-Brooks & Company Ltd) |
| 65899 | Efexor XL 225mg capsules (Pfizer Ltd) |
| 66292 | Seroxat 10mg tablets (Waymade Healthcare Plc) |
| 66405 | Duloxetine 60mg gastro-resistant capsules (DE Pharmaceuticals) |
| 66412 | Duloxetine 30mg gastro-resistant capsules (DE Pharmaceuticals) |
| 66437 | Venlafaxine 75mg tablets (DE Pharmaceuticals) |
| 66509 | Pregabalin 75mg capsules (Zentiva) |
| 66749 | Trazodone 10mg/5ml oral solution |
| 66941 | Lecaent 75mg capsules (Actavis UK Ltd) |
| 67053 | Pregabalin 75mg capsules (DE Pharmaceuticals) |
| 67184 | Lyrica 50mg capsules (Mawdsley-Brooks & Company Ltd) |
| 67192 | Diazepam 5mg/5ml oral suspension (Sandoz Ltd) |
| 67193 | Diazepam 2mg/5ml oral suspension (Sandoz Ltd) |
| 67259 | Paroxetine 10mg/5ml oral solution |
| 67263 | Buspar 5mg tablets (Dowelhurst Ltd) |
| 67271 | Efexor 37.5mg tablets (Waymade Healthcare Plc) |
| 67288 | Efexor 75mg tablets (Dowelhurst Ltd) |
| 67297 | Diazepam 25mg/5ml oral solution |
| 67305 | Moclobemide 150mg tablets (Sigma Pharmaceuticals Plc) |
| 67384 | Pregabalin 75mg/5ml oral suspension |
| 67440 | Pregabalin 300mg capsules (A A H Pharmaceuticals Ltd) |
| 67451 | Diazepam 2mg tablets (Mawdsley-Brooks & Company Ltd) |
| 67554 | Lorazepam 1mg tablets (Almus Pharmaceuticals Ltd) |
| 67563 | Vensir XL 225mg capsules (Morningside Healthcare Ltd) |
| 67564 | Duloxetine 20mg gastro-resistant capsules (Actavis UK Ltd) |
| 67777 | Pericyazine 2.5mg tablets (Zentiva) |
| 67785 | Diazepam 5mg tablets (Wockhardt UK Ltd) |
| 67805 | Stelazine 1mg tablets (Imported (South Africa)) |
| 67957 | Diazepam 5mg/5ml oral suspension (A A H Pharmaceuticals Ltd) |
| 68014 | Lyrica 75mg capsules (Lexon (UK) Ltd) |
| 68050 | Venlafaxine 37.5mg tablets (Alliance Healthcare (Distribution) Ltd) |
| 68096 | Duloxetine 60mg gastro-resistant capsules (Zentiva) |
| 68325 | Paroxetine 40mg tablets |
| 68414 | Diazepam 2mg/5ml oral solution sugar free (Alliance Healthcare (Distribution) Ltd) |
| 68441 | Pregabalin 25mg/5ml oral solution |
| 68876 | Venlafaxine 75mg modified-release capsules (Mawdsley-Brooks & Company Ltd) |
| 69034 | Pregabalin 300mg capsules (Mylan) |
| 69125 | Axalid 100mg capsules (Kent Pharmaceuticals Ltd) |
| 69296 | Axalid 25mg capsules (Kent Pharmaceuticals Ltd) |
| 69418 | Axalid 300mg capsules (Kent Pharmaceuticals Ltd) |
| 69428 | Duloxetine 60mg gastro-resistant capsules (Alliance Healthcare (Distribution) Ltd) |
| 69497 | Axalid 225mg capsules (Kent Pharmaceuticals Ltd) |
| 69498 | Axalid 75mg capsules (Kent Pharmaceuticals Ltd) |
| 69499 | Axalid 150mg capsules (Kent Pharmaceuticals Ltd) |
| 69501 | Lyrica 75mg capsules (DE Pharmaceuticals) |
| 69554 | Axalid 50mg capsules (Kent Pharmaceuticals Ltd) |
| 69752 | Duloxetine 60mg gastro-resistant capsules (Creo Pharma Ltd) |
| 69781 | Lyrica 25mg capsules (Lexon (UK) Ltd) |
| 69783 | Diazepam 2mg/5ml oral suspension (A A H Pharmaceuticals Ltd) |
| 69799 | Axalid 200mg capsules (Kent Pharmaceuticals Ltd) |
| 69810 | Diazepam 5mg tablets (Crescent Pharma Ltd) |
| 69819 | Vencarm XL 37.5mg capsules (Aspire Pharma Ltd) |
| 69877 | Lecaent 300mg capsules (Actavis UK Ltd) |
| 69965 | Duloxetine 60mg gastro-resistant capsules (Consilient Health Ltd) |
| 69987 | Pregabalin 75mg capsules (A A H Pharmaceuticals Ltd) |
| 70063 | Duloxetine 20mg gastro-resistant capsules (A A H Pharmaceuticals Ltd) |
| 70064 | Pregabalin 25mg capsules (Accord Healthcare Ltd) |
| 70229 | Pregabalin 75mg capsules (Mylan) |
| 70315 | Vencarm XL 75mg capsules (Aspire Pharma Ltd) |
| 70353 | Venlafaxine 37.5mg tablets (DE Pharmaceuticals) |
| 70405 | Duloxetine 30mg gastro-resistant capsules (Teva UK Ltd) |
| 70420 | Vencarm XL 150mg capsules (Aspire Pharma Ltd) |
| 70478 | Pregabalin 150mg capsules (Teva UK Ltd) |
| 70495 | Vencarm XL 225mg capsules (Aspire Pharma Ltd) |
| 70521 | Trazodone 50mg/5ml oral solution sugar free (AMCo) |
| 70536 | Diazepam 1mg/5ml oral solution |
| 70544 | Pregabalin 150mg capsules (Alliance Healthcare (Distribution) Ltd) |
| 70545 | Pregabalin 25mg capsules (Alliance Healthcare (Distribution) Ltd) |
| 70546 | Pregabalin 50mg capsules (Alliance Healthcare (Distribution) Ltd) |
| 70648 | Pregabalin 300mg capsules (Teva UK Ltd) |
| 70682 | Lorazepam 1mg/ml oral solution sugar free |
| 70728 | Duloxetine 30mg gastro-resistant capsules (Alliance Healthcare (Distribution) Ltd) |
| 70729 | Pregabalin 200mg capsules (Alliance Healthcare (Distribution) Ltd) |
| 70730 | Pregabalin 100mg capsules (Alliance Healthcare (Distribution) Ltd) |
| 70731 | Pregabalin 75mg capsules (Alliance Healthcare (Distribution) Ltd) |
| 70735 | Pregabalin 300mg capsules (Alliance Healthcare (Distribution) Ltd) |
| 70806 | Venlafaxine 150mg modified-release capsules (DE Pharmaceuticals) |
| 70931 | Venlasov XL 75mg capsules (Sovereign Medical Ltd) |
| 71031 | Trazodone 50mg capsules (Actavis UK Ltd) |
| 71221 | Lecaent 200mg capsules (Actavis UK Ltd) |
| 71245 | Diazepam 5mg tablets (Relonchem Ltd) |
| 71257 | Venlafaxine 75mg modified-release capsules (DE Pharmaceuticals) |
| 71313 | Pregabalin 50mg capsules (Zentiva) |
| 71336 | Diazepam 2mg tablets (DE Pharmaceuticals) |
| 71387 | Stelazine 5mg tablets (Lexon (UK) Ltd) |
| 71461 | Pregabalin 50mg/5ml oral suspension |
| 71533 | Pregabalin 50mg capsules (Mylan) |
| 71659 | Lecaent 25mg capsules (Actavis UK Ltd) |
| 71669 | Duloxetine 20mg gastro-resistant capsules (Zentiva) |
| 71782 | Venlafaxine 150mg modified-release capsules (Mawdsley-Brooks & Company Ltd) |
| 71806 | Amphero XL 75mg capsules (Mylan) |
| 71932 | Venlasov XL 150mg capsules (Sovereign Medical Ltd) |
| 72068 | Lecaent 150mg capsules (Actavis UK Ltd) |
| 72211 | Duloxetine 60mg gastro-resistant capsules (Dr Reddy's Laboratories (UK) Ltd) |
| 72291 | Trazodone 100mg/5ml oral solution sugar free |

# S1 Table D Codelist for anxiety – sensitivity analysis.

| medcode | readcode | Read term |
| --- | --- | --- |
| 962 | Eu41111 | [X]Anxiety neurosis |
| 2571 | Eu40000 | [X]Agoraphobia |
| 4081 | Eu41012 | [X]Panic state |
| 4171 | Eu43100 | [X]Post - traumatic stress disorder |
| 5304 | Eu42.00 | [X]Obsessive - compulsive disorder |
| 5385 | Eu41.00 | [X]Other anxiety disorders |
| 6408 | Eu41011 | [X]Panic attack |
| 7749 | Eu41211 | [X]Mild anxiety depression |
| 8205 | Eu41000 | [X]Panic disorder [episodic paroxysmal anxiety] |
| 9386 | Eu40.00 | [X]Phobic anxiety disorders |
| 10344 | Eu41100 | [X]Generalized anxiety disorder |
| 10535 | Eu43012 | [X]Acute reaction to stress |
| 11098 | Eu43.00 | [X]Reaction to severe stress, and adjustment disorders |
| 11607 | Eu43000 | [X]Acute stress reaction |
| 11913 | Eu41200 | [X]Mixed anxiety and depressive disorder |
| 14890 | Eu40012 | [X]Panic disorder with agoraphobia |
| 15220 | Eu34114 | [X]Persistant anxiety depression |
| 16729 | Eu40011 | [X]Agoraphobia without history of panic disorder |
| 18399 | Eu42200 | [X]Mixed obsessional thoughts and acts |
| 20773 | Eu05400 | [X]Organic anxiety disorder |
| 21753 | Eu43y00 | [X]Other reactions to severe stress |
| 21836 | Eu42.12 | [X]Obsessive-compulsive neurosis |
| 22019 | Eu42100 | [X]Predominantly compulsive acts [obsessional rituals] |
| 22721 | Eu42z00 | [X]Obsessive-compulsive disorder, unspecified |
| 23838 | Eu41z00 | [X]Anxiety disorder, unspecified |
| 24066 | Eu41y00 | [X]Other specified anxiety disorders |
| 25638 | Eu41z11 | [X]Anxiety NOS |
| 27685 | Eu40y00 | [X]Other phobic anxiety disorders |
| 28167 | Eu41y11 | [X]Anxiety hysteria |
| 31515 | Eu43z00 | [X]Reaction to severe stress, unspecified |
| 34064 | Eu40z00 | [X]Phobic anxiety disorder, unspecified |
| 35825 | Eu41112 | [X]Anxiety reaction |
| 38809 | Eu42y00 | [X]Other obsessive-compulsive disorders |
| 44321 | Eu41300 | [X]Other mixed anxiety disorders |
| 50191 | Eu41113 | [X]Anxiety state |

# S1 Table E Codelist for depression – main analysis.

| medcode | readcode | Read term | Depression |
| --- | --- | --- | --- |
| 1996 | 1B17.00 | Depressed | Probable |
| 4824 | 1B17.11 | C/O - feeling depressed | Probable |
| 9796 | 1B1U.00 | Symptoms of depression | Probable |
| 10438 | 1B1U.11 | Depressive symptoms | Probable |
| 10015 | 1BT..00 | Depressed mood | Probable |
| 8928 | 1BT..11 | Low mood | Probable |
| 26028 | 1BT..12 | Sad mood | Probable |
| 1908 | 2257 | O/E - depressed | Probable |
| 44848 | 8BK0.00 | Depression management programme | Probable |
| 30483 | 8CAa.00 | Patient given advice about management of depression | Probable |
| 32841 | 8HHq.00 | Referral for guided self-help for depression | Probable |
| 12399 | 9H90.00 | Depression annual review | Probable |
| 12122 | 9H91.00 | Depression medication review | Probable |
| 30405 | 9H92.00 | Depression interim review | Probable |
| 42931 | 9HA0.00 | On depression register | Probable |
| 51258 | 9Ov..00 | Depression monitoring administration | Probable |
| 71009 | 9Ov0.00 | Depression monitoring first letter | Probable |
| 72966 | 9Ov1.00 | Depression monitoring second letter | Probable |
| 91105 | 9Ov2.00 | Depression monitoring third letter | Probable |
| 88644 | 9Ov3.00 | Depression monitoring verbal invite | Probable |
| 85852 | 9Ov4.00 | Depression monitoring telephone invite | Probable |
| 48970 | 9hC..00 | Exception reporting: depression quality indicators | Probable |
| 28970 | 9hC0.00 | Excepted from depression quality indicators: Patient unsuita | Probable |
| 43239 | 9hC1.00 | Excepted from depression quality indicators: Informed dissen | Probable |
| 30583 | 9k4..00 | Depression - enhanced services administration | Probable |
| 65435 | 9k40.00 | Depression - enhanced service completed | Probable |
| 96995 | 9kQ..00 | On full dose long term treatment depression - enh serv admin | Probable |
| 27677 | E001300 | Presenile dementia with depression | Probable |
| 21887 | E002100 | Senile dementia with depression | Probable |
| 41089 | E002z00 | Senile dementia with depressive or paranoid features NOS | Probable |
| 43292 | E004300 | Arteriosclerotic dementia with depression | Probable |
| 2560 | E11..12 | Depressive psychoses | Probable |
| 10610 | E112.00 | Single major depressive episode | Probable |
| 5879 | E112.11 | Agitated depression | Probable |
| 6546 | E112.12 | Endogenous depression first episode | Probable |
| 6950 | E112.13 | Endogenous depression first episode | Probable |
| 595 | E112.14 | Endogenous depression | Probable |
| 34390 | E112000 | Single major depressive episode, unspecified | Probable |
| 16506 | E112100 | Single major depressive episode, mild | Probable |
| 15155 | E112200 | Single major depressive episode, moderate | Probable |
| 15219 | E112300 | Single major depressive episode, severe, without psychosis | Probable |
| 32159 | E112400 | Single major depressive episode, severe, with psychosis | Probable |
| 7011 | E112z00 | Single major depressive episode NOS | Probable |
| 15099 | E113.00 | Recurrent major depressive episode | Probable |
| 6932 | E113.11 | Endogenous depression - recurrent | Probable |
| 35671 | E113000 | Recurrent major depressive episodes, unspecified | Probable |
| 29342 | E113100 | Recurrent major depressive episodes, mild | Probable |
| 14709 | E113200 | Recurrent major depressive episodes, moderate | Probable |
| 25697 | E113300 | Recurrent major depressive episodes, severe, no psychosis | Probable |
| 24171 | E113400 | Recurrent major depressive episodes, severe, with psychosis | Probable |
| 6482 | E113700 | Recurrent depression | Probable |
| 25563 | E113z00 | Recurrent major depressive episode NOS | Probable |
| 10825 | E118.00 | Seasonal affective disorder | Probable |
| 27491 | E11y200 | Atypical depressive disorder | Probable |
| 9183 | E11z200 | Masked depression | Probable |
| 8478 | E130.00 | Reactive depressive psychosis | Probable |
| 17770 | E130.11 | Psychotic reactive depression | Probable |
| 1055 | E135.00 | Agitated depression | Probable |
| 655 | E200300 | Anxiety with depression | Probable |
| 1131 | E204.00 | Neurotic depression reactive type | Probable |
| 1533 | E290.00 | Brief depressive reaction | Probable |
| 36246 | E290z00 | Brief depressive reaction NOS | Probable |
| 16632 | E291.00 | Prolonged depressive reaction | Probable |
| 324 | E2B..00 | Depressive disorder NEC | Probable |
| 2972 | E2B0.00 | Postviral depression | Probable |
| 4323 | E2B1.00 | Chronic depression | Probable |
| 27759 | Eu02z16 | [X] Senile dementia, depressed or paranoid type | Probable |
| 4639 | Eu32.00 | [X]Depressive episode | Probable |
| 9055 | Eu32.11 | [X]Single episode of depressive reaction | Probable |
| 18510 | Eu32.12 | [X]Single episode of psychogenic depression | Probable |
| 7604 | Eu32.13 | [X]Single episode of reactive depression | Probable |
| 11717 | Eu32000 | [X]Mild depressive episode | Probable |
| 9211 | Eu32100 | [X]Moderate depressive episode | Probable |
| 9667 | Eu32200 | [X]Severe depressive episode without psychotic symptoms | Probable |
| 41989 | Eu32211 | [X]Single episode agitated depressn w'out psychotic symptoms | Probable |
| 22806 | Eu32212 | [X]Single episode major depression w'out psychotic symptoms | Probable |
| 59386 | Eu32213 | [X]Single episode vital depression w'out psychotic symptoms | Probable |
| 12099 | Eu32300 | [X]Severe depressive episode with psychotic symptoms | Probable |
| 24117 | Eu32311 | [X]Single episode of major depression and psychotic symptoms | Probable |
| 52678 | Eu32312 | [X]Single episode of psychogenic depressive psychosis | Probable |
| 24112 | Eu32313 | [X]Single episode of psychotic depression | Probable |
| 28863 | Eu32314 | [X]Single episode of reactive depressive psychosis | Probable |
| 10667 | Eu32400 | [X]Mild depression | Probable |
| 98346 | Eu32500 | [X]Major depression, mild | Probable |
| 98252 | Eu32600 | [X]Major depression, moderately severe | Probable |
| 98414 | Eu32700 | [X]Major depression, severe without psychotic symptoms | Probable |
| 98417 | Eu32800 | [X]Major depression, severe with psychotic symptoms | Probable |
| 6854 | Eu32y00 | [X]Other depressive episodes | Probable |
| 10720 | Eu32y11 | [X]Atypical depression | Probable |
| 56609 | Eu32y12 | [X]Single episode of masked depression NOS | Probable |
| 2970 | Eu32z00 | [X]Depressive episode, unspecified | Probable |
| 543 | Eu32z11 | [X]Depression NOS | Probable |
| 3291 | Eu32z12 | [X]Depressive disorder NOS | Probable |
| 28248 | Eu32z13 | [X]Prolonged single episode of reactive depression | Probable |
| 5987 | Eu32z14 | [X] Reactive depression NOS | Probable |
| 3292 | Eu33.00 | [X]Recurrent depressive disorder | Probable |
| 8851 | Eu33.11 | [X]Recurrent episodes of depressive reaction | Probable |
| 19696 | Eu33.12 | [X]Recurrent episodes of psychogenic depression | Probable |
| 8902 | Eu33.13 | [X]Recurrent episodes of reactive depression | Probable |
| 28756 | Eu33.14 | [X]Seasonal depressive disorder | Probable |
| 8826 | Eu33.15 | [X]SAD - Seasonal affective disorder | Probable |
| 29784 | Eu33000 | [X]Recurrent depressive disorder, current episode mild | Probable |
| 29520 | Eu33100 | [X]Recurrent depressive disorder, current episode moderate | Probable |
| 33469 | Eu33200 | [X]Recurr depress disorder cur epi severe without psyc sympt | Probable |
| 11329 | Eu33211 | [X]Endogenous depression without psychotic symptoms | Probable |
| 11252 | Eu33212 | [X]Major depression, recurrent without psychotic symptoms | Probable |
| 73991 | Eu33214 | [X]Vital depression, recurrent without psychotic symptoms | Probable |
| 47009 | Eu33300 | [X]Recurrent depress disorder cur epi severe with psyc symp | Probable |
| 23731 | Eu33311 | [X]Endogenous depression with psychotic symptoms | Probable |
| 32941 | Eu33313 | [X]Recurr severe episodes/major depression+psychotic symptom | Probable |
| 16861 | Eu33315 | [X]Recurrent severe episodes of psychotic depression | Probable |
| 22116 | Eu33400 | [X]Recurrent depressive disorder, currently in remission | Probable |
| 47731 | Eu33y00 | [X]Other recurrent depressive disorders | Probable |
| 44300 | Eu33z00 | [X]Recurrent depressive disorder, unspecified | Probable |
| 36616 | Eu33z11 | [X]Monopolar depression NOS | Probable |
| 7953 | Eu34100 | [X]Dysthymia | Probable |
| 8584 | Eu34111 | [X]Depressive neurosis | Probable |
| 7737 | Eu34113 | [X]Neurotic depression | Probable |
| 15220 | Eu34114 | [X]Persistant anxiety depression | Probable |
| 50998 | Eu3y000 | [X]Other single mood affective disorders | Probable |
| 19054 | Eu3y111 | [X]Recurrent brief depressive episodes | Probable |
| 11913 | Eu41200 | [X]Mixed anxiety and depressive disorder | Probable |
| 7749 | Eu41211 | [X]Mild anxiety depression | Probable |
| 29527 | R007z13 | [D]Postoperative depression | Probable |
| 55288 | ZRLfH00 | Health of the Nation Outcome Scale item 7 - depressed mood | Probable |
| 12303 | 13HL.14 | Social withdrawal | Possible |
| 26195 | 14OK.00 | Risk of self neglect | Possible |
| 57949 | 14OM.00 | Moderate risk of self neglect | Possible |
| 57950 | 14ON.00 | High risk of self neglect | Possible |
| 5751 | 1683 | Tired all the time | Possible |
| 2893 | 1685 | Tearful | Possible |
| 101422 | 16ZB100 | Feeling low or worried | Possible |
| 6142 | 1B15.11 | Irritable - symptom | Possible |
| 2930 | 1B17.12 | C/O - feeling unhappy | Possible |
| 22264 | 1B1I100 | C/O tearfulness | Possible |
| 1449 | 1B1J.00 | Emotional problem | Possible |
| 3502 | 1B1J.11 | Emotional upset | Possible |
| 6212 | 1B1P.00 | Crying | Possible |
| 6021 | 1BO..00 | Mood swings | Possible |
| 30740 | 1BP..00 | Loss of interest | Possible |
| 59869 | 1BP0.00 | Loss of interest in previously enjoyable activity | Possible |
| 25435 | 1BQ..00 | Loss of capacity for enjoyment | Possible |
| 42988 | 1BR0.00 | Reduced concentration span | Possible |
| 53148 | 1BU..00 | Loss of hope for the future | Possible |
| 100977 | 1JJ..00 | Suspected depression | Possible |
| 48349 | 1S40.00 | Dysphoric mood | Possible |
| 106647 | 3880100 | Visual analogue mood scale | Possible |
| 19630 | 388J.00 | Hospital anxiety and depression scale | Possible |
| 18315 | 388K.00 | Geriatric depression scale | Possible |
| 19409 | 388P.00 | HAD scale: depression score | Possible |
| 9970 | 388Z.00 | Depression anxiety stress scales depression score | Possible |
| 41066 | 388a.00 | Depression anxiety stress scales stress score | Possible |
| 19163 | 388b.00 | Depression anxiety stress scales anxiety score | Possible |
| 13583 | 388f.00 | Patient health questionnaire (PHQ-9) score | Possible |
| 26817 | 388g.00 | Beck depression inventory second edition score | Possible |
| 91400 | 388l.00 | BASDEC - Brief Assessment Schedule Depression Cards score | Possible |
| 101250 | 38Dp.00 | HAMD - Hamilton rating scale for depression | Possible |
| 109588 | 38Dp.11 | HRSD - Hamilton rating scale for depression | Possible |
| 101423 | 38Dq.00 | MADRS - Montgomery-Asberg depression rating scale | Possible |
| 108928 | 38GJ000 | EuroQol five dimension five level anxiety depression score | Possible |
| 108741 | 6658000 | Antidepressant drug treatment changed | Possible |
| 102632 | 6659000 | Antidepressant drug treatment started | Possible |
| 4740 | 6891 | Depression screen | Possible |
| 103903 | 6891000 | Assessment using Whooley depression screen | Possible |
| 12450 | 6896 | Depression screening using questions | Possible |
| 83565 | 8IA1.00 | Patient health questionnaire (PHQ-9) declined | Possible |
| 110350 | 8IE5100 | Hospital Anxiety and Depression Scale declined | Possible |
| 109182 | 8IH3100 | Depression screening declined | Possible |
| 112682 | 8IH5200 | Referral for guided self-help for depression declined | Possible |
| 25832 | 8O82.00 | Emotional and psychosocial support and advice | Possible |
| 58610 | 9ON..11 | Stress clinic administration | Possible |
| 63249 | 9ON1.00 | Attends stress monitoring | Possible |
| 35648 | 9ON4.00 | Stress monitoring 1st letter | Possible |
| 106542 | 9ON6.00 | Stress monitoring 3rd letter | Possible |
| 96503 | 9ON8.00 | Stress monitoring phone invite | Possible |
| 37248 | 9ONA.00 | Stress monitoring check done | Possible |
| 44674 | E002.00 | Senile dementia with depressive or paranoid features | Possible |
| 41992 | E11z.00 | Other and unspecified affective psychoses | Possible |
| 15551 | E282.00 | Acute stupor state due to acute stress reaction | Possible |
| 38640 | E283.00 | Other acute stress reactions | Possible |
| 29707 | E283z00 | Other acute stress reaction NOS | Possible |
| 23869 | E284.00 | Stress reaction causing mixed disturbance of emotion/conduct | Possible |
| 24212 | E292.00 | Adjustment reaction, predominant disturbance other emotions | Possible |
| 48588 | E292y00 | Adjustment reaction with mixed disturbance of emotion | Possible |
| 15665 | E292z00 | Adjustment reaction with disturbance of other emotion NOS | Possible |
| 45603 | E294.00 | Adjustment reaction with disturbance emotion and conduct | Possible |
| 19921 | E29y500 | Other adjustment reaction with withdrawal | Possible |
| 41599 | E2C4.00 | Mixed disturbance of conduct and emotion | Possible |
| 6121 | E2C4z00 | Mixed disturbance of conduct and emotion NOS | Possible |
| 24000 | Eu05300 | [X]Organic mood [affective] disorders | Possible |
| 5726 | Eu3..00 | [X]Mood - affective disorders | Possible |
| 31757 | Eu33314 | [X]Recurr severe episodes/psychogenic depressive psychosis | Possible |
| 37764 | Eu33316 | [X]Recurrent severe episodes/reactive depressive psychosis | Possible |
| 42857 | Eu34.00 | [X]Persistent mood affective disorders | Possible |
| 50243 | Eu34y00 | [X]Other persistent mood affective disorders | Possible |
| 39767 | Eu34z00 | [X]Persistent mood affective disorder, unspecified | Possible |
| 28008 | Eu3y.00 | [X]Other mood affective disorders | Possible |
| 30688 | Eu3y011 | [X]Mixed affective episode | Possible |
| 29921 | Eu3y100 | [X]Other recurrent mood affective disorders | Possible |
| 29579 | Eu3yy00 | [X]Other specified mood affective disorders | Possible |
| 37090 | Eu3z.00 | [X]Unspecified mood affective disorder | Possible |
| 21753 | Eu43y00 | [X]Other reactions to severe stress | Possible |
| 24483 | Eu52111 | [X]Anhedonia sexual | Possible |
| 91580 | Eu92.11 | [X]Emotional behavioural problems | Possible |
| 22687 | R00z600 | [D]Unhappiness | Possible |
| 2777 | R2y3.11 | [D] Self neglect | Possible |
| 27665 | Ry15.00 | [D]Undue concern and preoccupation with stressful events | Possible |
| 7230 | Ry18.00 | [D]Self neglect | Possible |
| 56020 | Ryu5800 | [X]State of emotional shock and stress, unspecified | Possible |
| 7569 | TJ90.00 | Adverse reaction to antidepressants | Possible |
| 39808 | TJ90z00 | Adverse reaction to antidepressants NOS | Possible |
| 33618 | U609000 | [X]Tricyc/tetracyc antidepres caus advers eff therapeut use | Possible |
| 31557 | U609200 | [X]Other unspec antidepres caus advers effect in therap use | Possible |
| 46796 | U609211 | [X] Adverse reaction to antidepressant | Possible |
| 55631 | U609213 | [X] Adverse reaction to antidepressants NOS | Possible |
| 32593 | Z787.00 | Self-neglect | Possible |
| 32226 | Z787400 | Neglect of personal hygiene | Possible |
| 22885 | Z7C5111 | Lack of concentration | Possible |
| 38194 | Z7C5300 | Reduced concentration span | Possible |
| 26224 | ZR2A.00 | Beck depression inventory | Possible |
| 26080 | ZR2A.11 | BDI - Beck depression inventory | Possible |
| 42839 | ZR2B.00 | Beck hopelessness scale | Possible |
| 28442 | ZR2G.00 | Behaviour and mood disturbance scale | Possible |
| 38058 | ZR2h.00 | Brief depression rating scale | Possible |
| 52655 | ZR7..00 | Depression anxiety scale | Possible |
| 54999 | ZR8..00 | Depression self rating scale | Possible |
| 56130 | ZR8..11 | DSRS - Depression self rating scale | Possible |
| 26374 | ZRL6.00 | Geriatric depression scale | Possible |
| 35320 | ZRL6.11 | GDS - Geriatric depression scale | Possible |
| 39131 | ZRL6.12 | Geriatric depression score | Possible |
| 42975 | ZRLU.00 | Hamilton rating scale for depression | Possible |
| 42836 | ZRLU.11 | HAMD - Hamilton rating scale for depression | Possible |
| 44927 | ZRLU.12 | HRSD - Hamilton rating scale for depression | Possible |
| 96038 | ZRLfI00 | Health of the Nation Outcome Scale item 7 - depressed mood | Possible |
| 59794 | ZRLn.00 | Hopelessness scale | Possible |
| 19245 | ZRLr.00 | Hospital anxiety and depression scale | Possible |
| 35398 | ZRLr.11 | HAD - Hospital anxiety and depression scale | Possible |
| 24820 | ZRLr.12 | HADS - Hospital anxiety and depression scale | Possible |
| 56034 | ZRVM.00 | Leeds scale for the self-assessment of anxiety & depression | Possible |
| 34275 | ZRaH.00 | Mood affective checklist | Possible |
| 64584 | ZRaH.11 | MACL - Mood affective checklist | Possible |
| 94518 | ZRbS.00 | Positive and negative affect schedule | Possible |
| 44287 | ZRby.00 | Profile of mood states | Possible |
| 100194 | ZRby.11 | POMS - Profile of mood states | Possible |
| 102465 | ZRrI.00 | Wakefield self-assessment depression inventory | Possible |
| 89707 | ZRrY.00 | WHO depression scale | Possible |
| 56982 | ZRrc.00 | Zung self-rating depression scale | Possible |
| 37942 | ZRrc.11 | SDS - Zung self-rating depression scale | Possible |
| 4876 | ZV79000 | [V]Screening for depression | Possible |
| 2716 | 1465 | H/O: depression | History of |
| 19439 | 212S.00 | Depression resolved | History of |
| 100179 | 665A000 | Antidepressant drug treatment stopped | History of |
| 44936 | 9HA1.00 | Removed from depression register | History of |
| 43324 | E112500 | Single major depressive episode, partial or unspec remission | History of |
| 57409 | E112600 | Single major depressive episode, in full remission | History of |
| 56273 | E113500 | Recurrent major depressive episodes,partial/unspec remission | History of |
| 55384 | E113600 | Recurrent major depressive episodes, in full remission | History of |
| 15117 | ZV11100 | [V]Personal history of affective disorder | History of |

# S1 Table F Codelist for antidepressants – main analysis.

| prodcode | Product Name |
| --- | --- |
| 22 | Fluoxetine 20mg capsules |
| 49 | Amitriptyline 25mg tablets |
| 50 | Paroxetine 20mg tablets |
| 67 | Citalopram 20mg tablets |
| 74 | Dosulepin 75mg tablets |
| 83 | Amitriptyline 10mg tablets |
| 84 | Dosulepin 25mg capsules |
| 114 | Lofepramine 70mg tablets |
| 252 | Prozac 20mg/5ml liquid (Eli Lilly and Company Ltd) |
| 301 | Venlafaxine 37.5mg tablets |
| 418 | Prozac 20mg capsules (Eli Lilly and Company Ltd) |
| 470 | Venlafaxine 75mg modified-release capsules |
| 476 | Citalopram 10mg tablets |
| 487 | Amitriptyline 25mg modified-release capsules |
| 488 | Sertraline 50mg tablets |
| 513 | Citalopram 40mg/ml oral drops sugar free |
| 527 | Paroxetine 10mg/5ml oral suspension sugar free |
| 595 | Amitriptyline 25mg / Perphenazine 2mg tablets |
| 603 | Escitalopram 10mg tablets |
| 623 | Efexor 37.5mg tablets (Wyeth Pharmaceuticals) |
| 648 | Cipralex 10mg tablets (Lundbeck Ltd) |
| 727 | Sertraline 100mg tablets |
| 742 | Mirtazapine 30mg tablets |
| 785 | Cipralex 5mg tablets (Lundbeck Ltd) |
| 815 | Cipramil 40mg/ml drops (Lundbeck Ltd) |
| 841 | Seroxat 20mg tablets (GlaxoSmithKline UK Ltd) |
| 1169 | Prothiaden 25mg capsules (Teofarma) |
| 1208 | Triptafen tablets (AMCo) |
| 1222 | Venlafaxine 75mg tablets |
| 1310 | Imipramine 10mg tablets |
| 1397 | Paroxetine 30mg tablets |
| 1453 | Triptafen m 2mg+10mg Tablet (Goldshield Pharmaceuticals Ltd) |
| 1474 | Efexor XL 75mg capsules (Pfizer Ltd) |
| 1575 | Seroxat 30mg tablets (GlaxoSmithKline UK Ltd) |
| 1612 | Lustral 50mg tablets (Pfizer Ltd) |
| 1712 | Cipramil 20mg tablets (Lundbeck Ltd) |
| 1730 | Trazodone 100mg capsules |
| 1809 | Imipramine 25mg tablets |
| 1888 | Amitriptyline 50mg tablets |
| 1940 | Dothapax 25 capsules (Ashbourne Pharmaceuticals Ltd) |
| 2039 | Trimipramine 25mg tablets |
| 2093 | Gamanil 70mg tablets (Merck Serono Ltd) |
| 2290 | Fluvoxamine 100mg tablets |
| 2320 | Prothiaden 75mg tablets (Teofarma) |
| 2356 | Reboxetine 4mg tablets |
| 2408 | Cipramil 40mg tablets (Lundbeck Ltd) |
| 2486 | Lentizol 25mg modified-release capsules (Pfizer Ltd) |
| 2525 | Amitriptyline 75mg modified-release capsules |
| 2531 | Surmontil 50mg capsules (Sanofi) |
| 2532 | Surmontil 25mg tablets (Sanofi) |
| 2548 | Fluoxetine 20mg/5ml oral solution |
| 2579 | Tofranil 10mg Tablet (Novartis Pharmaceuticals UK Ltd) |
| 2617 | Venlafaxine 50mg tablets |
| 2654 | Venlafaxine 150mg modified-release capsules |
| 2880 | Fluvoxamine 50mg tablets |
| 2883 | Moclobemide 150mg tablets |
| 2897 | Faverin 50mg tablets (Mylan) |
| 2985 | Lentizol 50mg modified-release capsules (Pfizer Ltd) |
| 3083 | Mianserin 10mg tablets |
| 3183 | Nortriptyline 10mg tablets |
| 3194 | Clomipramine 10mg capsules |
| 3196 | Trimipramine 50mg capsules |
| 3349 | Nardil 15mg tablets (Kyowa Kirin Ltd) |
| 3355 | Trazodone 50mg capsules |
| 3490 | Amitriptyline 10mg / Perphenazine 2mg tablets |
| 3601 | Seroxat 20mg/10ml liquid (GlaxoSmithKline UK Ltd) |
| 3657 | Anafranil 25mg capsules (Novartis Pharmaceuticals UK Ltd) |
| 3670 | Clomipramine 25mg capsules |
| 3777 | Amitriptyline 10mg/5ml sugar free oral solution |
| 3783 | Tranylcypromine 10mg tablets |
| 3861 | Cipramil 10mg tablets (Lundbeck Ltd) |
| 3903 | Nortriptyline 25mg tablets |
| 3925 | Clomipramine 50mg capsules |
| 4003 | Molipaxin 150mg tablets (Zentiva) |
| 4020 | Trazodone 150mg tablets |
| 4075 | Fluoxetine 60mg capsules |
| 4118 | Nortriptyline 10mg Capsule |
| 4194 | Molipaxin 100mg capsules (Zentiva) |
| 4218 | Lofepramine 70mg/5ml oral suspension sugar free |
| 4310 | Trimipramine 10mg tablets |
| 4321 | Phenelzine 15mg tablets |
| 4329 | Mianserin 20mg tablets |
| 4352 | Lustral 100mg tablets (Pfizer Ltd) |
| 4404 | Tofranil 25mg/5ml syrup (Novartis Pharmaceuticals UK Ltd) |
| 4682 | Amitriptyline 50mg modified-release capsules |
| 4690 | Amitriptyline 50mg/5ml oral solution sugar free |
| 4726 | Zispin 30mg tablets (Organon Laboratories Ltd) |
| 4770 | Citalopram 40mg tablets |
| 4874 | Molipaxin 50mg capsules (Zentiva) |
| 4907 | Prozac 60mg capsules (Eli Lilly and Company Ltd) |
| 5187 | Moclobemide 300mg tablets |
| 5710 | Efexor XL 150mg capsules (Pfizer Ltd) |
| 5832 | Manerix 300mg tablets (Meda Pharmaceuticals Ltd) |
| 6054 | Dosulepin 25mg/5ml oral solution sugar free |
| 6218 | Escitalopram 20mg tablets |
| 6255 | Mianserin 30mg tablets |
| 6274 | Efexor 50mg tablets (Wyeth Pharmaceuticals) |
| 6312 | Amitriptyline 25mg/5ml oral solution sugar free |
| 6360 | Cipralex 20mg tablets (Lundbeck Ltd) |
| 6405 | Escitalopram 5mg tablets |
| 6421 | Mirtazapine 15mg orodispersible tablets |
| 6442 | Trazodone 50mg/5ml oral solution sugar free |
| 6481 | Mirtazapine 45mg orodispersible tablets |
| 6488 | Mirtazapine 30mg orodispersible tablets |
| 6795 | Mirtazapine 15mg tablets |
| 6846 | Zispin SolTab 15mg orodispersible tablets (Merck Sharp & Dohme Ltd) |
| 6854 | Mirtazapine 45mg tablets |
| 6894 | Perphenazine 2mg with Amitriptyline 25mg tablet |
| 6895 | Duloxetine 60mg gastro-resistant capsules |
| 7122 | Duloxetine 30mg gastro-resistant capsules |
| 7147 | Duloxetine 40mg gastro-resistant capsules |
| 7153 | Duloxetine 20mg gastro-resistant capsules |
| 7328 | Sertraline 50mg/5ml oral suspension |
| 7468 | Bolvidon 10mg Tablet (Organon Laboratories Ltd) |
| 7515 | Anafranil 10mg capsules (Novartis Pharmaceuticals UK Ltd) |
| 7677 | Allegron 10mg tablets (King Pharmaceuticals Ltd) |
| 7678 | Nortriptyline 25mg Capsule |
| 7693 | Anafranil 50mg capsules (Novartis Pharmaceuticals UK Ltd) |
| 7751 | Tryptizol 25mg Tablet (Merck Sharp & Dohme Ltd) |
| 7894 | Anafranil SR 75mg tablets (Novartis Pharmaceuticals UK Ltd) |
| 7910 | Tofranil 25mg tablets (Novartis Pharmaceuticals UK Ltd) |
| 8055 | Imipramine 25mg/5ml oral solution |
| 8144 | Bolvidon 20mg Tablet (Organon Laboratories Ltd) |
| 8174 | Molipaxin 50mg/5ml oral liquid (Sanofi) |
| 8332 | Tryptizol 50mg Tablet (Merck Sharp & Dohme Ltd) |
| 8585 | Bolvidon 30mg Tablet (Organon Laboratories Ltd) |
| 8640 | Allegron 25mg tablets (King Pharmaceuticals Ltd) |
| 8661 | Clomipramine 75mg modified-release tablets |
| 8719 | Anafranil 25mg/5ml syrup (Novartis Pharmaceuticals UK Ltd) |
| 8720 | Clomipramine 25mg/5ml oral solution |
| 8726 | Tryptizol 10mg Tablet (Merck Sharp & Dohme Ltd) |
| 8831 | Tryptizol mr 75mg Modified-release capsule (Merck Sharp & Dohme Ltd) |
| 8878 | Tryptizol 10mg/5ml sugar free Oral solution (Merck Sharp and Dohme Ltd) |
| 8928 | Surmontil 10mg tablets (Sanofi) |
| 9182 | Efexor 75mg tablets (Wyeth Pharmaceuticals) |
| 9206 | Manerix 150mg tablets (Meda Pharmaceuticals Ltd) |
| 10083 | Zispin SolTab 30mg orodispersible tablets (Merck Sharp & Dohme Ltd) |
| 10787 | Parnate 10mg Tablet (Goldshield Pharmaceuticals Ltd) |
| 10948 | Dosulepin 75mg/5ml oral solution sugar free |
| 11956 | Norval 20mg Tablet (Bencard) |
| 12123 | Faverin 100mg tablets (Mylan) |
| 12192 | Norval 30mg Tablet (Bencard) |
| 12207 | Isocarboxazid 10mg tablets |
| 12353 | Aventyl 25mg Capsule (Eli Lilly and Company Ltd) |
| 12368 | Norval 10mg Tablet (Bencard) |
| 12503 | Marplan 10mg Tablet (Cambridge Laboratories Ltd) |
| 12549 | Aventyl 10mg/5ml Liquid (Eli Lilly and Company Ltd) |
| 12710 | Trazodone 150mg modified-release tablets |
| 13151 | Cymbalta 30mg gastro-resistant capsules (Eli Lilly and Company Ltd) |
| 13237 | Venlafaxine 37.5mg/5ml oral suspension |
| 13621 | Molipaxin CR 150mg tablets (Aventis Pharma) |
| 14740 | Oxactin 20mg capsules (Discovery Pharmaceuticals) |
| 14803 | Yentreve 40mg gastro-resistant capsules (Eli Lilly and Company Ltd) |
| 14849 | Cymbalta 60mg gastro-resistant capsules (Eli Lilly and Company Ltd) |
| 15163 | Edronax 4mg tablets (Pfizer Ltd) |
| 15268 | Zispin SolTab 45mg orodispersible tablets (Merck Sharp & Dohme Ltd) |
| 15632 | Dothapax 75 tablets (Ashbourne Pharmaceuticals Ltd) |
| 16154 | Mirtazapine 15mg/ml oral solution sugar free |
| 16323 | Perphenazine 2mg with Amitriptyline 10mg tablet |
| 16969 | Yentreve 20mg gastro-resistant capsules (Eli Lilly and Company Ltd) |
| 17183 | Aventyl 10mg Capsule (Eli Lilly and Company Ltd) |
| 19168 | Dosulepin 25mg/5ml mixture |
| 19181 | Trazodone 100mg capsules (Mylan) |
| 19183 | Fluoxetine 20mg capsules (A A H Pharmaceuticals Ltd) |
| 19186 | Dosulepin 75mg tablets (Actavis UK Ltd) |
| 19470 | Fluoxetine 20mg capsules (Ranbaxy (UK) Ltd) |
| 20026 | Domical 25mg Tablet (Berk Pharmaceuticals Ltd) |
| 20152 | Escitalopram 10mg/ml oral drops sugar free |
| 21157 | Thaden 75mg tablets (Opus Pharmaceuticals Ltd) |
| 21819 | Prepadine 75mg tablets (Teva UK Ltd) |
| 21820 | Prepadine 25mg capsules (Teva UK Ltd) |
| 22070 | Amitriptyline 10mg/5ml Oral solution (Rosemont Pharmaceuticals Ltd) |
| 23426 | Dosulepin 25mg capsules (A A H Pharmaceuticals Ltd) |
| 24134 | Amitriptyline 25mg tablets (Kent Pharmaceuticals Ltd) |
| 24141 | Amitriptyline 10mg tablets (Actavis UK Ltd) |
| 24145 | Amitriptyline 25mg tablets (Actavis UK Ltd) |
| 24147 | Amitriptyline 25mg tablets (Teva UK Ltd) |
| 24152 | Amitriptyline 10mg tablets (Teva UK Ltd) |
| 24680 | Elavil 10mg Tablet (DDSA Pharmaceuticals Ltd) |
| 25444 | Lomont 70mg/5ml oral suspension (Rosemont Pharmaceuticals Ltd) |
| 26016 | Citalopram 20mg tablets (Sandoz Ltd) |
| 26056 | Cipralex 10mg/ml oral drops (Lundbeck Ltd) |
| 26213 | Domical 10mg Tablet (Berk Pharmaceuticals Ltd) |
| 27008 | Domical 50mg Tablet (Berk Pharmaceuticals Ltd) |
| 29339 | Trazodone 50mg capsules (Mylan) |
| 29756 | Paxoran 20mg Tablet (Ranbaxy (UK) Ltd) |
| 29786 | Ranflutin 20mg capsules (Ranbaxy (UK) Ltd) |
| 29857 | Trazodone 150mg tablets (Teva UK Ltd) |
| 29875 | Dosulepin 25mg capsules (Mylan) |
| 30258 | Fluoxetine 20mg/5ml oral solution (Teva UK Ltd) |
| 30376 | Thaden 25mg capsules (Opus Pharmaceuticals Ltd) |
| 30983 | Trazodone 150mg tablets (Mylan) |
| 31824 | Dosulepin 25mg capsules (IVAX Pharmaceuticals UK Ltd) |
| 31826 | Dosulepin 75mg tablets (IVAX Pharmaceuticals UK Ltd) |
| 32121 | Dosulepin 75mg tablets (A A H Pharmaceuticals Ltd) |
| 32401 | Sertraline 50mg tablets (A A H Pharmaceuticals Ltd) |
| 32439 | Amitriptyline 25mg Tablet (Sussex Pharmaceutical Ltd) |
| 32546 | Paxoran 10mg Tablet (Ranbaxy (UK) Ltd) |
| 32848 | Citalopram 10mg tablets (Actavis UK Ltd) |
| 32863 | Imipramine 10mg tablets (Teva UK Ltd) |
| 32899 | Paroxetine 20mg tablets (Actavis UK Ltd) |
| 33071 | Felicium 20mg capsules (Opus Pharmaceuticals Ltd) |
| 33074 | Praminil 10mg Tablet (DDSA Pharmaceuticals Ltd) |
| 33090 | Amitriptyline 10mg tablets (A A H Pharmaceuticals Ltd) |
| 33164 | Dosulepin 25mg capsules (Sandoz Ltd) |
| 33337 | Mirtazapine 45mg tablets (A A H Pharmaceuticals Ltd) |
| 33410 | Fluoxetine 20mg capsules (Zentiva) |
| 33624 | Amitriptyline 50mg tablets (Teva UK Ltd) |
| 33720 | Citalopram 10mg tablets (IVAX Pharmaceuticals UK Ltd) |
| 33779 | Prozit 20mg/5ml oral solution (Pinewood Healthcare) |
| 33978 | Paroxetine 20mg tablets (Mylan) |
| 34003 | Trazodone 50mg capsules (A A H Pharmaceuticals Ltd) |
| 34046 | Lofepramine 70mg tablets (A A H Pharmaceuticals Ltd) |
| 34058 | Dosulepin 75mg tablets (Teva UK Ltd) |
| 34107 | Amitriptyline 50mg tablets (Wockhardt UK Ltd) |
| 34129 | Amitriptyline 25mg tablets (Wockhardt UK Ltd) |
| 34182 | Amitriptyline 50mg tablets (Kent Pharmaceuticals Ltd) |
| 34197 | Amitriptyline 25mg Tablet (Berk Pharmaceuticals Ltd) |
| 34202 | Fluoxetine 20mg capsules (Genus Pharmaceuticals Ltd) |
| 34216 | Fluoxetine 20mg/5ml oral solution (A A H Pharmaceuticals Ltd) |
| 34222 | Imipramine 10mg tablets (Actavis UK Ltd) |
| 34223 | Dosulepin 25mg capsules (Teva UK Ltd) |
| 34224 | Amitriptyline 25mg/5ml oral solution sugar free (Rosemont Pharmaceuticals Ltd) |
| 34245 | Clomipramine 25mg capsules (A A H Pharmaceuticals Ltd) |
| 34251 | Amitriptyline 50mg/5ml oral solution sugar free (Rosemont Pharmaceuticals Ltd) |
| 34274 | Amitriptyline 50mg tablets (A A H Pharmaceuticals Ltd) |
| 34288 | Fluoxetine 20mg capsules (Mylan) |
| 34294 | Fluoxetine 20mg capsules (IVAX Pharmaceuticals UK Ltd) |
| 34351 | Paroxetine 20mg tablets (IVAX Pharmaceuticals UK Ltd) |
| 34355 | Imipramine 25mg tablets (Actavis UK Ltd) |
| 34356 | Citalopram 20mg tablets (A A H Pharmaceuticals Ltd) |
| 34401 | Amitriptyline 10mg tablets (Wockhardt UK Ltd) |
| 34413 | Citalopram 10mg tablets (Zentiva) |
| 34415 | Citalopram 20mg tablets (Mylan) |
| 34419 | Paroxetine 20mg tablets (A A H Pharmaceuticals Ltd) |
| 34421 | Trazodone 50mg capsules (Zentiva) |
| 34436 | Citalopram 10mg tablets (Mylan) |
| 34456 | Fluoxetine 20mg capsules (Teva UK Ltd) |
| 34466 | Citalopram 40mg tablets (Sandoz Ltd) |
| 34470 | Trazodone 150mg tablets (Zentiva) |
| 34474 | Amitriptyline 25mg Tablet (Regent Laboratories Ltd) |
| 34498 | Citalopram 10mg Tablet (Neo Laboratories Ltd) |
| 34499 | Citalopram 10mg tablets (Sandoz Ltd) |
| 34503 | Amitriptyline 25mg tablets (IVAX Pharmaceuticals UK Ltd) |
| 34525 | Dosulepin 75mg tablets (Mylan) |
| 34578 | Lofepramine 70mg tablets (IVAX Pharmaceuticals UK Ltd) |
| 34580 | Trazodone 100mg capsules (A A H Pharmaceuticals Ltd) |
| 34586 | Citalopram 10mg tablets (A A H Pharmaceuticals Ltd) |
| 34587 | Paroxetine 30mg tablets (A A H Pharmaceuticals Ltd) |
| 34603 | Citalopram 40mg tablets (Mylan) |
| 34634 | Amitriptyline 50mg tablets (Actavis UK Ltd) |
| 34641 | Dosulepin 25mg capsules (Sovereign Medical Ltd) |
| 34643 | Dosulepin 25mg capsules (Almus Pharmaceuticals Ltd) |
| 34672 | Lofepramine 70mg tablets (Sterwin Medicines) |
| 34722 | Citalopram 20mg Tablet (Neo Laboratories Ltd) |
| 34731 | Amitriptyline 10mg tablets (Kent Pharmaceuticals Ltd) |
| 34745 | Dosulepin 25mg capsules (Actavis UK Ltd) |
| 34782 | Amitriptyline 25mg tablets (A A H Pharmaceuticals Ltd) |
| 34813 | Imipramine 25mg tablets (A A H Pharmaceuticals Ltd) |
| 34822 | Citalopram 20mg tablets (Zentiva) |
| 34849 | Fluoxetine 20mg capsules (Tillomed Laboratories Ltd) |
| 34856 | Fluoxetine 60mg capsules (Mylan) |
| 34866 | Clomipramine 10mg capsules (A A H Pharmaceuticals Ltd) |
| 34871 | Citalopram 20mg tablets (Actavis UK Ltd) |
| 34872 | Imipramine 25mg Tablet (C P Pharmaceuticals Ltd) |
| 34916 | Amitriptyline 10mg Tablet (Berk Pharmaceuticals Ltd) |
| 34950 | Lofepramine 70mg tablets (Accord Healthcare Ltd) |
| 34966 | Citalopram 20mg tablets (Teva UK Ltd) |
| 34970 | Citalopram 20mg tablets (Niche Generics Ltd) |
| 35021 | Paroxetine 10mg tablets |
| 35112 | Seroxat 10mg tablets (GlaxoSmithKline UK Ltd) |
| 36746 | Citalopram 40mg tablets (A A H Pharmaceuticals Ltd) |
| 36893 | Fluoxetine 20mg/5ml oral solution sugar free |
| 37256 | Prozep 20mg/5ml oral solution (Chemidex Pharma Ltd) |
| 38274 | Clomipramine 50mg/5ml oral suspension |
| 38827 | Triptafen-M tablets (Mercury Pharma Group Ltd) |
| 38890 | Fluoxetine 20mg Capsule (Milpharm Ltd) |
| 39145 | Nortriptyline 10mg/5ml Liquid |
| 39359 | Venlafaxine 75mg modified-release tablets |
| 39360 | Venlafaxine 150mg modified-release tablets |
| 39770 | Tifaxin XL 75mg capsules (Genus Pharmaceuticals Ltd) |
| 39809 | Tifaxin XL 150mg capsules (Genus Pharmaceuticals Ltd) |
| 40048 | ViePax XL 75mg tablets (Dexcel-Pharma Ltd) |
| 40049 | ViePax XL 150mg tablets (Dexcel-Pharma Ltd) |
| 40054 | Venlafaxine 225mg modified-release tablets |
| 40059 | Venlalic XL 75mg tablets (Ethypharm UK Ltd) |
| 40062 | Venlalic XL 150mg tablets (Ethypharm UK Ltd) |
| 40092 | Vensir XL 150mg capsules (Morningside Healthcare Ltd) |
| 40160 | Mirtazapine 30mg tablets (Accord Healthcare Ltd) |
| 40165 | Paroxetine 30mg tablets (Actavis UK Ltd) |
| 40277 | Vensir XL 75mg capsules (Morningside Healthcare Ltd) |
| 40295 | Valdoxan 25mg tablets (Servier Laboratories Ltd) |
| 40396 | Amitriptyline 50mg Tablet (Berk Pharmaceuticals Ltd) |
| 40407 | Venlalic XL 225mg tablets (Ethypharm UK Ltd) |
| 40494 | Agomelatine 25mg tablets |
| 40514 | Venaxx XL 150mg capsules (AMCo) |
| 40515 | Venaxx XL 75mg capsules (AMCo) |
| 40517 | Vexarin XL 150mg capsules (Mylan) |
| 40726 | Escitalopram 20mg/ml oral drops sugar free |
| 40764 | ViePax 37.5mg tablets (Dexcel-Pharma Ltd) |
| 40815 | Tardcaps XL 75mg capsules (IXL Pharma Ltd) |
| 40817 | Tardcaps XL 150mg capsules (IXL Pharma Ltd) |
| 40892 | Paroxetine 20mg tablets (Genus Pharmaceuticals Ltd) |
| 40917 | ViePax 75mg tablets (Dexcel-Pharma Ltd) |
| 41033 | Rodomel XL 75mg capsules (Teva UK Ltd) |
| 41062 | Cipralex 20mg/ml oral drops (Lundbeck Ltd) |
| 41299 | Politid XL 75mg capsules (Actavis UK Ltd) |
| 41314 | Rodomel XL 150mg capsules (Teva UK Ltd) |
| 41408 | Imipramine 25mg tablets (Teva UK Ltd) |
| 41528 | Citalopram 10mg tablets (Teva UK Ltd) |
| 41563 | Clomipramine 25mg capsules (IVAX Pharmaceuticals UK Ltd) |
| 41597 | Clomipramine 50mg capsules (IVAX Pharmaceuticals UK Ltd) |
| 41609 | Trazodone 50mg capsules (Teva UK Ltd) |
| 41627 | Lofepramine 70mg Tablet (Teva UK Ltd) |
| 41628 | Clomipramine 10mg capsules (IVAX Pharmaceuticals UK Ltd) |
| 41654 | Tranylcypromine 10mg tablets (AMCo) |
| 41681 | Imipramine 10mg tablets (A A H Pharmaceuticals Ltd) |
| 41709 | Trazodone 100mg capsules (Teva UK Ltd) |
| 41710 | Trazodone 100mg capsules (Zentiva) |
| 41729 | Amitriptyline 25mg Tablet (Celltech Pharma Europe Ltd) |
| 41731 | Isocarboxazid 10mg Tablet (Cambridge Laboratories Ltd) |
| 41747 | Moclobemide 150mg tablets (Teva UK Ltd) |
| 42078 | Amitriptyline 25mg tablets (Almus Pharmaceuticals Ltd) |
| 42107 | Fluoxetine 20mg capsules (Niche Generics Ltd) |
| 42228 | Trimipramine 10mg tablets (A A H Pharmaceuticals Ltd) |
| 42247 | Imipramine 25mg/5ml oral solution sugar free |
| 42387 | Sertraline 50mg tablets (Actavis UK Ltd) |
| 42394 | Amitriptyline 25mg Tablet (Crosspharma Ltd) |
| 42499 | Fluoxetine 10mg tablets |
| 42600 | Vexarin XL 75mg capsules (Mylan) |
| 42660 | Citalopram 10mg tablets (Almus Pharmaceuticals Ltd) |
| 42734 | Dosulepin 75mg tablets (Almus Pharmaceuticals Ltd) |
| 42803 | Fluoxetine 20mg/5ml oral solution (IVAX Pharmaceuticals UK Ltd) |
| 43024 | Dosulepin 100mg/5ml oral solution |
| 43203 | Venlafaxine 75mg modified-release capsules (Sandoz Ltd) |
| 43234 | Mirtazapine 45mg orodispersible tablets (Teva UK Ltd) |
| 43235 | Mirtazapine 45mg orodispersible tablets (A A H Pharmaceuticals Ltd) |
| 43236 | Mirtazapine 45mg orodispersible tablets (Accord Healthcare Ltd) |
| 43237 | Mirtazapine 15mg orodispersible tablets (Teva UK Ltd) |
| 43239 | Mirtazapine 15mg tablets (A A H Pharmaceuticals Ltd) |
| 43241 | Mirtazapine 15mg orodispersible tablets (Aurobindo Pharma Ltd) |
| 43242 | Mirtazapine 15mg tablets (Genus Pharmaceuticals Ltd) |
| 43246 | Mirtazapine 15mg orodispersible tablets (Genus Pharmaceuticals Ltd) |
| 43247 | Mirtazapine 45mg orodispersible tablets (Genus Pharmaceuticals Ltd) |
| 43248 | Mirtazapine 15mg orodispersible tablets (Focus Pharmaceuticals Ltd) |
| 43250 | Mirtazapine 30mg orodispersible tablets (A A H Pharmaceuticals Ltd) |
| 43253 | Mirtazapine 15mg orodispersible tablets (A A H Pharmaceuticals Ltd) |
| 43256 | Mirtazapine 45mg orodispersible tablets (Focus Pharmaceuticals Ltd) |
| 43257 | Mirtazapine 15mg tablets (Teva UK Ltd) |
| 43334 | Venlafaxine 150mg modified-release capsules (Sandoz Ltd) |
| 43518 | Fluvoxamine 100mg tablets (IVAX Pharmaceuticals UK Ltd) |
| 43519 | Citalopram 40mg Tablet (Neo Laboratories Ltd) |
| 43534 | Lofepramine 70mg/5ml Oral suspension (Rosemont Pharmaceuticals Ltd) |
| 43561 | Clomipramine 10mg capsules (Teva UK Ltd) |
| 43673 | Politid XL 150mg capsules (Actavis UK Ltd) |
| 43968 | Foraven XL 75mg capsules (Forum Products Ltd) |
| 44853 | Dosulepin 25mg capsules (Kent Pharmaceuticals Ltd) |
| 44861 | Fluvoxamine 100mg tablets (Actavis UK Ltd) |
| 44936 | Venlaneo XL 150mg capsules (Kent Pharmaceuticals Ltd) |
| 44937 | Venlaneo XL 75mg capsules (Kent Pharmaceuticals Ltd) |
| 44944 | Sertraline 100mg tablets (Teva UK Ltd) |
| 45223 | Citalopram 40mg tablets (Niche Generics Ltd) |
| 45224 | Fluoxetine 20mg capsules (Sandoz Ltd) |
| 45226 | Trimipramine 25mg tablets (A A H Pharmaceuticals Ltd) |
| 45233 | Amitriptyline 10mg tablets (IVAX Pharmaceuticals UK Ltd) |
| 45242 | Amitriptyline 10mg Tablet (Sussex Pharmaceutical Ltd) |
| 45247 | Fluoxetine 20mg capsules (Fannin UK Ltd) |
| 45286 | Citalopram 10mg tablets (Niche Generics Ltd) |
| 45304 | Citalopram 40mg tablets (Teva UK Ltd) |
| 45316 | Fluoxetine 20mg capsules (Wockhardt UK Ltd) |
| 45318 | Clomipramine 50mg capsules (A A H Pharmaceuticals Ltd) |
| 45329 | Fluoxetine 20mg capsules (Actavis UK Ltd) |
| 45350 | Clomipramine 25mg capsules (Teva UK Ltd) |
| 45664 | Depefex XL 150mg capsules (Chiesi Ltd) |
| 45737 | Dosulepin 25mg/5ml Oral solution (Rosemont Pharmaceuticals Ltd) |
| 45806 | Venlafaxine 37.5mg modified-release tablets |
| 45818 | Venlalic XL 37.5mg tablets (Ethypharm UK Ltd) |
| 45915 | Sertraline 50mg tablets (Almus Pharmaceuticals Ltd) |
| 45959 | Depefex XL 75mg capsules (Chiesi Ltd) |
| 46668 | Mirtazapine 15mg tablets (Arrow Generics Ltd) |
| 46801 | Amitriptyline 10mg/5ml oral solution |
| 46818 | Amitriptyline 10mg/5ml oral suspension |
| 46926 | Citalopram 40mg tablets (Zentiva) |
| 46970 | Amitriptyline 50mg tablets (IVAX Pharmaceuticals UK Ltd) |
| 46977 | Citalopram 40mg tablets (Actavis UK Ltd) |
| 47363 | Mianserin 20mg Tablet (Berk Pharmaceuticals Ltd) |
| 47945 | Mirtazapine 30mg tablets (A A H Pharmaceuticals Ltd) |
| 47966 | Mirtazapine 15mg/ml oral solution sugar free (Rosemont Pharmaceuticals Ltd) |
| 48026 | Citalopram 20mg tablets (Almus Pharmaceuticals Ltd) |
| 48045 | Fluvoxamine 100mg tablets (A A H Pharmaceuticals Ltd) |
| 48065 | Amitriptyline oral solution |
| 48185 | Mirtazapine 30mg orodispersible tablets (Almus Pharmaceuticals Ltd) |
| 48199 | Ranfaxine XL 75mg capsules (Ranbaxy (UK) Ltd) |
| 48216 | Nortriptyline 25mg tablets (A A H Pharmaceuticals Ltd) |
| 48220 | Prozac 20mg capsules (Lexon (UK) Ltd) |
| 48698 | Mirtazapine 15mg orodispersible tablets sugar free |
| 49165 | Citalopram 10mg tablets (Alliance Healthcare (Distribution) Ltd) |
| 49511 | Venlablue XL 75mg capsules (Creo Pharma Ltd) |
| 49519 | Sertraline 100mg/5ml oral suspension |
| 49820 | Mirtazapine 45mg orodispersible tablets sugar free |
| 50081 | Venlablue XL 150mg capsules (Creo Pharma Ltd) |
| 50722 | Dosulepin 25mg/5ml oral solution |
| 50892 | Zispin SolTab 15mg orodispersible tablets (Necessity Supplies Ltd) |
| 50934 | Venlafaxine 150mg/5ml oral solution |
| 51280 | Efexor XL 150mg capsules (Waymade Healthcare Plc) |
| 51361 | Venlafaxine 37.5mg tablets (Ranbaxy (UK) Ltd) |
| 51383 | Duloxetine 60mg gastro-resistant capsules (Sigma Pharmaceuticals Plc) |
| 51699 | Venlafaxine 37.5mg/5ml oral solution |
| 51758 | Prothiaden 25mg capsules (Stephar (U.K.) Ltd) |
| 52074 | Alventa XL 75mg capsules (Consilient Health Ltd) |
| 52100 | Citalopram 10mg tablets (Arrow Generics Ltd) |
| 52354 | Citalopram 20mg tablets (DE Pharmaceuticals) |
| 52408 | Citalopram 10mg tablets (Kent Pharmaceuticals Ltd) |
| 52516 | Alventa XL 150mg capsules (Consilient Health Ltd) |
| 52607 | Citalopram 20mg tablets (Bristol Laboratories Ltd) |
| 52716 | Tonpular XL 75mg capsules (Wockhardt UK Ltd) |
| 52824 | Citalopram 10mg tablets (PLIVA Pharma Ltd) |
| 52867 | Amitriptyline 10mg tablets (Accord Healthcare Ltd) |
| 53161 | Clomipramine 50mg/5ml oral solution |
| 53187 | Clomipramine 50mg capsules (Kent Pharmaceuticals Ltd) |
| 53321 | Mirtazapine 15mg/ml oral solution sugar free (A A H Pharmaceuticals Ltd) |
| 53326 | Venlafaxine 75mg/5ml oral solution |
| 53394 | Citalopram 20mg tablets (Alliance Healthcare (Distribution) Ltd) |
| 53543 | Zispin SolTab 30mg orodispersible tablets (Necessity Supplies Ltd) |
| 53648 | Mirtazapine 30mg orodispersible tablets (Accord Healthcare Ltd) |
| 53699 | Mirtazapine 15mg tablets (Accord Healthcare Ltd) |
| 53787 | Citalopram 10mg tablets (Bristol Laboratories Ltd) |
| 53808 | Trimipramine 10mg tablets (Phoenix Healthcare Distribution Ltd) |
| 54012 | Mirtazapine 15mg orodispersible tablets sugar free (Sandoz Ltd) |
| 54081 | Sertraline 25mg/5ml oral suspension |
| 54342 | Mirtazapine 15mg tablets (Medreich Plc) |
| 54644 | Mirtazapine 15mg tablets (Pfizer Ltd) |
| 54792 | Mirtazapine 30mg tablets (Alliance Healthcare (Distribution) Ltd) |
| 54826 | Sertraline 150mg/5ml oral suspension |
| 54827 | Citalopram 10mg/5ml oral suspension |
| 54877 | Amitriptyline 25mg tablets (Accord Healthcare Ltd) |
| 54933 | Sertraline 100mg tablets (PLIVA Pharma Ltd) |
| 55023 | Paroxetine 20mg tablets (Medreich Plc) |
| 55033 | Citalopram 40mg tablets (DE Pharmaceuticals) |
| 55137 | Trazodone 150mg/5ml oral suspension |
| 55138 | Trazodone 250mg/5ml oral solution |
| 55139 | Amitriptyline 25mg tablets (Alliance Healthcare (Distribution) Ltd) |
| 55146 | Sertraline 100mg tablets (A A H Pharmaceuticals Ltd) |
| 55424 | Venlafaxine |
| 55482 | Mirtazapine 15mg orodispersible tablets (Mylan) |
| 55488 | Sertraline 50mg tablets (Teva UK Ltd) |
| 55491 | Amitriptyline 10mg tablets (Almus Pharmaceuticals Ltd) |
| 55501 | Venlafaxine 150mg Modified-release capsule (Hillcross Pharmaceuticals Ltd) |
| 55537 | Seroxat 30mg tablets (Lexon (UK) Ltd) |
| 55970 | Nortriptyline 10mg tablets (King Pharmaceuticals Ltd) |
| 56009 | Citalopram 20mg tablets (Arrow Generics Ltd) |
| 56209 | Mirtazapine 30mg tablets (Phoenix Healthcare Distribution Ltd) |
| 56229 | Lofepramine 70mg/5ml oral solution |
| 56292 | Citalopram 40mg/ml oral drops sugar free (Actavis UK Ltd) |
| 56355 | Citalopram 10mg tablets (Waymade Healthcare Plc) |
| 56457 | Venlafaxine 75mg tablets (Teva UK Ltd) |
| 56501 | Tofranil 25mg tablets (Lexon (UK) Ltd) |
| 56662 | Venlafaxine 37.5mg tablets (A A H Pharmaceuticals Ltd) |
| 56703 | Lofepramine 70mg tablets (Sandoz Ltd) |
| 57107 | Amitriptyline 10mg tablets (Phoenix Healthcare Distribution Ltd) |
| 57226 | Trazodone 25mg/5ml oral suspension |
| 57532 | Prozac 20mg capsules (Waymade Healthcare Plc) |
| 57751 | Tonpular XL 150mg capsules (Wockhardt UK Ltd) |
| 57926 | Dosulepin 75mg/5ml oral solution |
| 57936 | Citalopram 40mg/ml oral drops sugar free (A A H Pharmaceuticals Ltd) |
| 57972 | Amitriptyline 10mg tablets (Alliance Healthcare (Distribution) Ltd) |
| 57978 | Trimipramine 25mg tablets (Waymade Healthcare Plc) |
| 58291 | Mirtazapine 15mg orodispersible tablets (Pfizer Ltd) |
| 58450 | Feprapax 70mg tablets (Ashbourne Pharmaceuticals Ltd) |
| 58476 | Citalopram 20mg tablets (Aurobindo Pharma Ltd) |
| 58625 | Mirtazapine 45mg tablets (Accord Healthcare Ltd) |
| 58664 | Sertraline 50mg tablets (Mylan) |
| 58681 | Venladex XL 75mg tablets (Dexcel-Pharma Ltd) |
| 58723 | Sertraline 50mg tablets (Accord Healthcare Ltd) |
| 58726 | Venladex XL 150mg tablets (Dexcel-Pharma Ltd) |
| 58837 | Venlafaxine 37.5mg modified-release capsules |
| 59035 | Venlablue XL 37.5mg capsules (Creo Pharma Ltd) |
| 59161 | Amitriptyline 10mg tablets (Waymade Healthcare Plc) |
| 59193 | Citalopram 10mg tablets (Ranbaxy (UK) Ltd) |
| 59288 | Paroxetine 10mg tablets (Actavis UK Ltd) |
| 59358 | Fluoxetine 20mg capsules (Milpharm Ltd) |
| 59563 | Venlafaxine 75mg modified-release capsules (Kent Pharmaceuticals Ltd) |
| 59600 | Sertraline 100mg tablets (Almus Pharmaceuticals Ltd) |
| 59650 | Citalopram 10mg tablets (Aurobindo Pharma Ltd) |
| 59694 | Mirtazapine 30mg orodispersible tablets (Phoenix Healthcare Distribution Ltd) |
| 59753 | Sunveniz XL 150mg tablets (Sun Pharmaceuticals UK Ltd) |
| 59820 | Amitriptyline 50mg/5ml oral solution sugar free (Wockhardt UK Ltd) |
| 59923 | Venlafaxine 37.5mg tablets (Bristol Laboratories Ltd) |
| 59931 | Trazodone 50mg/5ml oral solution sugar free (A A H Pharmaceuticals Ltd) |
| 59953 | Mirtazapine 15mg tablets (Almus Pharmaceuticals Ltd) |
| 59954 | Mirtazapine 45mg tablets (Almus Pharmaceuticals Ltd) |
| 60138 | Fluoxetine 20mg orodispersible tablets sugar free |
| 60355 | Amitriptyline 25mg tablets (Phoenix Healthcare Distribution Ltd) |
| 60370 | Zispin SolTab 15mg orodispersible tablets (Mawdsley-Brooks & Company Ltd) |
| 60410 | Amitriptyline 25mg/5ml oral solution sugar free (Wockhardt UK Ltd) |
| 60449 | Venlafaxine 75mg tablets (A A H Pharmaceuticals Ltd) |
| 60534 | Fluoxetine 20mg dispersible tablets sugar free |
| 60538 | Mirtazapine 30mg tablets (DE Pharmaceuticals) |
| 60549 | Venlafaxine 150mg modified-release capsules (Kent Pharmaceuticals Ltd) |
| 60568 | Citalopram 20mg tablets (Waymade Healthcare Plc) |
| 60591 | Lofepramine 70mg tablets (Teva UK Ltd) |
| 60619 | Fluoxetine 20mg/5ml oral solution (Kent Pharmaceuticals Ltd) |
| 60839 | Citalopram 40mg tablets (Almus Pharmaceuticals Ltd) |
| 60843 | Sunveniz XL 75mg tablets (Sun Pharmaceuticals UK Ltd) |
| 60888 | Citalopram 10mg tablets (Sigma Pharmaceuticals Plc) |
| 60895 | Venlafaxine 37.5mg tablets (Teva UK Ltd) |
| 60962 | Fluoxetine 20mg capsules (Alliance Healthcare (Distribution) Ltd) |
| 61236 | Bonilux XL 150mg capsules (Sandoz Ltd) |
| 61335 | Prozac 20mg capsules (Mawdsley-Brooks & Company Ltd) |
| 61503 | Sertraline 100mg tablets (Actavis UK Ltd) |
| 61547 | Mirtazapine 15mg/ml oral solution sugar free (DE Pharmaceuticals) |
| 61657 | Trazodone 75mg/5ml oral solution |
| 61835 | Amitriptyline 10mg tablets (DE Pharmaceuticals) |
| 61842 | Trazodone 50mg/5ml oral solution |
| 61856 | Mirtazapine 15mg orodispersible tablets (Consilient Health Ltd) |
| 62155 | Fluoxetine 20mg capsules (Phoenix Healthcare Distribution Ltd) |
| 62335 | Olena 20mg dispersible tablets (AMCo) |
| 62620 | Clomipramine 10mg capsules (Mylan) |
| 62681 | Dosulepin 75mg tablets (Sandoz Ltd) |
| 62688 | Duloxetine 30mg gastro-resistant capsules (Sigma Pharmaceuticals Plc) |
| 62692 | Sertraline 100mg tablets (Bristol Laboratories Ltd) |
| 62693 | Sertraline 50mg tablets (Bristol Laboratories Ltd) |
| 62734 | Venlafaxine 150mg/5ml oral suspension |
| 62819 | Sertraline 12.5mg/5ml oral suspension |
| 62927 | Sertraline 50mg tablets (Wockhardt UK Ltd) |
| 62950 | Sertraline 100mg tablets (Accord Healthcare Ltd) |
| 63216 | Cymbalta 60mg gastro-resistant capsules (Mawdsley-Brooks & Company Ltd) |
| 63268 | Venlafaxine 75mg/5ml oral suspension |
| 63276 | Nortriptyline 25mg tablets (Alliance Healthcare (Distribution) Ltd) |
| 63370 | Duloxetine 30mg gastro-resistant capsules (Mawdsley-Brooks & Company Ltd) |
| 63403 | Mirtazapine 30mg tablets (Teva UK Ltd) |
| 63441 | Citalopram 10mg tablets (Rivopharm (UK) Ltd) |
| 63481 | Sertraline 50mg tablets (Milpharm Ltd) |
| 63763 | Duloxetine 60mg gastro-resistant capsules (A A H Pharmaceuticals Ltd) |
| 63859 | Venlafaxine 75mg tablets (Waymade Healthcare Plc) |
| 63916 | Escitalopram 10mg tablets (Actavis UK Ltd) |
| 63953 | Cipramil 20mg tablets (DE Pharmaceuticals) |
| 64000 | Amitriptyline 10mg/5ml oral solution sugar free |
| 64101 | Mirtazapine 15mg orodispersible tablets (Accord Healthcare Ltd) |
| 64139 | Mirtazapine 45mg orodispersible tablets (Mylan) |
| 64141 | Amitriptyline 5mg/5ml oral solution |
| 64223 | Mirtazapine 45mg tablets (Teva UK Ltd) |
| 64330 | Amitriptyline 50mg tablets (Almus Pharmaceuticals Ltd) |
| 64423 | Citalopram 10mg tablets (Accord Healthcare Ltd) |
| 64442 | Duloxetine 60mg gastro-resistant capsules (Teva UK Ltd) |
| 64458 | Clomipramine 25mg/5ml oral suspension |
| 64647 | Amitriptyline 25mg tablets (DE Pharmaceuticals) |
| 64785 | Paroxetine 30mg tablets (Alliance Healthcare (Distribution) Ltd) |
| 65152 | Trazodone 100mg/5ml oral solution |
| 65165 | Duloxetine 20mg gastro-resistant capsules (DE Pharmaceuticals) |
| 65213 | Trimipramine 50mg/5ml oral solution |
| 65237 | Nortriptyline 10mg tablets (A A H Pharmaceuticals Ltd) |
| 65439 | Amitriptyline 25mg tablets (Sandoz Ltd) |
| 65445 | Trimipramine 50mg capsules (A A H Pharmaceuticals Ltd) |
| 65482 | Vortioxetine 5mg tablets |
| 65483 | Vortioxetine 10mg tablets |
| 65555 | Mirtazapine 15mg orodispersible tablets (Sigma Pharmaceuticals Plc) |
| 65618 | Duloxetine 30mg gastro-resistant capsules (A A H Pharmaceuticals Ltd) |
| 65666 | Venlafaxine 225mg modified-release capsules |
| 65738 | Efexor 37.5mg tablets (Sigma Pharmaceuticals Plc) |
| 65762 | Clomipramine 25mg capsules (Waymade Healthcare Plc) |
| 65771 | Sertraline 200mg/5ml oral suspension (Special Order) |
| 65804 | Clomipramine 50mg capsules (Teva UK Ltd) |
| 65809 | Duloxetine 30mg gastro-resistant capsules (Actavis UK Ltd) |
| 65879 | Amitriptyline 10mg tablets (Sigma Pharmaceuticals Plc) |
| 65888 | Duloxetine 60mg gastro-resistant capsules (Actavis UK Ltd) |
| 65892 | Duloxetine 60mg gastro-resistant capsules (Mawdsley-Brooks & Company Ltd) |
| 65899 | Efexor XL 225mg capsules (Pfizer Ltd) |
| 65987 | Amitriptyline 25mg tablets (Crescent Pharma Ltd) |
| 66100 | Lofepramine 70mg tablets (DE Pharmaceuticals) |
| 66183 | Mirtazapine 15mg tablets (Alliance Healthcare (Distribution) Ltd) |
| 66201 | Nortriptyline 25mg tablets (Sigma Pharmaceuticals Plc) |
| 66292 | Seroxat 10mg tablets (Waymade Healthcare Plc) |
| 66405 | Duloxetine 60mg gastro-resistant capsules (DE Pharmaceuticals) |
| 66412 | Duloxetine 30mg gastro-resistant capsules (DE Pharmaceuticals) |
| 66413 | Sertraline 100mg tablets (Ranbaxy (UK) Ltd) |
| 66437 | Venlafaxine 75mg tablets (DE Pharmaceuticals) |
| 66493 | Trimipramine 25mg/5ml oral suspension |
| 66560 | Sertraline 100mg tablets (Mylan) |
| 66572 | Amitriptyline 25mg tablets (Sigma Pharmaceuticals Plc) |
| 66578 | Amitriptyline 10mg tablets (Mawdsley-Brooks & Company Ltd) |
| 66579 | Amitriptyline 25mg tablets (Mawdsley-Brooks & Company Ltd) |
| 66580 | Mirtazapine 15mg orodispersible tablets (Bluefish Pharmaceuticals AB) |
| 66744 | Fluoxetine 20mg capsules (Morningside Healthcare Ltd) |
| 66749 | Trazodone 10mg/5ml oral solution |
| 66752 | Mirtazapine 15mg tablets (Aurobindo Pharma Ltd) |
| 66890 | Vortioxetine 20mg tablets |
| 66919 | Trimipramine 50mg capsules (Waymade Healthcare Plc) |
| 67092 | Fluoxetine 20mg capsules (Waymade Healthcare Plc) |
| 67097 | Citalopram 20mg tablets (Accord Healthcare Ltd) |
| 67127 | Amitriptyline 25mg/5ml oral solution sugar free (DE Pharmaceuticals) |
| 67259 | Paroxetine 10mg/5ml oral solution |
| 67271 | Efexor 37.5mg tablets (Waymade Healthcare Plc) |
| 67272 | Zispin 30mg tablets (Waymade Healthcare Plc) |
| 67288 | Efexor 75mg tablets (Dowelhurst Ltd) |
| 67305 | Moclobemide 150mg tablets (Sigma Pharmaceuticals Plc) |
| 67431 | Fluoxetine 10mg capsules |
| 67496 | Fluoxetine 30mg capsules |
| 67562 | Fluoxetine 40mg capsules |
| 67563 | Vensir XL 225mg capsules (Morningside Healthcare Ltd) |
| 67564 | Duloxetine 20mg gastro-resistant capsules (Actavis UK Ltd) |
| 67728 | Dosulepin 75mg tablets (Alliance Healthcare (Distribution) Ltd) |
| 67730 | Sertraline 50mg tablets (Ranbaxy (UK) Ltd) |
| 67736 | Fluoxetine 20mg capsules (Dr Reddy's Laboratories (UK) Ltd) |
| 67742 | Lofepramine 70mg tablets (Mylan) |
| 67758 | Prozac 20mg capsules (DE Pharmaceuticals) |
| 67769 | Fluoxetine 20mg capsules (Strides Shasun (UK) Ltd) |
| 67874 | Brintellix 10mg tablets (Lundbeck Ltd) |
| 67888 | Fluoxetine 60mg capsules (Kent Pharmaceuticals Ltd) |
| 67928 | Sertraline 100mg tablets (Milpharm Ltd) |
| 67935 | Imipramine 10mg tablets (Almus Pharmaceuticals Ltd) |
| 67990 | Prothiaden 25mg capsules (Sigma Pharmaceuticals Plc) |
| 68050 | Venlafaxine 37.5mg tablets (Alliance Healthcare (Distribution) Ltd) |
| 68052 | Mirtazapine 30mg orodispersible tablets (Almus Pharmaceuticals Ltd) |
| 68096 | Duloxetine 60mg gastro-resistant capsules (Zentiva) |
| 68228 | Nortriptyline 10mg/5ml oral suspension |
| 68266 | Fluoxetine 20mg/5ml oral solution sugar free (Actavis UK Ltd) |
| 68325 | Paroxetine 40mg tablets |
| 68544 | Mirtazapine 2mg capsules |
| 68657 | Lofepramine 70mg tablets (Kent Pharmaceuticals Ltd) |
| 68665 | Clomipramine 10mg capsules (Almus Pharmaceuticals Ltd) |
| 68680 | Mirtazapine 15mg orodispersible tablets (Mawdsley-Brooks & Company Ltd) |
| 68756 | Sertraline 100mg tablets (Sandoz Ltd) |
| 68876 | Venlafaxine 75mg modified-release capsules (Mawdsley-Brooks & Company Ltd) |
| 68933 | Mirtazapine 30mg tablets (PLIVA Pharma Ltd) |
| 69005 | Mirtazapine 30mg tablets (Almus Pharmaceuticals Ltd) |
| 69317 | Nortriptyline 50mg tablets |
| 69355 | Trazodone Oral solution |
| 69420 | Mirtazapine 30mg orodispersible tablets (Aurobindo Pharma Ltd) |
| 69428 | Duloxetine 60mg gastro-resistant capsules (Alliance Healthcare (Distribution) Ltd) |
| 69525 | Fluoxetine 20mg capsules (Medreich Plc) |
| 69542 | Prozac 20mg capsules (Necessity Supplies Ltd) |
| 69571 | Citalopram 40mg tablets (Accord Healthcare Ltd) |
| 69685 | Fluoxetine 20mg/5ml oral solution sugar free (Morningside Healthcare Ltd) |
| 69712 | Amitriptyline 50mg tablets (Sigma Pharmaceuticals Plc) |
| 69725 | Sertraline 50mg tablets (Crescent Pharma Ltd) |
| 69726 | Sertraline 100mg tablets (Crescent Pharma Ltd) |
| 69752 | Duloxetine 60mg gastro-resistant capsules (Creo Pharma Ltd) |
| 69819 | Vencarm XL 37.5mg capsules (Aspire Pharma Ltd) |
| 69898 | Sertraline 50mg tablets (Sandoz Ltd) |
| 69941 | Fluoxetine 10mg capsules (A A H Pharmaceuticals Ltd) |
| 69965 | Duloxetine 60mg gastro-resistant capsules (Consilient Health Ltd) |
| 69991 | Brintellix 20mg tablets (Lundbeck Ltd) |
| 69992 | Brintellix 5mg tablets (Lundbeck Ltd) |
| 70063 | Duloxetine 20mg gastro-resistant capsules (A A H Pharmaceuticals Ltd) |
| 70287 | Imipramine 10mg tablets (Sigma Pharmaceuticals Plc) |
| 70300 | Amitriptyline 10mg/5ml oral solution sugar free (Alliance Healthcare (Distribution) Ltd) |
| 70315 | Vencarm XL 75mg capsules (Aspire Pharma Ltd) |
| 70353 | Venlafaxine 37.5mg tablets (DE Pharmaceuticals) |
| 70405 | Duloxetine 30mg gastro-resistant capsules (Teva UK Ltd) |
| 70420 | Vencarm XL 150mg capsules (Aspire Pharma Ltd) |
| 70495 | Vencarm XL 225mg capsules (Aspire Pharma Ltd) |
| 70521 | Trazodone 50mg/5ml oral solution sugar free (AMCo) |
| 70593 | Dosulepin 25mg/5ml oral suspension |
| 70728 | Duloxetine 30mg gastro-resistant capsules (Alliance Healthcare (Distribution) Ltd) |
| 70790 | Citalopram 40mg tablets (Aurobindo Pharma Ltd) |
| 70806 | Venlafaxine 150mg modified-release capsules (DE Pharmaceuticals) |
| 70838 | Dosulepin 25mg Capsule (Celltech Pharma Europe Ltd) |
| 70931 | Venlasov XL 75mg capsules (Sovereign Medical Ltd) |
| 70991 | Amitriptyline 10mg tablets (Arrow Generics Ltd) |
| 71005 | Citalopram 10mg tablets (DE Pharmaceuticals) |
| 71023 | Dosulepin 25mg/5ml oral solution sugar free (Special Order) |
| 71031 | Trazodone 50mg capsules (Actavis UK Ltd) |
| 71042 | Amitriptyline 25mg tablets (Arrow Generics Ltd) |
| 71059 | Dosulepin 75mg tablets (Sovereign Medical Ltd) |
| 71067 | Lofepramine 70mg tablets (Almus Pharmaceuticals Ltd) |
| 71253 | Imipramine 10mg tablets (DE Pharmaceuticals) |
| 71257 | Venlafaxine 75mg modified-release capsules (DE Pharmaceuticals) |
| 71543 | Mirtazapine 30mg tablets (Sigma Pharmaceuticals Plc) |
| 71669 | Duloxetine 20mg gastro-resistant capsules (Zentiva) |
| 71782 | Venlafaxine 150mg modified-release capsules (Mawdsley-Brooks & Company Ltd) |
| 71806 | Amphero XL 75mg capsules (Mylan) |
| 71848 | Citalopram 20mg/5ml oral suspension |
| 71852 | Fluoxetine 20mg capsules (Accord Healthcare Ltd) |
| 71932 | Venlasov XL 150mg capsules (Sovereign Medical Ltd) |
| 72124 | Citalopram 40mg/ml oral drops sugar free (Teva UK Ltd) |
| 72211 | Duloxetine 60mg gastro-resistant capsules (Dr Reddy's Laboratories (UK) Ltd) |
| 72291 | Trazodone 100mg/5ml oral solution sugar free |
| 72373 | Citalopram 20mg tablets (Ranbaxy (UK) Ltd) |
| 72626 | Nortriptyline 10mg/5ml oral solution |

# S1 Table G Codelist for depression – sensitivity analysis.

| medcode | readcode | Read term |
| --- | --- | --- |
| 543 | Eu32z11 | [X]Depression NOS |
| 2970 | Eu32z00 | [X]Depressive episode, unspecified |
| 3291 | Eu32z12 | [X]Depressive disorder NOS |
| 3292 | Eu33.00 | [X]Recurrent depressive disorder |
| 4639 | Eu32.00 | [X]Depressive episode |
| 5987 | Eu32z14 | [X] Reactive depression NOS |
| 6854 | Eu32y00 | [X]Other depressive episodes |
| 7604 | Eu32.13 | [X]Single episode of reactive depression |
| 7737 | Eu34113 | [X]Neurotic depression |
| 7749 | Eu41211 | [X]Mild anxiety depression |
| 7953 | Eu34100 | [X]Dysthymia |
| 8584 | Eu34111 | [X]Depressive neurosis |
| 8826 | Eu33.15 | [X]SAD - Seasonal affective disorder |
| 8851 | Eu33.11 | [X]Recurrent episodes of depressive reaction |
| 8902 | Eu33.13 | [X]Recurrent episodes of reactive depression |
| 9055 | Eu32.11 | [X]Single episode of depressive reaction |
| 9211 | Eu32100 | [X]Moderate depressive episode |
| 9667 | Eu32200 | [X]Severe depressive episode without psychotic symptoms |
| 10667 | Eu32400 | [X]Mild depression |
| 10720 | Eu32y11 | [X]Atypical depression |
| 11252 | Eu33212 | [X]Major depression, recurrent without psychotic symptoms |
| 11329 | Eu33211 | [X]Endogenous depression without psychotic symptoms |
| 11717 | Eu32000 | [X]Mild depressive episode |
| 11913 | Eu41200 | [X]Mixed anxiety and depressive disorder |
| 12099 | Eu32300 | [X]Severe depressive episode with psychotic symptoms |
| 15220 | Eu34114 | [X]Persistant anxiety depression |
| 16861 | Eu33315 | [X]Recurrent severe episodes of psychotic depression |
| 18510 | Eu32.12 | [X]Single episode of psychogenic depression |
| 19054 | Eu3y111 | [X]Recurrent brief depressive episodes |
| 19696 | Eu33.12 | [X]Recurrent episodes of psychogenic depression |
| 22116 | Eu33400 | [X]Recurrent depressive disorder, currently in remission |
| 22806 | Eu32212 | [X]Single episode major depression w'out psychotic symptoms |
| 23731 | Eu33311 | [X]Endogenous depression with psychotic symptoms |
| 24112 | Eu32313 | [X]Single episode of psychotic depression |
| 24117 | Eu32311 | [X]Single episode of major depression and psychotic symptoms |
| 27759 | Eu02z16 | [X] Senile dementia, depressed or paranoid type |
| 28248 | Eu32z13 | [X]Prolonged single episode of reactive depression |
| 28756 | Eu33.14 | [X]Seasonal depressive disorder |
| 28863 | Eu32314 | [X]Single episode of reactive depressive psychosis |
| 29520 | Eu33100 | [X]Recurrent depressive disorder, current episode moderate |
| 29784 | Eu33000 | [X]Recurrent depressive disorder, current episode mild |
| 32941 | Eu33313 | [X]Recurr severe episodes/major depression+psychotic symptom |
| 33469 | Eu33200 | [X]Recurr depress disorder cur epi severe without psyc sympt |
| 36616 | Eu33z11 | [X]Monopolar depression NOS |
| 41989 | Eu32211 | [X]Single episode agitated depressn w'out psychotic symptoms |
| 44300 | Eu33z00 | [X]Recurrent depressive disorder, unspecified |
| 47009 | Eu33300 | [X]Recurrent depress disorder cur epi severe with psyc symp |
| 47731 | Eu33y00 | [X]Other recurrent depressive disorders |
| 50998 | Eu3y000 | [X]Other single mood affective disorders |
| 52678 | Eu32312 | [X]Single episode of psychogenic depressive psychosis |
| 56609 | Eu32y12 | [X]Single episode of masked depression NOS |
| 59386 | Eu32213 | [X]Single episode vital depression w'out psychotic symptoms |
| 73991 | Eu33214 | [X]Vital depression, recurrent without psychotic symptoms |
| 98252 | Eu32600 | [X]Major depression, moderately severe |
| 98346 | Eu32500 | [X]Major depression, mild |
| 98414 | Eu32700 | [X]Major depression, severe without psychotic symptoms |
| 98417 | Eu32800 | [X]Major depression, severe with psychotic symptoms |

# S1 Table H Codelist for cognitive dysfunction – main analysis.

| medcode | readcode | readterm | Cognitive dysfunction |
| --- | --- | --- | --- |
| 1350 | E00..12 | senile/presenile dementia | Probable |
| 1916 | E00..11 | senile dementia | Probable |
| 1917 | F110.00 | alzheimer's disease | Probable |
| 2882 | E00z.00 | senile or presenile psychoses nos | Probable |
| 4357 | Eu02z14 | [x] senile dementia nos | Probable |
| 4693 | Eu02z00 | [x] unspecified dementia | Probable |
| 5931 | 1461 | h/o: dementia | Probable |
| 6578 | Eu01.00 | [x]vascular dementia | Probable |
| 7323 | E000.00 | uncomplicated senile dementia | Probable |
| 7572 | F116.00 | lewy body disease | Probable |
| 7664 | Eu00.00 | [x]dementia in alzheimer's disease | Probable |
| 7674 | 28E..00 | cognitive decline | Probable |
| 8195 | Eu00z11 | [x]alzheimer's dementia unspec | Probable |
| 8634 | E004.11 | multi infarct dementia | Probable |
| 8934 | Eu01200 | [x]subcortical vascular dementia | Probable |
| 9565 | Eu01.11 | [x]arteriosclerotic dementia | Probable |
| 10822 | Z7C1.00 | impaired cognition | Probable |
| 11136 | F111.00 | pick's disease | Probable |
| 11175 | Eu01100 | [x]multi-infarct dementia | Probable |
| 11379 | Eu00112 | [x]senile dementia,alzheimer's type | Probable |
| 11936 | Eu05700 | [x]mild cognitive disorder | Probable |
| 12621 | Eu02.00 | [x]dementia in other diseases classified elsewhere | Probable |
| 12710 | 6AB..00 | dementia annual review | Probable |
| 15165 | E001.00 | presenile dementia | Probable |
| 15249 | E00y.00 | other senile and presenile organic psychoses | Probable |
| 16797 | F110000 | alzheimer's disease with early onset | Probable |
| 18386 | E002000 | senile dementia with paranoia | Probable |
| 19393 | Eu01z00 | [x]vascular dementia, unspecified | Probable |
| 19477 | E004.00 | arteriosclerotic dementia | Probable |
| 21887 | E002100 | senile dementia with depression | Probable |
| 25386 | E041.00 | dementia in conditions ec | Probable |
| 25704 | Eu00011 | [x]presenile dementia,alzheimer's type | Probable |
| 26270 | Eu02500 | [x]lewy body dementia | Probable |
| 26323 | Eu10711 | [x]alcoholic dementia nos | Probable |
| 27342 | E012.11 | alcoholic dementia nos | Probable |
| 27677 | E001300 | presenile dementia with depression | Probable |
| 27759 | Eu02z16 | [x] senile dementia, depressed or paranoid type | Probable |
| 27935 | Eu02z15 | [x] senile psychosis nos | Probable |
| 28402 | Eu02000 | [x]dementia in pick's disease | Probable |
| 28626 | 13Y7.00 | alzheimer's disease society member | Probable |
| 29386 | Eu00z00 | [x]dementia in alzheimer's disease, unspecified | Probable |
| 30032 | E001200 | presenile dementia with paranoia | Probable |
| 30641 | 9hD0.00 | excepted from dementia quality indicators: patient unsuitabl | Probable |
| 30706 | Eu00200 | [x]dementia in alzheimer's dis, atypical or mixed type | Probable |
| 31016 | Eu01300 | [x]mixed cortical and subcortical vascular dementia | Probable |
| 32057 | F110100 | alzheimer's disease with late onset | Probable |
| 33707 | E00..00 | senile and presenile organic psychotic conditions | Probable |
| 34944 | Eu02z13 | [x] primary degenerative dementia nos | Probable |
| 37015 | E003.00 | senile dementia with delirium | Probable |
| 37072 | Eu03.00 | [x]organic amnesic synd not induced alc/oth psychoact subs | Probable |
| 38438 | E001z00 | presenile dementia nos | Probable |
| 38678 | Eu00100 | [x]dementia in alzheimer's disease with late onset | Probable |
| 40002 | ZS3..00 | language-related cognitive disorder | Probable |
| 40805 | 9hD1.00 | excepted from dementia quality indicators: informed dissent | Probable |
| 41089 | E002z00 | senile dementia with depressive or paranoid features nos | Probable |
| 42279 | E004z00 | arteriosclerotic dementia nos | Probable |
| 42602 | E001000 | uncomplicated presenile dementia | Probable |
| 43089 | E004000 | uncomplicated arteriosclerotic dementia | Probable |
| 43292 | E004300 | arteriosclerotic dementia with depression | Probable |
| 43346 | Eu00113 | [x]primary degen dementia of alzheimer's type, senile onset | Probable |
| 44341 | 9hD..00 | exception reporting: dementia quality indicators | Probable |
| 44674 | E002.00 | senile dementia with depressive or paranoid features | Probable |
| 46488 | Eu01000 | [x]vascular dementia of acute onset | Probable |
| 46762 | Eu00111 | [x]alzheimer's disease type 1 | Probable |
| 47619 | Eu02z12 | [x] presenile psychosis nos | Probable |
| 48501 | Eu02z11 | [x] presenile dementia nos | Probable |
| 49263 | Eu00000 | [x]dementia in alzheimer's disease with early onset | Probable |
| 49513 | E001100 | presenile dementia with delirium | Probable |
| 49674 | 9Ou1.00 | dementia monitoring first letter | Probable |
| 53446 | Eu04100 | [x]delirium superimposed on dementia | Probable |
| 54505 | E012.00 | other alcoholic dementia | Probable |
| 55023 | 66h..00 | dementia monitoring | Probable |
| 55222 | ZS7C500 | language disorder of dementia | Probable |
| 55313 | Eu01y00 | [x]other vascular dementia | Probable |
| 55467 | E004200 | arteriosclerotic dementia with paranoia | Probable |
| 55838 | Eu01111 | [x]predominantly cortical dementia | Probable |
| 56912 | E004100 | arteriosclerotic dementia with delirium | Probable |
| 57432 | Z7A1.00 | cognitive skills training | Probable |
| 59122 | Fyu3000 | [x]other alzheimer's disease | Probable |
| 60059 | Eu00012 | [x]primary degen dementia, alzheimer's type, presenile onset | Probable |
| 60263 | 3AE2.00 | gds level 3 - mild cognitive decline | Probable |
| 60726 | 3AE3.00 | gds level 4 - moderate cognitive decline | Probable |
| 61528 | Eu00013 | [x]alzheimer's disease type 2 | Probable |
| 62132 | E02y100 | drug-induced dementia | Probable |
| 64267 | Eu02y00 | [x]dementia in other specified diseases classif elsewhere | Probable |
| 65235 | 9Ou5.00 | dementia monitoring telephone invite | Probable |
| 70057 | 3AE4.00 | gds level 5 - moderately severe cognitive decline | Probable |
| 72520 | 3AE6.00 | gds level 7 - very severe cognitive decline | Probable |
| 83576 | 9Ou2.00 | dementia monitoring second letter | Probable |
| 85853 | 9Ou..00 | dementia monitoring administration | Probable |
| 89036 | 9Ou3.00 | dementia monitoring third letter | Probable |
| 89037 | 9Ou4.00 | dementia monitoring verbal invite | Probable |
| 94717 | 3AE5.00 | gds level 6 - severe cognitive decline | Probable |
| 103445 | 8Hla.00 | referral to dementia care advisor | Probable |
| 104534 | F118.00 | frontotemporal degeneration | Probable |
| 106311 | 8CMZ.00 | dementia care plan | Probable |
| 106627 | 8T05.00 | referral to dementia service | Probable |
| 107282 | 28E0.00 | mild cognitive impairment | Probable |
| 107389 | 38C1300 | assessment of psychotic and behavioural symptoms of dementia | Probable |
| 107402 | 28E2.00 | severe cognitive impairment | Probable |
| 107482 | 28E1.00 | moderate cognitive impairment | Probable |
| 108228 | 8CSA.00 | dementia advance care plan agreed | Probable |
| 108266 | 28E3.00 | cognitive impairment | Probable |
| 108268 | 8CMG200 | review of dementia advance care plan | Probable |
| 108391 | 8IAe000 | dementia advance care plan declined | Probable |
| 108612 | 8G18.00 | cognitive stimulation therapy | Probable |
| 109047 | 8BPa.00 | antipsychotic drug therapy for dementia | Probable |
| 109397 | 8T05000 | referral to dementia support organisation | Probable |
| 109708 | 8CMZ100 | dementia care plan reviewed | Probable |
| 109731 | 8CMZ000 | dementia care plan agreed | Probable |
| 109737 | 8BM0200 | dementia medication review | Probable |
| 109786 | 8CMZ200 | dementia care plan declined | Probable |
| 109790 | 8CMe000 | dementia advance care plan | Probable |
| 109834 | 8T05100 | referral to dementia support organisation declined | Probable |
| 110075 | 8IAe200 | dementia advance care plan review declined | Probable |
| 110119 | 8CET.00 | dementia leaflet given | Probable |
| 110123 | 8CMZ300 | dementia care plan review declined | Probable |
| 110729 | Eu05800 | [x]cognitive communication disorder | Probable |
| 111349 | 8T0Y.00 | referral to alzheimer's society | Probable |
| 1694 | 284..00 | o/e - disorientated | Possible |
| 1713 | 2841 | confused | Possible |
| 1993 | 1B1A.00 | memory loss - amnesia | Possible |
| 2908 | 1B1A.13 | memory disturbance | Possible |
| 3639 | 1B1A.11 | amnesia symptom | Possible |
| 4269 | E201700 | hysterical amnesia | Possible |
| 4284 | R00z000 | [d]amnesia (retrograde) | Possible |
| 4874 | 2232 | o/e - mentally confused | Possible |
| 5777 | 1B1A.12 | memory loss symptom | Possible |
| 6061 | E2A1100 | organic memory impairment | Possible |
| 6387 | E2A1000 | mild memory disturbance | Possible |
| 6489 | G655.00 | transient global amnesia | Possible |
| 6542 | 3A...12 | dementia assessment | Possible |
| 7389 | Eu04.12 | [x]acute / subacute confusional state, nonalcoholic | Possible |
| 7711 | R00z011 | [d]memory deficit | Possible |
| 7742 | 28G..00 | forgetful | Possible |
| 9786 | Z7CEC11 | loss of memory for recent events | Possible |
| 10123 | Z7CE611 | memory loss | Possible |
| 10493 | ZRaA.11 | mmse - mini-mental state examination | Possible |
| 10503 | ZRaA.00 | mini-mental state examination | Possible |
| 10514 | Z7CEH00 | memory impairment | Possible |
| 10571 | Z7CF811 | short-term memory loss | Possible |
| 11410 | Z7CF800 | poor short-term memory | Possible |
| 11735 | Z7CC300 | disorientated | Possible |
| 11862 | 388V.00 | mini mental state score | Possible |
| 12057 | Z7CEH14 | memory problem | Possible |
| 12277 | Z7CE616 | lom - loss of memory | Possible |
| 12583 | Z7CEH15 | poor memory | Possible |
| 12805 | Z7CE614 | memory loss - amnesia | Possible |
| 16968 | 2232.11 | o/e - confused | Possible |
| 18274 | 3A...11 | memory assessment | Possible |
| 18906 | Z7C..00 | cognitive function observations | Possible |
| 18996 | Z7CE700 | transient global amnesia | Possible |
| 19004 | Z7CE711 | tga - transient global amnesia | Possible |
| 19073 | Z7CEJ00 | memory lapses | Possible |
| 19297 | Z7CE615 | loss of memory | Possible |
| 19719 | Z7CC311 | orientation confused | Possible |
| 20683 | R00zX00 | [d]disorientation, unspecified | Possible |
| 21505 | 28...00 | nervous system and mental state general examination | Possible |
| 22257 | ZRa2.00 | microcog - assessment of cognitive function | Possible |
| 22802 | 8HTY.00 | referral to memory clinic | Possible |
| 24819 | ZRkB.00 | short orientation - memory - concentration test | Possible |
| 24952 | Z7CE500 | forgetful | Possible |
| 26141 | ZR1K.00 | alzheimer's disease assessment scale | Possible |
| 26142 | 3AE..00 | global deterioration scale: assessment of prim deg dementia | Possible |
| 26264 | ZRVa.00 | lowenstein ot cognitive assessment | Possible |
| 26326 | 3AD..00 | dementia test | Possible |
| 26421 | ZR1K.11 | adas - alzheimer's disease assessment scale | Possible |
| 26434 | Z7CEH13 | bad memory | Possible |
| 27788 | 1B1A000 | temporary loss of memory | Possible |
| 28278 | 1B1S.00 | transient global amnesia | Possible |
| 28406 | Z7CE600 | amnesia | Possible |
| 28704 | ZRkC.00 | short-term memory test | Possible |
| 32367 | Z7CEA11 | impairment of working memory | Possible |
| 32599 | Z7CFS00 | unable to remember own age | Possible |
| 35194 | ZRLfE00 | health of the nation outcome scale item 4 - cognitive probl | Possible |
| 35203 | ZR3V.11 | drs - clinical dementia rating scale | Possible |
| 35305 | 3AD3.00 | six item cognitive impairment test | Possible |
| 35538 | Z7CE900 | retrograde amnesia | Possible |
| 36637 | ZRaA200 | modified mini-mental state examination | Possible |
| 36650 | ZR3a.00 | cognitions questionnaire | Possible |
| 36658 | ZR2X.11 | memory concentration test | Possible |
| 36767 | Z7CE800 | anterograde amnesia | Possible |
| 37191 | Z7CEB12 | poor memory for remote events | Possible |
| 38025 | ZRhS.00 | rivermead behavioural memory test | Possible |
| 39471 | ZR3V.13 | dementia rating scale | Possible |
| 39915 | Z7CEH11 | memory dysfunction | Possible |
| 40091 | Z7CFI00 | forgets what has just done | Possible |
| 40994 | Eu44000 | [x]dissociative amnesia | Possible |
| 41366 | Z7CFH00 | forgets recent activities | Possible |
| 41993 | Z7CC400 | disorientated in time | Possible |
| 41994 | Z7CC500 | disorientated in place | Possible |
| 41995 | Z7CC611 | disorientated in person | Possible |
| 42059 | ZRaY.00 | neurobehavioural cognitive status examination | Possible |
| 43728 | 3AD1.00 | ten item dementia test | Possible |
| 43905 | Z7CE811 | antegrade amnesia | Possible |
| 44880 | ZR3V.00 | clinical dementia rating scale | Possible |
| 44956 | ZRrO.11 | wms - wechsler memory scale | Possible |
| 46314 | Z7CFq00 | unable to remember motor skills | Possible |
| 47581 | Z7CFO11 | long-term memory loss | Possible |
| 47882 | Z7CEG00 | transient memory loss | Possible |
| 47994 | Z7CFM00 | forgets what has just heard | Possible |
| 48513 | ZRa3.11 | meams - middlesex elderly assessment of mental state | Possible |
| 49265 | Z7A1300 | memory skills training | Possible |
| 50418 | Z7CEH12 | memory deficit | Possible |
| 50442 | ZRrX.00 | westmeade post-traumatic amnesia test | Possible |
| 50693 | ZR1H.00 | allen cognitive level screening tool | Possible |
| 50815 | ZR1I.00 | allen cognitive performance test | Possible |
| 51379 | Z7CE400 | memory disturbance (& amnesia (& symptom)) | Possible |
| 51724 | Z7CEL00 | mild memory disturbance | Possible |
| 51739 | Z7CEM00 | distortion of memory | Possible |
| 52781 | 3A9..00 | memory: count down | Possible |
| 52784 | 3A11.00 | memory: own age known | Possible |
| 52785 | 3A21.00 | memory: present time known | Possible |
| 52786 | 3A31.00 | memory: present place known | Possible |
| 52787 | 3A41.00 | memory: present year known | Possible |
| 52788 | 3A51.00 | memory: own dob known | Possible |
| 52789 | 3A61.00 | memory: present month known | Possible |
| 52790 | 3A81.00 | memory: important person known | Possible |
| 52791 | 3A90.00 | memory: count down successful | Possible |
| 52792 | 3AA0.00 | memory: address recall success | Possible |
| 52799 | 3A2..00 | memory: present time | Possible |
| 52800 | 3A70.00 | memory: important event not kn | Possible |
| 52801 | 3A80.00 | memory: import.person not knwn | Possible |
| 52804 | 3A71.00 | memory: important event known | Possible |
| 52805 | 3A91.00 | memory: count down unsuccess. | Possible |
| 52811 | Ryu5700 | [x]disorientation, unspecified | Possible |
| 52824 | 3A5..00 | memory: own dob | Possible |
| 52825 | 3A60.00 | memory: present month not knwn | Possible |
| 52826 | 3A8..00 | memory: important person | Possible |
| 52939 | Ryu5100 | [x]oth & unspec symptom/sign involv cognit funct/awareness | Possible |
| 52947 | 3A10.00 | memory: own age not known | Possible |
| 52948 | 3A40.00 | memory: present year not known | Possible |
| 53012 | 3A3..00 | memory: present place | Possible |
| 53014 | 3A30.00 | memory: present place not knwn | Possible |
| 53015 | 3A7..00 | memory: important event | Possible |
| 53016 | 3AA1.00 | memory: address recall unsucc. | Possible |
| 53125 | 3A50.00 | memory: own dob not known | Possible |
| 53146 | 3A20.00 | memory: present time not known | Possible |
| 53507 | Z7CEK00 | minor memory lapses | Possible |
| 53978 | Z7CFO00 | poor long-term memory | Possible |
| 54430 | Z7CFh00 | cannot remember birth dates of children | Possible |
| 54882 | R00z500 | [d]anterograde amnesia | Possible |
| 55460 | Z7CC700 | spatial disorientation | Possible |
| 57750 | Z7CFs00 | unable to remember new motor skills | Possible |
| 57812 | Z7CEE00 | amnesia for important personal information | Possible |
| 59387 | ZRaA100 | modified mini-mental state 3ms examination | Possible |
| 59502 | 3AD2.00 | thirty seven item dementia test | Possible |
| 59515 | Z7CFF00 | forgets what was going to do | Possible |
| 59830 | Z7CFw00 | memory aided by use of diary | Possible |
| 60600 | Z7CFG00 | forgets what was going to say | Possible |
| 61816 | Z7CEC00 | amnesia for recent events | Possible |
| 61830 | ZR1n.00 | autobiographical memory interview | Possible |
| 62114 | ZRVt.00 | measurement of cognitive linguistic ability | Possible |
| 62210 | ZRa3.00 | middlesex elderly assessment of mental state | Possible |
| 62749 | Z7CFi00 | cannot remember wedding anniversary | Possible |
| 63604 | ZRV9.11 | kendrick cognitive tests for the elderly | Possible |
| 63849 | Z7CFU00 | unable to remember day of the week | Possible |
| 63942 | Z7CFQ00 | unable to remember own date of birth | Possible |
| 64392 | ZRqa.00 | valentine auditory memory test | Possible |
| 64660 | ZR1n.12 | ami - autobiographical memory interview | Possible |
| 64813 | ZR2X.13 | information-memory-concentration test | Possible |
| 64892 | Z7CEN11 | invents experiences to compensate for loss of memory | Possible |
| 65365 | Z7A1500 | memory retraining | Possible |
| 65409 | ZRhS100 | rivermead behavioural memory test - adult version | Possible |
| 65696 | Z7CEA13 | impairment of primary memory | Possible |
| 65726 | ZR2V.00 | birmingham object recognition battery | Possible |
| 66012 | Z7CC600 | disorientation for person | Possible |
| 66080 | ZR3b.00 | cognitive failures questionnaire | Possible |
| 66144 | Z7CEB00 | amnesia for remote events | Possible |
| 66172 | Z7CA100 | isolated memory skills | Possible |
| 66271 | 2842 | disorientated in time | Possible |
| 66684 | Z7CFW00 | unable to remember today's date | Possible |
| 66686 | Z7CF.00 | observations of memory performance | Possible |
| 66927 | ZRrg.00 | wechsler memory scale revised | Possible |
| 67131 | ZR3V.12 | cdr - clinical dementia rating scale | Possible |
| 67163 | 1S21.00 | disturbance of memory for order of events | Possible |
| 67802 | Z7CEC12 | no memory for recent events | Possible |
| 67838 | Z7CE412 | memory loss symptom | Possible |
| 67951 | Z7CFz00 | memory aided by use of lists | Possible |
| 67998 | Z7CEF00 | temporary loss of memory | Possible |
| 68230 | Z7CE612 | memory gone | Possible |
| 68392 | Z7CED00 | amnesia for day to day facts | Possible |
| 69118 | Z7CFS11 | cannot remember own age | Possible |
| 70277 | Z7CF711 | good short-term memory | Possible |
| 70677 | Ryu5000 | [x]other amnesia | Possible |
| 70703 | Z7CE911 | ra - retrograde amnesia | Possible |
| 70723 | 3A1..00 | memory: own age | Possible |
| 70895 | Z7CFK00 | forgets what has just read | Possible |
| 72477 | Z7CFN11 | good long-term memory | Possible |
| 72737 | ZRrO.00 | wechsler memory scale | Possible |
| 73514 | ZRhS.11 | rbmt - rivermead behavioural memory test | Possible |
| 82481 | 388m.00 | mini-mental state examination | Possible |
| 83484 | 388m.11 | mmse score | Possible |
| 88787 | 7P10400 | neuropsychology test of memory | Possible |
| 91077 | 3A6..00 | memory: present month | Possible |
| 91078 | 3A4..00 | memory: present year | Possible |
| 92756 | ZRd..00 | rancho scale - levels of cognitive functioning | Possible |
| 93194 | ZRVt.11 | mcla - measurement of cognitive linguistic ability | Possible |
| 93236 | ZRh6.11 | rmt - recognition memory test | Possible |
| 93319 | Z7CFJ00 | forgets what has just said | Possible |
| 93856 | Z7CFe00 | unable to remember name of current prime minister | Possible |
| 94047 | Z7C2.00 | observations relating to recognition | Possible |
| 94164 | 9Nk1.00 | seen in memory clinic | Possible |
| 94513 | ZR2V.11 | borb - birmingham object recognition battery | Possible |
| 95076 | 3AA..00 | memory: address recall | Possible |
| 95853 | 311B.00 | cognitive assessment | Possible |
| 96549 | ZRV9.00 | kendrick battery for detection of dementia in the elderly | Possible |
| 96828 | ZRkL.00 | smith cognitive questionnaire | Possible |
| 96881 | ZRh6.00 | recognition memory test | Possible |
| 98256 | ZRVa.11 | lotca - lowenstein ot cognitive assessment | Possible |
| 98287 | Z7CFx00 | memory aided by use of labels | Possible |
| 98411 | ZRF..00 | fuld object memory evaluation | Possible |
| 98742 | ZR2X.12 | bdrs - blessed dementia rating scale | Possible |
| 98798 | Z7CGP00 | delayed verbal memory | Possible |
| 100140 | 3AF..00 | addenbrooke's cognitive examination revised | Possible |
| 101074 | 38Dv.00 | gpcog - general practitioner assessment of cognition | Possible |
| 101356 | ZD11300 | auditory memory therapy | Possible |
| 101710 | 38Dv100 | gpcog (gp assessment of cognition) informant interview | Possible |
| 102189 | ZR1T.00 | arizona battery for communication disorders of dementia | Possible |
| 102845 | ZR2X.00 | blessed dementia rating scale | Possible |
| 102880 | Z7CE415 | loss of memory | Possible |
| 103076 | 38Dv000 | gpcog (gp assessment of cognition) patient examination | Possible |
| 103375 | Z7CE413 | memory loss - amnesia | Possible |
| 103453 | 1B1A100 | short-term memory loss | Possible |
| 103479 | Z7CFL00 | forgets what has just seen | Possible |
| 104155 | 1JA2.00 | suspected dementia | Possible |
| 104472 | ZRrh.00 | williams memory assessment scales | Possible |
| 105538 | Z7CE414 | memory disturbance | Possible |
| 105793 | Z7CFg00 | cannot remember names of intimates | Possible |
| 106120 | Z7CGA12 | practice memory | Possible |
| 106295 | 8IEn.00 | referral to memory clinic declined | Possible |
| 106297 | 38C1000 | assessment for dementia | Possible |
| 106429 | ZRJ..11 | goat - galvestone orientation and amnesia test | Possible |
| 106868 | ZRLK.00 | graham-kendal memory for designs test | Possible |
| 106998 | ZRBg.00 | everyday memory questionnaire | Possible |
| 107351 | 38C1500 | initial memory assessment | Possible |
| 107396 | 9OqE.00 | dementia screening questionnaire completed | Possible |
| 107455 | 9OqD.00 | dementia screening questionnaire sent to patient | Possible |
| 108106 | 38Qa.00 | montreal cognitive assessment | Possible |
| 108150 | 38Q9.00 | tym (test your memory) test | Possible |
| 108582 | Z7CFa00 | unable to remember current year | Possible |
| 109237 | 9OqG.00 | everyday cognition questionnaire completed | Possible |
| 109599 | 38Qr.00 | oxford cognitive screen | Possible |
| 110258 | 38Qv.00 | everyday cognition questionnaire | Possible |
| 110307 | 1S23.00 | memory impairment | Possible |
| 110522 | 8T05200 | referral for dementia assessment | Possible |
| 111331 | ZRVb.00 | logical memory paragraph recall | Possible |

# S1 Table I Codelist for drugs used in cognitive dysfunction – main analysis.

| prodcode | productName |
| --- | --- |
| 2930 | Donepezil 5mg tablets |
| 2931 | Donepezil 10mg tablets |
| 5247 | Aricept 10mg tablets (Eisai Ltd) |
| 5334 | Reminyl 12mg tablets (Shire Pharmaceuticals Ltd) |
| 5400 | Aricept 5mg tablets (Eisai Ltd) |
| 6225 | Memantine 10mg tablets |
| 7329 | Galantamine 20mg/5ml oral solution sugar free |
| 7361 | Galantamine 24mg modified-release capsules |
| 9854 | Reminyl 4mg tablets (Shire Pharmaceuticals Ltd) |
| 9966 | Ebixa 5mg/0.5ml pump actuation oral solution (Lundbeck Ltd) |
| 10187 | Galantamine 4mg tablets |
| 10255 | Galantamine 8mg modified-release capsules |
| 11635 | Galantamine 12mg tablets |
| 11654 | Galantamine 8mg tablets |
| 11837 | Memantine 10mg/ml oral solution sugar free |
| 14309 | Galantamine 16mg modified-release capsules |
| 18062 | Reminyl 8mg tablets (Shire Pharmaceuticals Ltd) |
| 18587 | Reminyl XL 8mg capsules (Shire Pharmaceuticals Ltd) |
| 18800 | Ebixa 10mg tablets (Lundbeck Ltd) |
| 20140 | Reminyl XL 16mg capsules (Shire Pharmaceuticals Ltd) |
| 24088 | Reminyl XL 24mg capsules (Shire Pharmaceuticals Ltd) |
| 29288 | Reminyl 4mg/ml oral solution (Shire Pharmaceuticals Ltd) |
| 35088 | Donepezil 10mg orodispersible tablets sugar free |
| 35179 | Donepezil 5mg orodispersible tablets sugar free |
| 36848 | Aricept Evess 5mg orodispersible tablets (Eisai Ltd) |
| 37188 | Aricept Evess 10mg orodispersible tablets (Eisai Ltd) |
| 38976 | Memantine 5mg+10mg+15mg+20mg Tablet |
| 39240 | Memantine 20mg tablets |
| 39362 | Ebixa tablets treatment initiation pack (Lundbeck Ltd) |
| 39363 | Ebixa 20mg tablets (Lundbeck Ltd) |
| 48015 | Galsya XL 24mg capsules (Consilient Health Ltd) |
| 48442 | Donepezil 5mg orodispersible tablets |
| 48443 | Donepezil 10mg orodispersible tablets |
| 48482 | Galsya XL 8mg capsules (Consilient Health Ltd) |
| 53842 | Aricept 5mg tablets (Waymade Healthcare Plc) |
| 53922 | Donepezil 10mg orodispersible tablets (Consilient Health Ltd) |
| 55720 | Gatalin XL 24mg capsules (Aspire Pharma Ltd) |
| 56421 | Gatalin XL 8mg capsules (Aspire Pharma Ltd) |
| 56600 | Donepezil 5mg tablets (Zentiva) |
| 56709 | Gatalin XL 16mg capsules (Aspire Pharma Ltd) |
| 57139 | Ebixa 10mg tablets (DE Pharmaceuticals) |
| 58709 | Donepezil 10mg tablets (A A H Pharmaceuticals Ltd) |
| 58947 | Donepezil 10mg tablets (Accord Healthcare Ltd) |
| 59871 | Donepezil 10mg/5ml oral suspension |
| 59993 | Galzemic XL 16mg capsules (Creo Pharma Ltd) |
| 60107 | Donepezil 5mg tablets (Alliance Healthcare (Distribution) Ltd) |
| 60192 | Galzemic XL 8mg capsules (Creo Pharma Ltd) |
| 60493 | Galzemic XL 24mg capsules (Creo Pharma Ltd) |
| 61385 | Nemdatine 10mg tablets (Actavis UK Ltd) |
| 61476 | Acumor XL 24mg capsules (Mylan) |
| 61618 | Nemdatine 20mg tablets (Actavis UK Ltd) |
| 61676 | Donepezil 1mg/ml oral solution sugar free |
| 61920 | Luventa XL 8mg capsules (Fontus Health Ltd) |
| 61921 | Luventa XL 24mg capsules (Fontus Health Ltd) |
| 62867 | Gazylan XL 16mg capsules (Teva UK Ltd) |
| 62868 | Gazylan XL 24mg capsules (Teva UK Ltd) |
| 62925 | Acumor XL 16mg capsules (Mylan) |
| 63217 | Donepezil 5mg tablets (A A H Pharmaceuticals Ltd) |
| 63360 | Luventa XL 16mg capsules (Fontus Health Ltd) |
| 63405 | Galsya XL 16mg capsules (Consilient Health Ltd) |
| 64982 | Memantine 20mg tablets (Teva UK Ltd) |
| 65333 | Memantine 10mg/ml oral solution sugar free (Alliance Healthcare (Distribution) Ltd) |
| 65534 | Donepezil 5mg orodispersible tablets sugar free (A A H Pharmaceuticals Ltd) |
| 65573 | Gazylan XL 8mg capsules (Teva UK Ltd) |
| 66899 | Memantine 20mg orodispersible tablets sugar free |
| 66934 | Memantine 10mg orodispersible tablets sugar free |
| 67593 | Donepezil 10mg tablets (Zentiva) |
| 68792 | Memantine 10mg/ml oral solution sugar free (Chanelle Medical UK Ltd) |
| 68802 | Donepezil 5mg tablets (Waymade Healthcare Plc) |
| 68845 | Memantine 10mg/ml oral solution sugar free (A A H Pharmaceuticals Ltd) |
| 69595 | Marixino 20mg tablets (Consilient Health Ltd) |
| 69971 | Memantine 10mg/ml oral solution sugar free (Zentiva) |
| 70496 | Memantine 10mg soluble tablets sugar free |
| 70503 | Memantine 20mg soluble tablets sugar free |
| 71187 | Memantine 10mg tablets (A A H Pharmaceuticals Ltd) |
| 71960 | Donepezil 5mg tablets (Accord Healthcare Ltd) |
| 72344 | Lotprosin XL 16mg capsules (Actavis UK Ltd) |
| 72614 | Donepezil 5mg tablets (Mylan) |

# S1 Table J Codelist for fatigue – main analysis.

| medcode | readcode | Read term | Fatigue |
| --- | --- | --- | --- |
| 1147 | R007500 | [d]tiredness | Possible |
| 1371 | R007300 | [D]Lethargy | Possible |
| 1404 | 1682 | fatigue | Possible |
| 3361 | E205.00 | neurasthenia - nervous debility | Possible |
| 5049 | R007200 | [d]asthenia nos | Possible |
| 5583 | 168..12 | Lethargy - symptom | Possible |
| 5751 | 1683 | tired all the time | Possible |
| 5794 | 168..00 | tiredness symptom | Possible |
| 5814 | R007z11 | [D]Lassitude | Possible |
| 6242 | 168..11 | fatigue - symptom | Possible |
| 7235 | E205.12 | tired all the time | Possible |
| 9220 | 1688 | exhaustion | Possible |
| 9823 | 1684.11 | C/O - debility - malaise | Possible |
| 15516 | 1683.11 | c/o - 'tired all the time' | Possible |
| 16561 | Eu46000 | [x]neurasthenia | Possible |
| 17736 | 1684 | Malaise/lethargy | Possible |
| 20414 | 1684.13 | C/O - postviral syndrome | Possible |
| 23932 | R007z00 | [d]malaise and fatigue nos | Possible |
| 24382 | R204.00 | [d]senile exhaustion | Possible |
| 29292 | 168Z.00 | tiredness symptom nos | Possible |
| 43047 | R202.00 | [d]senile asthenia | Possible |
| 44215 | R007.00 | [d]malaise and fatigue | Possible |
| 97528 | 8Q1..11 | activity management for myalgic encephalopathy | Possible |
| 1040 | F286.15 | myalgic encephalomyelitis | Probable |
| 1042 | R007400 | [D]Postviral (asthenic) syndrome | Probable |
| 1688 | R007100 | [d]fatigue | Probable |
| 4364 | F03y.12 | myalgic encephalomyelitis | Probable |
| 4546 | F286.00 | chronic fatigue syndrome | Probable |
| 6190 | F286.12 | postviral fatigue syndrome | Probable |
| 6552 | F286.16 | me - myalgic encephalomyelitis | Probable |
| 7529 | F286.11 | cfs - chronic fatigue syndrome | Probable |
| 9127 | F286.14 | post-viral fatigue syndrome | Probable |
| 9656 | Eu46011 | [x]fatigue syndrome | Probable |
| 12411 | R007411 | [D]Post viral debility | Probable |
| 27877 | F286.13 | pvfs - postviral fatigue syn | Probable |
| 97140 | 8Q1..00 | activity management for chronic fatigue syndrome | Probable |
| 97284 | F286100 | moderate chronic fatigue syndrome | Probable |
| 98512 | F286000 | mild chronic fatigue syndrome | Probable |
| 98734 | F286200 | severe chronic fatigue syndrome | Probable |
| 99807 | 8HkW.00 | referral to chronic fatigue syndrome specialist team | Probable |
| 100414 | 8HlL.00 | referral for chronic fatigue syndrome activity management | Probable |
| 100464 | 8HkW.11 | referral to myalgic encephalomyelitis specialist team | Probable |
| 104407 | 8HlL.11 | referral for myalgic encephalopathy activity management | Probable |

# S1 Table K Codelist for fatigue – sensitivity analysis.

| medcode | readcode | readterm |
| --- | --- | --- |
| 9656 | Eu46011 | [x]fatigue syndrome |

# S1 Table L Codelist for sleep disorder – main analysis.

| medcode | readcode | readterm |
| --- | --- | --- |
| 750 | R005200 | [d]insomnia nos |
| 1244 | R005000 | [d]sleep disturbance, unspecified |
| 2129 | E274900 | nightmares |
| 2329 | Fy0..00 | sleep disorders |
| 3523 | 1B1B000 | initial insomnia |
| 4023 | E274111 | insomnia nos |
| 4537 | 1B1B.11 | c/o - insomnia |
| 4557 | R000100 | [d]somnolence |
| 4597 | 1B1B200 | late insomnia |
| 5675 | 1B1B100 | middle insomnia |
| 5921 | Fy00.00 | disorders of initiating and maintaining sleep |
| 6943 | Eu51300 | [x]sleepwalking |
| 7725 | 1B1Q.00 | poor sleep pattern |
| 7819 | E274.00 | non-organic sleep disorders |
| 8084 | R005.00 | [d]sleep disturbances |
| 8310 | 1B1B.12 | c/o - somnolence |
| 8853 | Eu51400 | [x]sleep terrors |
| 8913 | E274800 | night terrors |
| 8997 | Fy02.00 | disorders of the sleep-wake schedule |
| 10349 | R005.11 | [d]insomnia - symptom |
| 10421 | Fy01.00 | disorders of excessive somnolence |
| 10982 | E274311 | hypersomnia nos |
| 11721 | E274700 | somnambulism - sleep walking |
| 12072 | 8Q0..00 | sleep management |
| 15283 | K5A2100 | menopausal sleeplessness |
| 15407 | R005z00 | [d]sleep dysfunction nos |
| 15515 | E274100 | transient insomnia |
| 15732 | R005600 | [d]sleep rhythm irregular |
| 16115 | E274200 | persistent insomnia |
| 16434 | E274000 | unspecified non-organic sleep disorder |
| 16862 | E274A00 | sleep drunkenness |
| 17687 | Eu51511 | [x]dream anxiety disorder |
| 19514 | E274D11 | restless sleep |
| 21032 | Eu51z11 | [x]emotional sleep disorder nos |
| 21305 | 1B1B.00 | cannot sleep - insomnia |
| 22081 | Z1M..00 | sleep and rest interventions |
| 22819 | Eu51z00 | [x]nonorganic sleep disorder, unspecified |
| 23923 | Eu51200 | [x]nonorganic disorder of the sleep-wake schedule |
| 24894 | Eu51.00 | [x]nonorganic sleep disorders |
| 25211 | 1BX1.00 | excessive sleep |
| 25939 | E274400 | persistent hypersomnia |
| 26546 | E274.12 | insomnia due to nonorganic sleep disorder |
| 26547 | R005400 | [d]hypersomnia nos |
| 27649 | E274z00 | non-organic sleep disorder nos |
| 29288 | SL7z.11 | sleeping drug poisoning |
| 30626 | Eu51000 | [x]nonorganic insomnia |
| 31236 | R005.12 | [d]sleep rhythm problems |
| 32987 | E274E00 | 'short-sleeper' |
| 36992 | E274300 | transient hypersomnia |
| 37448 | TJ7z000 | adverse reaction to sleeping pill nos |
| 39990 | E274B00 | repeated rapid eye movement sleep interruptions |
| 41737 | R005900 | [d]sleep dysfunction with arousal disturbance |
| 41750 | Eu51100 | [x]nonorganic hypersomnia |
| 42847 | 1BX0.00 | delayed onset of sleep |
| 43098 | E274y00 | other non-organic sleep disorder |
| 44305 | Eu51500 | [x]nightmares |
| 46445 | U1A2.11 | [x]accidental poisoning with sleeping tablets |
| 47418 | U607z12 | [x] adverse reaction to sleeping pill nos |
| 48783 | E274D00 | repetitive intrusions of sleep |
| 53912 | Fyu5800 | [x]other sleep disorders |
| 54458 | R005800 | [d]sleep dysfunction with sleep stage disturbance |
| 55179 | E274C00 | other sleep stage or arousal dysfunction |
| 57487 | E274.11 | hypersomnia of non-organic origin |
| 62925 | Eu51y00 | [x]other nonorganic sleep disorders |
| 93615 | 9Nk0.00 | seen in sleep clinic |
| 95887 | 8HTn.00 | referral to sleep clinic |
| 96037 | 8G9B.00 | sleep hygiene behaviour education |
| 98268 | 38D1.00 | insomnia severity index |
| 101913 | Z1M1.00 | disturbing sleep |
| 101921 | 8G99.00 | sleep restriction therapy |
| 104942 | 38D0.00 | pittsburgh sleep quality index |
| 105440 | 1B6C.00 | excessive somnolence |
| 107666 | 9Ngt.00 | on melatonin for sleep disorder |
| 110184 | Fy06.00 | kleine-levin syndrome |

# S1 Table M Codelist for drugs used in sleep disorder – main analysis.

| prodcode | productName |
| --- | --- |
| 20 | Temazepam 10mg tablets |
| 35 | Nitrazepam 5mg tablets |
| 46 | Diazepam 2mg tablets |
| 47 | Diazepam 5mg tablets |
| 66 | Zopiclone 7.5mg tablets |
| 563 | Clomethiazole 192mg capsules |
| 721 | Zopiclone 3.75mg tablets |
| 780 | Temazepam 10mg/5ml oral solution sugar free |
| 918 | Phenergan 10mg tablets (Sanofi) |
| 921 | Temazepam 10mg capsules |
| 989 | Promethazine teoclate 25mg tablets |
| 1400 | Diazepam 10mg tablets |
| 1610 | Phenergan 5mg/5ml elixir (Sanofi) |
| 1729 | Temazepam 20mg tablets |
| 1943 | Avomine 25mg tablets (Manx Healthcare Ltd) |
| 1998 | Promethazine 5mg/5ml oral solution sugar free |
| 2017 | Zolpidem 5mg tablets |
| 2078 | Diazepam 5mg RecTubes (Wockhardt UK Ltd) |
| 2083 | Diazepam 10mg RecTubes (Wockhardt UK Ltd) |
| 2178 | Phensedyl Oral solution (Rhone-Poulenc Rorer Ltd) |
| 2352 | Diazepam 2mg/5ml oral solution |
| 2401 | Valium 2mg Tablet (Roche Products Ltd) |
| 2403 | Temazepam 20mg capsules |
| 2407 | Nitrazepam 5mg Capsule |
| 2535 | Clomethiazole 157.5mg/5ml oral solution sugar free |
| 2737 | Rohypnol 1mg tablets (Roche Products Ltd) |
| 3105 | Dalmane 30mg capsules (Meda Pharmaceuticals Ltd) |
| 3126 | Stilnoct 5mg tablets (Sanofi) |
| 3205 | Diazepam 5mg |
| 3320 | Zimovane 7.5mg tablets (Sanofi) |
| 3354 | Lormetazepam 1mg tablets |
| 3357 | Lormetazepam 1mg Capsule |
| 3491 | Heminevrin 192mg capsules (AstraZeneca UK Ltd) |
| 3494 | Promethazine hydrochloride 25mg tablets |
| 3524 | Mogadon 5mg Capsule (Roche Products Ltd) |
| 3582 | Phenergan 25mg tablets (Sanofi) |
| 3686 | Nitrazepam 10mg Tablet |
| 3687 | Lormetazepam 500microgram tablets |
| 3741 | Stilnoct 10mg tablets (Sanofi) |
| 3870 | Diazepam 2mg capsules |
| 3950 | Flurazepam 15mg capsules |
| 3956 | Dalmane 15mg capsules (Meda Pharmaceuticals Ltd) |
| 3973 | Tensium 10mg tablets (DDSA Pharmaceuticals Ltd) |
| 4140 | Oxazepam 30mg Capsule |
| 4141 | Oxazepam 15mg tablets |
| 4176 | Diazepam 10mg/2.5ml rectal solution tube |
| 4187 | Zimovane LS 3.75mg tablets (Sanofi) |
| 4203 | Medised Liquid (SSL International Plc) |
| 4338 | Valium 5mg Tablet (Roche Products Ltd) |
| 4395 | Stesolid 5mg rectal tube (Actavis UK Ltd) |
| 4566 | Oxazepam 10mg tablets |
| 5058 | Zileze 3.75 tablets (Opus Pharmaceuticals Ltd) |
| 5150 | Loprazolam 1mg tablets |
| 5459 | Zolpidem 10mg tablets |
| 5506 | Heminevrin 250mg/5ml syrup (AstraZeneca UK Ltd) |
| 5561 | Promethazine hydrochloride 10mg tablets |
| 5793 | Stesolid 10mg Rectal tubes (Dumex Ltd) |
| 5842 | Diazepam 2.5mg/1.25ml rectal solution tube |
| 6747 | Diazepam 5mg/2.5ml rectal solution tube |
| 7099 | Melatonin 2mg capsules |
| 7438 | Phenergan 25mg/ml Injection (Aventis Pharma) |
| 7566 | Flurazepam 30mg capsules |
| 7567 | Temazepam planpak Capsule (Manufacturer unknown) |
| 7569 | Temazepam 15mg capsules |
| 7786 | Mogadon 5mg Tablet (ICN Pharmaceuticals France S.A.) |
| 7924 | Nitrazepam 2.5mg/5ml oral suspension |
| 8334 | Diazepam 10mg suppositories |
| 8344 | Diazepam 5mg suppository |
| 8345 | Stesolid 10mg rectal tube (Accord Healthcare Ltd) |
| 8721 | Oxazepam 30mg Tablet |
| 8798 | Temazepam gelthix 10mg Capsule (Pharmacia Ltd) |
| 9045 | Diazepam 1mg/5ml suspension |
| 9065 | Diazepam 5mg/5ml oral solution |
| 9111 | Diazepam 10mg capsules |
| 9430 | Diazepam 2.5mg/5ml oral suspension |
| 9814 | Nitrazepam 5mg/5ml oral suspension |
| 10068 | Melatonin 1mg capsules |
| 10274 | Diazepam 2mg/5ml oral solution sugar free |
| 10402 | Valium 10mg Tablet (Roche Products Ltd) |
| 10430 | Temazepam 30mg capsules |
| 10901 | Paracetamol with promethazine hydrochloride 120mg+1.5mg/5ml suspension |
| 11004 | Promethazine hydrochloride 20mg tablets |
| 12124 | Diazepam 10mg/2ml solution for injection ampoules |
| 12214 | Promethazine hydrochloride 25mg/ml injection |
| 12237 | Diazepam 10mg/2ml emulsion for injection ampoules |
| 12293 | Normison 10mg Capsule (Wyeth Pharmaceuticals) |
| 12462 | Temazepam gelthix 20mg Capsule (Pharmacia Ltd) |
| 12849 | Diazepam 10mg/5ml oral suspension |
| 13023 | Melatonin 2.5mg capsules |
| 13756 | Diazemuls 10mg/2ml emulsion for injection ampoules (Accord Healthcare Ltd) |
| 14145 | Melatonin 3mg capsules |
| 14210 | Melatonin 2.5mg/5ml oral suspension |
| 14221 | Melatonin 1mg/ml sugar free Oral solution |
| 14250 | Melatonin 3mg tablets |
| 14365 | Zopiclone 3.75mg/5ml oral suspension |
| 14480 | Flunitrazepam 1mg tablets |
| 14955 | Phenergan Nightime 25mg tablets (Sanofi) |
| 15110 | Temazepam 10mg/5ml Oral solution (Generics (UK) Ltd) |
| 15492 | Nitrados 5mg Tablet (Rorer Pharmaceuticals Ltd) |
| 15852 | Zileze 7.5 tablets (Opus Pharmaceuticals Ltd) |
| 16610 | Valium 5mg/ml Injection (Roche Products Ltd) |
| 16734 | Diazepam rectubes 20mg Rectal tubes (C P Pharmaceuticals Ltd) |
| 16993 | Melatonin 3mg modified-release capsules |
| 17663 | Melatonin 1mg tablets |
| 18193 | Pholcodine 1.5mg/5ml / Promethazine 1.5mg/5ml oral solution sugar free |
| 18291 | Noctamid 1mg Tablet (Schering Health Care Ltd) |
| 18488 | Diazepam 2.5mg RecTubes (Wockhardt UK Ltd) |
| 18491 | Pamergan P100 solution for injection 2ml ampoules (Martindale Pharmaceuticals Ltd) |
| 19116 | Pethidine 100mg/2ml / Promethazine 50mg/2ml solution for injection ampoules |
| 19299 | Valium 5mg Capsule (Roche Products Ltd) |
| 19324 | Clomethiazole 8mg/ml IV infusion |
| 19450 | Mogadon 5mg tablets (Meda Pharmaceuticals Ltd) |
| 19479 | Tixylix night-time 1.5mg+1.5mg/5ml Oral solution (Novartis Consumer Health UK Ltd) |
| 20164 | Valium 2mg Capsule (Roche Products Ltd) |
| 20245 | Temazepam 10mg gel-fill capsules |
| 20514 | Valium 10mg Suppository (Roche Products Ltd) |
| 20801 | Temazepam gelthix 30mg Capsule (Pharmacia Ltd) |
| 20968 | Diazepam 2mg/5ml oral solution sugar free (Actavis UK Ltd) |
| 21359 | Sominex 20mg tablets (Teva UK Ltd) |
| 21437 | Loramet 1mg Capsule (Wyeth Pharmaceuticals) |
| 21454 | Normison 20mg Capsule (Wyeth Pharmaceuticals) |
| 22236 | Paracetamol with promethazine hydrochloride 120mg+1.5mg/5ml suspension sugar free colour free |
| 22267 | Tixylix night-time 1.5mg+1.5mg/5ml Oral solution (Novartis Consumer Health UK Ltd) |
| 23052 | Promethazine hydrochloride 1.5mg with paracetamol 120mg/5ml oral solution colour free and sugar free |
| 23107 | Night Nurse oral solution (GlaxoSmithKline Consumer Healthcare) |
| 23120 | Temazepam gelthix 15mg Capsule (Pharmacia Ltd) |
| 23205 | Dormonoct 1mg Tablet (Hoechst Marion Roussel) |
| 23820 | Diazepam 20mg rectal tubes |
| 23874 | Somnite 2.5mg/5ml oral suspension (Norgine Pharmaceuticals Ltd) |
| 24135 | Zopiclone 7.5mg tablets (Actavis UK Ltd) |
| 24321 | Paxane 30mg Capsule (M A Steinhard Ltd) |
| 24445 | Paracetamol with promethazine hydrochloride tablet |
| 24480 | Night Nurse capsules (GlaxoSmithKline Consumer Healthcare) |
| 25273 | Oxanid 10mg Tablet (M A Steinhard Ltd) |
| 26391 | Melatonin 10mg capsules |
| 26399 | Heminevrin 8mg/ml Intravenous infusion (AstraZeneca UK Ltd) |
| 27367 | Temazepam 10mg/5ml oral solution sugar free (Rosemont Pharmaceuticals Ltd) |
| 27847 | Surem 5mg Capsule (Galen Ltd) |
| 27988 | Pholcodine with promethazine hydrochloride linctus |
| 28347 | Diazepam 2mg Tablet (Crosspharma Ltd) |
| 28698 | Valium 5mg Suppository (Roche Products Ltd) |
| 28703 | Evacalm 5mg Tablet (Unimed Pharmaceuticals Ltd) |
| 28792 | Medised colour and sf 120mg+1.5mg/5ml Liquid (SSL International Plc) |
| 29219 | Zopiclone 3.75mg tablets (Actavis UK Ltd) |
| 29441 | Euhypnos forte 20mg Capsule (Pharmacia Ltd) |
| 29732 | Promethazine hydrochloride 1.5mg with pholcodine 1.5mg/5ml oral solution |
| 29869 | Zolpidem 5mg Tablet (Winthrop Pharmaceuticals Ltd) |
| 29945 | Diazepam 2mg tablets (Ranbaxy (UK) Ltd) |
| 30056 | Zopiclone 3.75mg tablets (IVAX Pharmaceuticals UK Ltd) |
| 30321 | Valium 2mg/5ml Oral solution (Roche Products Ltd) |
| 30377 | Zopiclone 3.75mg tablets (Mylan) |
| 30590 | Medised Tablet (SSL International Plc) |
| 30779 | Temazepam 20mg Tablet (Wyeth Pharmaceuticals) |
| 30981 | Zolpidem 10mg tablets (A A H Pharmaceuticals Ltd) |
| 30985 | Temazepam 20mg tablets (IVAX Pharmaceuticals UK Ltd) |
| 31633 | Valclair 10mg suppositories (Durbin Plc) |
| 31710 | Zolpidem 5mg tablets (A A H Pharmaceuticals Ltd) |
| 31889 | Ziz 10mg tablets (Chatfield Laboratories) |
| 32296 | Diazepam 5mg tablets (Actavis UK Ltd) |
| 32320 | Temazepam 20mg gel-fill capsules |
| 32417 | Diazepam 10mg/2ml solution for injection ampoules (Wockhardt UK Ltd) |
| 32847 | Temazepam 10mg/5ml oral solution sugar free (A A H Pharmaceuticals Ltd) |
| 32853 | Diazepam 2mg/5ml oral solution (Sandoz Ltd) |
| 33045 | Zopiclone 7.5mg tablets (IVAX Pharmaceuticals UK Ltd) |
| 33070 | Solis 5mg Capsule (Galen Ltd) |
| 33230 | Promethazine hydrochloride 1.5mg with paracetamol 120mg/5ml oral solution |
| 33648 | Temazepam 10mg Tablet (Wyeth Pharmaceuticals) |
| 33663 | Zopiclone 7.5mg tablets (Mylan) |
| 33672 | Diazepam 2mg tablets (A A H Pharmaceuticals Ltd) |
| 33776 | Diazepam 10mg/2ml solution for injection ampoules (Hameln Pharmaceuticals Ltd) |
| 33841 | Zolpidem 10mg tablets (Mylan) |
| 33954 | Promethazine hydrochloride 50mg with pethidine 100mg/2ml injection |
| 34002 | Temazepam 10mg tablets (IVAX Pharmaceuticals UK Ltd) |
| 34033 | Diazepam 5mg/2.5ml rectal solution tube (Sandoz Ltd) |
| 34045 | Diazepam 5mg/5ml oral solution (Sandoz Ltd) |
| 34292 | Lormetazepam 500microgram tablets (A A H Pharmaceuticals Ltd) |
| 34293 | Diazepam 10mg tablets (Mylan) |
| 34331 | Temazepam 10mg tablets (A A H Pharmaceuticals Ltd) |
| 34335 | Diazepam 2mg tablets (Teva UK Ltd) |
| 34338 | Diazepam 2mg tablets (Mylan) |
| 34340 | Diazepam 10mg tablets (Ranbaxy (UK) Ltd) |
| 34361 | Lormetazepam 500microgram tablets (Thornton & Ross Ltd) |
| 34372 | Zopiclone 7.5mg tablets (PLIVA Pharma Ltd) |
| 34406 | Temazepam 10mg tablets (Teva UK Ltd) |
| 34408 | Nitrazepam 5mg tablets (A A H Pharmaceuticals Ltd) |
| 34482 | Diazepam 5mg tablets (Teva UK Ltd) |
| 34508 | Temazepam 10mg tablets (Mylan) |
| 34516 | Lormetazepam 1mg tablets (Mylan) |
| 34524 | Diazepam 2mg tablets (Actavis UK Ltd) |
| 34534 | Lormetazepam 1mg Tablet (Wyeth Pharmaceuticals) |
| 34555 | Nitrazepam 5mg tablets (Wockhardt UK Ltd) |
| 34561 | Diazepam 2mg Tablet (Regent Laboratories Ltd) |
| 34572 | Temazepam 20mg tablets (Mylan) |
| 34612 | Zopiclone 3.75mg tablets (A A H Pharmaceuticals Ltd) |
| 34614 | Diazepam 10mg/2.5ml rectal solution tube (Sandoz Ltd) |
| 34615 | Diazepam 5mg tablets (Mylan) |
| 34635 | Diazepam 5mg tablets (A A H Pharmaceuticals Ltd) |
| 34642 | Lormetazepam 500microgram tablets (Mylan) |
| 34677 | Diazepam 2mg tablets (IVAX Pharmaceuticals UK Ltd) |
| 34681 | Diazepam 5mg Tablet (Crosspharma Ltd) |
| 34686 | Nitrazepam 5mg tablets (Teva UK Ltd) |
| 34692 | Lormetazepam 1mg tablets (Thornton & Ross Ltd) |
| 34770 | Nitrazepam 5mg Tablet (DDSA Pharmaceuticals Ltd) |
| 34777 | Zopiclone 3.75mg tablets (Teva UK Ltd) |
| 34806 | Nitrazepam 5mg tablets (Mylan) |
| 34807 | Diazepam 10mg tablets (Actavis UK Ltd) |
| 34823 | Zopiclone 7.5mg tablets (Teva UK Ltd) |
| 34874 | Zopiclone 7.5mg tablets (Kent Pharmaceuticals Ltd) |
| 34876 | Diazepam 2mg Tablet (Berk Pharmaceuticals Ltd) |
| 34892 | Diazepam 5mg Tablet (Berk Pharmaceuticals Ltd) |
| 34897 | Zopiclone 3.75mg tablets (Kent Pharmaceuticals Ltd) |
| 34964 | Nitrazepam 5mg Tablet (Berk Pharmaceuticals Ltd) |
| 35142 | Vytalonin 3mg Tablet (IDIS World Medicines) |
| 35224 | Melatonin 5mg capsules |
| 35437 | Phenergan 25mg/1ml solution for injection ampoules (Sanofi) |
| 35846 | Promethazine 25mg/1ml solution for injection ampoules |
| 35857 | Tixylix Night Cough oral solution (Novartis Consumer Health UK Ltd) |
| 36581 | Atensine 10mg Tablet (Rorer Pharmaceuticals Ltd) |
| 36602 | Temazepam 20mg Tablet (Pharmacia Ltd) |
| 36604 | Oxazepam 10mg tablets (A A H Pharmaceuticals Ltd) |
| 36611 | Euhypnos 10mg/5ml Oral solution (Pharmacia Ltd) |
| 37325 | Remnos 10mg Tablet (DDSA Pharmaceuticals Ltd) |
| 38208 | Melatonin 2mg modified-release tablets |
| 38265 | Circadin 2mg modified-release tablets (Flynn Pharma Ltd) |
| 38410 | Diazepam 5mg Rectal tubes (Hillcross Pharmaceuticals Ltd) |
| 38418 | Temazepam 10mg Capsule (Berk Pharmaceuticals Ltd) |
| 38424 | Temazepam 20mg Capsule (Berk Pharmaceuticals Ltd) |
| 41385 | Nitrazepam 5mg tablets (Actavis UK Ltd) |
| 41411 | Oxazepam 15mg tablets (A A H Pharmaceuticals Ltd) |
| 41516 | Temazepam 10mg Tablet (IVAX Pharmaceuticals UK Ltd) |
| 41531 | Oxazepam 10mg tablets (Actavis UK Ltd) |
| 41539 | Zolpidem 10mg tablets (IVAX Pharmaceuticals UK Ltd) |
| 41542 | Oxazepam 10mg Tablet (IVAX Pharmaceuticals UK Ltd) |
| 41553 | Oxazepam 10mg tablets (Thornton & Ross Ltd) |
| 41562 | Temazepam 10mg Tablet (Pharmacia Ltd) |
| 41596 | Loprazolam 1mg tablets (Zentiva) |
| 41601 | Oxazepam 15mg Tablet (IVAX Pharmaceuticals UK Ltd) |
| 41602 | Oxazepam 15mg tablets (Actavis UK Ltd) |
| 41607 | Diazepam 10mg tablets (Teva UK Ltd) |
| 41632 | Diazepam 10mg tablets (A A H Pharmaceuticals Ltd) |
| 41653 | Temazepam 20mg Capsule (Hillcross Pharmaceuticals Ltd) |
| 41689 | Diazepam 10mg Suppository (Sinclair IS Pharma Plc) |
| 41696 | Zolpidem 5mg tablets (Teva UK Ltd) |
| 41697 | Zolpidem 5mg tablets (IVAX Pharmaceuticals UK Ltd) |
| 41717 | Temazepam 20mg tablets (A A H Pharmaceuticals Ltd) |
| 41718 | Temazepam 10mg Capsule (Hillcross Pharmaceuticals Ltd) |
| 41961 | Melatonin Tablet |
| 42089 | Zolpidem 10mg Tablet (Winthrop Pharmaceuticals Ltd) |
| 42503 | Diazepam 5mg/5ml oral solution (A A H Pharmaceuticals Ltd) |
| 43445 | Zopiclone 7.5mg tablets (A A H Pharmaceuticals Ltd) |
| 43560 | Zolpidem 10mg tablets (Teva UK Ltd) |
| 45135 | Diazepam 2mg Tablet (M & A Pharmachem Ltd) |
| 45218 | Diazepam 5mg tablets (Ranbaxy (UK) Ltd) |
| 45230 | Melatonin Capsule |
| 45244 | Diazepam 5mg tablets (Sandoz Ltd) |
| 45254 | Temazepam 10mg tablets (Actavis UK Ltd) |
| 45283 | Temazepam 10mg tablets (Genus Pharmaceuticals Ltd) |
| 45313 | Diazepam 2mg tablets (Almus Pharmaceuticals Ltd) |
| 45353 | Zopiclone 7.5mg tablets (Sandoz Ltd) |
| 45367 | Bio-Melatonin 3mg tablets (Imported (Denmark)) |
| 45783 | Melatonin 5mg/5ml oral suspension |
| 45975 | Melatonin 5mg/5ml oral solution |
| 46078 | Temazepam 20mg tablets (Genus Pharmaceuticals Ltd) |
| 46799 | Zopiclone 3.75mg/5ml oral solution |
| 46913 | Diazepam 10mg tablets (IVAX Pharmaceuticals UK Ltd) |
| 46939 | Temazepam 20mg tablets (Teva UK Ltd) |
| 46946 | Oxazepam 15mg tablets (Thornton & Ross Ltd) |
| 46953 | Nitrazepam 5mg tablets (Ranbaxy (UK) Ltd) |
| 46964 | Temazepam 20mg tablets (Actavis UK Ltd) |
| 46966 | Diazepam 5mg tablets (IVAX Pharmaceuticals UK Ltd) |
| 47056 | Promethazine 50mg/2ml solution for injection ampoules |
| 48436 | Melatonin 6mg capsules |
| 48517 | Lormetazepam 1mg/5ml oral suspension |
| 49196 | Melatonin 2mg/5ml oral suspension |
| 49576 | Melatonin 500microgram tablets |
| 49589 | Temazepam 10mg tablets (Sandoz Ltd) |
| 49968 | Phenergan 25mg tablets (DE Pharmaceuticals) |
| 50115 | Melatonin 4mg capsules |
| 50258 | Melatonin 1mg/1ml oral liquid sugar free |
| 51335 | Diazepam 10mg/5ml oral solution |
| 51754 | Diazepam 10mg/2ml solution for injection ampoules (A A H Pharmaceuticals Ltd) |
| 51985 | Diazepam 2mg/5ml oral suspension |
| 52022 | Zimovane 7.5mg tablets (Lexon (UK) Ltd) |
| 52079 | Melatonin 2mg tablets |
| 52289 | Melatonin 3mg/5ml oral suspension |
| 52303 | Melatonin 20mg capsules |
| 52487 | Melatonin 2mg/5ml oral solution |
| 52683 | Melatonin 1mg/5ml oral suspension |
| 53064 | Melatonin 6mg/5ml oral suspension |
| 53461 | Diazepam 2mg/5ml oral solution sugar free (A A H Pharmaceuticals Ltd) |
| 53566 | Diazepam 2.5mg/5ml oral solution |
| 54695 | Diazepam 10mg Tablet (M & A Pharmachem Ltd) |
| 54717 | Melatonin 10mg/5ml oral suspension |
| 55100 | Melatonin 5mg/5ml oral solution (Drug Tariff Special Order) |
| 55191 | Melatonin 6mg/5ml oral solution |
| 55836 | Temazepam 10mg/5ml oral solution sugar free (Focus Pharmaceuticals Ltd) |
| 55838 | Phenergan 25mg tablets (Lexon (UK) Ltd) |
| 55860 | Melatonin 3mg modified-release capsules (Imported (United States)) |
| 56236 | Diazepam 2mg tablets (Wockhardt UK Ltd) |
| 56393 | Melatonin 5mg tablets |
| 56811 | Temazepam 10mg tablets (F.Maltby & Sons Ltd) |
| 56927 | Temazepam 10mg tablets (Ethigen Ltd) |
| 57092 | Night Nurse Hot Lemon Menthol oral powder sachets (GlaxoSmithKline Consumer Healthcare) |
| 57406 | Melatonin 1mg/5ml oral solution |
| 57749 | Diazepam 5mg tablets (Waymade Healthcare Plc) |
| 57838 | Diazepam 5mg tablets (Sovereign Medical Ltd) |
| 57937 | Zopiclone 3.75mg tablets (Almus Pharmaceuticals Ltd) |
| 58361 | Clomethiazole 31.5mg/ml oral solution sugar free (A A H Pharmaceuticals Ltd) |
| 58566 | Melatonin 4mg/5ml oral solution |
| 58692 | Melatonin 3mg/5ml oral solution |
| 58959 | Diazepam 10mg/5ml oral solution (AM Distributions (Yorkshire) Ltd) |
| 59122 | Diazepam 5mg/5ml oral solution (AM Distributions (Yorkshire) Ltd) |
| 59170 | Clomethiazole 192mg capsules (A A H Pharmaceuticals Ltd) |
| 59407 | Diazepam 5mg tablets (DE Pharmaceuticals) |
| 59640 | Zopiclone 7.5mg/5ml oral suspension |
| 59801 | Generic Night Nurse oral solution |
| 60825 | Temazepam 20mg tablets (Sandoz Ltd) |
| 60936 | Diazepam 5mg tablets (Arrow Generics Ltd) |
| 61477 | Zopiclone 7.5mg/5ml oral solution |
| 61678 | Nitrazepam 2mg/5ml oral suspension |
| 62216 | Diazepam 10mg tablets (Almus Pharmaceuticals Ltd) |
| 62411 | Melatonin 3mg orodispersible tablets |
| 62501 | Melatonin 10mg/5ml oral solution |
| 62541 | Diazepam 2mg tablets (Sovereign Medical Ltd) |
| 62590 | Promethazine 25mg/5ml oral suspension |
| 62645 | Temazepam 20mg Tablet (Lagap) |
| 62980 | Promethazine 25mg/5ml oral solution |
| 63050 | Melatonin 2mg modified-release capsules |
| 63238 | Diazepam 2mg tablets (Waymade Healthcare Plc) |
| 63592 | Zopiclone 7.5mg tablets (Sigma Pharmaceuticals Plc) |
| 63665 | Noctamid 0.5mg Tablet (Schering Health Care Ltd) |
| 63674 | Temazepam 30mg gel-fill capsules |
| 63694 | Diazepam 2mg tablets (Phoenix Healthcare Distribution Ltd) |
| 64002 | Melatonin 1mg/1ml oral liquid sugar free (Special Order) |
| 64200 | Diazepam 2mg tablets (Alliance Healthcare (Distribution) Ltd) |
| 64249 | Melatonin 2.5mg/5ml oral solution |
| 64614 | Melatonin 2mg capsules (Alissa Healthcare Research Ltd) |
| 64775 | Nitrazepam 10mg/5ml oral suspension |
| 64932 | Generic Night Nurse capsules |
| 64951 | Melatonin 8mg capsules |
| 65093 | Melatonin 2mg capsules (Imported (Denmark)) |
| 65135 | Nitrazepam 2.5mg/5ml oral suspension sugar free |
| 65190 | Zolpidem 10mg tablets (Zentiva) |
| 65375 | Melatonin 5mg/5ml oral suspension (Drug Tariff Special Order) |
| 65532 | Melatonin 3mg modified-release tablets |
| 65637 | Zopiclone 7.5mg tablets (Phoenix Healthcare Distribution Ltd) |
| 65844 | Melatonin 3mg capsules (Imported (Denmark)) |
| 66745 | Loprazolam 1mg tablets (Stephar (U.K.) Ltd) |
| 67192 | Diazepam 5mg/5ml oral suspension (Sandoz Ltd) |
| 67193 | Diazepam 2mg/5ml oral suspension (Sandoz Ltd) |
| 67297 | Diazepam 25mg/5ml oral solution |
| 67451 | Diazepam 2mg tablets (Mawdsley-Brooks & Company Ltd) |
| 67785 | Diazepam 5mg tablets (Wockhardt UK Ltd) |
| 67957 | Diazepam 5mg/5ml oral suspension (A A H Pharmaceuticals Ltd) |
| 68344 | Melatonin 7.5mg capsules |
| 68414 | Diazepam 2mg/5ml oral solution sugar free (Alliance Healthcare (Distribution) Ltd) |
| 68588 | Melatonin 4mg/5ml oral suspension |
| 68772 | Clomethiazole 31.5mg/ml oral solution sugar free (Intrapharm Laboratories Ltd) |
| 68938 | Melatonin 3mg/5ml oral solution (Drug Tariff Special Order) |
| 69783 | Diazepam 2mg/5ml oral suspension (A A H Pharmaceuticals Ltd) |
| 69810 | Diazepam 5mg tablets (Crescent Pharma Ltd) |
| 69979 | Circadin 2mg modified-release tablets (DE Pharmaceuticals) |
| 70071 | Temazepam 10mg tablets (Alliance Healthcare (Distribution) Ltd) |
| 70536 | Diazepam 1mg/5ml oral solution |
| 70564 | Melatonin 10mg tablets |
| 70583 | Melatonin 4mg tablets |
| 70727 | Zopiclone 3.75mg tablets (Sigma Pharmaceuticals Plc) |
| 71073 | Nitrazepam 5mg tablets (Pharmvit Ltd) |
| 71089 | Zopiclone 3.75mg tablets (Bristol Laboratories Ltd) |
| 71143 | Clomethiazole 250mg/5ml oral solution |
| 71245 | Diazepam 5mg tablets (Relonchem Ltd) |
| 71336 | Diazepam 2mg tablets (DE Pharmaceuticals) |

# S1 Table N Codelist for opioid analgesics – main analysis.

| prodcode | Product Name |
| --- | --- |
| 53 | Dihydrocodeine 30mg tablets |
| 86 | Tramadol 50mg capsules |
| 123 | Fortral 30mg/ml Injection (Sterwin Medicines) |
| 187 | Zydol 50mg capsules (Grunenthal Ltd) |
| 191 | Dihydrocodeine 10mg/5ml oral solution |
| 234 | Pethidine 25mg tablet |
| 249 | Pethidine 10mg/ml injection |
| 320 | Buprenorphine HCl 300micrograms injection |
| 328 | Pentazocine 50mg capsules |
| 354 | Morphine sulfate 100mg/50ml solution for infusion vials |
| 423 | Pethidine 50mg/ml injection |
| 458 | Morphine 15mg Suppository |
| 462 | Codeine 60mg/1ml solution for injection ampoules |
| 495 | MST Continus 10mg tablets (Napp Pharmaceuticals Ltd) |
| 607 | MST Continus Suspension 20mg granules sachets (Napp Pharmaceuticals Ltd) |
| 617 | Fentanyl 50microgram/ml Injection |
| 620 | Fentanyl 25micrograms/hour transdermal patches |
| 635 | Codeine 30mg/1ml solution for injection ampoules |
| 655 | Morphine sulfate 10mg/5ml oral solution unit dose vials sugar free |
| 659 | Morphine 30mg Suppository |
| 687 | Tramacet 37.5mg/325mg tablets (Grunenthal Ltd) |
| 701 | Tramadol 50mg modified-release capsules |
| 715 | Morphine sulphate 1mg/ml Injection |
| 748 | Durogesic 25micrograms transdermal patches (Janssen-Cilag Ltd) |
| 757 | Fentanyl 50micrograms/hour transdermal patches |
| 826 | Pethidine 50mg/1ml solution for injection ampoules |
| 1503 | Oramorph 10mg/5ml oral solution (Boehringer Ingelheim Ltd) |
| 1616 | Migraleve Pink tablets (McNeil Products Ltd) |
| 2041 | Dihydrocodeine 60mg modified-release tablets |
| 2367 | Pentazocine 25mg tablets |
| 2450 | Pethidine 50mg tablets |
| 2957 | MST Continus 30mg tablets (Napp Pharmaceuticals Ltd) |
| 2966 | Pethidine 50mg/ml injection |
| 2997 | Oramorph sr 10mg Tablet (Boehringer Ingelheim Ltd) |
| 3165 | Diamorphine 10mg Injection (Manufacturer unknown) |
| 3239 | Meptazinol 200mg tablets |
| 3378 | Tramadol 50mg soluble tablets sugar free |
| 3644 | Zydol SR 100mg tablets (Grunenthal Ltd) |
| 3653 | Dihydrocodeine 50mg/1ml solution for injection ampoules |
| 3698 | Df118 40mg Tablet (Martindale Pharmaceuticals Ltd) |
| 3919 | Sevredol 10mg tablets (Napp Pharmaceuticals Ltd) |
| 3990 | Dextromoramide 5mg tablets |
| 4114 | Tramadol 100mg modified-release capsules |
| 4115 | Tramadol 100mg modified-release tablets |
| 4236 | Palfium 5mg tablets (Roche Products Ltd) |
| 4266 | Morphine 10mg tablets |
| 4280 | MST Continus 5mg tablets (Napp Pharmaceuticals Ltd) |
| 4476 | MST Continus Suspension 60mg granules sachets (Napp Pharmaceuticals Ltd) |
| 4477 | MST Continus 60mg tablets (Napp Pharmaceuticals Ltd) |
| 4691 | Fentanyl 100micrograms/hour transdermal patches |
| 4693 | Oramorph 10mg/5ml oral solution unit dose vials (Boehringer Ingelheim Ltd) |
| 4823 | Dihydrocodeine 40mg tablets |
| 4834 | Tramadol 150mg modified-release capsules |
| 4999 | Tramadol 24 Modified-release tablet |
| 5028 | Tramadol 24 Modified-release tablet |
| 5048 | Durogesic 50micrograms transdermal patches (Janssen-Cilag Ltd) |
| 5079 | Diamorphine hydrochloride 5mg powder for injection solution |
| 5137 | Hydromorphone 2.6mg capsules |
| 5138 | Hydromorphone 1.3mg capsules |
| 5169 | Zydol SR 200mg tablets (Grunenthal Ltd) |
| 5257 | Tramadol 12 Modified-release tablet |
| 5555 | Sevredol 10mg/5ml oral solution (Napp Pharmaceuticals Ltd) |
| 5563 | Morphine sulphate 12 20mg Modified-release capsule |
| 5585 | Oxycodone 10mg capsules |
| 5599 | OxyContin 10mg modified-release tablets (Napp Pharmaceuticals Ltd) |
| 5651 | Fentanyl 400microgram lozenges |
| 5652 | Morphine sulphate 12 50mg Modified-release capsule |
| 5657 | Durogesic 75micrograms transdermal patches (Janssen-Cilag Ltd) |
| 5664 | Morphine hydrochloride 15mg suppositories |
| 5668 | Diamorphine hydrochloride 30mg powder for injection solution |
| 5670 | Diamorphine hydrochloride 10mg powder for injection solution |
| 5681 | Morphine 10mg modified-release tablets |
| 5696 | Fentanyl 600microgram lozenges |
| 5697 | Fentanyl 800microgram lozenges |
| 5714 | MST Continus 15mg tablets (Napp Pharmaceuticals Ltd) |
| 5833 | Cyclimorph 10 solution for injection 1ml ampoules (AMCo) |
| 5840 | Morphine sulfate 10mg/5ml oral solution |
| 5843 | Oxycodone 10mg modified-release tablets |
| 5936 | Transtec 35micrograms/hour transdermal patches (Napp Pharmaceuticals Ltd) |
| 5991 | MST Continus 100mg tablets (Napp Pharmaceuticals Ltd) |
| 6002 | Morphine 10mg modified-release capsules |
| 6040 | Transtec 52.5micrograms/hour transdermal patches (Napp Pharmaceuticals Ltd) |
| 6153 | Zydol SR 150mg tablets (Grunenthal Ltd) |
| 6181 | Transtec 70micrograms/hour transdermal patches (Napp Pharmaceuticals Ltd) |
| 6215 | Tramadol 200mg modified-release capsules |
| 6232 | Sevredol 20mg tablets (Napp Pharmaceuticals Ltd) |
| 6234 | Dihydrocodeine 120mg modified-release tablets |
| 6269 | Morphine sulfate 20mg/ml oral solution sugar free |
| 6298 | Fentanyl 75micrograms/hour transdermal patches |
| 6366 | Sevredol 50mg tablets (Napp Pharmaceuticals Ltd) |
| 6414 | Oxycodone hydrochloride 10mg/ml injection |
| 6458 | Diamorphine hydrochloride 500mg powder for injection solution |
| 6459 | Diamorphine hydrochloride 100mg powder for injection solution |
| 6557 | OxyNorm 5mg capsules (Napp Pharmaceuticals Ltd) |
| 6558 | Tramadol 37.5mg / Paracetamol 325mg tablets |
| 6608 | Oxycodone 20mg modified-release tablets |
| 6609 | Oxycodone 5mg/5ml oral solution sugar free |
| 6708 | Oxycodone 40mg modified-release tablets |
| 6736 | Morphine 20mg modified-release granules sachets sugar free |
| 6769 | Oxycodone 5mg modified-release tablets |
| 6790 | Oxycodone 5mg capsules |
| 6879 | Buprenorphine 35micrograms/hour transdermal patches |
| 6892 | Morphine sulphate 10mg/ml Injection |
| 6917 | Buprenorphine 52.5micrograms/hour transdermal patches |
| 6948 | Oxycodone 80mg modified-release tablets |
| 7082 | Durogesic DTrans 25micrograms transdermal patches (Janssen-Cilag Ltd) |
| 7107 | Durogesic DTrans 50micrograms transdermal patches (Janssen-Cilag Ltd) |
| 7114 | Diamorphine 3mg/5ml oral solution |
| 7126 | Fentanyl 12micrograms/hour transdermal patches |
| 7167 | OxyContin 5mg modified-release tablets (Napp Pharmaceuticals Ltd) |
| 7197 | Morphine sulphate 12 30mg Modified-release capsule |
| 7236 | Buprenorphine 10micrograms/hour transdermal patches |
| 7238 | Buprenorphine 20micrograms/hour transdermal patches |
| 7275 | Oxycodone 20mg capsules |
| 7334 | Buprenorphine 5micrograms/hour transdermal patches |
| 7372 | OxyNorm 20mg capsules (Napp Pharmaceuticals Ltd) |
| 7389 | OxyContin 20mg modified-release tablets (Napp Pharmaceuticals Ltd) |
| 7397 | Durogesic DTrans 75micrograms transdermal patches (Janssen-Cilag Ltd) |
| 7406 | OxyNorm 10mg/ml concentrate oral solution (Napp Pharmaceuticals Ltd) |
| 7457 | Temgesic 0.3mg/ml Injection (Reckitt Benckiser Healthcare (UK) Ltd) |
| 7469 | Df118 10mg/5ml Oral solution (Martindale Pharmaceuticals Ltd) |
| 7517 | Morphine sulfate 15mg suppositories |
| 7555 | BuTrans 5micrograms/hour transdermal patches (Napp Pharmaceuticals Ltd) |
| 7729 | Morphine hcl Oral solution (Thornton and Ross Ltd) |
| 7800 | Palfium 5mg/ml Injection (Roche Products Ltd) |
| 7849 | Diamorphine 100mg Injection (Manufacturer unknown) |
| 7875 | Morphine 30mg modified-release tablets |
| 7999 | Diamorphine 5mg Injection (Manufacturer unknown) |
| 8039 | MST Continus 200mg tablets (Napp Pharmaceuticals Ltd) |
| 8040 | Diamorphine 30mg Injection (Manufacturer unknown) |
| 8075 | Morphine sulphate 30mg/ml Injection |
| 8375 | Fortral 25mg tablets (Zentiva) |
| 8416 | Tramadol 12 Modified-release tablet |
| 8447 | Meptid 200mg Tablet (Shire Pharmaceuticals Ltd) |
| 8456 | DHC Continus 60mg tablets (Napp Pharmaceuticals Ltd) |
| 8735 | Diamorphine 5mg/5ml Oral solution (Manufacturer unknown) |
| 8740 | Morphine hydrochloride 30mg suppositories |
| 8822 | Morphine 60mg modified-release tablets |
| 8866 | Diamorphine 10mg Tablet (Manufacturer unknown) |
| 8876 | Oramorph 20mg/ml concentrated oral solution (Boehringer Ingelheim Ltd) |
| 9001 | Diconal tablets (Amdipharm Plc) |
| 9012 | Cyclimorph 15 solution for injection 1ml ampoules (AMCo) |
| 9053 | Diamorphine 500mg Injection (Manufacturer unknown) |
| 9137 | Morphine 20mg tablets |
| 9183 | Morphine 100mg modified-release tablets |
| 9209 | DHC Continus 90mg tablets (Napp Pharmaceuticals Ltd) |
| 9275 | DHC Continus 120mg tablets (Napp Pharmaceuticals Ltd) |
| 9313 | Dihydrocodeine 90mg modified-release tablets |
| 9325 | Hydromorphone 4mg modified-release capsules |
| 9330 | Palladone 2.6mg capsules (Napp Pharmaceuticals Ltd) |
| 9331 | Palladone SR 4mg capsules (Napp Pharmaceuticals Ltd) |
| 9332 | Palladone SR 2mg capsules (Napp Pharmaceuticals Ltd) |
| 9337 | MXL 30mg capsules (Napp Pharmaceuticals Ltd) |
| 9342 | MXL 60mg capsules (Napp Pharmaceuticals Ltd) |
| 9371 | MXL 120mg capsules (Napp Pharmaceuticals Ltd) |
| 9381 | MXL 90mg capsules (Napp Pharmaceuticals Ltd) |
| 9389 | Zamadol SR 50mg capsules (Meda Pharmaceuticals Ltd) |
| 9396 | Zamadol SR 100mg capsules (Meda Pharmaceuticals Ltd) |
| 9484 | Morphine sulphate 24 60mg Modified-release capsule |
| 9557 | Morphine 15mg modified-release tablets |
| 9602 | Morphine 5mg modified-release tablets |
| 9615 | Palladone 1.3mg capsules (Napp Pharmaceuticals Ltd) |
| 9672 | Morphine 100mg modified-release granules sachets sugar free |
| 9874 | OxyNorm liquid 5mg/5ml oral solution (Napp Pharmaceuticals Ltd) |
| 9927 | OxyContin 40mg modified-release tablets (Napp Pharmaceuticals Ltd) |
| 9928 | Oxynorm 10mg/ml Solution for injection (Napp Pharmaceuticals Ltd) |
| 9945 | Diamorphine 10mg Tablet (Aurum Pharmaceuticals Ltd) |
| 9960 | Morphine sulphate 12 60mg Modified-release capsule |
| 9973 | OxyNorm 10mg capsules (Napp Pharmaceuticals Ltd) |
| 10021 | OxyContin 80mg modified-release tablets (Napp Pharmaceuticals Ltd) |
| 10205 | BuTrans 10micrograms/hour transdermal patches (Napp Pharmaceuticals Ltd) |
| 10239 | MXL 150mg capsules (Napp Pharmaceuticals Ltd) |
| 10309 | Df118 50mg/ml Injection (Martindale Pharmaceuticals Ltd) |
| 10583 | Pentazocine 30mg/ml injection |
| 10631 | Morphine 10mg/ml Tincture |
| 10730 | Sevredol 10mg Suppository (Napp Pharmaceuticals Ltd) |
| 10769 | Fortral 50mg Capsule (Sanofi-Synthelabo Ltd) |
| 10922 | Durogesic 100micrograms transdermal patches (Janssen-Cilag Ltd) |
| 10925 | Meptid 100mg/1ml solution for injection ampoules (Almirall Ltd) |
| 11101 | Zydol 50mg soluble tablets (Grunenthal Ltd) |
| 11129 | Papaveretum 15.4mg/1ml solution for injection ampoules |
| 11275 | Zydol 100mg/2ml solution for injection ampoules (Grunenthal Ltd) |
| 11342 | Oramorph 30mg/5ml oral solution unit dose vials (Boehringer Ingelheim Ltd) |
| 11405 | Oxycodone 10mg/ml oral solution sugar free |
| 11471 | Tramadol 100mg/2ml solution for injection ampoules |
| 11549 | Tramadol 75mg modified-release tablets |
| 11554 | Ibuprofen 200mg / Codeine 12.8mg tablets |
| 11584 | Buprenorphine 70micrograms/hour transdermal patches |
| 11698 | Morphine sulphate 24 30mg Modified-release capsule |
| 11734 | Tramadol 50mg orodispersible tablets sugar free |
| 11746 | Tramadol 300mg modified-release tablets |
| 11748 | Tramadol 400mg modified-release tablets |
| 11801 | Meptazinol 100mg/1ml solution for injection ampoules |
| 11838 | Morphine 200mg modified-release tablets |
| 11843 | Fentanyl 200microgram lozenges |
| 11971 | Morphine and Cocaine elixir |
| 11982 | Durogesic DTrans 12micrograms transdermal patches (Janssen-Cilag Ltd) |
| 12020 | Dipipanone 10mg / Cyclizine 30mg tablets |
| 12076 | Dextropropoxyphene 60mg capsules |
| 12219 | Morphine sulfate 15mg/1ml solution for injection ampoules |
| 12591 | Morphine 60mg modified-release granules sachets sugar free |
| 12602 | Morphine sulfate 10mg suppositories |
| 12604 | MST Continus Suspension 100mg granules sachets (Napp Pharmaceuticals Ltd) |
| 12709 | Ibuprofen and codeine 200mg + 12.5mg Tablet |
| 12889 | Oramorph 100mg/5ml oral solution unit dose vials (Boehringer Ingelheim Ltd) |
| 12900 | MST Continus Suspension 30mg granules sachets (Napp Pharmaceuticals Ltd) |
| 12981 | Narcan 400microgram/ml Injection (Bristol-Myers Squibb Pharmaceuticals Ltd) |
| 13076 | Actiq 200microgram lozenges with integral oromucosal applicator (Teva UK Ltd) |
| 13114 | Zomorph 10mg modified-release capsules (Ethypharm UK Ltd) |
| 13117 | Zomorph 30mg modified-release capsules (Ethypharm UK Ltd) |
| 13172 | Morphine sulfate 20mg/1ml solution for injection ampoules |
| 13225 | Morphine sulfate 30mg/1ml solution for injection ampoules |
| 13280 | Morphine sulfate 60mg/2ml solution for injection ampoules |
| 13300 | BuTrans 20micrograms/hour transdermal patches (Napp Pharmaceuticals Ltd) |
| 13420 | Diamorphine 15mg/5ml Oral solution (Manufacturer unknown) |
| 13711 | Morcap sr 20mg Modified-release capsule (Faulding Pharmaceuticals (Dbl)) |
| 13813 | Zamadol 50mg capsules (Meda Pharmaceuticals Ltd) |
| 13893 | Nurofen Plus tablets (Reckitt Benckiser Healthcare (UK) Ltd) |
| 13995 | Morphine sulphate 24 200mg Modified-release capsule |
| 13997 | Morphine sulphate 100mg Modified-release capsule |
| 14050 | Morphine sulphate 12 100mg Modified-release capsule |
| 14063 | Zomorph 100mg modified-release capsules (Ethypharm UK Ltd) |
| 14156 | Morphine sulfate 30mg/5ml oral solution unit dose vials sugar free |
| 14226 | Morphine 30mg modified-release granules sachets sugar free |
| 14394 | Phenazocine hydrobromide 5mg tablet |
| 14490 | Tramake 50mg capsules (Galen Ltd) |
| 14900 | Durogesic DTrans 100micrograms transdermal patches (Janssen-Cilag Ltd) |
| 15337 | Fentanyl 1.2mg lozenges |
| 15339 | Diamorphine 10mg/5ml oral solution |
| 15353 | Papaveretum 7.7mg/1ml solution for injection ampoules |
| 15781 | Morphine sulphate 24 90mg Modified-release capsule |
| 15792 | Hydromorphone 2mg modified-release capsules |
| 15793 | Diamorphine hydrochloride powder |
| 15798 | Hydromorphone 8mg modified-release capsules |
| 15815 | Morphine 50mg tablets |
| 15950 | Zomorph 200mg modified-release capsules (Ethypharm UK Ltd) |
| 15964 | Zomorph 60mg modified-release capsules (Ethypharm UK Ltd) |
| 16189 | Morphine sulphate 10mg/ml Injection |
| 16271 | Zydol XL 300mg tablets (Grunenthal Ltd) |
| 16273 | Oramorph sr 30mg Tablet (Boehringer Ingelheim Ltd) |
| 16335 | Morphine tartrate 10mg/1ml / Cyclizine tartrate 50mg/1ml solution for injection ampoules |
| 16395 | Zydol XL 200mg tablets (Grunenthal Ltd) |
| 16618 | Tilofyl 75micrograms/hour transdermal patches (Tillomed Laboratories Ltd) |
| 16803 | Naloxone hc 400microgram/ml Injection |
| 16964 | Nubain 10mg/ml Injection (Bristol-Myers Squibb Pharmaceuticals Ltd) |
| 17043 | Pethidine 100mg/2ml solution for injection ampoules |
| 17167 | Narphen 5mg Tablet (Smith & Nephew Healthcare Ltd) |
| 17386 | Pethidine 50mg Tablet (Roche Products Ltd) |
| 17398 | Morphine tartrate 15mg/1ml / Cyclizine tartrate 50mg/1ml solution for injection ampoules |
| 17490 | Cyclizine tartrate with morphine tartrate 50mg+15mg/ml injection |
| 17734 | Dextromoramide 5mg/ml injection |
| 17893 | Oramorph sr 60mg Tablet (Boehringer Ingelheim Ltd) |
| 17936 | MXL 200mg capsules (Napp Pharmaceuticals Ltd) |
| 17943 | Sevredol 20mg/ml concentrated oral solution (Napp Pharmaceuticals Ltd) |
| 18166 | Morphine sulphate 12 200mg Modified-release capsule |
| 18174 | Actiq 400microgram lozenges with integral oromucosal applicator (Teva UK Ltd) |
| 18468 | J Collis Browne's mixture (Thornton & Ross Ltd) |
| 18491 | Pamergan P100 solution for injection 2ml ampoules (Martindale Pharmaceuticals Ltd) |
| 18656 | SRM-RHOTARD 10mg Modified-release tablet (Pharmacia Ltd) |
| 18700 | SRM-RHOTARD 30mg Modified-release tablet (Pharmacia Ltd) |
| 18734 | Oramorph sr 100mg Tablet (Boehringer Ingelheim Ltd) |
| 18792 | Diamorphine 10mg tablets |
| 18801 | Morcap SR 100mg capsules (Hospira UK Ltd) |
| 18881 | Morphgesic SR 10mg tablets (AMCo) |
| 19092 | Morcap sr 50mg Modified-release capsule (Faulding Pharmaceuticals (Dbl)) |
| 19116 | Pethidine 100mg/2ml / Promethazine 50mg/2ml solution for injection ampoules |
| 19119 | Nalbuphine hc 10mg/ml Injection |
| 19291 | Morphine sulfate 100mg/5ml oral solution unit dose vials sugar free |
| 19317 | Omnopon 10mg Tablet (Roche Products Ltd) |
| 19449 | Morphgesic SR 30mg tablets (AMCo) |
| 19471 | Morphgesic SR 60mg tablets (AMCo) |
| 19477 | Morphgesic SR 100mg tablets (AMCo) |
| 19764 | Papaveretum 10mg tablet |
| 19954 | Palladone SR 16mg capsules (Napp Pharmaceuticals Ltd) |
| 19972 | Hydromorphone 16mg modified-release capsules |
| 19993 | Dromadol SR 100mg tablets (Teva UK Ltd) |
| 20005 | Morphine sulfate 10mg/10ml solution for injection pre-filled syringes |
| 20039 | Dromoran roche 1.5mg Tablet (Roche Products Ltd) |
| 20310 | Zamadol Melt 50mg tablets (Meda Pharmaceuticals Ltd) |
| 20783 | Morphine sulfate 30mg suppositories |
| 20815 | Morphine sulfate 20mg suppositories |
| 21251 | Ultramol Soluble tablets (Zentiva) |
| 21275 | Palladone SR 8mg capsules (Napp Pharmaceuticals Ltd) |
| 21285 | Palladone SR 24mg capsules (Napp Pharmaceuticals Ltd) |
| 21397 | Zydol XL 400mg tablets (Grunenthal Ltd) |
| 21777 | Dromadol SR 200mg tablets (Teva UK Ltd) |
| 21797 | Zamadol SR 200mg capsules (Meda Pharmaceuticals Ltd) |
| 21868 | Diamorphine hydrochloride and cocaine oral solution |
| 21947 | Zydol XL 150mg tablets (Grunenthal Ltd) |
| 22024 | Rhotard Morphine SR 10mg tablets (Sovereign Medical Ltd) |
| 22026 | Rhotard Morphine SR 30mg tablets (Sovereign Medical Ltd) |
| 22066 | Tilofyl 50micrograms/hour transdermal patches (Tillomed Laboratories Ltd) |
| 22571 | Morphine sulphate and atropine 10mg + 600microgram/ml Injection |
| 22690 | Morphine sulphate 24 120mg Modified-release capsule |
| 22756 | Filnarine SR 30mg tablets (Teva UK Ltd) |
| 23060 | MST Continus Suspension 200mg granules sachets (Napp Pharmaceuticals Ltd) |
| 23063 | Morphine 1mg/5ml / Peppermint oil 1.5microlitres/5ml oral solution |
| 23375 | Palfium 10mg Suppository (Roche Products Ltd) |
| 23442 | Pethidine 50mg/5ml solution for injection ampoules |
| 23625 | Dromadol SR 150mg tablets (Teva UK Ltd) |
| 23906 | Alfentanil 5mg/1ml solution for injection ampoules |
| 23981 | Zamadol SR 150mg capsules (Meda Pharmaceuticals Ltd) |
| 24108 | Diagesil 10mg Injection (Berk Pharmaceuticals Ltd) |
| 24453 | Morcap sr 100mg Modified-release capsule (Faulding Pharmaceuticals (Dbl)) |
| 24640 | Diaphine 100mg Injection (Napp Pharmaceuticals Ltd) |
| 24736 | Hydromorphone 24mg modified-release capsules |
| 24790 | Alfentanil 500micrograms/ml injection |
| 24830 | Morphine sulphate 20mg/ml Injection |
| 24867 | Pethidine with levallorphan tartrate injection |
| 24986 | Fentanyl 1.6mg lozenges |
| 25185 | Sublimaze 50microgram/ml Injection (Janssen-Cilag Ltd) |
| 25199 | Actiq 600microgram lozenges with integral oromucosal applicator (Teva UK Ltd) |
| 25316 | Cyclizine tartrate with morphine tartrate 50mg+10mg/ml injection |
| 25481 | Nepenthe 8.4mg/ml Injection (Celltech Pharma Europe Ltd) |
| 25611 | Palfium 10mg/ml Injection (Roche Products Ltd) |
| 25979 | Doloxene 60mg Capsule (Eli Lilly and Company Ltd) |
| 26021 | Actiq 1.6mg lozenges with integral oromucosal applicator (Teva UK Ltd) |
| 26115 | Omnopon-scopolamine Injection (Roche Products Ltd) |
| 26283 | Filnarine SR 10mg tablets (Teva UK Ltd) |
| 26284 | Filnarine SR 100mg tablets (Teva UK Ltd) |
| 26336 | Dromadol XL 300mg tablets (IVAX Pharmaceuticals UK Ltd) |
| 26908 | Actiq 800microgram lozenges with integral oromucosal applicator (Teva UK Ltd) |
| 26986 | Zamadol 24hr 200mg modified-release tablets (Meda Pharmaceuticals Ltd) |
| 27058 | Filnarine SR 60mg tablets (Teva UK Ltd) |
| 27591 | Zamadol 24hr 150mg modified-release tablets (Meda Pharmaceuticals Ltd) |
| 27749 | Morphine sulphate 24 150mg Modified-release capsule |
| 28143 | Naloxone hc 400microgram/ml Injection |
| 28189 | Tilofyl 100micrograms/hour transdermal patches (Tillomed Laboratories Ltd) |
| 28421 | Moraxen 35mg Rectal tampon (Schwarz Pharma Ltd) |
| 28567 | Rapifen 100microgram/ml Paediatric injection (Janssen-Cilag Ltd) |
| 28711 | Diamorphine hydrochloride bpc 1973 3mg/5ml oral solution |
| 28728 | Zamadol 24hr 300mg modified-release tablets (Meda Pharmaceuticals Ltd) |
| 28732 | Papaveretum with hyoscine 7.7mg with 400 micrograms/ml injection |
| 28805 | Dextromoramide 10mg suppository |
| 29014 | Diaphine 10mg Injection (Napp Pharmaceuticals Ltd) |
| 29020 | Morphine 200mg modified-release granules sachets sugar free |
| 29426 | Pethidine 50mg/ml Injection (Martindale Pharmaceuticals Ltd) |
| 29500 | Diamorphine 5mg/5ml oral solution |
| 29577 | Actiq 1.2mg lozenges with integral oromucosal applicator (Teva UK Ltd) |
| 29860 | Tramadol 50mg capsules (IVAX Pharmaceuticals UK Ltd) |
| 29898 | Morphine sulfate powder |
| 30049 | Morphine sulphate rapiject 1mg/ml Injection (International Medication Systems (UK) Ltd) |
| 30252 | Morphine 8.4mg/ml elixir |
| 30514 | Diaphine 30mg Injection (Napp Pharmaceuticals Ltd) |
| 30633 | Levorphanol 1.5mg Tablet |
| 30698 | Diagesil 500mg Injection (Berk Pharmaceuticals Ltd) |
| 30761 | Diamorphine 10mg/5ml Oral solution (Manufacturer unknown) |
| 31033 | Diamorphine hydrochloride 3mg/5ml oral solution |
| 31053 | Tilofyl 25micrograms/hour transdermal patches (Tillomed Laboratories Ltd) |
| 31105 | Dromadol XL 200mg tablets (IVAX Pharmaceuticals UK Ltd) |
| 31107 | Dromadol XL 150mg tablets (IVAX Pharmaceuticals UK Ltd) |
| 31253 | Pethidine 50mg/ml Injection (Auden McKenzie (Pharma Division) Ltd) |
| 31582 | Pentazocine 30mg/1ml solution for injection ampoules |
| 31584 | Pentazocine 60mg/2ml solution for injection ampoules |
| 31650 | Morphine sulfate 30mg/30ml solution for infusion vials |
| 31734 | Dromadol XL 400mg tablets (IVAX Pharmaceuticals UK Ltd) |
| 31885 | Pethidine 100mg/10ml solution for injection ampoules |
| 31935 | Pethidine 50mg Tablet (Roche Products Ltd) |
| 31960 | Diamorphine 15mg/5ml oral solution |
| 32165 | Tramadol 50mg Capsule (Generics (UK) Ltd) |
| 32381 | Rapifen 5mg/ml Concentrate for solution for infusion (Janssen-Cilag Ltd) |
| 32425 | OxyNorm 10mg/1ml solution for injection ampoules (Napp Pharmaceuticals Ltd) |
| 32450 | Zamadol 24hr 400mg modified-release tablets (Meda Pharmaceuticals Ltd) |
| 32688 | Diagesil 5mg Injection (Berk Pharmaceuticals Ltd) |
| 32831 | Pethidine 100mg/2ml Injection (Roche Products Ltd) |
| 32897 | Diamorphine 5mg powder for solution for injection ampoules (Novartis Vaccines and Diagnostics Ltd) |
| 33654 | Dihydrocodeine 30mg tablets (Wockhardt UK Ltd) |
| 33954 | Promethazine hydrochloride 50mg with pethidine 100mg/2ml injection |
| 34008 | Dihydrocodeine 30mg tablets (IVAX Pharmaceuticals UK Ltd) |
| 34065 | Tramadol sr 150mg Modified-release tablet (Winthrop Pharmaceuticals Ltd) |
| 34260 | Tramadol sr 100mg Modified-release tablet (Winthrop Pharmaceuticals Ltd) |
| 34281 | Tramadol sr 200mg Modified-release tablet (Winthrop Pharmaceuticals Ltd) |
| 34422 | Tramadol 50mg capsules (Actavis UK Ltd) |
| 34440 | Dihydrocodeine 30mg tablets (A A H Pharmaceuticals Ltd) |
| 34477 | Morphine sulfate 10mg/5ml oral solution (Martindale Pharmaceuticals Ltd) |
| 34489 | Diamorphine hydrochloride 10mg Injection (Hillcross Pharmaceuticals Ltd) |
| 34521 | Tramadol 50mg capsules (A A H Pharmaceuticals Ltd) |
| 34570 | Tramadol 50mg capsules (Teva UK Ltd) |
| 34579 | Dihydrocodeine 30mg tablets (Actavis UK Ltd) |
| 34639 | Tramadol 50mg capsules (Genus Pharmaceuticals Ltd) |
| 34662 | Dihydrocodeine 30mg tablets (Mylan) |
| 34730 | Dihydrocodeine 30mg Tablet (Berk Pharmaceuticals Ltd) |
| 34771 | Morphine sulphate 30mg/ml Injection (Celltech Pharma Europe Ltd) |
| 34786 | Diamorphine 10mg powder for solution for injection ampoules (Novartis Vaccines and Diagnostics Ltd) |
| 34787 | Diamorphine 30mg powder for solution for injection ampoules (Novartis Vaccines and Diagnostics Ltd) |
| 34808 | Tramadol 50mg capsules (PLIVA Pharma Ltd) |
| 35038 | OxyNorm 20mg/2ml solution for injection ampoules (Napp Pharmaceuticals Ltd) |
| 35085 | Oxycodone 20mg/2ml solution for injection ampoules |
| 35093 | Morphine sulfate 50mg/50ml solution for infusion vials |
| 35330 | Fentanyl 100micrograms/2ml solution for injection ampoules |
| 35341 | Oxycodone 10mg/1ml solution for injection ampoules |
| 35347 | Tramadol 24 Modified-release tablet |
| 35438 | Tramquel SR 100mg capsules (Beechmere Pharmaceuticals Ltd) |
| 35500 | Alfentanil 1mg/2ml solution for injection ampoules |
| 35651 | Tradorec XL 200mg tablets (Endo Ventures Ltd) |
| 35656 | Tradorec XL 100mg tablets (Endo Ventures Ltd) |
| 35798 | Min-i-jet naloxone 400microgram/ml Injection (Celltech Pharma Europe Ltd) |
| 35806 | Larapam SR 100mg tablets (Sandoz Ltd) |
| 35853 | Fentanyl 500micrograms/10ml solution for injection ampoules |
| 35968 | Matrifen 25micrograms/hour transdermal patches (Teva UK Ltd) |
| 36035 | Tradorec XL 300mg tablets (Endo Ventures Ltd) |
| 36040 | Matrifen 100micrograms/hour transdermal patches (Teva UK Ltd) |
| 36185 | Alfentanil 5mg/10ml solution for injection ampoules |
| 36211 | Matrifen 50micrograms/hour transdermal patches (Teva UK Ltd) |
| 36697 | Mabron 200mg modified-release tablets (Morningside Healthcare Ltd) |
| 36732 | Tramadol 50mg modified-release tablets |
| 36873 | Zydol SR 50mg tablets (Grunenthal Ltd) |
| 36949 | Tramquel SR 50mg capsules (Beechmere Pharmaceuticals Ltd) |
| 37020 | Tramadol 150mg modified-release tablets |
| 37021 | Tramadol 200mg modified-release tablets |
| 37251 | Alfentanil 0.1% nasal spray |
| 37703 | Pethidine 50mg/1ml solution for injection ampoules (AMCo) |
| 37719 | Fentalis Reservoir 100micrograms/hour transdermal patches (Sandoz Ltd) |
| 37779 | Fentalis Reservoir 25micrograms/hour transdermal patches (Sandoz Ltd) |
| 37831 | Mabron 100mg modified-release tablets (Morningside Healthcare Ltd) |
| 37867 | Tramadol (roi) Tablet |
| 37923 | Fentalis Reservoir 50micrograms/hour transdermal patches (Sandoz Ltd) |
| 37928 | Matrifen 12micrograms/hour transdermal patches (Teva UK Ltd) |
| 37954 | Mezolar Matrix 12micrograms/hour transdermal patches (Sandoz Ltd) |
| 37960 | Osmach 50micrograms/hour transdermal patches (Teva UK Ltd) |
| 37968 | Fentanyl 40micrograms/dose transdermal system |
| 38013 | Pethidine 50mg capsules |
| 38031 | Mezolar Matrix 25micrograms/hour transdermal patches (Sandoz Ltd) |
| 38092 | Pentazocine 30mg/ml Injection (Sterwin Medicines) |
| 38103 | Pethidine 25mg Tablet (Roche Products Ltd) |
| 38183 | Remifentanil 1mg powder for solution for injection vials |
| 38196 | Larapam SR 200mg tablets (Sandoz Ltd) |
| 38301 | Cyclizine 30mg with dipipanone 10mg tablets |
| 38326 | Mezolar Matrix 75micrograms/hour transdermal patches (Sandoz Ltd) |
| 38351 | Matrifen 75micrograms/hour transdermal patches (Teva UK Ltd) |
| 38365 | Fentalis Reservoir 75micrograms/hour transdermal patches (Sandoz Ltd) |
| 38521 | Dihydrocodeine 30mg tablets (Teva UK Ltd) |
| 38524 | Rapifen 1mg/2ml solution for injection ampoules (Piramal Critical Care Ltd) |
| 38528 | Tramadol 50mg Capsule (Tillomed Laboratories Ltd) |
| 38553 | Mezolar Matrix 100micrograms/hour transdermal patches (Sandoz Ltd) |
| 38874 | Zamadol 100mg/2ml solution for injection ampoules (Meda Pharmaceuticals Ltd) |
| 38956 | Tramquel SR 200mg capsules (Beechmere Pharmaceuticals Ltd) |
| 38970 | DF 118 Forte 40mg tablets (Martindale Pharmaceuticals Ltd) |
| 39084 | Osmach 100micrograms/hour transdermal patches (Ratiopharm UK Ltd) |
| 39180 | Mezolar Matrix 50micrograms/hour transdermal patches (Sandoz Ltd) |
| 39251 | Osmach 25micrograms/hour transdermal patches (Teva UK Ltd) |
| 39419 | Palfium 5mg Tablet (IDIS World Medicines) |
| 39461 | Solpadeine Migraine Ibuprofen & Codeine tablets (Omega Pharma Ltd) |
| 39469 | Fentanyl 100microgram sublingual tablets sugar free |
| 39475 | Oxycodone 10mg / Naloxone 5mg modified-release tablets |
| 39477 | Targinact 10mg/5mg modified-release tablets (Napp Pharmaceuticals Ltd) |
| 39478 | Targinact 20mg/10mg modified-release tablets (Napp Pharmaceuticals Ltd) |
| 39498 | Oxycodone 20mg / Naloxone 10mg modified-release tablets |
| 39505 | Marol 100mg modified-release tablets (Morningside Healthcare Ltd) |
| 39518 | Abstral 100microgram sublingual tablets (Kyowa Kirin Ltd) |
| 39558 | Dihydrocodeine 30mg tablets (Zentiva) |
| 39590 | Fentanyl 200microgram sublingual tablets sugar free |
| 39709 | Marol 200mg modified-release tablets (Morningside Healthcare Ltd) |
| 39723 | Fentanyl 100microgram buccal tablets sugar free |
| 39746 | Effentora 100microgram buccal tablets (Teva UK Ltd) |
| 39750 | Marol 150mg modified-release tablets (Morningside Healthcare Ltd) |
| 39756 | Abstral 200microgram sublingual tablets (Kyowa Kirin Ltd) |
| 39798 | Nobligan retard 100mg tablets (Grunenthal Ltd) |
| 39799 | Abstral 800microgram sublingual tablets (Kyowa Kirin Ltd) |
| 39811 | Maxitram SR 200mg capsules (Chiesi Ltd) |
| 39842 | Meptid 200mg tablets (Almirall Ltd) |
| 39929 | Effentora 200microgram buccal tablets (Teva UK Ltd) |
| 39987 | Abstral 400microgram sublingual tablets (Kyowa Kirin Ltd) |
| 40018 | Fentanyl 200microgram buccal tablets sugar free |
| 40058 | Tramulief SR 100mg tablets (AMCo) |
| 40060 | Tramulief SR 200mg tablets (AMCo) |
| 40061 | Tramulief SR 150mg tablets (AMCo) |
| 40098 | Fentanyl 400microgram sublingual tablets sugar free |
| 40128 | Effentora 400microgram buccal tablets (Teva UK Ltd) |
| 40159 | Dihydrocodeine 10mg/5ml oral solution (Martindale Pharmaceuticals Ltd) |
| 40166 | Tramadol 50mg capsules (Niche Generics Ltd) |
| 40239 | Pethidine 50mg tablets (Martindale Pharmaceuticals Ltd) |
| 40249 | Maxitram SR 100mg capsules (Chiesi Ltd) |
| 40254 | Maxitram SR 50mg capsules (Chiesi Ltd) |
| 40427 | Rapifen 500microgram/ml Injection (Janssen-Cilag Ltd) |
| 40434 | Effentora 600microgram buccal tablets (Teva UK Ltd) |
| 40508 | Abstral 600microgram sublingual tablets (Kyowa Kirin Ltd) |
| 40563 | Morphine 30mg modified-release capsules |
| 40576 | Fentanyl 400microgram buccal tablets sugar free |
| 40616 | Oxycodone 5mg / Naloxone 2.5mg modified-release tablets |
| 40645 | Targinact 5mg/2.5mg modified-release tablets (Napp Pharmaceuticals Ltd) |
| 40688 | Oxycodone 50mg/1ml solution for injection ampoules |
| 40718 | Tramadol 50mg capsules (Almus Pharmaceuticals Ltd) |
| 40752 | OxyNorm 50mg/1ml solution for injection ampoules (Napp Pharmaceuticals Ltd) |
| 40785 | Oxycodone 40mg / Naloxone 20mg modified-release tablets |
| 40805 | Tramquel SR 150mg capsules (Beechmere Pharmaceuticals Ltd) |
| 40883 | Maxitram SR 150mg capsules (Chiesi Ltd) |
| 40926 | Larapam SR 150mg tablets (Sandoz Ltd) |
| 40940 | Fentanyl 300microgram sublingual tablets sugar free |
| 40957 | Effentora 800microgram buccal tablets (Teva UK Ltd) |
| 40961 | Targinact 40mg/20mg modified-release tablets (Napp Pharmaceuticals Ltd) |
| 41135 | Fentanyl 50micrograms/dose nasal spray |
| 41161 | Osmach 75micrograms/hour transdermal patches (Teva UK Ltd) |
| 41286 | Abstral 300microgram sublingual tablets (Kyowa Kirin Ltd) |
| 41348 | Fentanyl 600microgram sublingual tablets sugar free |
| 41550 | Pethidine 100mg/2ml Injection (C P Pharmaceuticals Ltd) |
| 41668 | Morphine hcl 15mg Suppository (Martindale Pharmaceuticals Ltd) |
| 41673 | Morphine sulphate 10mg Suppository (Aurum Pharmaceuticals Ltd) |
| 41674 | Morphine sulphate 15mg Suppository (Celltech Pharma Europe Ltd) |
| 41722 | Diamorphine 30mg powder for solution for injection ampoules (Wockhardt UK Ltd) |
| 41974 | Morphine sulfate 10mg/1ml solution for injection ampoules (Martindale Pharmaceuticals Ltd) |
| 41976 | Tramadol 100mg modified-release tablets (A A H Pharmaceuticals Ltd) |
| 42021 | Victanyl 100micrograms/hour transdermal patches (Actavis UK Ltd) |
| 42094 | Fortral 30mg/1ml solution for injection ampoules (Zentiva) |
| 42208 | Df118 30mg Tablet (Martindale Pharmaceuticals Ltd) |
| 42280 | Tramadol 37.5mg / Paracetamol 325mg effervescent tablets sugar free |
| 42332 | Tramacet 37.5mg/325mg effervescent tablets (Grunenthal Ltd) |
| 42380 | Morphine sulphate 10mg Modified-release capsule |
| 42399 | Fentanyl 800microgram sublingual tablets sugar free |
| 42538 | Fentanyl 600microgram buccal tablets sugar free |
| 42576 | Victanyl 75micrograms/hour transdermal patches (Actavis UK Ltd) |
| 42590 | Osmanil 75micrograms/hour transdermal patches (Zentiva) |
| 42591 | Osmanil 25micrograms/hour transdermal patches (Zentiva) |
| 42708 | Pethidine 50mg/ml intramuscular injection (Roche Products Ltd) |
| 42798 | Tramadol 150mg modified-release tablets (A A H Pharmaceuticals Ltd) |
| 42913 | Diamorphine 10mg powder for solution for injection ampoules (Wockhardt UK Ltd) |
| 43089 | Instanyl 100micrograms/dose nasal spray (Takeda UK Ltd) |
| 43152 | Osmanil 50micrograms/hour transdermal patches (Zentiva) |
| 43198 | Tramadol sr 50mg Capsule (Hillcross Pharmaceuticals Ltd) |
| 43260 | Methadone Oral solution |
| 43315 | Morphine sulfate 10mg/1ml suspension for injection vials |
| 43384 | Rapifen Intensive Care 5mg/1ml solution for injection ampoules (Piramal Critical Care Ltd) |
| 43504 | Codeine phosphate Oral solution (William Ransom) |
| 43513 | Tramadol 50mg capsules (Zentiva) |
| 43617 | Instanyl 200micrograms/dose nasal spray (Takeda UK Ltd) |
| 43652 | Morphine 100mg modified-release capsules |
| 43657 | Morphine 200mg modified-release capsules |
| 43720 | Rapifen 5mg/10ml solution for injection ampoules (Piramal Critical Care Ltd) |
| 43812 | Sublimaze 500micrograms/10ml solution for injection ampoules (Piramal Critical Care Ltd) |
| 44371 | Mabron 150mg modified-release tablets (Morningside Healthcare Ltd) |
| 44487 | Osmanil 12micrograms/hour transdermal patches (Zentiva) |
| 44837 | Victanyl 50micrograms/hour transdermal patches (Actavis UK Ltd) |
| 44867 | Fortral 60mg/2ml solution for injection ampoules (Zentiva) |
| 45092 | Fentanyl 200micrograms/dose nasal spray |
| 45325 | Pethidine injection |
| 45439 | PecFent 100micrograms/dose nasal spray (Kyowa Kirin Ltd) |
| 45460 | Osmanil 100micrograms/hour transdermal patches (Zentiva) |
| 45549 | Victanyl 25micrograms/hour transdermal patches (Actavis UK Ltd) |
| 45598 | Instanyl 50micrograms/dose nasal spray (Takeda UK Ltd) |
| 45736 | Morphine 60mg modified-release capsules |
| 45745 | OxyContin 30mg modified-release tablets (Napp Pharmaceuticals Ltd) |
| 45766 | OxyContin 60mg modified-release tablets (Napp Pharmaceuticals Ltd) |
| 45788 | OxyContin 15mg modified-release tablets (Napp Pharmaceuticals Ltd) |
| 45790 | Oxycodone 15mg modified-release tablets |
| 45800 | Tapentadol 200mg modified-release tablets |
| 45811 | Tapentadol 50mg tablets |
| 45827 | Oxycodone 30mg modified-release tablets |
| 45830 | OxyContin 120mg modified-release tablets (Napp Pharmaceuticals Ltd) |
| 45894 | Fentanyl 100micrograms/dose nasal spray |
| 45929 | Oxycodone 60mg modified-release tablets |
| 45936 | Palexia 50mg tablets (Grunenthal Ltd) |
| 45982 | Palexia SR 50mg tablets (Grunenthal Ltd) |
| 46018 | Tapentadol 100mg modified-release tablets |
| 46019 | Tapentadol 150mg modified-release tablets |
| 46020 | Palexia SR 100mg tablets (Grunenthal Ltd) |
| 46021 | Tapentadol 50mg modified-release tablets |
| 46022 | Palexia 75mg tablets (Grunenthal Ltd) |
| 46159 | Palexia SR 150mg tablets (Grunenthal Ltd) |
| 46187 | Oxycodone 120mg modified-release tablets |
| 46279 | Tramadol 200mg modified-release capsules (A A H Pharmaceuticals Ltd) |
| 46354 | PecFent 400micrograms/dose nasal spray (Kyowa Kirin Ltd) |
| 46461 | Tapentadol 75mg tablets |
| 46555 | Fentanyl 800microgram buccal tablets sugar free |
| 46559 | Fencino 12micrograms/hour transdermal patches (Ethypharm UK Ltd) |
| 46560 | Fencino 50micrograms/hour transdermal patches (Ethypharm UK Ltd) |
| 46587 | Tramadol 100mg/ml oral drops |
| 46643 | Zeridame SR 150mg tablets (Actavis UK Ltd) |
| 46657 | Fencino 75micrograms/hour transdermal patches (Ethypharm UK Ltd) |
| 46658 | Fencino 100micrograms/hour transdermal patches (Ethypharm UK Ltd) |
| 46659 | Palexia SR 200mg tablets (Grunenthal Ltd) |
| 46733 | Fencino 25micrograms/hour transdermal patches (Ethypharm UK Ltd) |
| 47072 | Omnopon 15.4mg/ml Injection (Roche Products Ltd) |
| 47154 | Filnarine SR 200mg tablets (Teva UK Ltd) |
| 47399 | Tapentadol 250mg modified-release tablets |
| 47413 | Fentanyl 75micrograms/hr Transdermal patch (Sandoz Ltd) |
| 47460 | Palexia SR 250mg tablets (Grunenthal Ltd) |
| 47555 | Morphine sulfate 10mg/10ml solution for injection Minijet pre-filled syringes (UCB Pharma Ltd) |
| 47671 | Diamorphine hydrochloride 10mg Injection (Approved Prescription Services Ltd) |
| 47672 | Diamorphine hydrochloride 30mg Injection (Hillcross Pharmaceuticals Ltd) |
| 47753 | Morphine 90mg modified-release capsules |
| 47759 | Fentanyl 400micrograms/dose nasal spray |
| 47854 | Tramadol (roi) Tablet |
| 47867 | Morphine 150mg modified-release capsules |
| 47919 | Codeine phosphate 15mg Tablet (Wockhardt UK Ltd) |
| 47949 | Morphine 120mg modified-release capsules |
| 47952 | Codeine phosphate 30mg Tablet (Wockhardt UK Ltd) |
| 47985 | M-eslon 10mg Capsule (Trinity Pharmaceuticals Ltd) |
| 48090 | Tramadol 200mg modified-release tablets (A A H Pharmaceuticals Ltd) |
| 48128 | Pethidine 100mg/2ml solution for injection ampoules (Martindale Pharmaceuticals Ltd) |
| 48133 | Dihydrocodeine 30mg tablets (Almus Pharmaceuticals Ltd) |
| 48148 | Pethidine 100mg/2ml solution for injection ampoules (Actavis UK Ltd) |
| 48158 | Diamorphine 5mg powder for solution for injection vials (Teva UK Ltd) |
| 48183 | Fentanyl 100micrograms/2ml solution for injection ampoules (Martindale Pharmaceuticals Ltd) |
| 48259 | Diamorphine 5mg powder for solution for injection ampoules |
| 48413 | Diamorphine 5mg powder for solution for injection vials |
| 48434 | Diamorphine 10mg powder for solution for injection ampoules |
| 48483 | Diamorphine 30mg powder for solution for injection ampoules |
| 48571 | Durogesic DTrans 50micrograms transdermal patches (Waymade Healthcare Plc) |
| 48604 | Morphine sulfate 10mg/1ml solution for injection ampoules |
| 48880 | Diamorphine 10mg powder for solution for injection vials |
| 48912 | Diamorphine 30mg powder for solution for injection vials |
| 48913 | Diamorphine 100mg powder for solution for injection ampoules |
| 48953 | Diamorphine 100mg powder for solution for injection vials |
| 49323 | Marol 150mg modified-release tablets (Teva UK Ltd) |
| 49324 | Marol 100mg modified-release tablets (Teva UK Ltd) |
| 49742 | OxyContin 5mg modified-release tablets (DE Pharmaceuticals) |
| 49787 | OxyContin 20mg modified-release tablets (Lexon (UK) Ltd) |
| 49791 | OxyNorm 20mg/2ml solution for injection ampoules (Waymade Healthcare Plc) |
| 49940 | OxyNorm 5mg capsules (Lexon (UK) Ltd) |
| 49976 | Morphine sulfate 10mg/1ml solution for injection ampoules (A A H Pharmaceuticals Ltd) |
| 50095 | OxyNorm 5mg capsules (DE Pharmaceuticals) |
| 50421 | Codeine phosphate 15mg Tablet (Celltech Pharma Europe Ltd) |
| 50513 | Morphine sulfate 10mg/1ml solution for injection ampoules (UCB Pharma Ltd) |
| 50532 | Dihydrocodeine 30mg tablets (Bristol Laboratories Ltd) |
| 50671 | Fentanyl 12micrograms/hour transdermal patches (A A H Pharmaceuticals Ltd) |
| 50726 | OxyNorm 10mg/1ml solution for injection ampoules (Waymade Healthcare Plc) |
| 50733 | OxyContin 10mg modified-release tablets (Mawdsley-Brooks & Company Ltd) |
| 50862 | Marol 200mg modified-release tablets (Teva UK Ltd) |
| 50929 | Durogesic DTrans 12micrograms transdermal patches (Mawdsley-Brooks & Company Ltd) |
| 50947 | Tramadol 100mg modified-release capsules (Alliance Healthcare (Distribution) Ltd) |
| 51235 | Fentanyl 25micrograms/hour transdermal patches (Phoenix Healthcare Distribution Ltd) |
| 51384 | OxyNorm 20mg capsules (DE Pharmaceuticals) |
| 51611 | OxyNorm 10mg/1ml solution for injection ampoules (Mawdsley-Brooks & Company Ltd) |
| 51789 | OxyNorm 10mg capsules (Waymade Healthcare Plc) |
| 51896 | OxyContin 80mg modified-release tablets (Mawdsley-Brooks & Company Ltd) |
| 52178 | Fentanyl 100micrograms/2ml solution for injection ampoules (AMCo) |
| 52216 | Longtec 5mg modified-release tablets (Qdem Pharmaceuticals Ltd) |
| 52217 | Longtec 10mg modified-release tablets (Qdem Pharmaceuticals Ltd) |
| 52220 | Longtec 40mg modified-release tablets (Qdem Pharmaceuticals Ltd) |
| 52400 | Pethidine 100mg/10ml solution for injection ampoules (Alliance Healthcare (Distribution) Ltd) |
| 52495 | Tramadol 50mg capsules (Bristol Laboratories Ltd) |
| 52592 | OxyNorm 10mg capsules (DE Pharmaceuticals) |
| 52605 | Tramadol 50mg capsules (Accord Healthcare Ltd) |
| 52809 | OxyContin 10mg modified-release tablets (Lexon (UK) Ltd) |
| 52977 | Tramadol 100mg modified-release capsules (A A H Pharmaceuticals Ltd) |
| 53062 | Hydromorphone 10mg/1ml solution for injection ampoules |
| 53106 | Morphine sulfate 5mg/5ml solution for injection ampoules |
| 53113 | OxyContin 10mg modified-release tablets (DE Pharmaceuticals) |
| 53116 | Longtec 20mg modified-release tablets (Qdem Pharmaceuticals Ltd) |
| 53181 | Diamorphine 10mg powder for solution for injection vials (Teva UK Ltd) |
| 53273 | Morphine hydrochloride 10mg/5ml oral solution (Special Order) |
| 53417 | Diamorphine 10mg powder for solution for injection ampoules (Accord Healthcare Ltd) |
| 53617 | Ibuprofen and codeine 200mg+12.8mg Tablet (Almus Pharmaceuticals Ltd) |
| 53639 | Morphine 10mg modified-release tablets (Sigma Pharmaceuticals Plc) |
| 53709 | Pethidine oral liquid |
| 53918 | Morphine sulfate 10mg/1ml solution for injection ampoules (Hameln Pharmaceuticals Ltd) |
| 53929 | Pethidine 50mg/1ml solution for injection ampoules (A A H Pharmaceuticals Ltd) |
| 53999 | Codeine phosphate 60mg Tablet (Wockhardt UK Ltd) |
| 54017 | Morphine sulphate Capsule |
| 54023 | Tramadol 50mg modified-release capsules (A A H Pharmaceuticals Ltd) |
| 54085 | Pethidine capsule |
| 54354 | Dihydrocodeine 30mg tablets (Kent Pharmaceuticals Ltd) |
| 54406 | Morphine sulfate 30mg suppositories (Martindale Pharmaceuticals Ltd) |
| 54520 | Morphine sulphate Oral solution |
| 54694 | Longtec 80mg modified-release tablets (Qdem Pharmaceuticals Ltd) |
| 54790 | Pethidine 50mg tablets (Teva UK Ltd) |
| 54806 | Transtec 52.5micrograms/hour transdermal patches (DE Pharmaceuticals) |
| 54979 | Fentanyl 50micrograms/hour transdermal patches (A A H Pharmaceuticals Ltd) |
| 55221 | Diamorphine 500mg powder for solution for injection ampoules |
| 55365 | Morphine sulfate 50mg/50ml solution for infusion vials (A A H Pharmaceuticals Ltd) |
| 55724 | Diamorphine 500mg powder for solution for injection vials |
| 55752 | Fentanyl 100micrograms/hour transdermal patches (Phoenix Healthcare Distribution Ltd) |
| 55832 | Morphine sulphate 15mg/ml Injection (Celltech Pharma Europe Ltd) |
| 55839 | Pethidine 50mg/ml Injection (Roche Products Ltd) |
| 55852 | Pethidine 10mg/ml Injection (Martindale Pharmaceuticals Ltd) |
| 56022 | Pethidine 50mg Capsule (Martindale Pharmaceuticals Ltd) |
| 56178 | Codeine 60mg/1ml solution for injection ampoules (A A H Pharmaceuticals Ltd) |
| 56202 | Morphine sulphate Injection |
| 56329 | Morphine sulfate 10mg/2ml solution for injection ampoules |
| 56491 | Zamadol SR 200mg capsules (Lexon (UK) Ltd) |
| 56544 | Morphine sulfate 50mg/50ml solution for infusion vials (Martindale Pharmaceuticals Ltd) |
| 56581 | Alfentanil 5mg/5ml buccal spray |
| 56665 | Oxylan 10mg modified-release tablets (Chanelle Medical UK Ltd) |
| 56670 | Fentanyl 25micrograms/hour transdermal patches (Sigma Pharmaceuticals Plc) |
| 56671 | BuTrans 5micrograms/hour transdermal patches (Waymade Healthcare Plc) |
| 56788 | Morphine sulfate 10mg/5ml oral solution (A A H Pharmaceuticals Ltd) |
| 57027 | Pethidine 50mg tablets (A A H Pharmaceuticals Ltd) |
| 57033 | Oxylan 10mg modified-release tablets (Actavis UK Ltd) |
| 57052 | Oxylan 20mg modified-release tablets (Actavis UK Ltd) |
| 57623 | Morphine sulfate 15mg/1ml solution for injection ampoules (UCB Pharma Ltd) |
| 57750 | Morphine sulfate 15mg/1ml solution for injection ampoules (Wockhardt UK Ltd) |
| 58039 | Oxycodone 5mg/5ml oral solution |
| 58114 | Lynlor 20mg capsules (Actavis UK Ltd) |
| 58129 | Zeridame SR 100mg tablets (Actavis UK Ltd) |
| 58190 | Pethidine 50mg/5ml solution for injection ampoules (A A H Pharmaceuticals Ltd) |
| 58217 | Lynlor 5mg capsules (Actavis UK Ltd) |
| 58279 | Diamorphine 10mg tablets (A A H Pharmaceuticals Ltd) |
| 58290 | Morphine sulfate 10mg suppositories (Martindale Pharmaceuticals Ltd) |
| 58316 | Tramadol 50mg modified-release capsules (DE Pharmaceuticals) |
| 58493 | Oxylan 20mg modified-release tablets (Chanelle Medical UK Ltd) |
| 58499 | Diamorphine 3mg/5ml oral solution |
| 58710 | Morphine sulfate 1mg/1ml solution for injection ampoules |
| 58737 | Pethidine 50mg tablets (Alliance Healthcare (Distribution) Ltd) |
| 58766 | BuTrans 10micrograms/hour transdermal patches (Waymade Healthcare Plc) |
| 58836 | Morphine sulfate 5mg/1ml solution for injection ampoules |
| 58853 | Lynlor 10mg capsules (Actavis UK Ltd) |
| 58879 | Morphine hydrochloride 10mg/5ml oral solution |
| 59057 | Fentanyl 400microgram buccal films sugar free |
| 59146 | BuTrans 20micrograms/hour transdermal patches (Waymade Healthcare Plc) |
| 59392 | Hapoctasin 70micrograms/hour transdermal patches (Actavis UK Ltd) |
| 59443 | Fentanyl 200microgram buccal films sugar free |
| 59473 | Hapoctasin 52.5micrograms/hour transdermal patches (Actavis UK Ltd) |
| 59482 | Fentanyl 37.5microgram/hour transdermal patches |
| 59490 | Mezolar Matrix 37.5microgram/hour transdermal patches (Sandoz Ltd) |
| 59584 | Morphine sulfate 10mg/5ml oral solution (Alliance Healthcare (Distribution) Ltd) |
| 59618 | Transtec 35micrograms/hour transdermal patches (Mawdsley-Brooks & Company Ltd) |
| 59678 | Fentanyl 100micrograms/2ml solution for injection ampoules (A A H Pharmaceuticals Ltd) |
| 59865 | Shortec 10mg capsules (Qdem Pharmaceuticals Ltd) |
| 59978 | Dihydrocodeine 30mg tablets (Waymade Healthcare Plc) |
| 59989 | Dihydrocodeine 30mg tablets (Ranbaxy (UK) Ltd) |
| 60040 | Generic Migraleve Pink tablets |
| 60080 | Alfentanil 500micrograms/1ml solution for injection ampoules |
| 60082 | Morphine sulfate 5mg/10ml solution for injection ampoules |
| 60121 | Tramadol 50mg modified-release capsules (Waymade Healthcare Plc) |
| 60146 | Shortec 5mg capsules (Qdem Pharmaceuticals Ltd) |
| 60158 | Shortec 20mg capsules (Qdem Pharmaceuticals Ltd) |
| 60170 | Hapoctasin 35micrograms/hour transdermal patches (Actavis UK Ltd) |
| 60196 | Oxylan 40mg modified-release tablets (Chanelle Medical UK Ltd) |
| 60296 | Remifentanil 5mg powder for solution for injection vials |
| 60477 | Fentanyl 25micrograms/hour transdermal patches (A A H Pharmaceuticals Ltd) |
| 60489 | Codeine 30mg suppositories |
| 60507 | Morphine 0.1% in Intrasite gel |
| 60518 | Morphine sulfate 500micrograms/5ml oral solution |
| 60721 | Diamorphine 5mg powder for solution for injection ampoules (Accord Healthcare Ltd) |
| 60751 | Tilodol SR 200mg tablets (Sandoz Ltd) |
| 60759 | Tapentadol 20mg/ml oral solution sugar free |
| 60766 | Fentanyl 25micrograms/hour transdermal patches (Waymade Healthcare Plc) |
| 60943 | Transtec 35micrograms/hour transdermal patches (Sigma Pharmaceuticals Plc) |
| 60950 | Morphine sulfate 5mg/5ml oral solution |
| 61086 | Opiodur 12micrograms/hour transdermal patches (Pfizer Ltd) |
| 61156 | Fentanyl 12micrograms/hour transdermal patches (Waymade Healthcare Plc) |
| 61241 | Morphine sulfate 50mg/50ml solution for infusion vials (Alliance Healthcare (Distribution) Ltd) |
| 61272 | Tramadol 50mg capsules (Phoenix Healthcare Distribution Ltd) |
| 61305 | Mylafent 75micrograms/hour transdermal patches (Mylan) |
| 61400 | Morphine sulfate 30mg/1ml solution for injection ampoules (A A H Pharmaceuticals Ltd) |
| 61423 | Morphine 30mg modified-release tablets (Sigma Pharmaceuticals Plc) |
| 61506 | Morphine sulfate 10mg/1ml solution for injection ampoules (Wockhardt UK Ltd) |
| 61584 | Morphine sulfate 50mg/50ml solution for infusion vials (Torbay Pharmaceuticals) |
| 61610 | Tramadol 50mg capsules (Morningside Healthcare Ltd) |
| 61708 | Recivit 267microgram sublingual tablets (Grunenthal Ltd) |
| 61744 | Morphine sulfate 100mg/50ml solution for infusion vials (A A H Pharmaceuticals Ltd) |
| 61764 | Palexia 20mg/ml oral solution (Grunenthal Ltd) |
| 61775 | Tramadol 50mg capsules (Sigma Pharmaceuticals Plc) |
| 61779 | Reltebon 40mg modified-release tablets (Actavis UK Ltd) |
| 61836 | Reltebon 20mg modified-release tablets (Actavis UK Ltd) |
| 61918 | Morphine sulfate 20mg/2ml solution for injection ampoules |
| 61935 | Reltebon 10mg modified-release tablets (Actavis UK Ltd) |
| 61936 | Reltebon 5mg modified-release tablets (Actavis UK Ltd) |
| 61942 | Morphine sulfate 2.5mg/5ml solution for injection ampoules |
| 62322 | Reltebon 80mg modified-release tablets (Actavis UK Ltd) |
| 62689 | Morphine hydrochloride 1mg/1ml solution for injection ampoules |
| 62778 | Tramacet 37.5mg/325mg tablets (Waymade Healthcare Plc) |
| 63047 | Tramadol 100mg modified-release capsules (Waymade Healthcare Plc) |
| 63139 | Fentanyl 12micrograms/hour transdermal patches (Phoenix Healthcare Distribution Ltd) |
| 63182 | Pethidine 50mg/1ml solution for injection ampoules (Alliance Healthcare (Distribution) Ltd) |
| 63198 | OxyContin 40mg modified-release tablets (DE Pharmaceuticals) |
| 63332 | Reltebon 30mg modified-release tablets (Actavis UK Ltd) |
| 63340 | Fentanyl 133microgram sublingual tablets sugar free |
| 63398 | Fentanyl 2.5mg/50ml solution for infusion vials |
| 63423 | Morphine sulfate 10mg/1ml solution for injection ampoules (DE Pharmaceuticals) |
| 63547 | Codeine 1mg suppositories |
| 63593 | Morphine sulfate 10mg/5ml oral solution (Actavis UK Ltd) |
| 63714 | Reltebon 60mg modified-release tablets (Actavis UK Ltd) |
| 63898 | Tramadol 50mg modified-release capsules (J M McGill Ltd) |
| 64079 | Dihydrocodeine 10mg/5ml oral solution (Waymade Healthcare Plc) |
| 64150 | Oxylan 5mg modified-release tablets (Chanelle Medical UK Ltd) |
| 64164 | Reltebon 15mg modified-release tablets (Actavis UK Ltd) |
| 64333 | Longtec 30mg modified-release tablets (Qdem Pharmaceuticals Ltd) |
| 64417 | Morphine sulfate 2mg/5ml oral solution |
| 64426 | Longtec 15mg modified-release tablets (Qdem Pharmaceuticals Ltd) |
| 64459 | Tramadol 37.5mg / Paracetamol 325mg tablets (A A H Pharmaceuticals Ltd) |
| 64496 | Tramadol 100mg modified-release capsules (Ennogen Healthcare Ltd) |
| 64552 | Longtec 60mg modified-release tablets (Qdem Pharmaceuticals Ltd) |
| 64731 | Tramadol 100mg modified-release capsules (Icarus Pharmaceuticals Ltd) |
| 64751 | Codeine 60mg/1ml solution for injection ampoules (Alliance Healthcare (Distribution) Ltd) |
| 64780 | Morphine sulfate 50mg/1ml solution for injection ampoules |
| 64781 | Morphine sulfate 50mg/5ml solution for injection ampoules |
| 64807 | Longtec 120mg modified-release tablets (Qdem Pharmaceuticals Ltd) |
| 64860 | Morphine sulfate 10mg/10ml solution for injection ampoules |
| 64871 | Maneo 100mg modified-release tablets (Mylan) |
| 64965 | Oxycodone 5mg/5ml oral solution sugar free (Wockhardt UK Ltd) |
| 65168 | Mylafent 12micrograms/hour transdermal patches (Mylan) |
| 65266 | Tramadol 50mg capsules (Kent Pharmaceuticals Ltd) |
| 65359 | Mylafent 50micrograms/hour transdermal patches (Mylan) |
| 65372 | Diamorphine 5mg powder for solution for injection ampoules (A A H Pharmaceuticals Ltd) |
| 65390 | Abtard 30mg modified-release tablets (Ethypharm UK Ltd) |
| 65392 | Abtard 20mg modified-release tablets (Ethypharm UK Ltd) |
| 65437 | Fentanyl 100micrograms/hour transdermal patches (A A H Pharmaceuticals Ltd) |
| 65646 | Fentanyl 267microgram sublingual tablets sugar free |
| 65689 | Dihydrocodeine 30mg/5ml oral solution |
| 65932 | Abtard 40mg modified-release tablets (Ethypharm UK Ltd) |
| 65933 | Abtard 80mg modified-release tablets (Ethypharm UK Ltd) |
| 65954 | Tramadol 50mg modified-release capsules (Cubic Pharmaceuticals Ltd) |
| 66121 | Dihydrocodeine 10mg/5ml oral suspension |
| 66280 | BuTrans 15micrograms/hour transdermal patches (Napp Pharmaceuticals Ltd) |
| 66298 | Abtard 10mg modified-release tablets (Ethypharm UK Ltd) |
| 66299 | Maneo 200mg modified-release tablets (Mylan) |
| 66336 | Morphine sulphate 10mg/ml Injection (Celltech Pharma Europe Ltd) |
| 66463 | Buprenorphine 15micrograms/hour transdermal patches |
| 66470 | Butec 10micrograms/hour transdermal patches (Qdem Pharmaceuticals Ltd) |
| 66606 | Oxeltra 10mg modified-release tablets (Wockhardt UK Ltd) |
| 66616 | OxyContin 5mg modified-release tablets (Mawdsley-Brooks & Company Ltd) |
| 66619 | OxyContin 20mg modified-release tablets (Waymade Healthcare Plc) |
| 66654 | Diamorphine 15mg powder for solution for injection ampoules |
| 66689 | Butec 5micrograms/hour transdermal patches (Qdem Pharmaceuticals Ltd) |
| 66695 | Butec 20micrograms/hour transdermal patches (Qdem Pharmaceuticals Ltd) |
| 66729 | Tilodol SR 100mg tablets (Sandoz Ltd) |
| 66760 | Abtard 5mg modified-release tablets (Ethypharm UK Ltd) |
| 66815 | Morphine sulfate 10mg/10ml solution for injection ampoules (Hameln Pharmaceuticals Ltd) |
| 66837 | Abtard 15mg modified-release tablets (Ethypharm UK Ltd) |
| 67018 | Buprenorphine 35micrograms/hour transdermal patches (A A H Pharmaceuticals Ltd) |
| 67161 | Tramadol 150mg modified-release capsules (A A H Pharmaceuticals Ltd) |
| 67197 | Tramadol 50mg capsules (DE Pharmaceuticals) |
| 67258 | Durogesic DTrans 25micrograms transdermal patches (Waymade Healthcare Plc) |
| 67310 | Zydol SR 200mg tablets (Mawdsley-Brooks & Company Ltd) |
| 67323 | Zydol SR 150mg tablets (Waymade Healthcare Plc) |
| 67356 | Transtec 70micrograms/hour transdermal patches (Lexon (UK) Ltd) |
| 67425 | Yemex 25micrograms/hour transdermal patches (Sandoz Ltd) |
| 67446 | Abtard 60mg modified-release tablets (Ethypharm UK Ltd) |
| 67474 | Opiodur 50micrograms/hour transdermal patches (Pfizer Ltd) |
| 67599 | Pethidine 100mg/2ml solution for injection ampoules (AMCo) |
| 67744 | Zydol 50mg capsules (Lexon (UK) Ltd) |
| 67766 | Fentanyl 75micrograms/hour transdermal patches (Phoenix Healthcare Distribution Ltd) |
| 67796 | Diamorphine 10mg powder for solution for injection ampoules (A A H Pharmaceuticals Ltd) |
| 67830 | Fentanyl 12micrograms/hour transdermal patches (DE Pharmaceuticals) |
| 67901 | Transtec 52.5micrograms/hour transdermal patches (Lexon (UK) Ltd) |
| 68167 | Reletrans 5micrograms/hour transdermal patches (Sandoz Ltd) |
| 68172 | Reletrans 20micrograms/hour transdermal patches (Sandoz Ltd) |
| 68196 | Reletrans 15micrograms/hour transdermal patches (Sandoz Ltd) |
| 68209 | Mylafent 100micrograms/hour transdermal patches (Mylan) |
| 68210 | Tramadol 100mg modified-release tablets (Elite Pharma (Surrey) Ltd) |
| 68241 | Reletrans 10micrograms/hour transdermal patches (Sandoz Ltd) |
| 68402 | Panitaz 5micrograms/hour transdermal patches (Dr Reddy's Laboratories (UK) Ltd) |
| 68427 | Tramadol 50mg modified-release capsules (CST Pharma Ltd) |
| 68472 | Prenotrix 52.5micrograms/hour transdermal patches (Genesis Pharmaceuticals Ltd) |
| 68479 | Bupeaze 70micrograms/hour transdermal patches (Dr Reddy's Laboratories (UK) Ltd) |
| 68509 | Codeine phosphate 30mg Tablet (Celltech Pharma Europe Ltd) |
| 68559 | Panitaz 10micrograms/hour transdermal patches (Dr Reddy's Laboratories (UK) Ltd) |
| 68712 | Morphine hydrochloride 100mg/5ml oral solution |
| 68743 | Bupeaze 35micrograms/hour transdermal patches (Dr Reddy's Laboratories (UK) Ltd) |
| 68797 | Oxeltra 20mg modified-release tablets (Wockhardt UK Ltd) |
| 68833 | Tramadol 100mg modified-release capsules (DE Pharmaceuticals) |
| 68848 | Buplast 52.5micrograms/hour transdermal patches (Mylan) |
| 68888 | Sevodyne 20micrograms/hour transdermal patches (Aspire Pharma Ltd) |
| 68889 | Sevodyne 10micrograms/hour transdermal patches (Aspire Pharma Ltd) |
| 68890 | Sevodyne 5micrograms/hour transdermal patches (Aspire Pharma Ltd) |
| 69023 | Yemex 100micrograms/hour transdermal patches (Sandoz Ltd) |
| 69063 | Hydromorphone 2mg/1ml solution for injection ampoules |
| 69243 | Bupeaze 52.5micrograms/hour transdermal patches (Dr Reddy's Laboratories (UK) Ltd) |
| 69254 | Buplast 35micrograms/hour transdermal patches (Mylan) |
| 69285 | Boots Ibuprofen and Codeine 200mg/12.8mg tablets (The Boots Company Plc) |
| 69315 | Butec 15micrograms/hour transdermal patches (Qdem Pharmaceuticals Ltd) |
| 69474 | Oxycodone 5mg/5ml oral solution sugar free (DE Pharmaceuticals) |
| 69559 | Shortec liquid 5mg/5ml oral solution (Qdem Pharmaceuticals Ltd) |
| 69795 | Prenotrix 35micrograms/hour transdermal patches (Genesis Pharmaceuticals Ltd) |
| 69894 | Zeridame SR 200mg tablets (Actavis UK Ltd) |
| 69993 | Shortec 10mg/ml concentrate oral solution (Qdem Pharmaceuticals Ltd) |
| 70117 | Panitaz 20micrograms/hour transdermal patches (Dr Reddy's Laboratories (UK) Ltd) |
| 70123 | Shortec 10mg/1ml solution for injection ampoules (Qdem Pharmaceuticals Ltd) |
| 70124 | Relevtec 70micrograms/hour transdermal patches (Sandoz Ltd) |
| 70139 | Relevtec 35micrograms/hour transdermal patches (Sandoz Ltd) |
| 70274 | Morphine sulfate 5mg/5ml solution for injection ampoules (Torbay Pharmaceuticals) |
| 70376 | Yemex 50micrograms/hour transdermal patches (Sandoz Ltd) |
| 70460 | Bupramyl 10micrograms/hour transdermal patches (Mylan) |
| 70461 | Bupramyl 5micrograms/hour transdermal patches (Mylan) |
| 70552 | Codeine 60mg/1ml solution for injection ampoules (Torbay Pharmaceuticals) |
| 70631 | Relevtec 52.5micrograms/hour transdermal patches (Sandoz Ltd) |
| 70800 | Palladone 10mg/1ml solution for injection ampoules (Napp Pharmaceuticals Ltd) |
| 70801 | Palladone 2mg/1ml solution for injection ampoules (Napp Pharmaceuticals Ltd) |
| 70810 | Yemex 12micrograms/hour transdermal patches (Sandoz Ltd) |
| 70988 | Fentanyl 50micrograms/hour transdermal patches (Phoenix Healthcare Distribution Ltd) |
| 71170 | Pentazocine 25mg tablets (Actavis UK Ltd) |
| 71171 | Morphine sulfate 10mg/10ml solution for injection ampoules (Torbay Pharmaceuticals) |
| 71310 | Bupramyl 20micrograms/hour transdermal patches (Mylan) |
| 71335 | Oxycodone 20mg modified-release tablets (Teva UK Ltd) |
| 71355 | Tramadol 150mg modified-release capsules (Waymade Healthcare Plc) |
| 71358 | Tramadol 150mg modified-release tablets (Sigma Pharmaceuticals Plc) |
| 71462 | Morphine sulfate 1mg/1ml solution for injection ampoules (Torbay Pharmaceuticals) |
| 71711 | Busiete 10micrograms/hour transdermal patches (Teva UK Ltd) |
| 72098 | Turgeon 35micrograms/hour transdermal patches (Teva UK Ltd) |
| 72160 | Turgeon 52.5micrograms/hour transdermal patches (Teva UK Ltd) |
| 72209 | Dihydrocodeine 30mg tablets (DE Pharmaceuticals) |
| 72210 | Dihydrocodeine 30mg tablets (Alliance Healthcare (Distribution) Ltd) |
| 72225 | Morphine sulfate 10mg/5ml oral solution (Wockhardt UK Ltd) |
| 72255 | Morphine 0.2% in Intrasite gel |
| 72265 | Dihydrocodeine 15mg/5ml oral solution |
| 72342 | Yemex 75micrograms/hour transdermal patches (Sandoz Ltd) |
| 72457 | Sevredol 20mg Suppository (Napp Pharmaceuticals Ltd) |
| 72646 | Zydol SR 100mg tablets (DE Pharmaceuticals) |

# S1 Table O Codelist for sexual dysfunction – main analysis.

| medcode | readcode | Read term |
| --- | --- | --- |
| 9485 | Eu52.00 | [x]sex dysfunction not caused by organic disorder or disease |
| 28283 | Eu52000 | [x]lack or loss of sexual desire |
| 56603 | Eu52012 | [x]hypoactive sexual desire disorder |
| 21122 | Eu52013 | [x] lack of libido |
| 42056 | Eu52100 | [x]sexual aversion and lack of sexual enjoyment |
| 24483 | Eu52111 | [x]anhedonia sexual |
| 60716 | Eu52211 | [x]female sexual arousal disorder |
| 18332 | Eu52300 | [x]orgasmic dysfunction |
| 34336 | Eu52311 | [x]inhibited orgasm |
| 10550 | Eu52312 | [x]psychogenic anorgasmy |
| 19745 | Eu52500 | [x]nonorganic vaginismus |
| 30442 | Eu52511 | [x]psychogenic vaginismus |
| 21089 | Eu52600 | [x]nonorganic dyspareunia |
| 37089 | Eu52611 | [x]psychogenic dyspareunia |
| 44683 | Eu52y00 | [x]oth sex dysfunction, not caused by organic disordr/dsease |
| 48953 | Eu52z00 | [x]unspec sex dysfunction not caused by organic disordr/dis |
| 27791 | Eu66200 | [x]sexual relationship disorder |
| 30414 | Eu66y00 | [x]other psychosexual development disorders |
| 48890 | Eu66z00 | [x]psychosexual development disorder, unspecified |
| 61908 | ZVu5000 | [x]sex counselling,unspecified |

# S1 Table P Codelist for self-harm – main analysis.

| medcode | readcode | readterm |
| --- | --- | --- |
| 171 | SL...15 | overdose of drug |
| 697 | U20..11 | [x]deliberate drug overdose / other poisoning |
| 713 | SLHz.00 | drug and medicament poisoning nos |
| 1435 | S....00 | injury and poisoning |
| 2557 | TK05.00 | suicide + selfinflicted poisoning by drug or medicine nos |
| 3246 | TK...15 | attempted suicide |
| 3406 | TK...17 | para-suicide |
| 3423 | TK...12 | injury - self-inflicted |
| 3500 | SLC1000 | digoxin poisoning |
| 3985 | U2...13 | [x]suicide |
| 5065 | SLH..00 | other and unspecified drug and medicament poisoning |
| 5242 | U2E..00 | [x]self mutilation |
| 5589 | 1BD4.00 | suicide risk |
| 5616 | TK3y.00 | suicide + selfinflicted inj oth mean hang/strangle/suffocate |
| 6595 | TK...11 | cause of overdose - deliberate |
| 7606 | TN82.00 | injury ?accidental, by scald |
| 8229 | U2...15 | [x]para-suicide |
| 9604 | TK60111 | slashed wrists self inflicted |
| 10057 | ZX1..13 | deliberate self-harm |
| 10436 | TN81.00 | injury ?accidental, by burns or fire |
| 10463 | U209.00 | [x]intent self poison/exposure to alcohol |
| 10464 | ZX...00 | self-harm |
| 10644 | TK...13 | poisoning - self-inflicted |
| 10717 | U2...11 | [x]self inflicted injury |
| 10859 | SL85000 | cocaine poisoning |
| 11122 | TK60100 | self inflicted lacerations to wrist |
| 11708 | SL...14 | overdose of biological substance |
| 11753 | 1BDC.00 | intent of deliberate self harm with detailed plans |
| 11910 | SLz..00 | drug, medicament or biological substance poisoning nos |
| 12333 | ZX13.00 | cutting self |
| 12694 | 1BDA.00 | thoughts of deliberate self harm |
| 13557 | TK30.00 | suicide and selfinflicted injury by hanging |
| 14853 | TK00.00 | suicide + selfinflicted poisoning by analgesic/antipyretic |
| 15177 | TK60.00 | suicide and selfinflicted injury by cutting |
| 15437 | 1B19.00 | suicidal |
| 15978 | SL51000 | aspirin poisoning |
| 16485 | TK01.00 | suicide + selfinflicted poisoning by barbiturates |
| 16821 | SL54200 | ibuprofen poisoning |
| 16907 | U30..11 | [x]deliberate drug poisoning |
| 17046 | U2...00 | [x]intentional self-harm |
| 17378 | U2...14 | [x]attempted suicide |
| 17465 | SLD2000 | dioctyl sulphosuccinate poisoning |
| 17661 | SL52100 | paracetamol poisoning |
| 18379 | U200.11 | [x]overdose - paracetamol |
| 18671 | 1BD8.00 | at risk of dsh - deliberate self harm |
| 18983 | ZX1I.00 | self-scalding |
| 19716 | ZQ54.00 | suicide risk assessment |
| 20485 | Tz...00 | causes of injury and poisoning nos |
| 20586 | SL61000 | phenytoin poisoning |
| 20625 | U2...12 | [x]injury - self-inflicted |
| 20650 | U200z00 | [x]intent self poison nonopioid analgesic unspecif place |
| 20668 | TN7..00 | injury ?accidental, fall from high place |
| 21027 | TK...14 | suicide and self harm |
| 21029 | TK...00 | suicide and selfinflicted injury |
| 21211 | U200.00 | [x]intent self poison/exposure to nonopioid analgesic |
| 22107 | ZV1B200 | [v]personal history of self-harm |
| 22199 | TK04.00 | suicide + selfinflicted poisoning by other drugs/medicines |
| 22281 | ZX11.00 | biting self |
| 22410 | ZRn..00 | suicide intent score scale |
| 22433 | SLG5.00 | eye drug poisoning nec |
| 22877 | 1BDD.00 | unknown risk of deliberate self harm |
| 23080 | TK3..00 | suicide + selfinflicted injury by hang/strangulate/suffocate |
| 23340 | SL23400 | insulin poisoning |
| 23753 | TK7..00 | suicide and selfinflicted injury by jumping from high place |
| 23823 | U40..00 | [x]poisoning/expos to noxious substance,undetermined intent |
| 23979 | SL90100 | imipramine poisoning |
| 23982 | SL51.00 | salicylate poisoning |
| 24086 | U20A.11 | [x]self poisoning from glue solvent |
| 24461 | U27..00 | [x]intentional self harm by smoke, fire and flames |
| 24463 | U20B.11 | [x]self carbon monoxide poisoning |
| 25274 | TNz..00 | injury undetermined accidental or purposely inflicted nos |
| 25684 | TN...11 | poisoning undetermined - accidentally or purposely inflicted |
| 25854 | ZX19.00 | hitting self |
| 25859 | ZX1G.00 | scratches self |
| 25912 | 38B0.00 | suicide risk assessment |
| 26465 | U29..00 | [x]intentional self harm by sharp object |
| 27470 | TK61.00 | suicide and selfinflicted injury by stabbing |
| 27522 | TK03.00 | suicide + selfinflicted poisoning tranquilliser/psychotropic |
| 27713 | U20..00 | [x]intentional self poisoning/exposure to noxious substances |
| 28080 | TK07.00 | suicide + selfinflicted poisoning by corrosive/caustic subst |
| 28115 | TK51.00 | suicide and selfinflicted injury by shotgun |
| 28164 | SL91200 | prochlorperazine poisoning |
| 28481 | SLG..12 | eye drug poisoning |
| 28680 | ZX1LD00 | [x]self mutilation |
| 28694 | ZX18.00 | hanging self |
| 28710 | U205.11 | [x]overdose - heroin |
| 29134 | TN5..00 | injury ?accidental, by firearms and explosives |
| 29288 | SL7z.11 | sleeping drug poisoning |
| 29305 | SLG0.00 | local anti-infective and anti-inflammatory poisoning |
| 29424 | 1BD2.00 | morbid thoughts |
| 29861 | U200.13 | [x]overdose - aspirin |
| 30074 | U290.00 | [x]intentional self harm by sharp object occurrence at home |
| 30292 | TK0..00 | suicide + selfinflicted poisoning by solid/liquid substances |
| 30360 | U21..00 | [x]intent self harm by hanging strangulation / suffocation |
| 30370 | ZX1H100 | self-strangulation |
| 31074 | SLC..00 | cardiovascular drug poisoning |
| 31202 | SL60100 | trimethadione poisoning |
| 31754 | SL97000 | amfetamine poisoning |
| 31836 | TN1..00 | injury ?accidental, poisoning by gases in domestic use |
| 31854 | TK4..00 | suicide and selfinflicted injury by drowning |
| 31861 | SL14300 | proguanil poisoning |
| 32267 | ZX1L.00 | self-mutilation |
| 32457 | SL54300 | naproxen poisoning |
| 32635 | SL24100 | gonadotrophin poisoning |
| 32760 | SL50.12 | opiate poisoning |
| 32864 | SL3y000 | heavy metal agonist poisoning |
| 33596 | TK02.00 | suicide + selfinflicted poisoning by oth sedatives/hypnotics |
| 34156 | U2z..00 | [x]intentional self harm by unspecified means |
| 34457 | SL83000 | ketamine poisoning |
| 34703 | U204.12 | [x]overdose - amitriptyline |
| 34774 | SL42.00 | anticoagulant poisoning |
| 35123 | ZX1L100 | self-mutilation of hands |
| 35183 | SL31.12 | immunosuppressive poisoning |
| 35247 | ZX1..00 | self-injurious behaviour |
| 35419 | 1BD5.00 | high suicide risk |
| 35798 | U4D0.00 | [x]crashng of motor vehicle undetermined intent occ at home |
| 35868 | U2D4.00 | [x]intent self harm by crash motor vehicl occ street/highway |
| 35879 | U208.00 | [x]int self poison/exposure to other/unspec drug/medicament |
| 36084 | TKx2.00 | suicide and selfinflicted injury by scald |
| 36197 | U4B..00 | [x]falling jumping/pushed from high place undeterm intent |
| 36231 | SL96000 | cannabis poisoning |
| 36255 | TK6..00 | suicide and selfinflicted injury by cutting and stabbing |
| 36398 | U204.00 | [x]intent self poison/exposure to psychotropic drug |
| 36750 | SL13000 | chiniofon poisoning |
| 36863 | ZX13.11 | cuts self |
| 37194 | 1BD6.00 | moderate suicide risk |
| 37874 | ZX12.00 | burning self |
| 38008 | U27z.00 | [x]intent self harm by smoke fire/flames occ unspecif place |
| 38079 | TN54.00 | injury ?accidental, by other firearm |
| 38749 | U20B200 | [x]int self poison other gas/vapour school/pub admin area |
| 38760 | U2y..00 | [x]intentional self harm by other specified means |
| 38922 | SL95100 | meprobamate poisoning |
| 39339 | SL04300 | oxytetracycline poisoning |
| 39814 | SLC0200 | propranolol poisoning |
| 41097 | SL6xz00 | anticonvulsant poisoning nos |
| 41241 | TKz..00 | suicide and selfinflicted injury nos |
| 41384 | ZX1B.00 | jumping from height |
| 41400 | U22..00 | [x]intentional self harm by drowning and submersion |
| 41638 | U209000 | [x]int self poison/exposure to alcohol at home |
| 42086 | U20yz00 | [x]intent self poison unspecif chemical unspecif place |
| 42097 | U2C..00 | [x]intent self harm by jumping / lying before moving object |
| 42103 | U2z0.00 | [x]intentional self harm by unspecif means occurrn at home |
| 42418 | U20A.00 | [x]intentional self poison organ solvent,halogen hydrocarb |
| 42433 | SL94600 | poisoning by temazepam |
| 42464 | TKy..00 | late effects of selfinflicted injury |
| 42471 | U21z.00 | [x]intent self harm by hangng strangul/suffoct unspecif plce |
| 42546 | SL30x00 | other antihistamine poisoning |
| 42688 | SLF0000 | ergot alkaloid poisoning |
| 42801 | SL30.13 | antihistamine poisoning |
| 42937 | TK71.00 | suicide+selfinflicted injury-jump from oth manmade structure |
| 43355 | TN85.00 | injury ?accidental, by crashing of motor vehicle |
| 43382 | U40C.00 | [x]poisoning/exposure, ? intent, to pesticide |
| 43738 | SL40100 | ferrous sulphate poisoning |
| 43891 | TN4..00 | injury ?accidental, drowning |
| 44099 | SL1y000 | flucytosine poisoning |
| 44168 | U4A..00 | [x]contact with blunt object, undetermined intent |
| 44441 | SL97100 | caffeine poisoning |
| 44447 | SL90.00 | antidepressant poisoning |
| 44493 | SL5z.00 | analgesic, antipyretic or antirheumatic poisoning nos |
| 44508 | U204000 | [x]int self poison/exposure to psychotropic drug at home |
| 44530 | U202000 | [x]int self poison/exposure to sedative hypnotic at home |
| 44886 | U200.12 | [x]overdose - ibuprofen |
| 44965 | ZX1B100 | jumping from building |
| 45166 | U2D0.00 | [x]intent self harm by crash of motor vehicl occurrn at home |
| 45542 | SLC8000 | adrenochrome poisoning |
| 45709 | U25..00 | [x]intent self harm by other/unspecified firearm discharge |
| 45710 | U205.00 | [x]intent self poison/exposure to narcotic drug |
| 45748 | U202.12 | [x]overdose - diazepam |
| 45796 | U72..00 | [x]sequel intentn self-harm assault+event of undeterm intent |
| 45878 | SL22.00 | ovarian hormone and synthetic substitute poisoning |
| 46144 | U49..00 | [x]contact with sharp object, undetermined intent |
| 46154 | ZX15.00 | drowning self |
| 46173 | ZR2D.00 | beck scale for suicide ideation |
| 46280 | U204.11 | [x]overdose - antidepressant |
| 46287 | SL00.00 | penicillin poisoning |
| 46456 | TKx1.00 | suicide and selfinflicted injury by burns or fire |
| 46623 | TN84.00 | injury ?accidental, by electrocution |
| 46747 | U2B..00 | [x]intentional self harm by jumping from a high place |
| 46911 | ZRLfC12 | honos item 2 - non-accidental self injury |
| 47022 | 1BDB.00 | plans for deliberate self harm without intent |
| 47234 | SL31200 | chlorambucil poisoning |
| 47266 | SL54400 | mefenamic acid poisoning |
| 47268 | SL01200 | nystatin poisoning |
| 47283 | ZX11.11 | bites self |
| 47387 | SLE7100 | colchicine poisoning |
| 47501 | TKx..00 | suicide and selfinflicted injury by other means |
| 47623 | ZX1..12 | sib - self-injurious behaviour |
| 47691 | SL...13 | medicinal poisoning |
| 47769 | SLD0200 | poisoning by histamine h2-receptor antagonists |
| 47793 | SLB..00 | autonomic nervous system drug poisoning |
| 48068 | T180.00 | mvta - accid poisoning - exhaust gas of moving motor vehicle |
| 48324 | U202.16 | [x]overdose - benzodiazepine |
| 48345 | ZX1N.00 | stabbing self |
| 48871 | TK20.00 | suicide + selfinflicted poisoning by motor veh exhaust gas |
| 48887 | SL3z.00 | systemic agent poisoning nos |
| 48934 | U200000 | [x]int self poison/exposure to nonopioid analgesic at home |
| 49135 | TK1y.00 | suicide and selfinflicted poisoning by other utility gas |
| 49552 | U202.17 | [x]overdose - barbiturate |
| 49566 | Eu14000 | [x]mental & behav dis due to use cocaine: acute intoxication |
| 49899 | SLD0.11 | antacid drug poisoning |
| 49914 | SL30400 | tripelennamine poisoning |
| 49930 | SLB1000 | atropine poisoning |
| 50416 | SL6y200 | levodopa (l-dopa) poisoning |
| 50482 | ZX1J.00 | self-electrocution |
| 50502 | TN00.00 | injury ?accidental, poisoning by analgesic or anti-pyretic |
| 50540 | SL94200 | flurazepam poisoning |
| 50543 | SL76.00 | mixed sedative poisoning nec |
| 50587 | SLF7100 | salbutamol poisoning |
| 50601 | ZX19200 | slapping self |
| 50877 | U408.00 | [x]poison/exposure, ?intent, to other/unspec drug/medicament |
| 51046 | U48..00 | [x]contact with steam hot vapours+objects undetermn intent |
| 51047 | SLG2.12 | local detergent poisoning |
| 51157 | SL44300 | urokinase poisoning |
| 51192 | ZX1S.00 | throwing self onto floor |
| 51224 | U24..00 | [x]intent self harm by rifle shotgun/larger firearm disch |
| 51248 | SL1x200 | isoniazid poisoning |
| 51292 | U202.00 | [x]intent self poison/exposure to sedative hypnotic |
| 51309 | U20B.00 | [x]intent self poison/exposure to other gas/vapour |
| 51328 | TK21.00 | suicide and selfinflicted poisoning by other carbon monoxide |
| 51362 | U20y000 | [x]int self poison/exposure to unspecif chemical at home |
| 51381 | U202.13 | [x]overdose - temazepam |
| 51473 | SLE2000 | acetazolamide poisoning |
| 51543 | SL7..12 | sedative poisoning |
| 51685 | TK31.00 | suicide + selfinflicted injury by suffocation by plastic bag |
| 51776 | SLC5.00 | other vasodilator poisoning |
| 52004 | TN06.00 | injury ?accidental, poisoning by corrosive/caustic substance |
| 52028 | SL14500 | quinine poisoning |
| 52148 | U470.00 | [x]exposure to smoke fire+flame undeterm intent occ at home |
| 52325 | U4D..00 | [x]crashing of motor vehicle, undetermined intent |
| 52376 | U42..00 | [x]drowning and submersion, undetermined intent |
| 52458 | TK06.00 | suicide + selfinflicted poisoning by agricultural chemical |
| 52611 | U40A.00 | [x]pois/exposure,?intent,to organ solvent,halogen hydrocarb |
| 52712 | U205000 | [x]int self poison/exposure to narcotic drug at home |
| 52718 | SL5..11 | analgesic poisoning |
| 52778 | U29z.00 | [x]intentional self harm by sharp object occ unspecif place |
| 52866 | SLC4100 | nitrate poisoning |
| 52881 | U291.00 | [x]intent self harm by sharp object occ resident instit'n |
| 52931 | U202.15 | [x]overdose - nitrazepam |
| 53004 | U20B000 | [x]int self poison/exposure to other gas/vapour at home |
| 53204 | U201000 | [x]int self poison/exposure to antiepileptic at home |
| 53346 | SL8..00 | central nervous system depressants and anaesthetic poisoning |
| 53350 | SL01.00 | antifungal antibiotic poisoning |
| 53444 | U2A3.00 | [x]intent self harm by blunt object occ sports/athlet area |
| 53918 | U1AA.00 | [x]accid poison/exposure to organ solvent,halogen hydrocarb |
| 54091 | U210.00 | [x]intent self harm by hanging strangulat/suffocat occ home |
| 54097 | SLB0100 | anticholinesterase poisoning |
| 54181 | SL5x.00 | other non-narcotic analgesic poisoning |
| 54406 | SL94.00 | benzodiazepine poisoning |
| 54531 | SLB0200 | pilocarpine poisoning |
| 54695 | U20By00 | [x]int self poison other gas/vapour other spec place |
| 54839 | SLF1100 | orciprenaline poisoning |
| 54849 | SL31300 | cyclophosphamide poisoning |
| 54929 | ZX1M.00 | shooting self |
| 54950 | U44..00 | [x]rifle shotgun+larger firearm discharge undetermin intent |
| 55240 | SLC0100 | procainamide poisoning |
| 55395 | U202.11 | [x]overdose - sleeping tabs |
| 55887 | ZRn3.00 | suicide intent score subscale - attempt circumstances |
| 56075 | U2B4.00 | [x]intent self harm by jump from high place occ street/h'way |
| 56137 | TK52.00 | suicide and selfinflicted injury by hunting rifle |
| 56138 | U242.00 | [x]int slf hrm rifl s'gun/lrg frarm dis sch/ins/pub adm area |
| 56296 | SLG4.00 | hair treatment poisoning |
| 56378 | U28..00 | [x]intentional self harm by steam hot vapours / hot objects |
| 56380 | U2D..00 | [x]intentional self harm by crashing of motor vehicle |
| 56421 | SLE1100 | theophylline poisoning |
| 56681 | ZX1H.00 | self-asphyxiation |
| 56693 | SL21.12 | androgen poisoning |
| 56702 | ZRn6.00 | suicide risk scale |
| 56877 | TN5z.00 | injury ?accidental, by firearm or explosive nos |
| 56978 | SL31400 | cytarabine poisoning |
| 57079 | U20y.00 | [x]intent self poison/exposure to unspecif chemical |
| 57163 | SL07200 | bleomycin poisoning |
| 57479 | ZX1L200 | self-mutilation of genitalia |
| 57860 | SLH2.11 | chelating agent poisoning |
| 58039 | ZX1C.00 | nipping self |
| 58594 | U20A000 | [x]intent self pois organ solvent,halogen hydrocarb, home |
| 58605 | TK7z.00 | suicide+selfinflicted injury-jump from high place nos |
| 58831 | TN80.00 | injury ?accidental, by jumping or lying before moving object |
| 58901 | ZX1B200 | jumping from bridge |
| 59021 | SL...11 | biological substance poisoning |
| 59101 | SL2..00 | hormone and synthetic substitute poisoning |
| 59357 | SL50300 | codeine (methylmorphine) poisoning |
| 59363 | SL86.00 | peripheral nerve and plexus-blocking anaesthetic poisoning |
| 59405 | TKx0000 | suicide + selfinflicted injury-jumping before moving object |
| 59414 | U209z00 | [x]intent self poison alcohol unspecif place |
| 59503 | SL85100 | lidocaine poisoning |
| 59611 | SLGx.00 | other skin and mucous membrane drug poisoning |
| 59743 | SL41000 | folic acid poisoning |
| 60201 | SL92000 | haloperidol poisoning |
| 60404 | U221.00 | [x]intent self harm by drowning/submersn occ resid instit'n |
| 60521 | U1A0z00 | [x]accid poison/expos to nonopioid analgesic unspecif place |
| 60559 | U204.13 | [x]overdose - ssri |
| 60652 | SL20300 | poisoning by glucocorticoids and synthetic analogues |
| 60684 | U2A1.00 | [x]intent self harm by blunt object occ resident instit'n |
| 60767 | TK3z.00 | suicide + selfinflicted inj by hang/strangle/suffocate nos |
| 60832 | SLD0000 | aluminium hydroxide poisoning |
| 61113 | TK72.00 | suicide+selfinflicted injury-jump from natural sites |
| 61177 | U2zz.00 | [x]intent self harm by unspecif means occ at unspecif place |
| 61187 | U1A9z00 | [x]accid poison/expos to alcohol unspecif place |
| 61190 | U409z00 | [x]pois/expos ?intent to alcohol unspecif place |
| 61469 | SLB3000 | phenoxybenzamine poisoning |
| 61541 | U408z00 | [x]pois/expos ?intent to oth/unsp drug/medic unspecif place |
| 61546 | U20y200 | [x]int self poison unspecif chemical school/pub admin area |
| 61569 | TK70.00 | suicide+selfinflicted injury-jump from residential premises |
| 61595 | SLFy.00 | other respiratory system drug poisoning |
| 61609 | U4y3.00 | [x]oth specif event undetermin intent occ sport/athlet area |
| 61618 | TK2..00 | suicide + selfinflicted poisoning by other gases and vapours |
| 61768 | SL52200 | phenacetin poisoning |
| 61784 | SL96200 | marihuana poisoning |
| 62115 | SL07100 | dactinomycin poisoning |
| 62231 | SL0y.00 | other specific antibiotic poisoning |
| 62241 | SL81.00 | halothane poisoning |
| 62314 | TN04.00 | injury ?accidental, poisoning by other spec drug/medicament |
| 62382 | ZX1K.00 | self-incineration |
| 62445 | U40B400 | [x]pois/expos ?intent other gas/vapour in street/highway |
| 62446 | SL1z.00 | anti-infective poisoning nos |
| 62462 | SL70100 | barbitone poisoning |
| 62515 | SyuG.00 | [x]toxic effects of substances chiefly nonmedicinal source |
| 62879 | SL95000 | hydroxyzine poisoning |
| 62950 | U208000 | [x]int self poison/exposure to oth/unsp drug/medicam home |
| 63043 | SL42300 | warfarin sodium poisoning |
| 63074 | U2A..00 | [x]intentional self harm by blunt object |
| 63099 | U2C4.00 | [x]int self harm jump/lying befr mov obje occ street/highway |
| 63100 | U274.00 | [x]intent self harm by smoke fire/flame occ street/highway |
| 63260 | SLC6z00 | hypertensive agent poisoning nos |
| 63553 | SL80100 | methocarbamol poisoning |
| 63800 | TN0..00 | injury ?accidental, poisoning by solid/liquid substances |
| 63947 | SL27100 | levothyroxine sodium poisoning |
| 63969 | SL42100 | heparin poisoning |
| 63987 | SL22100 | oestrogen poisoning |
| 64200 | ZX1L600 | self-mutilation of ears |
| 64227 | ZX1K.11 | setting fire to self |
| 64331 | ZX1E.00 | pinching self |
| 64364 | U20Bz00 | [x]intent self poison other gas/vapour unspecif place |
| 64410 | U211.00 | [x]intent self harm by hangng strangult/suffoct resid instit |
| 64559 | SL5yz00 | other analgesic or antipyretic poisoning nos |
| 64724 | SL94z00 | benzodiazepine poisoning nos |
| 64744 | TKx5.00 | suicide and selfinflicted injury by crashing motor vehicle |
| 64805 | U4zz.00 | [x]unspecif event undeterm intent occurrn unspecif place |
| 64912 | SLB2.11 | adrenergic poisoning |
| 65252 | SL50400 | meperidine (pethidine) poisoning |
| 65264 | SLE7.11 | urate metabolism drug poisoning |
| 65272 | U40A300 | [x]pois/exp ?intent org solvent,halogen hydrocarb,sport area |
| 65309 | TK54.00 | suicide and selfinflicted injury by other firearm |
| 65448 | TK6z.00 | suicide and selfinflicted injury by cutting and stabbing nos |
| 65497 | U4C..00 | [x]falling lying running befor/into moving obj undet intent |
| 65732 | TN21.00 | injury ?accidental, poisoning by other carbon monoxide |
| 65810 | SL54000 | gold salt poisoning |
| 65955 | U201.00 | [x]intent self poison/exposure to antiepileptic |
| 66063 | TKxy.00 | suicide and selfinflicted injury by other specified means |
| 66109 | TK01000 | suicide and self inflicted injury by amylobarbitone |
| 66117 | U201z00 | [x]intent self poison antiepileptic unspecif place |
| 66118 | U204z00 | [x]intent self poison psychotropic drug unspecif place |
| 66187 | Eu16000 | [x]mental & behav dis due hallucinogens: acute intoxicatn |
| 66272 | SLG3.00 | emollients, demulcents and protectant poisoning |
| 66329 | SL13.11 | hydroxyquinoline poisoning |
| 66332 | SLD3000 | magnesium sulphate poisoning |
| 66370 | SLEz.00 | water, mineral or uric acid metabolism poisoning nos |
| 66420 | SLC5000 | cyclandelate poisoning |
| 66518 | U1A7z00 | [x]accid poison/expos to oth autonomic drug unspecif place |
| 66538 | SL30000 | chlorphenamine poisoning |
| 66559 | SL82000 | ether poisoning |
| 66621 | TK0z.00 | suicide + selfinflicted poisoning by solid/liquid subst nos |
| 66634 | U207z00 | [x]intent self poison oth autonomic drug unspecif place |
| 66782 | SLF2.00 | skeletal muscle relaxant poisoning |
| 66915 | TK5..00 | suicide and selfinflicted injury by firearms and explosives |
| 66961 | TN20.00 | injury ?accidental, poisoning by motor vehicle exhaust gas |
| 67252 | SLF7000 | aminophylline poisoning |
| 67303 | U4y..00 | [x]other specified events, undetermined intent |
| 67382 | SLB2100 | noradrenalin poisoning |
| 67400 | U280.00 | [x]intent self harm by steam hot vapour/hot obj occ at home |
| 67409 | U2y0.00 | [x]intentionl self harm by oth specif means occurrn at home |
| 67424 | SL20.00 | adrenal cortico-steroid poisoning |
| 67525 | SL16.00 | anthelmintic drug poisoning |
| 67573 | SLE7000 | allopurinol poisoning |
| 67586 | U241.00 | [x]int self harm rifl s'gun/lrg frarm disch occ resid instit |
| 67658 | SL6z.00 | anticonvulsant or antiparkinsonian drug poisoning nos |
| 67676 | SLC6000 | clonidine poisoning |
| 67956 | U20C.12 | [x]self poisoning with paraquat |
| 67988 | TN05.00 | injury ?accidental, poisoning by drug or medicament nos |
| 68050 | SL92200 | trifluperidol poisoning |
| 68091 | U2A0.00 | [x]intentional self harm by blunt object occurrence at home |
| 68102 | U208z00 | [x]intent self poison oth/unsp drug/medic unspecif place |
| 68322 | SL04.00 | tetracycline group poisoning |
| 68714 | SL23.00 | insulins and antidiabetic poisoning |
| 68788 | U205z00 | [x]intent self poison narcotic drug unspecif place |
| 68790 | U202z00 | [x]intent self poison sedative hypnotic unspecif place |
| 68793 | U207.00 | [x]intent self poison/exposure to oth autonomic drug |
| 68798 | SL3..00 | poisoning by primarily systemic agents |
| 68806 | U20C.00 | [x]intent self poison/exposure to pesticide |
| 68963 | TN6..00 | injury ?accidental, by cutting and stabbing instruments |
| 69145 | ZX1K.12 | setting self alight |
| 69263 | U720.00 | [x]sequelae of intentional self-harm |
| 69277 | SL94400 | medazepam poisoning |
| 69342 | U2yz.00 | [x]intent self harm by oth specif means occ unspecif place |
| 69343 | U202y00 | [x]int self poison sedative hypnotic other spec place |
| 69407 | U409200 | [x]pois/exp ?intent alcohol school/pub admin area |
| 69422 | U1Ayz00 | [x]accid poison/expos to unspecif chemical unspecif place |
| 69499 | TN7z.00 | injury ?accidental, fall from high place nos |
| 69624 | SL53100 | phenylbutazone poisoning |
| 69628 | SLG5z00 | eye drug poisoning nos |
| 69793 | SL02000 | chloramphenicol poisoning |
| 69850 | SL35z00 | vitamin poisoning nos |
| 69867 | SLG6.00 | ear, nose and throat drug poisoning nec |
| 69969 | TK10.00 | suicide + selfinflicted poisoning by gas via pipeline |
| 70049 | TN08.00 | injury ?accidental, poisoning by arsenic or its compounds |
| 70391 | U205y00 | [x]int self poison narcotic drug other spec place |
| 70405 | TK1..00 | suicide + selfinflicted poisoning by gases in domestic use |
| 70410 | TN71.00 | injury ?accidental, fall from other man-made structure |
| 70414 | U20A400 | [x]int self poison org solvent,halogen hydrocarb,in highway |
| 70784 | SLAy.00 | other central nervous system stimulant poisoning |
| 70946 | TKx0.00 | suicide + selfinflicted injury-jump/lie before moving object |
| 71158 | SLG7.00 | topical dental drug poisoning |
| 71159 | TKx4.00 | suicide and selfinflicted injury by electrocution |
| 71375 | TK11.00 | suicide + selfinflicted poisoning by liquified petrol gas |
| 71432 | ZRn5.00 | suicide intent score subscale - risk |
| 71455 | SL94500 | nitrazepam poisoning |
| 71548 | 8G6Z.00 | anti-suicide psychotherapy nos |
| 71661 | ZX...11 | self-damage |
| 71781 | SLE7.00 | uric acid drug poisoning |
| 71789 | SL04z00 | tetracycline group poisoning nos |
| 71833 | SL54.00 | antirheumatic poisoning |
| 71843 | TKxz.00 | suicide and selfinflicted injury by other means nos |
| 71898 | SL1y100 | nitrofuran derivative poisoning |
| 71955 | 8G6..00 | anti-suicide psychotherapy |
| 72038 | SLC3.00 | ganglion-blocker poisoning |
| 72194 | SL31.00 | antineoplastic and immunosuppressive poisoning |
| 72213 | SL21.00 | androgen and anabolic poisoning |
| 72226 | SLC6.00 | other hypertensive agent poisoning |
| 72286 | SL20000 | cortisone derivative poisoning |
| 72559 | ZX19100 | punching self |
| 72627 | SyuFW00 | [x]poisoning by other laxatives, incl intestin atonia drugs |
| 72734 | U2B0.00 | [x]intent self harm by jumping from high place occ at home |
| 72747 | U270.00 | [x]intention self harm by smoke fire/flames occurrn at home |
| 72790 | SL12z00 | heavy metal anti-infective poisoning nos |
| 72792 | U206400 | [x]intent self pois hallucinogen in street/highway |
| 72893 | SL50000 | unspecified opium poisoning |
| 72957 | TN8y.00 | injury ?accidental, by other specified means |
| 73097 | TN72.00 | injury ?accidental, fall from natural site |
| 73212 | Sy...00 | [x] injury and poisoning classification terms |
| 73585 | U29y.00 | [x]intention self harm by sharp object occ oth specif place |
| 73603 | U28z.00 | [x]intent self harm by steam hot vapour/obj occ unspec place |
| 73628 | TK2z.00 | suicide + selfinflicted poisoning by gases and vapours nos |
| 73666 | U26..00 | [x]intentional self harm by explosive material |
| 73709 | SL30.00 | antiallergic and antiemetic drug poisoning |
| 73750 | U1A8100 | [x]accid poison/expos to oth/unsp drug/medicam res institut |
| 73776 | U207000 | [x]int self poison/exposure to oth autonomic drug at home |
| 73800 | U1A8z00 | [x]accid poison/expos to oth/unsp drug/medic unspecif place |
| 73825 | U2Cy.00 | [x]int self harm jump/lying bef mov obje occ oth specif plce |
| 87882 | U2y1.00 | [x]intent self harm by oth specif means occ resid instit'n |
| 89371 | ZX1Q.11 | jumping under train |
| 89429 | U20C.11 | [x]self poisoning with weedkiller |
| 89578 | ZX1H200 | self-suffocation |
| 90331 | SL8z.00 | local anaesthetic poisoning nos |
| 90440 | U294.00 | [x]intention self harm by sharp object occ street/highway |
| 90548 | U4z3.00 | [x]unspecif event undeterm intent occurrn sport/athlet area |
| 90857 | U21y.00 | [x]intent self harm by hangng strangul/suffoct oth spec plce |
| 91475 | U4D7.00 | [x]crashng of motor vehicle undetermined intent occ on farm |
| 92308 | ZX1R.00 | throwing self in front of vehicle |
| 92752 | SLC7000 | sodium morrhuate poisoning |
| 92907 | U1A3500 | [x]accid poison/expos antiparkinson drug trade/service area |
| 93052 | U410.00 | [x]hanging strangulat+suffocat undet intent occurrn at home |
| 93065 | SL52z00 | aromatic analgesic poisoning nos |
| 93208 | TN80100 | injury ?accidental, by lying before moving object |
| 93495 | 388s.00 | pierce suicide intent scale score |
| 93599 | SL07300 | daunorubicin poisoning |
| 93837 | U2C1.00 | [x]int self harm jump/lying befr mov obje occ resid instit'n |
| 93890 | SL24011 | acth - adrenocorticotropic hormone poisoning |
| 93908 | SL1x000 | ethambutol poisoning |
| 94066 | SL6x000 | primidone poisoning |
| 94223 | SC41.00 | late effect of poison due to nonmedical substance |
| 94377 | U2Bz.00 | [x]int self harm by jump from high place occ unspecif place |
| 94400 | SLF4z00 | antitussive poisoning nos |
| 94412 | TK1z.00 | suicide + selfinflicted poisoning by domestic gases nos |
| 94442 | TK01400 | suicide and self inflicted injury by phenobarbitone |
| 94471 | SL80z00 | central nervous system muscle-tone depressant poisoning nos |
| 94637 | U22y.00 | [x]intent self harm by drown/submersn occ oth specif place |
| 94644 | U20Cy00 | [x]int self poison pesticide other spec place |
| 94662 | U206.00 | [x]intent self poison/exposure to hallucinogen |
| 94705 | SL95z00 | tranquilliser poisoning nos |
| 94725 | U202.18 | [x]overdose - amobarbital |
| 95059 | SL20z00 | adrenal cortico-steroid poisoning nos |
| 95074 | U1Ay500 | [x]accid poison/expos unspecif chemical trade/service area |
| 95432 | SyuF.00 | [x]poisoning by drugs and biological substances |
| 95712 | ZX1B300 | jumping from cliff |
| 95790 | U2B6.00 | [x]int self harm by jump from high place indust/constr area |
| 95794 | U250.00 | [x]intent self harm oth/unspecif firearm disch occ at home |
| 96224 | U2zy.00 | [x]intent self harm by unspecif means occ oth specif place |
| 96430 | TKx7.00 | suicide and selfinflicted injury caustic subst, excl poison |
| 96651 | U200y00 | [x]int self poison nonopioid analgesic other spec place |
| 96687 | U204y00 | [x]int self poison psychotropic drug other spec place |
| 96714 | U202400 | [x]intent self pois sedative hypnotic in street/highway |
| 96728 | U200500 | [x]intent self pois nonopioid analgesic trade/service area |
| 96729 | U208400 | [x]intent self pois oth/unsp drug/medic in street/highway |
| 96730 | U200100 | [x]intent self poison nonopioid analgesic at res institut |
| 96740 | U20C000 | [x]int self poison/exposure to pesticide at home |
| 96753 | U208y00 | [x]int self poison oth/unsp drug/medic other spec place |
| 97085 | U209y00 | [x]int self poison alcohol other spec place |
| 97334 | U2z2.00 | [x]intent self harm by unspec mean occ sch/ins/pub adm area |
| 97430 | TN80000 | injury ?accidental, by jumping before moving object |
| 97590 | SLE3000 | benzothiazide poisoning |
| 97687 | SLC5200 | papaverine poisoning |
| 97788 | U404.00 | [x]poisoning/exposure, ? intent, to psychotropic drug |
| 97794 | U22z.00 | [x]intent self harm by drown/submersn occ unspecified place |
| 97817 | SLF6.00 | anti-common cold drug poisoning |
| 97845 | SL70z00 | barbiturate poisoning nos |
| 97943 | U20Az00 | [x]int self pois org solv,halogen hydrocarb, unspec place |
| 98325 | SLB2.00 | sympathomimetic poisoning |
| 98405 | SL14z00 | antimalarial drug poisoning nos |
| 98594 | TKx3.00 | suicide and selfinflicted injury by extremes of cold |
| 98607 | SC40.00 | late effect of poison drug/medicament/biological substance |
| 98768 | SL21200 | oxymetholone poisoning |
| 98865 | U40yz00 | [x]pois/expos ?intent to unspecif chemical unspecif place |
| 98955 | SL23100 | biguanide poisoning |
| 99005 | SL80.00 | central nervous system muscle-tone depressant poisoning |
| 99010 | SL96z00 | hallucinogen poisoning nos |
| 99011 | U204100 | [x]intent self poison psychotropic drug at res institut |
| 99049 | SyuG700 | [x]toxic effects of other specified gases, fumes & vapours |
| 99388 | SL31z00 | antineoplastic or immunosuppressive poisoning nos |
| 99427 | ZX1Q.00 | throwing self in front of train |
| 99566 | TK01100 | suicide and self inflicted injury by barbitone |
| 99678 | SyuFc00 | [x]poisoning by oth & unspecif drugs & biologic substances |
| 99775 | 14K1.00 | intentional overdose of prescription only medication |
| 99792 | U212.00 | [x]inten slf harm hang strang/suffc sch oth ins/pub adm area |
| 99845 | SL6x.00 | other anticonvulsant poisoning |
| 100372 | U4Bz.00 | [x]fall jump/push frm high plce undt intnt occ unspecif plce |
| 100635 | U2By.00 | [x]int self harm by jump from high place occ oth specif plce |
| 100700 | SLD3100 | poisoning by saline and osmotic laxatives |
| 100884 | SL3y.00 | other systemic agent poisoning |
| 101056 | TK5z.00 | suicide and selfinflicted injury by firearms/explosives nos |
| 101289 | SLB1400 | caramiphen poisoning |
| 101481 | U200400 | [x]intent self pois nonopioid analgesic in street/highway |
| 101872 | SL23000 | acetohexamide poisoning |
| 101906 | TKx6.00 | suicide and selfinflicted injury by crashing of aircraft |
| 101971 | U2D6.00 | [x]intent self harm crash motor vehic occ indust/constr area |
| 102047 | SL40z00 | iron and iron compound poisoning nos |
| 102060 | SL35000 | vitamin a poisoning |
| 102454 | U4B6.00 | [x]fall jump/push frm high plce undt intn indust/constr area |
| 102479 | U206000 | [x]int self poison/exposure to hallucinogen at home |
| 102607 | ZRn6.11 | srs - suicide risk scale |
| 102731 | U40y000 | [x]poison/exposure ?intent, to unspecif chemical at home |
| 102790 | U220.00 | [x]intent self harm by drowning/submersion occurrn at home |
| 102975 | U40yy00 | [x]pois/exp ?intent to unspecif chemical other spec place |
| 103014 | SLE6.00 | other mineral salt poisoning nec |
| 103422 | SL24211 | growth hormone poisoning |
| 103643 | 1JP..00 | suspected drug overdose |
| 104233 | U216.00 | [x]intent self harm by hang strangl/suffc indust/constr area |
| 104485 | 1BDE.00 | suicide risk increased from previous level |
| 104754 | TN02.00 | injury ?accidental, poisoning by other sedative/hypnotic |
| 104834 | TK2y.00 | suicide + selfinflicted poisoning by other gases and vapours |
| 105003 | U4z..00 | [x]unspecified event, undetermined intent |
| 105319 | SL22200 | combined oestrogen and progesterone poisoning |
| 105322 | SLC7100 | zinc salt poisoning |
| 105468 | U4A6.00 | [x]contct wth blunt obj undet intent occ industr/constr area |
| 105551 | SL5y200 | antipyretic poisoning, nec |
| 105584 | U2y6.00 | [x]intent self harm oth specif means occ indust/constr area |
| 105934 | SLG..00 | eye, otorhinolaryngological, skin and dental drug poisoning |
| 106028 | U200600 | [x]int self pois nonopioid analgesic indust/construct area |
| 106178 | TK01z00 | suicide and self inflicted injury by barbiturates |
| 106180 | TKx0z00 | suicide + selfinflicted inj-jump/lie before moving obj nos |
| 106258 | SLA0000 | lobeline poisoning |
| 106714 | U2Dz.00 | [x]intent self harm by crash motor vehic occ unspecif place |
| 107092 | U1Ay100 | [x]accid poison/expos to unspecif chemical at res institut |
| 107121 | U2yy.00 | [x]intent self harm oth specif means occ oth specif place |
| 107902 | SL14400 | pyrimethamine poisoning |
| 108187 | TK53.00 | suicide and selfinflicted injury by military firearms |
| 108448 | SL35100 | vitamin d poisoning |
| 108499 | U2Az.00 | [x]intentional self harm by blunt object occ unspecif place |
| 108532 | SL74.00 | methaqualone compound poisoning |
| 108575 | SyuFB00 | [x]poisoning by other opioids |
| 108654 | TN3y.00 | injury ?accidental, other means of hang/strangle/suffocate |
| 109625 | U296.00 | [x]intent self harm by sharp object occ indust/constr area |
| 110088 | U408400 | [x]pois/expos ?intent oth/unsp drug/medic in street/highway |
| 110167 | 9j2..00 | initiation of suicide risk management document |
| 110215 | SLF1.00 | smooth muscle relaxant poisoning |
| 110371 | 38B9.00 | assessment for risk of self harm |
| 110444 | 679j200 | educatn abt take home naloxone fr opiate overdose interventn |
| 110550 | SL85300 | tetracaine poisoning |
| 110578 | U27y.00 | [x]intent self harm by smoke fire/flame occ oth specif plce |
| 110585 | ZRn4.00 | suicide intent score subscale - self report |
| 110830 | SyuF900 | [x]poisoning by oth nonsteroidal anti-inflamm drugs [nsaid] |
| 110849 | SL31500 | fluorouracil poisoning |
| 110865 | U206z00 | [x]intent self poison hallucinogen unspecif place |
| 110884 | U295.00 | [x]intent self harm by sharp object occ trade/service area |
| 110900 | U292.00 | [x]intent self harm sharp obj occ sch oth ins/pub adm area |
| 110988 | SL42200 | phenindione poisoning |
| 111004 | U213.00 | [x]intent self harm by hang strangl/suffc sport/athlet area |
| 111117 | U2z1.00 | [x]intent self harm by unspecif means occurrn resid instit'n |
| 111291 | U2B1.00 | [x]intent self harm by jump from high place occ resid instit |
| 111381 | U282.00 | [x]int self harm by steam hot vapor/obj sch/ins/pub adm area |
| 111420 | U202.14 | [x]overdose - flurazepam |
| 111553 | SLC0300 | quinidine poisoning |
| 111573 | SL16100 | piperazine poisoning |
| 111639 | U4Ay.00 | [x]contact with blunt obj undeter intent occ oth specif plce |
| 111721 | U2A2.00 | [x]intent self harm blunt obj occ sch oth ins/pub adm area |
| 111829 | U40Bz00 | [x]pois/expos ?intent to other gas/vapour unspecif place |
| 112444 | SL2z.00 | hormone or synthetic substitute poisoning nos |
| 112519 | SLB0z00 | cholinergic poisoning nos |
| 112611 | SyuFT00 | [x]poisoning by other antihypertensive drugs, nec |
| 112645 | U209400 | [x]intent self pois alcohol in street/highway |
| 112646 | U20Ay00 | [x]int self pois org solv,halogen hydrocarb,oth spec place |
| 112693 | SL25.00 | posterior pituitary hormone poisoning |
| 112802 | U41z.00 | [x]hangng strangult+suffoct undet intent occ unspecif place |
