# Supplementary material for: Associations between breast cancer survivorship and adverse mental health outcomes: A matched population-based cohort study in the United Kingdom
Source: PLoS Med. 2021 Jan 7;18(1):e1003504. doi: 10.1371/journal.pmed.1003504 (PMC7822529; doi:10.1371/journal.pmed.1003504)
Supplement: S1 Protocol — (DOCX) [file pmed.1003504.s004.docx]

**PART 2: PROTOCOL INFORMATION**

| **Applicants must complete all sections listed below**  **Sections which do not apply should be completed as ‘*Not Applicable’* and justification provided** |
| --- |
| Study Title (Max. 255 characters) **Risk of adverse mental health outcomes in women with a history of breast cancer in the United Kingdom: a matched population-based cohort study** |
| Lay Summary (Max. 250 words) Women with a history of breast cancer are the largest group of cancer survivors in the general population. A breast cancer diagnosis may impact on mental health, and breast cancer treatments, which are necessary to control the disease, can result in side effects that may negatively affect the women’s quality of life. This study aims to understand whether women who have had breast cancer have different mental health several years post-treatment, compared to women who did not have cancer. For this, we will compare the risk of being diagnosed with anxiety and depression, the primary outcomes of this study, in women who have had breast cancer and in women who never had cancer, attending general practitioner (GP) practices in the UK. We will also compare GP recorded declines in the patients’ memory and thinking capacities, feelings of tiredness and weakness (fatigue), pain, insomnia, sexual problems, or self-harm and suicide (secondary outcomes), between the two groups, and explore factors that may be associated with increased risk of these outcomes. The results of this study can be used to better understand the needs of the women who carry on lives beyond breast cancer. |
| Technical Summary (Max. 300 words) The aim of this study is to estimate the relative risk of anxiety and depression (primary outcomes), and fatigue, pain, sleep disorders, neurocognitive and sexual dysfunctions, and fatal and non-fatal self-harm (secondary outcomes), in breast cancer survivors compared to non-cancer controls. This study will be a matched cohort study, utilising data from the CPRD GOLD primary care database. Outcome-specific algorithms will be developed and validated to identify outcomes in the data. Algorithm development will consider Read codes for diagnoses, prescriptions, referrals and symptoms; prevalence and incidence estimates by age-group and sex will be computed for a random sample of patients selected from CPRD GOLD primary care database. Validation will be against external sources of data, namely published data from population-based surveys in the UK. To estimate the associations between breast cancer survivorship and the primary and secondary outcomes, we will identify all women exposed to breast cancer in the CPRD GOLD primary care database, and randomly select an age- and primary-care-practice-matched cohort of women without prior cancer in a ratio of 1:4. Cox regression models will be used to estimate hazard ratios adjusted for important confounders, and to explore the role of effect modifiers; the proportionality of hazards will be tested graphically and inferentially. |
| Outcomes to be Measured  - Anxiety - Depression - Fatigue - Cognitive impairment - Pain - Sexual dysfunction - Sleep disorders - Completed suicide - Self-harm |
| Objectives, Specific Aims and RationaleGeneral objective To quantify the relative risk of common adverse mental health outcomes in breast cancer survivors compared to women who did not have cancer in the United Kingdom. Specific aims  1. To develop and validate algorithms to identify patients with anxiety and depression (primary outcomes), and fatigue, mild cognitive impairment, pain, sleep disturbance, sexual dysfunction, and fatal and non-fatal self-harm (secondary outcomes) in the CPRD GOLD primary care database. 2. To compare the risk of developing anxiety and depression (primary outcomes), and of recorded fatigue, mild cognitive impairment, pain, sleep disturbance, sexual dysfunction, and fatal and non-fatal self-harm (secondary outcomes), between women with a history of breast cancer and women who did not have cancer. 3. To estimate association between breast cancer history and anxiety/depression by presence of common complications of the breast cancer treatments (i.e. lymphoedema, pain, mild cognitive impairment, fatigue, sexual dysfunction, sleep disorders) and exposure to endocrine therapy.  Rationale It is currently unclear if the long-term mental health of breast cancer survivors differs from that of comparable women who never had cancer. This study will directly address this evidence gap, and help inform prevention and treatment needs relating to the mental health of breast cancer survivors. |
| Study Background Women with a history of breast cancer are the largest group of cancer survivors in the United Kingdom (UK). Approximately 570,000 women were estimated to be living beyond a breast cancer diagnosis in 2010; this figure was projected to rise to1.5 million women by 2040 [1].  Evidence on the mental health of breast cancer survivors in the UK suggests high levels of distress in this group. Capelan et al [2] reported that 60% of women post treatment for early breast cancer had ≥1 unmet needs; the most common were hot flushes, fatigue, pain, worry, fear or anxiety, and sleep problems. A third of the women who participated in the Standardisation of Radiotherapy Trial (START) [3] had relevant symptoms of anxiety at baseline; five years later, this proportion was 29%. Similar results were observed for depression, albeit the absolute frequency was lower: 12% scored above normal level at baseline, and 11% at the five-year evaluation [3]. The worse recollections reported by a sample of women in the UK seven years after diagnosis included the anxiety related to the future’s uncertainty (38%), the chemotherapy and related side effects (25%), and the shock of the cancer diagnosis (18%) [4]. Other reported concerns were the breast removal and body image implications in sexuality (8%), the suffering induced by their disease in their loved ones (7%), the co-morbidities (6%), and the side effects of radiotherapy and hormone therapy (6%) [4]. All of these may negatively affect the women’s mental status. Indeed, a study on the quality of life of breast cancer survivors one to five years post-diagnosis in England described lower scores than what had been described in other studies of the general population [5].  In the UK, women post-treatment for breast cancer are often followed in hospital outpatient clinics, where they may receive psychological support, and in primary care [6]. In the latter setting, evidence on the relative risk of adverse mental health outcomes in breast cancer survivors, compared to women who did not have cancer, is scant. A systematic review of quantitative studies that evaluated adverse mental health outcomes in breast cancer survivors and in the background female population identified one single study from the UK. In this study, Khan et al [7] used routinely collected primary care data to study the pattern of consultations and prescriptions for anxiety and depression in women with a history of breast cancer for ≥5 years. The results showed significantly increased odds of being prescribed with antidepressants or anxiolytics, even though there was no strong statistical evidence of increased odds of consulting for these conditions. The frequency of anxiety and depression among women diagnosed at <5 years is unknown. Population-based studies conducted elsewhere [8-12] reported highest risks of anxiety and depression shortly after the breast diagnosis, which declined over time. It is currently unclear if this same pattern is observed in the UK. In addition, the studies identified in the systematic review suggest that breast cancer survivors may be at increased risk of other outcomes, such as sleep disturbance, neurocognitive and sexual dysfunctions; no study addressed these in population-based samples of breast cancer survivors in the UK. Furthermore, to our knowledge, the frequency of fatigue, pain, and fatal and non-fatal self-harm, in breast cancer survivors in the UK is unknown, even though these relate to unmet needs often reported by breast cancer survivors. The Clinical Practice Research Datalink primary care database includes data prospectively collected on symptoms, diagnoses, prescriptions, and referrals, for over 5.8 million women being followed in primary care since the late 1980s [13], and therefore it represents a unique opportunity to assess the risk of these outcomes at population-level.  The aim of this study is to quantify the relative risk of adverse mental health outcomes in women with a history of breast cancer the UK, compared to women with no cancer background. The primary outcomes will be anxiety and depression, two common mental disorders that are commonly managed in primary care settings. Secondary outcomes will be fatigue, mild cognitive impairment, pain, sleep disorder, sexual dysfunction, and fatal and non-fatal self-harm; part of the contribution of this study will be to establish the feasibility or otherwise of using electronic health records to assess some of these less-studied outcomes. |
| Study Type Hypothesis testing  Study null hypothesis: There are no differences in the risks of anxiety and depression (primary outcomes), fatigue, mild cognitive impairment, pain, sleep disturbance, sexual dysfunction, and fatal and non-fatal self-harm (secondary outcomes), between women with a history of breast cancer and women who never had cancer receiving primary care in the UK. |
| Study Design The research aims will be addressed with a matched cohort study design.  Two cohorts will be assembled from the CPRD GOLD primary care database:  (1) The exposed cohort will include women diagnosed with a breast cancer (list of Read codes available in appendix 2) after at least 12 months of uninterrupted up-to-standard follow-up in CPRD (to ensure that the breast cancer is an incident event).  (2) A comparison cohort will be assembled by randomly selecting, for each woman with a breast cancer diagnosis, up to 4 women of similar age (3-year range), attending the same GP practice and with at least 12 months of uninterrupted up-to-standard data quality for research, but with no history of cancer at the date of the breast cancer diagnosis of the matched breast cancer patient.  The index date will be the date of breast cancer diagnosis for the exposed group; comparison patients will take the same index date as their exposed match. Please see section L for more details.  Inclusion criteria for both cohorts are: female sex, aged ≥18 years, and having a clinical record with at least 12 months of uninterrupted up-to-standard data quality for research (as measured by CPRD) before the breast cancer diagnosis date (to ensure the cancer record represents incident disease). Exclusions will be the diagnosis of severe mental illness (i.e. organic mental disorder, mental disorders due to substances, schizophrenia, delusional disorders, or manic or bipolar episodes), having a history of the specified mental health outcome in the year before index date; and having had a diagnosis of any other cancer prior to the index date.  All women will be followed from the index date until the earliest date of: outcome observed, a cancer diagnosis, death recorded, transference out of the practice; last data collection for the practice.  Matching will allow close control of key covariates, include GP practice that is difficult to adjust for in a statistical model (too many levels), and has the practical advantage of reducing the size of the comparison group (which might otherwise include several million women) by restricting to the most relevant comparison patients. |
| Feasibility counts Feasibility counts presented below are based on the January 2018 version of the CPRD GOLD primary care database.  We identified 65,136 women who had a diagnosis of breast cancer (Read codes provided in appendix 2) while aged between 18 and 80 years, registered with a primary care practice contributing with data to CPRD, and whose individual records were acceptable for research. Non-interrupted one year of follow up before the cancer diagnosis (index date) was not available for 6,757 women, and 6,044 women were further excluded because they had a lifetime diagnosis of severe mental illness or another cancer before their breast cancer diagnosis (see section J for the inclusion and exclusion criteria).  Hence, 52,335 women with a history of breast cancer were identified as eligible for this study; the table below provides details of the distribution by age and calendar period of diagnosis.  Table 1 Distribution of women with breast cancer history who are eligible for this study, by age and calendar period of diagnosis.   \|  \| **N** \| **%** \| \| --- \| --- \| --- \| \|  \|  \|  \| \| **All study participants** \| **52,335** \| **(100.0)** \| \|  \|  \|  \| \| **Calendar period of diagnosis** \|  \|  \| \| 1989-1994 \| 3,184 \| (6.1) \| \| 1995-1999 \| 4,768 \| (9.1) \| \| 2000-2004 \| 10,934 \| (20.9) \| \| 2005-2009 \| 14,705 \| (28.1) \| \| 2010-2014 \| 13,805 \| (26.4) \| \| ≥2015 \| 4,939 \| (9.4) \| \|  \|  \|  \| \| **Age at diagnosis (years)** \|  \|  \| \| 18-24 \| 24 \| (0.1) \| \| 25-34 \| 771 \| (1.5) \| \| 35-44 \| 4,916 \| (9.4) \| \| 45-54 \| 12,235 \| (25.3) \| \| 55-64 \| 14,910 \| (28.5) \| \| 65-74 \| 12,811 \| (24.5) \| \| 75-80 \| 5,668 \| (10.8) \| \|  \|  \|  \|   A comparison group of women without cancer (4 controls per breast cancer case) will be randomly selected from the same data source, same primary care practice and within a 3-year age range.  Appendix 2 provides the list of Read codes used to identify women with a history of breast cancer in the CPRD GOLD primary care database. The list of Read codes used to identify cancer diagnoses other than the breast one (exclusion criterion) has been published elsewhere [14]. A provisional list of Read codes to identify women with several mental illnesses (exclusion criterion) was defined for the purpose of this calculation; the final list will be refined in due course. |
| Sample size considerations Table 2 shows the minimum relative risk that could be detected with the 52,335 women identified in the CPRD GOLD primary care database, for different probabilities of type I (α) and type II error (β). These estimates were obtained with the command ‘power’ in Stata v15 [15].  Table 2 Minimum RR that can be detected with the 52,355 women with a history of breast cancer and 209,420 women who did not have cancer, for different probabilities of type I and type II errors, and baseline risk of the outcomes.   \| **Outcome** \| **α** \| **β** \| **% of outcome in unexposed group [ref]** \| **Min. HR possible to be estimated** \| \| **RR/HR estimated in other studies [ref]** \| \| \| --- \| --- \| --- \| --- \| --- \| --- \| --- \| --- \| \|  \|  \|  \|  \|  \| \|  \| \| \| **Primary outcomes** \|  \|  \|  \|  \| \|  \| \| \|  \|  \|  \|  \|  \| \|  \| \| \| **Anxiety, diagnoses** \| **0.05** \| **0.20** \| **5 [7]** \| **1.05** \| \| 1.06 [7]  1.08 [7]  1.22 [11]  1.25 [8] \| \| \| **0.01** \| **0.20** \| **5 [7]** \| **1.06** \| \| \| **0.05** \| **0.10** \| **5 [7]** \| **1.06** \| \| \| 0.01 \| 0.10 \| 5 [7] \| 1.07 \| \| \|  \|  \|  \|  \| \| \| **0.05** \| **0.20** \| **20 [11]** \| **1.02** \| \| \| **0.01** \| **0.20** \| **20 [11]** \| **1.03** \| \| \| **0.05** \| **0.10** \| **20 [11]** \| **1.03** \| \| \| **0.01** \| **0.10** \| **20 [11]** \| **1.03** \| \| \|  \|  \|  \|  \|  \| \|  \| \| \| **Anxiety, prescription of**  **anxiolytics** \| **0.05** \| **0.20** \| **3 [10]** \| **1.07** \| \| 1.08 [7]  2.52 [10] \| \| \| **0.01** \| **0.20** \| **3 [10]** \| **1.08** \| \| \| **0.05** \| **0.10** \| **3 [10]** \| **1.08** \| \| \| 0.01 \| 0.10 \| 3 [10] \| 1.09 \| \| \|  \|  \|  \|  \| \| \| **0.05** \| **0.20** \| **8 [7]** \| **1.04** \| \| \| **0.01** \| **0.20** \| **8 [7]** \| **1.05** \| \| \| **0.05** \| **0.10** \| **8 [7]** \| **1.05** \| \| \| **0.01** \| **0.10** \| **8 [7]** \| **1.06** \| \| \|  \|  \|  \|  \|  \| \|  \| \| \| **Depression, diagnoses** \| 0.05 \| 0.20 \| 3 [11] \| 1.07 \| \| 1.06 [7]  1.39 [9]  1.49 [8]  1.94 [11] \| \| \| 0.01 \| 0.20 \| 3 [11] \| 1.08 \| \| \| 0.05 \| 0.10 \| 3 [11] \| 1.08 \| \| \| 0.01 \| 0.10 \| 3 [11] \| 1.09 \| \| \|  \|  \|  \|  \| \| \| **0.05** \| **0.20** \| **9 [7]** \| **1.04** \| \| \| **0.01** \| **0.20** \| **9 [7]** \| **1.05** \| \| \| **0.05** \| **0.10** \| **9 [7]** \| **1.04** \| \| \| **0.01** \| **0.10** \| **9 [7]** \| **1.05** \| \| \|  \|  \|  \|  \|  \| \|  \| \| \| **Depression, prescription of**  **antidepressants** \| **0.05** \| **0.20** \| **2 [10]** \| **1.08** \| \| 1.16 [7]  1.95 [10] \| \| \| **0.01** \| **0.20** \| **2 [10]** \| **1.10** \| \| \| **0.05** \| **0.10** \| **2 [10]** \| **1.09** \| \| \| **0.01** \| **0.10** \| **2 [10]** \| **1.11** \| \| \|  \|  \|  \|  \| \| \| **0.05** \| **0.20** \| **20 [7]** \| **1.02** \| \| \| **0.01** \| **0.20** \| **20 [7]** \| **1.03** \| \| \| **0.05** \| **0.10** \| **20 [7]** \| **1.03** \| \| \| **0.01** \| **0.10** \| **20 [7]** \| **1.03** \| \| \|  \|  \|  \|  \|  \| \|  \| \| \| **Secondary outcomes *** \|  \|  \|  \|  \| \|  \| \| \|  \|  \|  \|  \|  \| \|  \| \| \| **Sexual dysfunction** \| 0.05 \| 0.20 \| 4.1 [16] \| 1.06 \| \| 1.03 [16]  2.27 [17] \| \| \| 0.01 \| 0.20 \| 4.1 [16] \| 1.07 \| \| \| 0.05 \| 0.10 \| 4.1 [16] \| 1.07 \| \| \| 0.01 \| 0.10 \| 4.1 [16] \| 1.08 \| \| \|  \|  \|  \|  \| \| \| 0.05 \| 0.20 \| 9.1 [17] \| 1.04 \| \| \| 0.01 \| 0.20 \| 9.1 [17] \| 1.05 \| \| \| 0.05 \| 0.10 \| 9.1 [17] \| 1.04 \| \| \| 0.01 \| 0.10 \| 9.1 [17] \| 1.05 \| \| \|  \|  \|  \|  \|  \| \|  \| \| \| **Suicide** \| 0.05 \| 0.20 \| 0.0008** [18] \| 1.47 \| \| 1.37 [18]  1.6 [19] \| \| \| 0.01 \| 0.20 \| 0.0008** [18] \| 1.60 \| \| \| 0.05 \| 0.10 \| 0.0008** [18] \| 1.57 \| \| \| 0.01 \| 0.10 \| 0.0008** [18] \| 1.70 \| \| \|  \|  \|  \|  \|  \| \|  \| \| \| **Fatal and**  **non-fatal self-harm** \| 0.05 \| 0.20 \| 0.005 [18, 20] \| 1.17 \| \| 1.03 [21]  1.37 [18] \| \| \| 0.01 \| 0.20 \| 0.005 [18, 20] \| 1.21 \| \| \| 0.05 \| 0.10 \| 0.005 [18, 20] \| 1.20 \| \| \| 0.01 \| 0.10 \| 0.005 [18, 20] \| 1.24 \| \| \|  \|  \|  \|  \|  \| \|  \| \| \| **Sleep disturbances** \| 0.05 \| 0.20 \| 50 [22] \| 1.01 \| \| 0.8 [22] \| \| \| 0.01 \| 0.20 \| 50 [22] \| 1.02 \| \| \| 0.05 \| 0.10 \| 50 [22] \| 1.02 \| \| \| 0.01 \| 0.10 \| 50 [22] \| 1.02 \| \| \|  \|  \|  \|  \|  \| \|  \| \| \| **Prescription of hypnotics** \| **0.05** \| **0.20** \| **4 [23]** \| **1.06** \| \| 3.75 [23] \| \| \| **0.01** \| **0.20** \| **4 [23]** \| **1.07** \| \| \| **0.05** \| **0.10** \| **4 [23]** \| **1.07** \| \| \| **0.01** \| **0.10** \| **4 [23]** \| **1.08** \| \| \|  \|  \|  \|  \|  \| \|  \| \| \| **Mild cognitive impairment** \| **0.05** \| **0.20** \| **5 [24]** \| **1.05** \| \| 1.58 [25]  1.60 [26]  2.43 [24]  3.67 [27] \| \| \| **0.01** \| **0.20** \| **5 [24]** \| **1.06** \| \| \| **0.05** \| **0.10** \| **5 [24]** \| **1.06** \| \| \| **0.01** \| **0.10** \| **5 [24]** \| **1.07** \| \| \|  \|  \|  \|  \| \| \| **0.05** \| **0.20** \| **19 [26]** \| **1.02** \| \| \| **0.01** \| **0.20** \| **19 [26]** \| **1.03** \| \| \| **0.05** \| **0.10** \| **19 [26]** \| **1.03** \| \| \| **0.01** \| **0.10** \| **19 [26]** \| **1.04** \| \| \|  \|  \|  \|  \| \|  \| \|  \| \|   HR – hazard ratio. RR – risk ratio. Bold is used to denote where the minimum risk ratio that could be detected with the available sample size is lower than the lowest estimate reported in the literature.  * No population-based studies have been identified reporting the relative risk of sexual dysfunctions, pain, fatigue, sleep disorders or cognitive dysfunction in breast cancer survivors compared to the non-cancer female population. Thus, the relative risk reported in the column for the other studies comes from studies involving convenience samples of cancer survivors. Pain and fatigue are often evaluated using psychometric instruments whose mean scores are summarised as means for between-group comparisons; no studies were identified providing data for the prevalence of pain and fatigue in breast cancer survivors and in women who did not have cancer, and thus these two outcomes were not included in the table.  ** Calculated as the proportion of suicides in the exposed group divided by the inverse of the standardised mortality ratio reported in the original study.  The available sample size is expected to be sufficient for detecting clinically significant increases of anxiety and depression (primary outcomes) in breast cancer survivors compared to the women who did not have cancer.  For the secondary outcomes, sample size is likely to be enough to assess differences in sleep disturbance measured by hypnotics’ prescription, and mild cognitive impairment. However, the available sample size will have relatively small power to detect small differences in suicide between women with history of breast cancer and those who did not have cancer, because this is a rare outcome, but we will still have enough power to detect associations of a magnitude seen in some previous studies; data from our analysis can also contribute to future meta-analyses. |
| Planned use of linked data (if applicable): The following linkages will be requested:  Death registration data from the Office for National Statistics (ONS)  HES Admitted Patient Care (HES-APC)  Index of Multiple Deprivation 2015 (IMD), practice and patient level  Data coming from these data sets will supplement information available in the CPRD GOLD primary care database, but linked data will be used in sensitivity analysis only.  Data from the ONS-mortality and HES-APC databases will be important to increase the completeness and validity of some outcomes. For example, for suicide, only 26% of the suicides registered in the ONS mortality data (gold standard) were captured in CPRD, indicating low sensitivity of this source [28]. Similarly for non-fatal self-harm, only 68% of the cases registered in HES-APC could be identified in CPRD [28]. Of note, patients who had the outcome recorded in the year prior to the index date will be excluded, and thus this finer definition of the outcomes will impact patients’ selection and patients’ who are identified as having had the outcome. Information on the exposure will not be supplemented by data from HES-APC.  Patient-level quintiles of IMD will be used to control for socioeconomic status, which is a major confounder of the association between breast cancer history and adverse mental health outcomes. Even though women in the comparison group are selected from the same primary care practice of the index-case, and thus IMD at practice level will not vary by matched set, the patient-level IMD will allow for a finer adjustment of socioeconomic status. Practice level of IMD is requested to allow us to study effect modification in the full dataset.  We acknowledge that analyses including linked data will be restricted to the subset of practices and patients who consent to the linkage scheme (~75% of the practices in England); the coverage periods for the dataset will be taken into account (see table below). This will result in reductions in sample size and potentially impact the power of the study to reject the null-hypothesis. Thus, these data will be used in sensitivity analysis only.  Table 3 Coverage period of the data included in the databases that will be linked to the CPRD GOLD primary care database.   \| **Database** \| **Coverage period** \| \| --- \| --- \| \| HES Admitted Patient Care (APC) \| April 1997 – December 2017 \| \| ONS death registration \| January 1998 – February 2018 \| |
| Definition of the Study population Breast cancer cohort  The study population will consist of all adult women recorded in the CPRD GOLD primary care database as having had an incident breast cancer (Read codes provided in appendix 2) diagnosed during up-to-standard follow-up and prior to the most recent version of the CPRD GOLD primary care database available after all approvals have been obtained.  Inclusion criteria:   1. Female sex, aged ≥18 years; 2. Recorded with a breast cancer diagnosis during CPRD follow-up; 3. Clinical record with at least 12 months of uninterrupted up-to-standard data quality for research (as measured by CPRD) before the breast cancer diagnosis date (to ensure the cancer record represents incident disease).   Exclusion criteria:   1. Diagnosis of severe mental illness before the breast cancer diagnosis (i.e. organic mental disorder, mental disorders due to substances, schizophrenia, delusional disorders, or manic or bipolar episodes) (a provisional list of Read codes was defined for feasibility counts); 2. History of the specified mental health outcome in analysis in the year before the breast cancer diagnosis (list of Read codes to be defined in objective 1 of this study); 3. Diagnosis of any other cancer prior to breast cancer (Read codes available from Ranopa et al. [14]). |
| Selection of comparison group(s) or controls Non-cancer comparison cohort  A comparison cohort will be assembled by randomly selecting, for each index case, up to 4 women of similar age (3-year range), attending the same GP practice, but with no history of cancer at the index date. Matching will also consider the eligibility of the patients’ data for linkage, to ensure that matched-sets have the same probability of having had the information recorded when conducting sub-set analysis using linked data. Controls will be selected using nearest neighbour matching methods without replacement [29]. Women in the non-cancer comparison cohort who meet one or more exclusion criteria will be excluded, as well as their index case.  Women diagnosed with breast cancer during the follow up period will be censored from the unexposed group at the date of the cancer diagnosis, but will be eligible to separately contribute in the exposed group from this date (with corresponding unexposed matches). |
| Exposures, Outcomes and CovariatesExposure: breast cancer Women will be considered exposed at the day of the breast cancer diagnosis (index date), denoted by the first entry of one or more of the Read codes provided in appendix 2. Primary outcomes: anxiety and depression To our knowledge, there is no validated list of Read codes to identify anxiety and depression in the CPRD GOLD primary care database. Algorithms will be developed and tested to identify anxiety and depression cases in CPRD. The algorithms will be chiefly determined by clinical diagnoses of anxiety and depression registered in the EHR with Read codes, and supplemented with information from drug prescriptions, referrals, and symptoms (if deemed suitable). Please see section N, plan of analysis for specific aim 1, for more details on the construction and validation of algorithms. Secondary outcomes: fatigue, mild cognitive impairment, pain, sleep disturbance, sexual dysfunction, and fatal and non-fatal self-harm Similarly, we will develop and test outcome-specific algorithms that identify events using a hierarchy of data on clinical diagnoses, prescriptions, referrals and symptoms.  In sensitivity analysis, we will consider linked data (see section J for data linkage requested) to develop more precise definitions of the outcomes, when possible. For example, two definitions of suicide will be considered: (1) considering Read codes only to identify suicides in CPRD; (2) considering Read codes for suicide as well as death registration data where suicide was recorded as primary cause of death (ICD-10 codes provided in appendix 3) Covariates Variables considered as potential confounders or effect modifiers of the association between breast cancer history and anxiety and depression (the primary outcomes of this study) are described below. The directed, acyclic graph (DAG) in appendix 4 explicitly describes the assumptions of the causal relations between the variables that underpin the choice. Please refer to the data analysis (section N) for details on which variables will be considered for sensitivity analyses only. *Potential confounders*  - Age at diagnosis (categorical variable, in 10-year age bands)   Defined as the absolute difference between the year of breast cancer diagnosis and year of birth. Age is a strong risk factor for breast cancer and for mental disorders.   - Alcohol drinking habits at diagnosis (categorical variable: current drinker, former drinker, never drinker)   Excess alcohol drinking is a well-established risk factor for breast cancer [30], besides being positively associated with anxiety and depression [31-33]. The most recent data on alcohol drinking habits prior to index date will be used in analysis. Current drinking will be further sub-divided into high, moderate, low or unknown intake of alcohol.   - Body mass index at diagnosis (categorical variable: underweight, normal weight, overweight, obesity class I, obesity class II and above)   Calculated as BMI=(weight/height^2^). Higher body mass index is protective against breast cancer in pre-menopausal women, but a risk factor for breast cancer post-menopause [34]. Obesity increases the risk of anxiety and depression [35, 36]. The most recent recording of BMI prior to index date will be used in analysis. The BMI values will be categorised into 5 categories: <18.5 kg/m^2^ (underweight), 18.5-24.9 kg/m^2^ (normal weight), 25.0-29.9 kg/m^2^ (overweight), 30.0-34.9 kg/m^2^ (obesity class I), ≥35.0 kg/m^2^ (obesity class II and above). Read codes for body weight categories recorded in the year prior to the breast cancer diagnosed will be considered to supplement missing information for this variable.   - Calendar period of diagnosis (categorical variable: ≤1994; 1995-1999; 2000-2004; 2005-2009; 2010-2014; >2014)   Even though calendar time *per se* does not change the risk of breast cancer, the risk of being diagnosed with breast cancer changed over time, probably due to increase awareness of the disease along with widespread use of mammography to screen for breast cancer. Time has also contributed for mental disorders being more likely to be diagnosed, due to raises awareness and increased recognition of the importance of mental disorders among health care professionals.   - Diabetes mellitus (dichotomous variable: yes/no)   Diabetes mellitus has been associated with an increased risk of breast cancer [37] and depression [38]. Algorithms previously defined elsewhere will be applied to identify patients with diabetes mellitus in the CPRD GOLD primary care database [39, 40].   - Level of deprivation (categorical variable: quintiles of patient-level IMD).   The IMD is an ecological measure of deprivation for small areas in England that combines information from seven domains (income, employment, education, health deprivation, crime, barriers to housing, and living environment), and ranks the small area from 1 (most deprived) to 32,844 (least deprived). Patients will be categorised in quintiles of IMD, with quintile 1 representing those least deprived and quintile 5 those most deprived.   - Menopausal status (dichotomous variable: premenopausal/postmenopausal)   Menopausal status is a potential confounder of the association between breast cancer history and depression, as the risk of breast cancer increases with menopause [30], and so does the risk of depression [42]. However, information on menopausal status is not easily available in the CPRD database. We will therefore produce results stratified by an age cut-off, as a proxy of the menopausal status of the women. The cut-off will be the mean/median age at natural menopause in the UK.   - Smoking history at diagnosis (categorical variable: current smoker, former smoker, never smoker)   Information on Read codes available on the data will be used to classify patients by smoking history. The most recent data on smoking prior to index date will be used in analysis. *Potential effect modifiers*  - Living alone (dichotomous variable: yes/no)   Ascertained from the CPRD GOLD primary care data using Read codes (list provided in [41]) and the patient’s family number (variable ‘famnum’ from the patient file). Women living in household of <2 people will be classified as living alone.   - Residing in a care home (dichotomous variable: yes/no).   This variable will be defined from the CPRD GOLD primary care database, using Read codes (list provided in [41]), and information gathered in the family number variable (‘famnum’). For the latter, ‘care home’ will be defined as a household with >3 individuals aged ≥65 years and if their total count was more than of individuals <65 years.   - Ethnicity (categorical variable: White, South Asian, Black, Others and mixed)   Ethnicity data recorded in the CPRD GOLD primary care database will be categorised in five groups, following the categories defined in the UK 2011 Census: White, South Asian, Black, Others and mixed. This variable will be derived from Read codes available in the CPRD GOLD primary care database and from HES, since the combined data sources increase completeness from 55% to 79% [41]. For analysis, four groups will be considered: White, South Asian, Black, Others and mixed.   - Previous mental health history (categorical variable: yes/no)   Mental health history will be defined as having had an episode of anxiety- or depression-related disorders (primary outcomes) or any the secondary outcomes, ever recorded at more than 1 year before the breast cancer diagnosis (patients who had the outcome in the year before the breast cancer diagnosis will be excluded from the cohort). These will be identified based on the algorithms defined in aim 1 of this study.   - History of stroke or coronary heart disease at diagnosis (two dichotomous variables: yes/no)   These will include ischaemic heart disease (angina and myocardial infarction) and stroke, which are amongst the leading causes of disability-adjusted life years in females in the UK. These will be identified through Read codes recorded in the CPRD GOLD primary care database.   - Socioeconomic status (IMD quintiles of deprivation)   The IMD is an ecological measure of deprivation for small areas in England (Lower Super Output Areas). It combines information from seven domain indices (income, employment, education, health deprivation, crime, barriers to housing, and living environment). The index ranks the areas from 1 to 32,844; usually the quintiles are used for research purposes: from 1 (most deprived) up to 5 (least deprived). The IMD is linked to the primary care data using the postcode of the patient or practice. *Potential mediators of the association between breast cancer history and anxiety/depression*  - Sequelae from cancer treatments (six separate binary variables (yes/no): lymphoedema, pain, mild cognitive impairment, fatigue, sexual dysfunction, sleep disorder)   Lymphoedema will be defined using Read codes for the condition, in the CPRD GOLD primary care database. Pain, mild cognitive impairment, fatigue, sexual dysfunction, and sleep disorder are secondary outcomes of this study, and will be identified based on the algorithms defined in aim 1 of this study. Patients will be classified as having had one of these conditions if there was more than one record for these conditions within a 6-month interval.   - Exposed to endocrine treatment for breast cancer: binary variable (yes/no).   This will be defined from the CPRD GOLD primary care database using Read codes for at least two prescriptions of anastrozole, tamoxifen, exemestane, or letrozole [43] within a 6-month period. |
| Data/ Statistical AnalysisPrimary analysesSpecific aim 1. To develop and validate algorithms to identify patients with anxiety and depression Algorithm development  A systematic review is currently under way to identify the lists of Read codes previously used to define anxiety and depression of primary care databases in the UK. The systematic review search expressions are provided in appendix 5.  *(A) Raw data tabulations*  We will estimate the number and proportion of patients recorded during the observation period with:   1. Diagnostic Read code for anxiety/depression; 2. Prescription of anxiolytics/antidepressants; 3. Referred to mental health services; 4. Symptoms of anxiety/depression.   Proportions will be estimated by calendar year.  *(B) Simpler algorithm*  We will estimate the additional contribution of prescriptions, referrals and symptoms to identify cases of anxiety/depression in CPRD. Referrals and prescriptions of anxiolytics/antidepressants will be considered as sufficient to identify cases of anxiety/depression if a Read code for symptoms of anxiety/depression, respectively, were recorded during the previous year. The reasons for this are threefold: (1) there is good evidence that GPs switched from anxiety/depression diagnostic codes to symptomatic ones [44, 45], following claims of over diagnosis of these conditions; (2) antidepressants and anxiolytics have also other indications, including anxiety disorders for antidepressants [46], which raise questions of the use of these data their own to identify these outcomes; (3) pharmacological treatment of mild depression has been discouraged since 2004 [47], and thus referrals to psychotherapy may help to capture milder cases.  *(C) Complex algorithm*  A more detailed algorithm will be developed considering that some drugs are prescribed for both depression and anxiety, in addition to manage vasomotor symptoms, which may be more frequent in breast cancer survivors than in women who did not have cancer. An example for depression is given in appendix 6. We will estimate the number of patients with anxiety/depression at each step, to evaluate how much each category adds to what has been previously recorded.  Algorithm validation  A random sample of 1 million patients of both sexes will be selected from the CPRD GOLD primary care database. We will apply each algorithm and produce descriptive statistics stratified by likelihood of having the outcome, including:   - Number and proportion of patients with the anxiety, depression or both, by 3-year calendar period; - Number and proportion of patients with the anxiety, depression or both, by 3-year calendar period, age and sex; - Number and proportion of patients with the anxiety and depression by 3-year calendar period and country in the UK.   For each outcome, we will compare the estimates obtained with others obtained from the literature, prioritising national surveys of population-based data such as the following:   - Adult Psychiatric Morbidity Survey 2007 [48] and 2014 [49]; - Measuring National Well-being programme, made available by the Office for National Statistics (includes prevalence of those in the UK with some evidence indicating depression or anxiety, since 2013, by country in the UK and English regions, and by 10-year age groups up to 75 years) [50].   The final algorithm will be chosen by considering information in the numeric value closer to the estimates obtained from the literature. Sensitivity analysis Proportion estimates considering the presence of symptomatic codes within the previous year will be re-calculated to consider shorter periods of time (i.e. 3 and 6 months). Specific aim 2. Risk of mental health outcomes in women who had breast cancer compared to women who did not have cancer**Primary outcomes analysis – depression and anxiety**Main analysis Descriptive statistics including number of events observed and person-years at risk will be computed, overall and stratified by the covariates listed in section M (see above). Medical procedures for the diagnosis of breast cancer are likely to cause anxiety. To deal with this we planned to exclude patients who had a record of anxiety diagnosed within 1 year before the breast cancer; this could result in patients with higher levels of trait anxiety being excluded from the analysis. We will calculate the number and proportion of patients who were excluded because they had the outcome in the year prior to the breast cancer, stratified by month. We will also describe how many of those who were excluded from analysis had an outcome after the breast cancer diagnosis by age-group. The quintiles of the distribution of the number of consultations (defined using the ‘consid’ variable) will be described, as this can indicate the patterns of seeking care between women with a history of cancer, compared to those who did not.  The association between breast cancer history and anxiety and breast cancer history and depression, will be quantified using Cox regression models with time since index as the underlying time scale, and stratifying on matched set to account for matching by age and primary care practice. Follow up will begin at the index date (vide section M for definition of the exposure) and will terminate when an outcome is observed. Women will be censored at the earliest date of any of these: cancer recurrence, other cancer diagnosis, death, transference out of the practice; if these events don’t occur, censoring will be observed at the date of last data collection for the practice. Crude measures of the association between breast cancer history and anxiety and depression will be reported stratifying by the covariates described in section M.  Cox multivariate regression analysis will be use to estimate hazard ratios adjusted for calendar period of breast cancer diagnosis, menopausal status, and diabetes mellitus at baseline (see list of confounders in section L, covariates, for the definition of these variables).  Interaction terms between the exposure and the following variables will be added, to explore effect modification by ethnicity (White, South Asian, Black, Others and mixed), place of residence (care home vs. household), co-habitation status (living alone vs. cohabiting), SES (quintiles of IMD), having mental health disorders history (yes vs. no), having history of stroke (yes vs. no) and coronary heart disease (yes vs. no).  Confidence intervals will be calculated using robust estimates of the standard errors, to account for the fact that patients may also contribute with time at risk in the unexposed cohort prior to their cancer diagnosis.  The proportional hazards assumption will be tested in two ways: 1) graphically, by plotting the cumulative rates on a log scale; 2) inferentially, by applying a likelihood ratio test to the estimates obtained for the entire period of observation and for time split into intervals. Sensitivity analyses Sensitivity analyses planned for this study will include a subset of patients only; this is because they use variables from linked data, which is available for a fraction of the patients only, and include variables amenable to have missing data (i.e. BMI, alcohol intake, smoking and patient-level IMD). Regarding the latter, we will quantify the completeness of each variable to decide on their inclusion in the final models. Analyses including variables with missing data will be restricted to patients with complete data for covariates (complete case analysis), if missing data is likely to be missing not a random. The following sensitivity analyses are planned:   1. The main analysis will be repeated further adjusting for age at diagnosis (continuous variable, since matching allowed for 3-year gap), patient quintile level of IMD, alcohol drinking patterns prior to index date, smoking history and body mass index categories prior to index date. 2. We will exclude women diagnosed with the outcome of interest in the year prior to the cancer diagnosis. To account for the fact that treatment for these conditions may often last for more than one year, and that mild anxiety and depression may be treated in psychological services and not result in visits to the GP, we will repeated the main analysis including only patients who had 5 years of complete follow up prior to the index date and did not have the outcome recorded at any point during this period.  Multiple comparisons We acknowledge that this study includes multiple comparisons for each outcome, and several outcomes. Thus, P-values in the range ~0.01-0.05 be considered as some statistical evidence of an effect and interpreted cautiously. Sample size considerations considering a 0.01 probability of type I error (α) are provided in see section I. Specific aim 3. To estimate association between breast cancer history and anxiety/depression by presence of lymphoedema, pain, mild cognitive impairment, fatigue, sexual dysfunction, sleep disorders, and having done endocrine therapy, during the follow up period. We will estimate the cumulative incidence and period prevalence of lymphoedema, pain, mild cognitive impairment, fatigue, sexual dysfunction, and sleep disorders, in breast cancer survivors during the overall follow up period and by 5-year of follow up period.  We will estimate the hazards of developing anxiety and depression (the main outcomes) for three groups of patients: 1) breast cancer survivors who did not develop the common complication (i.e. lymphoedema, pain, mild cognitive impairment, fatigue, sexual dysfunction, sleep disorders) up to time *t*; 2) breast cancer survivors who have had the common complication at time *t*; 3) women who never had cancer. A Cox regression model will be used to estimate the association between time-updated exposure and the main outcomes (anxiety and depression), having as reference the hazard observed for the women who did not have cancer. The exposure variable will be time-updated; this means that women who develop a common complication will contribute with information to group 1 until the date at which they develop the complication of interest; after this point they will contribute with information to group 2. All models will be adjusted for calendar period of breast cancer diagnosis.  Confidence intervals will be calculated using robust estimates of the standard errors. Sensitivity analyses planned in objective 2 will be applied to this objective as well. Secondary outcomes analyses – fatigue, mild cognitive impairment, pain, sleep disturbance, sexual dysfunction, fatal and non-fatal self-harm The steps outlined above will be repeated for the secondary outcomes. Sensitivity analysis will follow the same rationale as described for the primary analysis. |
| Plan for addressing confounding The confounding effect of age and socio-economic status (SES) will be limited at the study design phase, as  women who had breast cancer will be individually matched to women who never had cancer by age and primary  care practice. Variables considered as important confounders will be included in the multivariate Cox regression  models (vide section M, covariates). |
| Plans for addressing missing data There are no plans for using multiple imputation methods in this study. Three variables in the main analysis are likely to have missing data: body mass index, smoking and alcohol intake. The probability of these values being recorded in the patients’ medical records is likely to depend on the actual value (e.g. obese patients may have their weight more often assessed and smoking may be more often recorded in patients who visit the GP for complications of smoking (e.g. chronic obstructive pulmonary disease). This is a direct violation of the missing at random assumption needed for multiple imputation. We will therefore conduct a complete case analysis, which is a valid method when missingness is conditionally independent of the outcome [52]. |
| Patient or user group involvement (if applicable) No patients were involved in the drafting of this protocol. |
| Plans for disseminating and communicating study results, including the presence or absence of any restrictions on the extent and timing of publication The results of this study will be presented at scientific conferences in the area, and submitted for publication in peer-reviewed journals.  **Conflict of interest statement:** Ms Williams reports that CPRD has financial relationships with its clients, including the London School of Hygiene and Tropical Medicine, in relation to providing access to research data and services outside the submitted work. Dr. Stanway reports personal fees from Roche, Clinigen, Eli Lilly, and Novartis, not related to this work. Dr. Bhaskaran reports grants from Wellcome Trust, the Royal Society, Medical Research Council, and British Heart Foundation, outside the submitted work. |
| Limitations of the study design, data sources, and analytic methods Validity of the mental health diagnosis in the CPRD GOLD primary care database  The validity and the completeness of the recording of the mental disorders in CPRD have not been evaluated, and this will limit our results. The diagnosis and treatment of depression has also changed over time, as a result of the 2004 NICE guidelines (discouraging the treatment of mild depression with antidepressants) and the Quality and Outcomes Framework scheme in 2006, which recommended validated questionnaires to evaluate its severity [8, 9]. As for completeness, mental disorders such as depression and anxiety are managed at the primary care level, and therefore the potential for recording is high. Nevertheless, some of these conditions, especially in the sub-threshold or milder severities, may not result in GP visits and go therefore undiagnosed [10].  Unmeasured and residual confounding  This study is also limited by the lack of historical data on potential confounders, such as physical activity. Residual confounding will not be possible to rule out for variables such as smoking [53].  Multiple indications of the psychotropic medicines (complex algorithm definition)  In clinical practice, several classes of pharmacological agents are currently used to manage anxiety and depressive disorders, and many of these pharmacological agents are used to treat other physical and mental disorders (appendix 7). An example of this is illustrated by the guidelines. The National Institute for Health and Care Excellence (NICE) issued guidelines for recognition and management of depression in people who have a physical chronic condition such as cancer [54], and for management of generalized anxiety disorder (GAD) and panic disorder [46]. Low- or high-intensity psychological interventions (e.g. low intensity: individual non-facilitated or guided self-help; high intensity: cognitive behavioural therapy) are recommended as the first line for GAD, mild depression and long-term insomnia (>4 weeks) [46]. Pharmacological treatment is recommended for persisting GAD, moderate to severe depression and insomnia that causes severe daytime dysfunction. SSRI are currently recommended for GAD; benzodiazepines are most often restricted to crisis and not recommended for long-term use [46]. This will raise issues on the indication under which the patient has been prescribed the medicine. We will select all pharmacological agents used to treat each of the outcomes, and seek experts’ advice on which drugs are often prescribed. In any case, this offers a potential for misclassification (thought to be non-differential). |
| References 1. Maddams J, M Utley, and H Moller. Projections of cancer prevalence in the United Kingdom, 2010-2040. Br J Cancer 2012:107:1195-202.  2. Capelan M, NML Battisti, A McLoughlin, V Maidens, et al. The prevalence of unmet needs in 625 women living beyond a diagnosis of early breast cancer. Br J Cancer 2017:117:1113-1120.  3. Hopwood P, G Sumo, J Mills, J Haviland, et al. The course of anxiety and depression over 5 years of follow-up and risk factors in women with early breast cancer: results from the UK Standardisation of Radiotherapy Trials (START). Breast 2010:19:84-91.  4. Lindberg P, M Koller, B Steinger, W Lorenz, et al. Breast cancer survivors' recollection of their illness and therapy seven years after enrolment into a randomised controlled clinical trial. BMC Cancer 2015:15:554.  5. Glaser AW, LK Fraser, J Corner, R Feltbower, et al. Patient-reported outcomes of cancer survivors in England 1-5 years after diagnosis: a cross-sectional survey. BMJ Open 2013:3.  6. Lewis RA, RD Neal, NH Williams, B France, et al. Follow-up of cancer in primary care versus secondary care: systematic review. Br J Gen Pract 2009:59:e234-47.  7. Khan NF, AM Ward, E Watson, and PW Rose. Consulting and prescribing behaviour for anxiety and depression in long-term survivors of cancer in the UK. Eur J Cancer 2010:46:3339-44.  8. Hjerl K, EW Andersen, N Keiding, PB Mortensen, et al. Increased incidence of affective disorders, anxiety disorders, and non-natural mortality in women after breast cancer diagnosis: a nation-wide cohort study in Denmark. Acta Psychiatr Scand 2002:105:258-64.  9. Suppli NP, C Johansen, J Christensen, LV Kessing, et al. Increased risk for depression after breast cancer: a nationwide population-based cohort study of associated factors in Denmark, 1998-2011. J Clin Oncol 2014:32:3831-9.  10. Yang H, JS Brand, F Fang, F Chiesa, et al. Time-dependent risk of depression, anxiety, and stress-related disorders in patients with invasive and in situ breast cancer. Int J Cancer 2017:140:841-852.  11. Hung YP, CJ Liu, CF Tsai, MH Hung, et al. Incidence and risk of mood disorders in patients with breast cancers in Taiwan: a nationwide population-based study. Psychooncology 2013:22:2227-34.  12. Kim MS, SY Kim, JH Kim, B Park, et al. Depression in breast cancer patients who have undergone mastectomy: A national cohort study. PLoS One 2017:12:e0175395.  13. Herrett E, AM Gallagher, K Bhaskaran, H Forbes, et al. Data Resource Profile: Clinical Practice Research Datalink (CPRD). Int J Epidemiol 2015:44:827-36.  14. Ranopa M, I Douglas, T van Staa, L Smeeth, et al. The identification of incident cancers in UK primary care databases: a systematic review. Pharmacoepidemiol Drug Saf 2015:24:11-8.  15. StataCorp, *STATA statistical software*, 2015, Stata Corporation: College Station TX.  16. Earle CC, BA Neville, and R Fletcher. Mental health service utilization among long-term cancer survivors. J Cancer Surviv 2007:1:156-60.  17. Rubino C, A Figus, L Lorettu, and G Sechi. Post-mastectomy reconstruction: a comparative analysis on psychosocial and psychopathological outcomes. J Plast Reconstr Aesthet Surg 2007:60:509-18.  18. Schairer C, LM Brown, BE Chen, R Howard, et al. Suicide after breast cancer: an international population-based study of 723,810 women. J Natl Cancer Inst 2006:98:1416-9.  19. Fang F, K Fall, MA Mittleman, P Sparen, et al. Suicide and cardiovascular death after a cancer diagnosis. N Engl J Med 2012:366:1310-8.  20. Geulayov G, N Kapur, P Turnbull, C Clements, et al. Epidemiology and trends in non-fatal self-harm in three centres in England, 2000-2012: findings from the Multicentre Study of Self-harm in England. BMJ Open 2016:6:e010538.  21. Webb RT, E Kontopantelis, T Doran, P Qin, et al. Risk of self-harm in physically ill patients in UK primary care. J Psychosom Res 2012:73:92-7.  22. El Rafihi-Ferreira R, Nogueira Pires ML, and Zoega Soares MR. Sleep, quality of life and depression in women in breast cancer post-treatment. Psicologia: Reflexao e Critica 2011:25:506-513.  23. Dahl AA, IL Nesvold, KV Reinertsen, and SD Fossa. Arm/shoulder problems and insomnia symptoms in breast cancer survivors: cross-sectional, controlled and longitudinal observations. Sleep Med 2011:12:584-90.  24. Hermelink K, M Buhner, P Sckopke, F Neufeld, et al. Chemotherapy and Post-traumatic Stress in the Causation of Cognitive Dysfunction in Breast Cancer Patients. J Natl Cancer Inst 2017:109.  25. Jenkins V, V Shilling, G Deutsch, D Bloomfield, et al. A 3-year prospective study of the effects of adjuvant treatments on cognition in women with early stage breast cancer. Br J Cancer 2006:94:828-34.  26. Fan HG, N Houede-Tchen, QL Yi, I Chemerynsky, et al. Fatigue, menopausal symptoms, and cognitive function in women after adjuvant chemotherapy for breast cancer: 1- and 2-year follow-up of a prospective controlled study. J Clin Oncol 2005:23:8025-32.  27. Collins B, J Mackenzie, GA Tasca, C Scherling, et al. Persistent cognitive changes in breast cancer patients 1 year following completion of chemotherapy. J Int Neuropsychol Soc 2014:20:370-9.  28. Thomas KH, N Davies, C Metcalfe, F Windmeijer, et al. Validation of suicide and self-harm records in the Clinical Practice Research Datalink. Br J Clin Pharmacol 2013:76:145-57.  29. Stuart EA. Matching methods for causal inference: A review and a look forward. Stat Sci 2010:25:1-21.  30. World Cancer Research Fund/American Institute for Cancer, *Continuous Update Project Report. Food, Nutrition, Physical Activity, and the Prevention of Breast Cancer*, 2010.  31. Liang W and T Chikritzhs. Affective disorders, anxiety disorders and the risk of alcohol dependence and misuse. Br J Psychiatry 2011:199:219-24.  32. Bulloch A, D Lavorato, J Williams, and S Patten. Alcohol consumption and major depression in the general population: the critical importance of dependence. Depress Anxiety 2012:29:1058-64.  33. Berglund M and A Ojehagen. The influence of alcohol drinking and alcohol use disorders on psychiatric disorders and suicidal behavior. Alcohol Clin Exp Res 1998:22:333S-345S.  34. Bhaskaran K, I Douglas, H Forbes, I dos-Santos-Silva, et al. Body-mass index and risk of 22 specific cancers: a population-based cohort study of 5.24 million UK adults. Lancet 2014:384:755-65.  35. Kivimaki M, DA Lawlor, A Singh-Manoux, GD Batty, et al. Common mental disorder and obesity: insight from four repeat measures over 19 years: prospective Whitehall II cohort study. BMJ 2009:339:b3765.  36. Simon GE, M Von Korff, K Saunders, DL Miglioretti, et al. Association between obesity and psychiatric disorders in the US adult population. Arch Gen Psychiatry 2006:63:824-30.  37. Tsilidis KK, JC Kasimis, DS Lopez, EE Ntzani, et al. Type 2 diabetes and cancer: umbrella review of meta-analyses of observational studies. BMJ 2015:350:g7607.  38. Anderson RJ, KE Freedland, RE Clouse, and PJ Lustman. The prevalence of comorbid depression in adults with diabetes: a meta-analysis. Diabetes Care 2001:24:1069-78.  39. Eastwood SV, R Mathur, M Atkinson, S Brophy, et al. Algorithms for the Capture and Adjudication of Prevalent and Incident Diabetes in UK Biobank. PLoS One 2016:11:e0162388.  40. Tate AR, S Dungey, S Glew, N Beloff, et al. Quality of recording of diabetes in the UK: how does the GP's method of coding clinical data affect incidence estimates? Cross-sectional study using the CPRD database. BMJ Open 2017:7:e012905.  41. Jain A, AJ van Hoek, JL Walker, R Mathur, et al. Identifying social factors amongst older individuals in linked electronic health records: An assessment in a population based study. PLoS One 2017:12:e0189038.  42. Sandilyan MB and T Dening. Mental health around and after the menopause. Menopause Int 2011:17:142-7.  43. National Institute for Health and Care Excellence (NICE), *Early and locally advanced breast cancer: diagnosis and treatment. Clinical Guideline [CG80] (updated 2017)*, 2009.  44. Kendrick T, B Stuart, C Newell, AW Geraghty, et al. Changes in rates of recorded depression in English primary care 2003-2013: Time trend analyses of effects of the economic recession, and the GP contract quality outcomes framework (QOF). J Affect Disord 2015:180:68-78.  45. Walters K, G Rait, M Griffin, M Buszewicz, et al. Recent trends in the incidence of anxiety diagnoses and symptoms in primary care. PLoS One 2012:7:e41670.  46. National Institute for Health and Care Excellence (NICE), *Generalised anxiety disorder and panic disorder in adults: management (CG113)*, 2011.  47. National Institute for Health and Care Excellence (NICE), *Depression - Management of depression in primary and secondary care. Clinical Guideline 23*, 2004.  48. NHS Digital. *Adult Psychiatric Morbidity in England - 2007, Results of a household survey*. 2009; Available from: <https://digital.nhs.uk/data-and-information/publications/statistical/adult-psychiatric-morbidity-survey/adult-psychiatric-morbidity-in-england-2007-results-of-a-household-survey#section-related-links>.  49. McManus S, P Bebbington, R Jenkins, T Brugha, et al., *Mental health and wellbeing in England: Adult Psychiatric Morbidity Survey 2014*, 2016: Leeds: NHS Digital.  50. Office for National Statistics, *Measuring National Well-being: Domains and Measures, and Personal well-being estimates (2012-2017)*, 2018.  51. Bhaskaran K, HJ Forbes, I Douglas, DA Leon, et al. Representativeness and optimal use of body mass index (BMI) in the UK Clinical Practice Research Datalink (CPRD). BMJ Open 2013:3:e003389.  52. White IR and JB Carlin. Bias and efficiency of multiple imputation compared with complete-case analysis for missing covariate values. Stat Med 2010:29:2920-31.  53. Lewis JD and C Brensinger. Agreement between GPRD smoking data: a survey of general practitioners and a population-based survey. Pharmacoepidemiol Drug Saf 2004:13:437-41.  54. National Institute for Health and Care Excellence (NICE), *Depression in adults with a chronic physical health problem: recognition and management (CG91)*, 2009.  55. British Medical Association and Royal Pharmaceutical Society of Great Britain, *British national formulary*, 2018: London, United Kingdom. |
| List of Appendices Appendix 1. Helena Carreira CV  Appendix 2. List of codes to identify BC cases in the CPRD GOLD primary care database.  Appendix 3. ICD-9 and ICD-10 codes for self-harm, attempted suicide and completed suicide.  Appendix 4. Direct acyclic graph.  Appendix 5. Systematic review search expressions.  Appendix 6. Algorithm to identify cases of depression in the CPRD GOLD primary care database.  Appendix 7. Drugs used to manage anxiety-related disorders, according to the British National Formulary [55]. |
