# Supplementary material for: Associations between breast cancer survivorship and adverse mental health outcomes: A matched population-based cohort study in the United Kingdom
Source: PLoS Med. 2021 Jan 7;18(1):e1003504. doi: 10.1371/journal.pmed.1003504 (PMC7822529; doi:10.1371/journal.pmed.1003504)
Supplement: S1 Table — Table A: characteristics of the patients excluded and number and mean follow-up time of patients available for analyses: anxiety, depression, and cognitive dysfunction. Table B: characteristics of the patients excluded and number and mean follow-up time of patients available for analyses: fatigue, sexual dysfunction, and sleep disorder. Table C: characteristics of the patients excluded and number and mean follow-up time of patients available for analyses: pain, opioids analgesics, and fatal and nonfatal self-harm. (DOCX) [file pmed.1003504.s005.docx]

# **S1 Table A** Characteristics of the patients excluded, and number and mean follow up time of patients available for analyses: anxiety, depression and cognitive dysfunction.

|  | | **Anxiety** | | | | |  | **Depression** | | | | |  | **Cognitive dysfunction** | | | | |
| --- | --- | --- | --- | --- | --- | --- | --- | --- | --- | --- | --- | --- | --- | --- | --- | --- | --- | --- |
|  | | **Unexposed** | |  | **Exposed** | |  | **Unexposed** | |  | **Exposed** | |  | **Unexposed** | |  | **Exposed** | |
| **Patients eligible for analysis, N (%)** | | 230,067 | (100.00) |  | 57,571 | (100.00) |  | 230,067 | (100.00) |  | 57,571 | (100.00) |  | 230,067 | (100.00) |  | 57,571 | (100.00) |
|  | |  |  |  |  |  |  |  |  |  |  |  |  |  |  |  |  |  |
| **Exclusions from outcome-specific analysis, N (%)** | | 5,929 | (2.58) |  | 1,955 | (3.40) |  | 13,712 | (5.96) |  | 3,498 | (6.08) |  | 5,623 | (2.44) |  | 1,519 | (2.64) |
|  | Patients excluded who had the outcome recorded *after* the index date, N (%) | 2,707 | (45.7) |  | 896 | (45.8) |  | 8,511 | (62.1) |  | 2,175 | (62.2) |  | - | - |  | - | - |
|  | Mean time (SD) between the mental health outcome diagnosis and the index date, days | 214 | (109) |  | 218 | (110) |  | 212 | (109) |  | 214 | (110) |  | - | - |  | - | - |
|  | Age at index date (years) |  |  |  |  |  |  |  |  |  |  |  |  |  |  |  |  |  |
|  | Mean (SD) | 60 | (14) |  | 60 | (13) |  | 61 | (14) |  | 61 | (14) |  | 74 | (13.12) |  | 74 | (13.30) |
|  | Minimum-maximum | 21-101 | |  | 24-102 | |  | 19-101 | |  | 22-101 | |  | 28-103 | |  | 29-101 | |
|  |  |  | |  |  | |  |  | |  |  | |  |  | |  |  | |
| **Total number of patients included in analysis (%)** | | **224,138** | **(97.42)** |  | **55,616** | **(96.60)** |  | **216,355** | **(94.04)** |  | **54,073** | **(93.92)** |  | **224,444** | **(97.56)** |  | **56,052** | **(97.36)** |
| **Total number of person-years at risk** | | **1,306,784** | |  | **288,115** | |  | **1,202,647** | |  | **261,081** | |  | **1,385,179** | |  | **315,453** | |
| **Duration of follow up (years)** | |  |  |  |  |  |  |  |  |  |  |  |  |  |  |  |  |  |
|  | Mean (SD) | 5.83 | (4.79) |  | 5.18 | (4.57) |  | 5.56 | (4.74) |  | 4.83 | (4.48) |  | 6.17 | (4.94) |  | 5.63 | (4.71) |
|  | Median | 4.61 |  |  | 3.89 |  |  | 4.26 |  |  | 3.44 |  |  | 5.00 |  |  | 4.38 |  |
| **Outcomes during follow-up, N** | | 20,224 | |  | 5,888 | |  | 34,558 | |  | 10,175 | |  | 19,845 | |  | 4,368 | |

SD – standard deviation.

# **S1 Table B** Characteristics of the patients excluded, and number and mean follow up time of patients available for analyses: fatigue, sexual dysfunction and sleep disorder.

|  | | **Fatigue** | | | | |  | **Sexual dysfunction** | | | | |  | **Sleep disorder** | | | | |
| --- | --- | --- | --- | --- | --- | --- | --- | --- | --- | --- | --- | --- | --- | --- | --- | --- | --- | --- |
|  | | **Unexposed** | |  | **Exposed** | |  | **Unexposed** | |  | **Exposed** | |  | **Unexposed** | |  | **Exposed** | |
| **Patients eligible for analysis, N (%)** | | 230,067 | (100.00) |  | 57,571 | (100.00) |  | 230,067 | (100.00) |  | 57,571 | (100.00) |  | 230,067 | (100.00) |  | 57,571 | (100.00) |
|  | |  |  |  |  |  |  |  |  |  |  |  |  |  |  |  |  |  |
| **Exclusions from outcome-specific analysis, N (%)** | | 6,561 | (2.85) |  | 1,660 | (2.88) |  | 490 | (0.21) |  | 127 | (0.22) |  | 4,484 | (1.95) |  | 1,361 | (2.36) |
|  | Patients excluded who had the outcome recorded *after* the index date, N (%) | 2,409 | (36.7) |  | 609 | (36.7) |  | 87 | (17.8) |  | 12 | (9.5) |  | 1,822 | (40.6) |  | 622 | (45.7) |
|  | Mean time (SD) between the mental health outcome diagnosis and the index date, days | 208 | (110) |  | 211 | (107) |  | 203 | (111) |  | 202 | (106) |  | 206 | (106) |  | 215 | (106) |
|  | Age at index date (years) |  |  |  |  |  |  |  |  |  |  |  |  |  |  |  |  |  |
|  | Mean (SD) | 62 | (14) |  | 63 | (14) |  | 53 | (9) |  | 51 | (10) |  | 67 | (15) |  | 65 | (15) |
|  | Minimum-maximum | 19-101 | |  | 26-103 | |  | 21-92 | |  | 21-77 | |  | 21-99 | |  | 19-101 | |
|  |  |  | |  |  | |  |  | |  |  | |  |  | |  |  | |
| **Total number of patients included in analysis (%)** | | **223,506** | **(97.10)** |  | **55,911** | **(97.10)** |  | **229,577** | **(99.8)** |  | **57,444** | **(99.8)** |  | **225,583** | **(98.10)** |  | **56,210** | **(97.60)** |
| **Total number of person-years at risk** | | **1,266,975** | |  | **280,982** | |  | **1,435,837** | |  | **325,393** | |  | **1,338,065** | |  | **290,786** | |
| **Duration of follow up (years)** | |  |  |  |  |  |  |  |  |  |  |  |  |  |  |  |  |  |
|  | Mean (SD) | 5.67 | (4.73) |  | 5.03 | (4.48) |  | 6.25 | (4.98) |  | 5.66 | (4.75) |  | 5.93 | (4.85) |  | 5.17 | (4.60) |
|  | Median | 4.44 |  |  | 3.69 |  |  | 5.09 |  |  | 4.40 |  |  | 4.71 |  |  | 3.86 |  |
| **Outcomes during follow-up, N** | | 28,886 | |  | 8,359 | |  | 2,153 | |  | 683 | |  | 16,798 | |  | 6,002 | |

SD – standard deviation.

# **S1 Table C** Characteristics of the patients excluded, and number and mean follow up time of patients available for analyses: pain, opioids analgesics, fatal and non-fatal self-harm.

|  | | **Pain** | | | | |  | **Opioids analgesics** | | | | |  | **Fatal and non-fatal self-harm** | | | | |
| --- | --- | --- | --- | --- | --- | --- | --- | --- | --- | --- | --- | --- | --- | --- | --- | --- | --- | --- |
|  | | **Unexposed** | |  | **Exposed** | |  | **Unexposed** | |  | **Exposed** | |  | **Unexposed** | |  | **Exposed** | |
| **Patients eligible for analysis, N (%)** | | 230,067 | (100.00) |  | 57,571 | (100.00) |  | 230,067 | (100.00) |  | 57,571 | (100.00) |  | 132,647 | (100.00) |  | 33,168 | (100.00) |
|  | |  |  |  |  |  |  |  |  |  |  |  |  |  |  |  |  |  |
| **Exclusions from outcome-specific analysis, N (%)** | | 68,030 | (29.57) |  | 18,800 | (32.66) |  | 16,877 | (7.34) |  | 4,899 | (8.51) |  | 315 | (0.24) |  | 63 | (0.19) |
|  | Patients excluded who had the outcome recorded *after* the index date, N (%) | 54,493 | (80.10) |  | 14867 | (79.08) |  | 12,547 | (74.3) |  | 3,855 | (78.69) |  | 80 | (25.4) |  | 14 | (22.2) |
|  | Mean time (SD) between the mental health outcome diagnosis and the index date, days | 215 | (107.80) |  | 214 | (109.78) |  | 217 | (108) |  | 215 | (111) |  | 229 | (110) |  | 218 | (110) |
|  | Age at index date (years) |  |  |  |  |  |  |  |  |  |  |  |  |  |  |  |  |  |
|  | Mean (SD) | 63 | (14) |  | 63 | (14) |  | 66 | (14) |  | 67 | (13) |  | 54 | (13) |  | 52 | (17) |
|  | Minimum-maximum | 19-102 | |  | 19-103 | |  | 27-91 | |  | 25-91 | |  | 30-102 | |  | 25-91 | |
|  |  |  | |  |  | |  |  | |  |  | |  |  | |  |  | |
| **Total number of patients included in analysis (%)** | | **162,037** | **(70.43)** |  | **38,771** | **(67.34)** |  | **213,190** | **(92.7)** |  | **52,672** | **(91.5)** |  | **132,332** | **(99.8)** |  | **33,105** | **(99.8)** |
| **Total number of person-years at risk** | | **505,451** | |  | **100312** | |  | **1,181,155** | |  | **248,654** | |  | **831,516** | |  | **190,182** | |
| **Duration of follow up (years)** | |  |  |  |  |  |  |  |  |  |  |  |  |  |  |  |  |  |
|  | Mean (SD) | 3.12 | (3.30) |  | 2.59 | (2.88) |  | 5.54 | (4.70) |  | 4.72 | (4.49) |  | 6.28 | (4.98) |  | 5.74 | (4.76) |
|  | Median | 1.99 |  |  | 1.59 |  |  | 4.27 |  |  | 3.36 |  |  | 5.13 |  |  | 4.50 |  |
| **Outcomes during follow-up, N** | | 94,171 | |  | 24,522 | |  | 44,850 | |  | 17,315 | |  | 794 | |  | 182 | |

SD – standard deviation.
