# Supplementary material for: Associations between breast cancer survivorship and adverse mental health outcomes: A matched population-based cohort study in the United Kingdom
Source: PLoS Med. 2021 Jan 7;18(1):e1003504. doi: 10.1371/journal.pmed.1003504 (PMC7822529; doi:10.1371/journal.pmed.1003504)
Supplement: S2 Table — (DOCX) [file pmed.1003504.s006.docx]

# **S2 Table A** Adjusted associations between breast cancer survivors and anxiety, depression, cognitive dysfunction and fatigue.

|  | **Anxiety** | | |  | **Depression** | | |  | **Cognitive dysfunction** | | |  | **Fatigue** | | |
| --- | --- | --- | --- | --- | --- | --- | --- | --- | --- | --- | --- | --- | --- | --- | --- |
|  | **HR** | **95%CI** | **P value** |  | **HR** | **95%CI** | **P value** |  | **HR** | **95%CI** | **P value** |  | **HR** | **95%CI** | **P value** |
| **Breast cancer** |  |  |  |  |  |  |  |  |  |  |  |  |  |  |  |
| No | Ref |  |  |  | Ref |  |  |  | Ref |  |  |  | Ref |  |  |
| Yes | 1.33 | 1.29-1.36 | <0.001 |  | 1.35 | 1.32-1.38 | <0.001 |  | 1.00 | 0.97-1.04 | 0.88 |  | 1.28 | 1.25-1.31 | <0.001 |
| **Diabetes** |  |  |  |  |  |  |  |  |  |  |  |  |  |  |  |
| No | Ref |  |  |  | Ref |  |  |  | Ref |  |  |  | Ref |  |  |
| Yes | 0.93 | 0.87-0.99 | 0.02 |  | 1.75 | 1.68-1.83 | <0.001 |  | 1.80 | 1.71-1.88 | <0.001 |  | 1.00 | 0.96-1.05 | 0.90 |
| **Body mass index (kg/m^2^)*** |  |  |  |  |  |  |  |  |  |  |  |  |  |  |  |
| <18.50 | 1.08 | 0.98-1.19 | 0.12 |  | 1.09 | 1.01-1.17 | 0.03 |  | 1.24 | 1.12-1.37 | <0.001 |  | 0.97 | 0.89-1.06 | 0.50 |
| 18.50-24.99 | Ref |  |  |  | Ref |  |  |  | Ref |  |  |  | Ref |  |  |
| 25.00-29.99 | 1.00 | 0.97-1.03 | 0.89 |  | 1.11 | 1.08-1.13 | <0.001 |  | 0.95 | 0.92-0.98 | 0.003 |  | 1.05 | 1.03-1.08 | <0.001 |
| 30.00-34.99 | 0.98 | 0.94-1.01 | 0.23 |  | 1.26 | 1.22-1.30 | <0.001 |  | 1.04 | 1.00-1.09 | 0.07 |  | 1.09 | 1.05-1.12 | <0.001 |
| 35.00-39.99 | 0.93 | 0.87-0.98 | 0.01 |  | 1.37 | 1.31-1.43 | <0.001 |  | 1.07 | 1.00-1.14 | 0.41 |  | 1.09 | 1.04-1.15 | <0.001 |
| ≥40.00 | 1.04 | 0.95-1.12 | 0.41 |  | 1.68 | 1.58-1.77 | <0.001 |  | 1.21 | 1.11-1.33 | <0.001 |  | 1.14 | 1.06-1.22 | <0.001 |
| **Smoking status** |  |  |  |  |  |  |  |  |  |  |  |  |  |  |  |
| Non-smoker | Ref |  |  |  | Ref |  |  |  | Ref |  |  |  | Ref |  |  |
| Current smoker | 1.40 | 1.35-1.44 | <0.001 |  | 1.70 | 1.65-1.74 | <0.001 |  | 1.20 | 1.15-1.26 | <0.001 |  | 1.06 | 1.03-1.09 | <0.001 |
| Former smoker | 1.18 | 1.15-1.22 | <0.001 |  | 1.32 | 1.28-1.35 | <0.001 |  | 1.13 | 1.09-1.17 | <0.001 |  | 1.08 | 1.05-1.11 | <0.001 |
| **Alcohol intake** |  |  |  |  |  |  |  |  |  |  |  |  |  |  |  |
| Never drinker | 1.10 | 1.06-1.14 | <0.001 |  | 1.11 | 1.07-1.14 | <0.001 |  | 1.07 | 1.03-1.11 | <0.001 |  | 1.10 | 1.06-1.13 | <0.001 |
| Current drinker | Ref |  |  |  | Ref |  |  |  | Ref |  |  |  | Ref |  |  |
| Former drinker | 1.28 | 1.21-1.34 | <0.001 |  | 1.29 | 1.24-1.34 | <0.001 |  | 1.16 | 1.11-1.22 | <0.001 |  | 1.18 | 1.13-1.23 | <0.001 |

* In the main analysis, we modelled continuous BMI data using restricted cubic splines. To present the results in this table, we re-ran the models including a categorical variable for BMI, as the splines coefficients do not have a direct interpretation. The results between the two models were identical. HR= Hazards ratio; Ref = reference category.

# **S2 Table B** Adjusted associations between breast cancer survivors and sexual dysfunction, sleep disorders, pain, opioid prescription, and fatal and non-fatal self-harm.

|  |  | **Sexual dysfunction** | | |  | **Sleep disorders** | | |  | **Pain** | | |  | **Opioid prescription** | | |  | **Fatal and non-fatal self-harm** | | |
| --- | --- | --- | --- | --- | --- | --- | --- | --- | --- | --- | --- | --- | --- | --- | --- | --- | --- | --- | --- | --- |
|  |  | **HR** | **95%CI** | **P value** |  | **HR** | **95%CI** | **P value** |  | **HR** | **95%CI** | **P value** |  | **HR** | **95%CI** | **P value** |  | **HR** | **95%CI** | **P value** |
| **Breast cancer** |  |  |  |  |  |  |  |  |  |  |  |  |  |  |  |  |  |  |  |  |
| No |  | Ref |  |  |  | Ref |  |  |  | Ref |  |  |  | Ref |  |  |  | Ref |  |  |
| Yes |  | 1.27 | 1.17-1.38 | <0.001 |  | 1.68 | 1.63-1.73 | <0.001 |  | 1.22 | 1.20-1.24 | <0.001 |  | 1.86 | 1.83-1.90 | <0.001 |  | 1.15 | 0.94-1.36 | 0.11 |
| **Diabetes** |  |  |  |  |  |  |  |  |  |  |  |  |  |  |  |  |  |  |  |  |
| No |  | Ref |  |  |  | Ref |  |  |  | Ref |  |  |  | Ref |  |  |  | Ref |  |  |
| Yes |  | 1.28 | 1.00-1.64 | 0.05 |  | 1.02 | 0.96-1.09 | 0.53 |  | 1.06 | 1.03-1.10 | <0.001 |  | 1.22 | 1.18-1.27 | <0.001 |  | 1.40 | 0.96-2.04 | 0.08 |
| **Body mass index (kg/m^2^)** |  |  |  |  |  |  |  |  |  |  |  |  |  |  |  |  |  |  |  |  |
| <18.50 |  | 1.38 | 1.06-1.81 | 0.02 |  | 1.09 | 0.98-1.21 | 0.10 |  | 0.92 | 0.87-0.97 | 0.002 |  | 1.10 | 1.02-1.17 | 0.008 |  | 1.47 | 0.92-2.33 | 0.11 |
| 18.50-24.99 |  | Ref |  |  |  | Ref |  |  |  | Ref |  |  |  | Ref |  |  |  | Ref |  |  |
| 25.00-29.99 |  | 0.77 | 0.71-0.85 | <0.001 |  | 1.01 | 0.97-1.04 | 0.73 |  | 1.13 | 1.11-1.15 | <0.001 |  | 1.23 | 1.20-1.26 | <0.001 |  | 0.96 | 0.80-1.16 | 0.70 |
| 30.00-34.99 |  | 0.62 | 0.54-0.71 | <0.001 |  | 0.98 | 0.93-1.02 | 0.26 |  | 1.23 | 1.21-1.26 | <0.001 |  | 1.54 | 1.49-1.58 | <0.001 |  | 0.90 | 0.80-1.16 | 0.41 |
| 35.00-39.99 |  | 0.61 | 0.50-0.75 | <0.001 |  | 1.03 | 0.96-1.10 | 0.41 |  | 1.28 | 1.24-1.32 | <0.001 |  | 1.78 | 1.71-1.85 | <0.001 |  | 1.19 | 0.84-1.69 | 0.33 |
| ≥40.00 |  | 0.46 | 0.33-0.63 | <0.001 |  | 1.11 | 1.01-1.21 | 0.03 |  | 1.35 | 1.29-1.41 | <0.001 |  | 2.08 | 1.98-2.20 | <0.001 |  | 1.09 | 0.69-1.73 | 0.72 |
| **Smoking status** |  |  |  |  |  |  |  |  |  |  |  |  |  |  |  |  |  |  |  |  |
| Non-smoker |  | Ref |  |  |  | Ref |  |  |  | Ref |  |  |  | Ref |  |  |  | Ref |  |  |
| Current smoker |  | 0.73 | 0.66-0.82 | <0.001 |  | 1.39 | 1.34-1.45 | <0.001 |  | 1.07 | 1.05-1.09 | <0.001 |  | 1.54 | 1.50-1.58 | <0.001 |  | 2.75 | 2.26-3.36 | <0.001 |
| Former smoker |  | 1.26 | 1.14-1.38 | <0.001 |  | 1.24 | 1.20-1.29 | <0.001 |  | 1.15 | 1.13-1.17 | <0.001 |  | 1.29 | 1.26-1.32 | <0.001 |  | 1.71 | 1.39-2.10 | <0.001 |
| **Alcohol intake** |  |  |  |  |  |  |  |  |  |  |  |  |  |  |  |  |  |  |  |  |
| Never drinker |  | 0.75 | 0.66-0.85 | <0.001 |  | 1.00 | 0.96-1.04 | 0.938 |  | 1.01 | 0.99-1.03 | 0.275 |  | 1.12 | 1.09-1.15 | <0.001 |  | 0.99 | 0.79-1.24 | 0.93 |
| Current drinker |  | Ref |  |  |  | Ref |  |  |  | Ref |  |  |  | Ref |  |  |  | Ref |  |  |
| Former drinker |  | 1.26 | 1.06-1.49 | 0.008 |  | 1.10 | 1.04-1.17 | 0.001 |  | 1.09 | 1.06-1.13 | <0.001 |  | 1.24 | 1.20-1.28 | <0.001 |  | 1.56 | 1.17-2.07 | 0.002 |

* In the main analysis, we modelled continuous BMI data using restricted cubic splines. To present the results in this table, we re-ran the models including a categorical variable for BMI, as the splines coefficients do not have a direct interpretation. The results between the two models were identical. HR= Hazards ratio; Ref = reference category.
