# Supplementary material for: Associations between breast cancer survivorship and adverse mental health outcomes: A matched population-based cohort study in the United Kingdom
Source: PLoS Med. 2021 Jan 7;18(1):e1003504. doi: 10.1371/journal.pmed.1003504 (PMC7822529; doi:10.1371/journal.pmed.1003504)
Supplement: S3 Table — Table A: adjusted associations between breast cancer survivors and anxiety, depression, cognitive dysfunction, and fatigue. Table B: adjusted associations between breast cancer survivors and sexual dysfunction, sleep disorders, pain, opioid prescription, and fatal and nonfatal self-harm. (DOCX) [file pmed.1003504.s007.docx]

# **S3 Table** Cumulative incidence of adverse mental health outcomes, fatigue and pain, in breast cancer survivors and in women who did not have cancer.

|  | **Women with no history of cancer** | | | | | | | | | | | | | |  | **Women with history of breast cancer** | | | | | | | | | | | | | |
| --- | --- | --- | --- | --- | --- | --- | --- | --- | --- | --- | --- | --- | --- | --- | --- | --- | --- | --- | --- | --- | --- | --- | --- | --- | --- | --- | --- | --- | --- |
|  | **1 year** | | | |  | **5 years** | | | |  | **10 years** | | | |  | **1 year** | | | |  | **5 years** | | | |  | **10 years** | | | |
|  | **%** | **95%CI** | | |  | **%** | **95%CI** | | |  | **%** | **95%CI** | | |  | **%** | **95%CI** | | |  | **%** | **95%CI** | | |  | **%** | **95%CI** | | |
| **Anxiety** | 2.0 | 1.9 | - | 2.1 |  | 8.2 | 8.1 | - | 8.4 |  | 13.8 | 13.6 | - | 14.0 |  | 3.9 | 3.8 | - | 4.1 |  | 10.6 | 10.3 | - | 10.9 |  | 16.4 | 15.9 | - | 16.8 |
| **Depression** | 4.0 | 3.9 | - | 4.0 |  | 14.8 | 14.6 | - | 15.0 |  | 24.0 | 23.7 | - | 24.3 |  | 7.1 | 6.9 | - | 7.3 |  | 19.4 | 19.0 | - | 19.8 |  | 28.5 | 28.0 | - | 29.1 |
| **Cognitive dysfunction** | 1.1 | 1.1 | - | 1.2 |  | 5.8 | 5.7 | - | 5.9 |  | 13.2 | 12.9 | - | 13.4 |  | 1.0 | 0.9 | - | 1.1 |  | 5.7 | 5.4 | - | 5.9 |  | 13.1 | 12.7 | - | 13.6 |
| **Fatigue** | 2.7 | 2.6 | - | 2.8 |  | 11.4 | 11.3 | - | 11.6 |  | 19.9 | 19.7 | - | 20.2 |  | 4.0 | 3.8 | - | 4.1 |  | 15.2 | 14.8 | - | 15.5 |  | 23.9 | 23.4 | - | 24.5 |
| **Pain** | 22.1 | 21.9 | - | 22.4 |  | 61.0 | 60.7 | - | 61.3 |  | 79.1 | 78.8 | - | 79.4 |  | 27.0 | 26.6 | - | 27.5 |  | 69.7 | 69.2 | - | 70.3 |  | 85.6 | 85.1 | - | 86.2 |
| **Sexual dysfunction** | 0.2 | 0.2 | - | 0.2 |  | 0.8 | 0.8 | - | 0.9 |  | 1.5 | 1.4 | - | 1.5 |  | 0.2 | 0.2 | - | 0.3 |  | 1.2 | 1.1 | - | 1.4 |  | 2.0 | 1.8 | - | 2.1 |
| **Sleep disorder** | 1.6 | 1.5 | - | 1.6 |  | 6.6 | 6.5 | - | 6.8 |  | 11.6 | 11.4 | - | 11.7 |  | 4.7 | 4.6 | - | 4.9 |  | 10.8 | 10.5 | - | 11.1 |  | 16.0 | 15.5 | - | 16.4 |
| **Opioid analgesics** | 4.2 | 4.1 | - | 4.3 |  | 17.7 | 17.5 | - | 17.9 |  | 31.1 | 30.8 | - | 31.3 |  | 12.2 | 12.0 | - | 12.5 |  | 30.8 | 30.3 | - | 31.2 |  | 45.5 | 44.9 | - | 46.1 |
| **Fatal and non-fatal self-harm** | 0.1 | 0.1 | - | 0.1 |  | 0.5 | 0.5 | - | 0.6 |  | 0.9 | 0.8 | - | 1.0 |  | 0.1 | 0.1 | - | 0.2 |  | 0.5 | 0.5 | - | 0.6 |  | 0.9 | 0.8 | - | 1.0 |

95%CI: 95% confidence interval.
